# 2-Sulfonylpyrimidines as Privileged Warheads for the Development of *S. aureus* Sortase A Inhibitors

**DOI:** 10.3389/fmolb.2021.804970

**Published:** 2022-01-03

**Authors:** Fabian Barthels, Jessica Meyr, Stefan J. Hammerschmidt, Tessa Marciniak, Hans-Joachim Räder, Wilma Ziebuhr, Bernd Engels, Tanja Schirmeister

**Affiliations:** ^1^ Institute for Pharmaceutical and Biomedical Sciences, Johannes Gutenberg-University, Mainz, Germany; ^2^ Institute of Physical and Theoretical Chemistry, Julius-Maximilians-University of Würzburg, Würzburg, Germany; ^3^ Institute for Molecular Infection Biology, Julius-Maximilians-University of Würzburg, Würzburg, Germany; ^4^ Max Planck Institute for Polymer Research, Mainz, Germany

**Keywords:** covalent inhibition, sortase, quantum mechanics, anti-virulence, drug discovery

## Abstract

*Staphylococcus aureus* is one of the most frequent causes of nosocomial and community-acquired infections, with emerging multiresistant isolates causing a significant burden to public health systems. We identified 2-sulfonylpyrimidines as a new class of potent inhibitors against *S. aureus* sortase A acting by covalent modification of the active site cysteine 184. Series of derivatives were synthesized to derive structure-activity relationship (SAR) with the most potent compounds displaying low micromolar K_I_ values. Studies on the inhibition selectivity of homologous cysteine proteases showed that 2-sulfonylpyrimidines reacted efficiently with protonated cysteine residues as found in sortase A, though surprisingly, no reaction occurred with the more nucleophilic cysteine residue from imidazolinium-thiolate dyads of cathepsin-like proteases. By means of enzymatic and chemical kinetics as well as quantum chemical calculations, it could be rationalized that the *S*_*N*_Ar reaction between protonated cysteine residues and 2-sulfonylpyrimidines proceeds in a concerted fashion, and the mechanism involves a ternary transition state with a conjugated base. Molecular docking and enzyme inhibition at variable pH values allowed us to hypothesize that in sortase A this base is represented by the catalytic histidine 120, which could be substantiated by QM model calculation with 4-methylimidazole as histidine analog.

## 1 Introduction

The emergence of bacterial strains resistant to antibiotic therapy is one of the greatest medical challenges of our time. In addition to conventional antibiotics, there are efforts to develop drugs that can interfere with the virulence mechanisms of bacteria to reduce their pathogenicity (Allen et al., 2014; Dickey et al., 2017). The cysteine transpeptidase sortase A (SrtA) has been discussed as an anti-virulence drug target for nearly 20 years since SrtA mediates the attachment of virulence-associated surface proteins to the bacterial cell wall (Perry et al., 2002). It was shown that the *S. aureus* ΔSrtA knock-out mutant is attenuated in mouse infection models compared to the wild type (Mazmanian et al., 2000; Weiss et al., 2004). Neither genetic deletion (Mazmanian et al., 1999) nor selective chemical inhibition (Cascioferro et al., 2014; Zhang et al., 2014; Mu et al., 2018) of *S. aureus* SrtA was found to affect the growth properties of bacterial cells, thus deducing a lower selective pressure for resistance development compared to bactericidal antibiotics.

At the bacterial cell wall, virulence-associated surface proteins with C-terminal LPXTG-tagged sorting signals (e.g., protein A, fibronectin-binding proteins, clumping factors) are cleaved between threonine and glycine by the membrane-anchored SrtA and subsequently ligated to the pentaglycine tail of the peptidoglycan layer to yield covalent attachment of these surface proteins (Tsompanidou et al., 2012; Schneewind and Missiakas, 2019). The fact that SrtA plays a key role in the pathogenesis of *S. aureus* and the enzyme is drug-accessible on the outside of the bacterial cell membrane makes SrtA seemingly a well-druggable target for the development of anti-virulence agents (Cascioferro et al., 2015).

However, the privileged position of SrtA is protected from pharmacological manipulation by several structural and biochemical properties of this enzyme. *S. aureus* SrtA is an eight-stranded β-barrel protein with three conserved catalytic residues: His^120^, Cys^184^, and Arg^197^, each of which cannot be mutated without disrupting enzymatic functionality (Ton-That et al., 2002; Bentley et al., 2008; Clancy et al., 2010). Three SrtA characteristics were identified that complicate the development of small molecule SrtA inhibitors:

1) The catalytic Cys^184^ is “reversely protonated,” which means it does not form a thiolate-imidazolium pair under physiological conditions (<0.1%) and is therefore significantly less nucleophilic than structurally related cysteine proteases such as enzymes of the papain family (Ilangovan et al., 2001; Frankel et al., 2005). Hence, covalent modification of the catalytic Cys^184^ is inefficient with cysteine protease-specific warheads such as Michael acceptors and similar electrophiles (Ton-That and Schneewind, 1999; Scott et al., 2002; Connolly et al., 2003; Kruger et al., 2004a; Liew et al., 2004).
2) The active site is predominantly defined by the intrinsic flexibility of the β6/7- and β7/8-loops (Kappel et al., 2012). Binding of the LPXTG-substrate leads to strong protein rigidization, observable from the NMR structure, and is therefore entropically penalized, which is expressed in the substrates’ high K_M_ value of 5.5 mM (Kruger et al., 2004b; Suree et al., 2009a).
3) To compensate for the low activity and the high KM value, both the LPXTG- and the pentaglycine-substrate are co-localized with the SrtA enzyme at the bacterial outer membrane (Ton-That et al., 1999). Spatial co-localization yields high local concentrations, which cannot easily be competed with reversible-competitive inhibitors *in cellulo.* Hence, most active compounds were found to be irreversible covalent inhibitors containing an electrophilic warhead that reacts with the active-site Cys^184^ of SrtA, such as disulfides, benzisothiazolinones, thiadiazolidine-3,5-diones, and 1,3,4-thiadiazoles (Maresso et al., 2007; Suree et al., 2009b; Zhulenkovs et al., 2014; Chan et al., 2017; Wehrli et al., 2019; Barthels et al., 2020; Yang et al., 2020).

Previously, Jaudzems et al. have performed a high-throughput screening with a library of 50,000 compounds and identified 27 novel covalent modifiers of SrtA (Jaudzems et al., 2020). One of these inhibitors, parent compound sulfonylpyrimidine **4** (Table 1), showed 97% inhibition after 16 h of incubation with 100 µM inhibitor. The irreversibility of the reaction was confirmed by 2D^15^N-^1^H HSQC NMR, however, characterization of the inhibition kinetics and detection of the covalent adduct were still to be determined.

**TABLE 1:** Inhibition constants (K_I_, *k*_inact_, *k*_2nd_) of the compounds **4** and **5a–k** for *S. aureus* SrtA; n. i. = <20% inhibition at 100 µM.

Cpd.	Structure	K_I_ [µM]	*k*_inact_ [s^−1^]	*k*_2nd_ [M^−1^min^−1^]
4	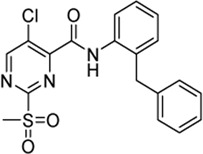	47.1 ± 15.7	0.00065 ± 0.00007	870 ± 170
5a	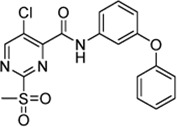	57.6 ± 3.55	0.00774 ± 0.00007	8,088 ± 464
5b	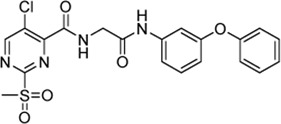	42.37 ± 4.62	0.00082 ± 0.00004	1,175 ± 67
5c	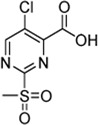	n. i.		
5d	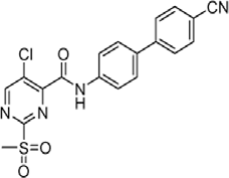	n. i.		
5f	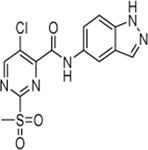	n. i.		
5g	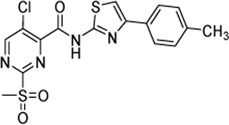	n. i.		
5h	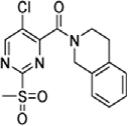	n. i.		
5i	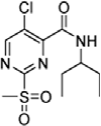	n. i.		
5k	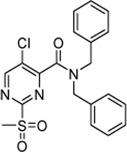	n. i.		

The use of 2-sulfonylpyrimidines as electrophilic building blocks has been documented in the synthesis of heterocyclic compounds for nearly 100 years (Sprague and Johnson, 1936). In a more biological context, heteroaromatic methyl sulfones, such as 2-methylsulfonyl benzothiazole, are well established as bioorthogonal reagents for the selective modification of non-catalytic cysteine residues (Zhang et al., 2012). Regardless of the knowledge of this potential cysteine reactivity, 2-sulfonylpyrimidines have been previously considered almost exclusively as reversible modulators of pharmacological targets during drug design campaigns. Besides the studies by Jaudzems et al., 2-sulfonylpyrimidines have just recently come into the spotlight for mechanistic studies of irreversible inhibition. In the last few years, four independent studies identified 2-sulfonylpyrimidines as covalent and pharmacologically active modifiers of the following disease-associated protein targets: p53 (Bauer et al., 2016), kinesin HSET (Förster et al., 2019), succinate dehydrogenase subunit B (Li L. et al., 2017), and *S. mansoni* thioredoxin glutathione reductase (Lyu et al., 2020). All these targets share a covalently addressed cysteine residue, that is not catalytically activated and thus predominantly protonated under physiological conditions (Hallenbeck et al., 2017).

A preference for the reactivity with protonated thiols is also reflected in the ability of sulfonylpyrimidine-mediated glutathione depletion in a cellular context (Bauer et al., 2016; Wilke et al., 2018). However, compared to other electrophilic warheads, which react quantitatively with protonated cysteine residues under physiological conditions, the second-order rate constant (60–200 M^−1^min^−1^) is lower by a factor of 10–1,000 and thus 2-sulfonylpyrimidines may have the potential to be mild enough to achieve favorable target *vs.* off-target selectivity (Schoonen et al., 2005; Böhme et al., 2009; Chipinda et al., 2010; Wilke et al., 2018).

**FIGURE 1:**
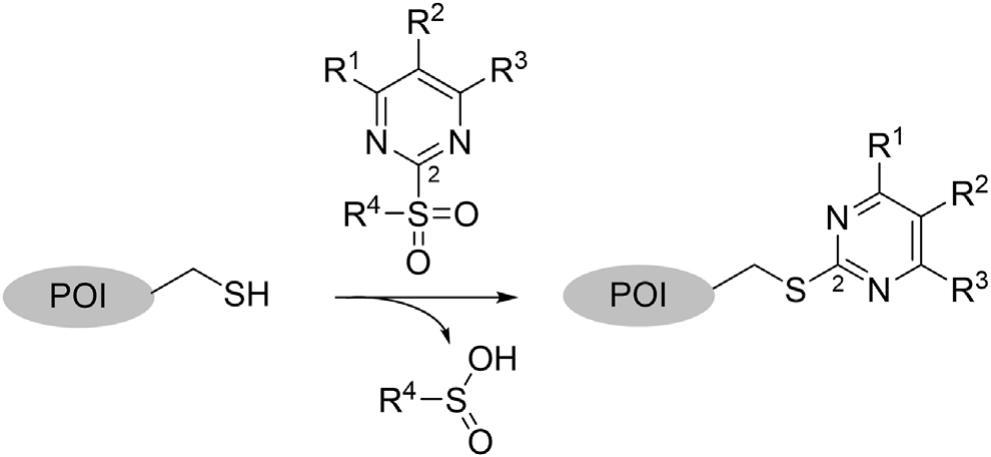
Covalent Reaction of 2-sulfonylpyrimidines. Reaction mechanism of 2-sulfonylpyrimidines with cysteine residues of a respective protein of interest (POI) yielding a covalent 2-pyrimidyl cysteine adduct. The electrophilic C-2 atom was highlighted with a small numeral “2.”

2-Sulfonylpyrimidines react with thiols under nucleophilic aromatic substitution (*S*_*N*_Ar) to form the respective pyrimidyl thioether and release the corresponding sulfinic acid (Bauer et al., 2016). Electron-withdrawing substituents on the aromatic ring (R^1^–R^3^) increase the polarization of the electrophilic C-2 atom and generally lead to higher reaction rates (Buděšínský and Vavřina, 1972). A schematic representation of the reaction mechanism between 2-sulfonylpyrimidines and proteinogenic cysteine residues is shown in Figure 1.

In this work, evidence of the covalent modification by 2-sulfonylpyrimidines, as well as the characterization of the SrtA inhibition kinetics were conducted in following up on the previously reported parent compound **4** (Jaudzems et al., 2020)*.* Further, by using methods from molecular biology, enzyme kinetics, and quantum chemical calculations, we aimed to elucidate the underlying reaction mechanism of the 2-sulfonylpyrimidine warhead with cysteine residues, which might help to explain the mild yet efficient reactivity towards protonated thiols.

## 2 Results and Discussion

### 2.1 Kinetic Characterization and Inhibitor Optimization Strategy

To evaluate the inhibition potency of the parent sulfonylpyrimidine **4**, this compound was tested by means of a fluorometric enzyme assay with recombinantly expressed *S. aureus* SrtA (Schmohl et al., 2017b) and Abz-LPETG-Dap (Dnp)-OH as substrate. Parent compound **4** was found to act as a time-dependent and irreversible inhibitor, which is in agreement with the literature data (Lit.: 97% inhibition after 16 h of incubation with 100 µM inhibitor; Jaudzems et al., 2020). To verify the irreversible mode of inhibition, we determined the maximum inactivation rate *k*_inact_, the dissociation constant of the reversible enzyme-inhibitor complex K_I_, and the second-order rate of inhibition *k*_2nd_. For compound **4**, a *k*_inact_ = 0.00065 s^−1^ was found, which is very low compared to other covalent SrtA inhibitors (Chan et al., 2017; Barthels et al., 2020; Jaudzems et al., 2020). The K_I_ value was as high as 47.1 µM, thus, we decided to increase the potency of the sulfonylpyrimidine inhibitors by rational optimization before conducting mechanistic studies of this new inhibitor class.

In order to increase the affinity of the sulfonylpyrimidine inhibitors, a first strategy was applied to optimize the recognition sequence while maintaining the warhead functionality. Previously, we have performed a recognition sequence optimization study on disulfanylbenzamide SrtA inhibitors (Barthels et al., 2020). Therefore, we designed and tested two chimeric inhibitors (**5b,c** Supplementary Figure S1). In general, sulfonylpyrimidines were synthesized based on multi-step procedures according to literature protocols. Detailed synthesis instructions can be found in the supporting information. Briefly, 2-(methanesulfanyl)pyrimidinecarboxylic acids (**2a–d**) were coupled to various commercially available or synthesized amines (**1a–o**) in the presence of 2-(1*H*-benzotriazole-1-yl)-1,1,3,3-tetramethylaminium tetrafluoroborate (TBTU) to provide the inhibitor scaffold precursors (**3a–z**). The potential sulfonylpyrimidine inhibitors (**4, 5a–k, 6a–n, 7a–e**) were subsequently synthesized by oxidation with potassium peroxymonosulfate (Webb, 1994).

The chimeric 3-phenoxyphenyl glycine derivative (**5b**) showed slightly higher overall inhibition potency (*k*_2nd_ = 1,175 M^−1^min^−1^) than parent compound **4** (Table 1). In contrast, the adapted fragment inhibitor (**5c**) showed no SrtA inhibition even at a final inhibitor concentration of 300 µM. From the inhibition results, we concluded that the binding geometry of the previously optimized disulfanylbenzamide recognition sequence (**5b**, Supplementary Figure S1) is probably not optimally transferable to the novel sulfonylpyrimidine derivates because of the different aromatic substitution pattern of the warheads’ electrophilic center (ortho vs*.* meta).

**FIGURE 2:**
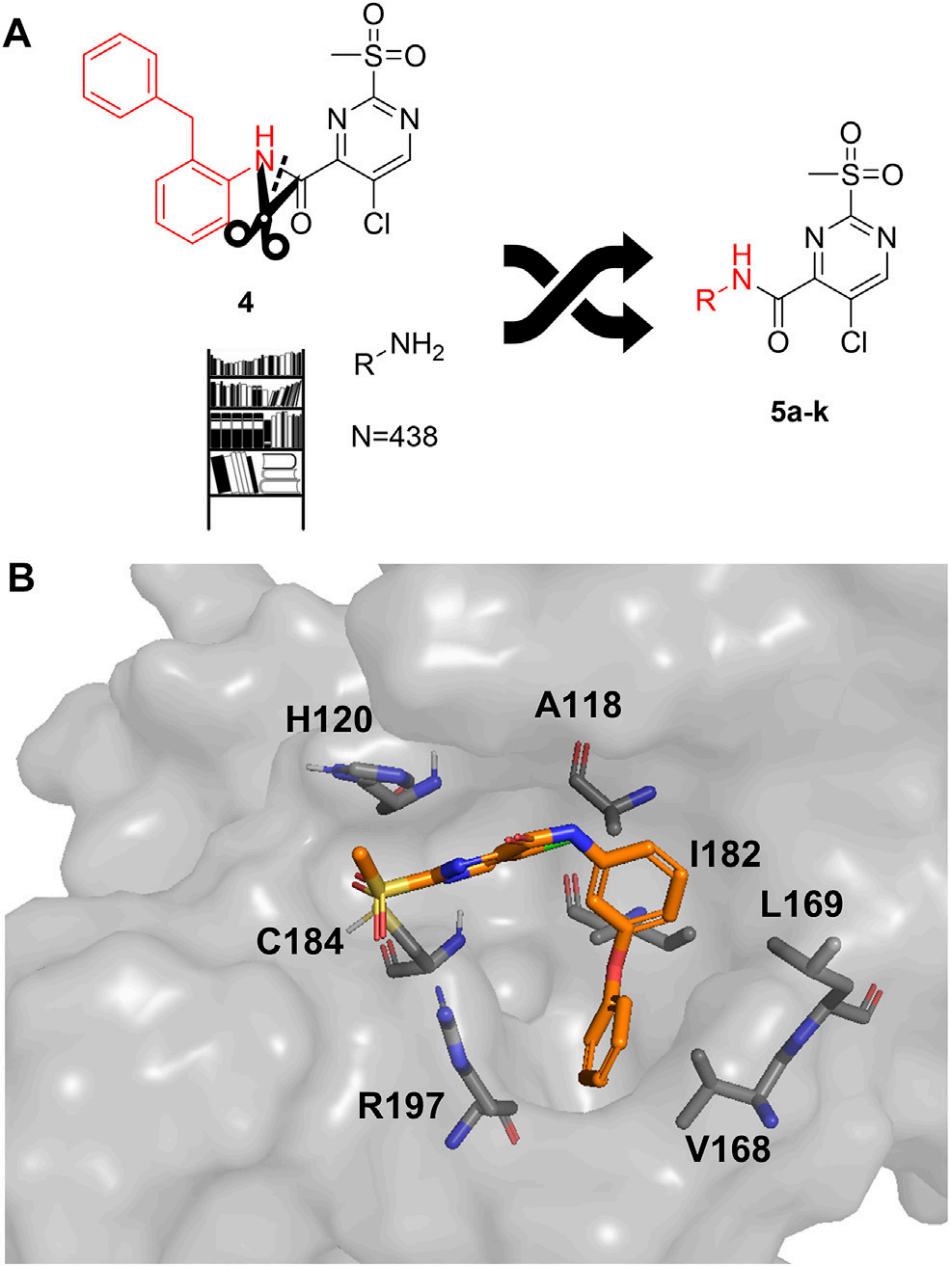
Optimization strategy overview. **(A)** Virtual synthesis strategy to optimize the inhibition properties of parent compound **4** by synthesis of the sulfonylpyrimidines **5a–k**. Carboxylic acid **5c** was coupled *in silico* with 438 amines and the resulting amides were docked into a receptor of the SrtA active site. Subsequently, virtual screening hits **5a–k** were synthesized. **(B)** Docking pose of virtual screening hit **5a** with the labeling of interacting residues in the SrtA binding pocket (pdb: 2kid).

As a second attempt, we tried an *in silico* supported optimization approach (Figure 2A): Using virtual synthesis, we combined the warhead function of parent compound **4** with our in-house inventory of aliphatic and aromatic amines (N = 438). Subsequently, molecular docking to the binding pocket of SrtA was carried out using the Glide docking algorithm (Friesner et al., 2004). Potential binders were discriminated in this virtual screening approach by a distance scan of the electrophilic C-2 atom in the pyrimidine ring from the catalytic Cys^184^ in the binding pocket. Structures that showed a distance of less than 5 Å were sorted according to their docking score and manual inspection of the binding pose. The seven best sulfonylpyrimidines were synthesized for *in vitro* evaluation (**5a,d–k**).

The exchange of the 2-benzylaniline fragment (**4**) for the 3-phenoxyaniline fragment (**5a**) led to a significant improvement of the overall inhibition potency by a factor of 10 (Table 1, *k*_2nd_ = 8,088 M^−1^min^−1^). The docking pose of **5a** with putative interactions in the SrtA binding pocket can be seen in Figure 2B. Here, the sulfonylpyrimidine warhead is positioned orthogonally to the axis of the three catalytic residues (His^120^, Cys^184^, Arg^197^) and is thus reasonably positioned for a nucleophilic attack of the Cys^184^ thiol to the pyrimidine ring. The 3-phenoxyaniline moiety fits well to the L-shaped binding pocket of SrtA and shows interactions with the hydrophobic residues (Ala^118^, Val^168^, Leu^169^, Ile^182^) in the S3/S4 substrate binding pockets.

**FIGURE 3:**
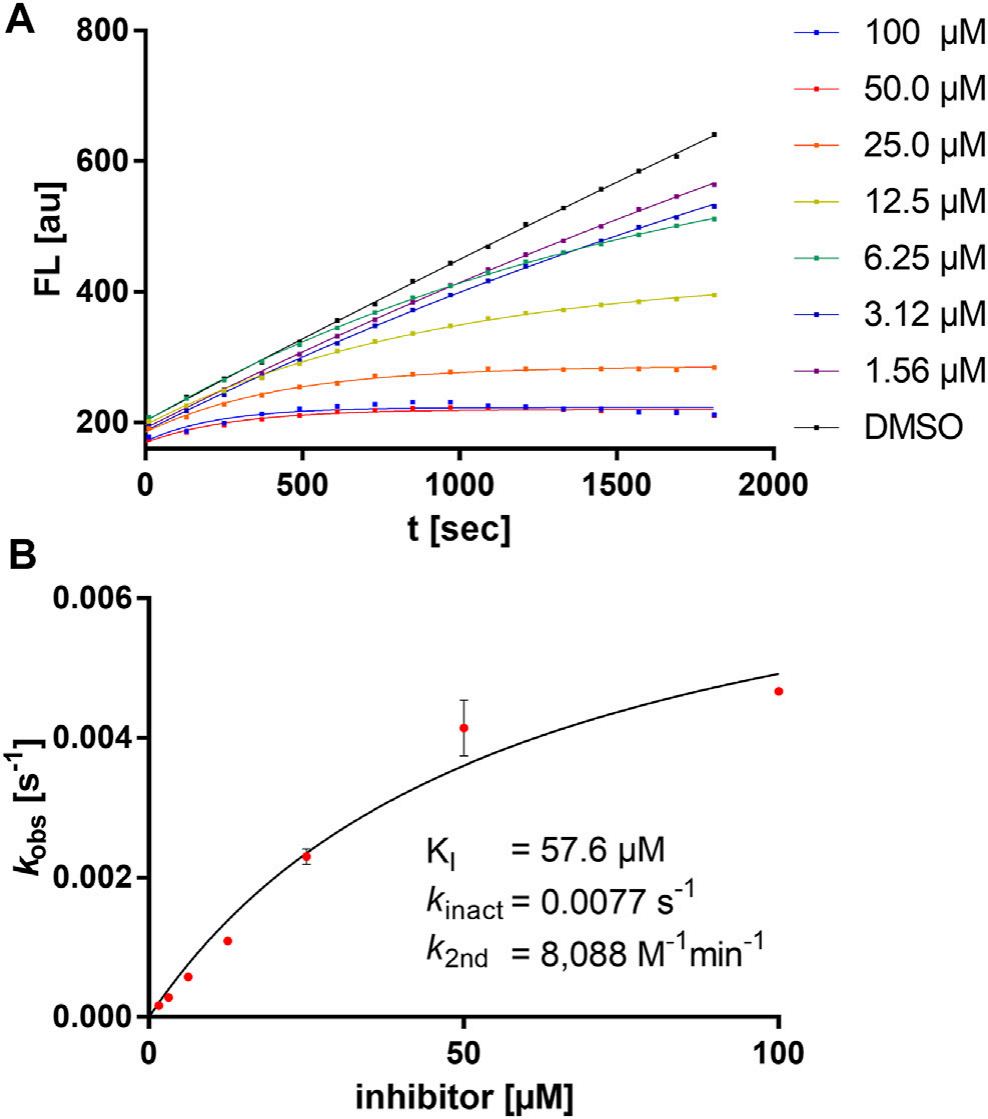
Kinetic characterization of compound **5a**. **(A)** Fluorometric assay with compound **5a** showing time-dependent enzyme inhibition with hyperbolic substrate conversion plots. The fluorescence was recorded for 30 min every 30 s. For clarity, only every fourth data point is shown. **(B)** *k*_obs_ vs. (I) for the determination of inhibition constants (K_I_, *k*_inact_).

Exemplarily, the substrate conversion plot with time-dependent inhibition is shown in Figure 3. The apparent first-order rate constant (*k*_obs_) in the presence of inhibitor **5a** varied hyperbolically with the concentration of the inhibitor. A limiting value was approached asymptotically at higher inhibitor concentrations indicating two-step mechanism inactivation kinetics.

The other virtual screening hits (**5d–k**) were also assayed using the fluorometric enzyme assay at an inhibitor concentration of 100 µM but showed no measurable inhibition of SrtA (Table 1). For achieving warhead selectivity towards off-targets, such a strong dependence on a recognition sequence for a covalent inhibitor might be desirable (Copeland, 2005; Awoonor-Williams and Rowley, 2018). The inhibition data and structures of the sulfonylpyrimidines **4** and **5a–k** are summarized in Table 1.

### 2.2 SAR Study Around Hit Compound 5a

The subtle structural transformation from 2-benzylaniline derivate (**4**) to 3-phenoxyaniline derivate (**5a**) resulted in a tenfold increase of the inhibitory potency (*k*_2nd_ = 870 vs. 8,088 M^−1^min^−1^), hence, we hypothesized that the inhibitor’s recognition sequence can be further systematically optimized. Therefore, we conducted a SAR study around virtual screening hit **5a**. The inhibition results of the synthesized compounds **6a–n** are shown in Table 2.

**TABLE 2:** Inhibition constants (K_I_, *k*_inact_, *k*_2nd_) of the compounds **6a–n** for *S. aureus* SrtA; n. i. = <20% inhibition at 100 µM.

Cpd.	Structure	K_I_ [µM]	*k*_inact_ [s^−1^]	*k*_2nd_ [M^−1^min^−1^]
6a	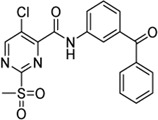	29.6 ± 9.39	0.00986 ± 0.00128	20,785 ± 4,074
6b	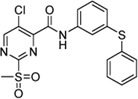	47.5 ± 4.62	0.00578 ± 0.00033	7,305 ± 294
6c	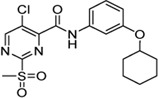	78.8 ± 9.92	0.00864 ± 0.00092	6,593 ± 243
6d	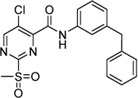	60.3 ± 6.40	0.00618 ± 0.00025	6,181 ± 489
6e	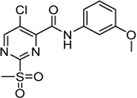	—	—	882 ± 43
6f	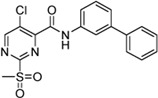	—	—	588 ± 48
6g	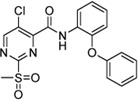	n. i.		
6h	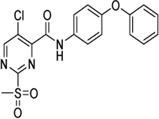	n. i.		
6i	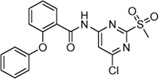	n. i.		
6k	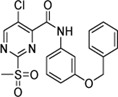	n. i.		
6l	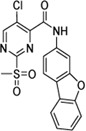	n. i.		
6m	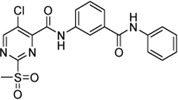	n. i.		
6n	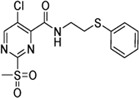	n. i.		

Six out of 13 sulfonylpyrimidines (**6a–f**) did inhibit SrtA in the fluorometric enzyme assay, but with varying potency. Strikingly, we observed a drop in SrtA inhibition for most modifications on the 3-phenoxyaniline motif. These structural “activity cliffs” highlight the importance of the accurate positioning of the warhead towards the catalytic Cys^184^ for a successful covalent reaction (Bajorath, 2019).

Only small changes in inhibition potency were observed upon changes of the aryl ether connecting group of compound **5a** to the analogous aryl thioether (**6b**) or the methylene derivate (**6d**). However, the exchange to a ketone connecting unit (**6a**) resulted in a significant increase of inhibition potency which might be due to beneficial interactions with Arg^197^. Based on the *k*_2nd_ value, compound **6a** (*k*_2nd_ = 20,785 M^−1^min^−1^) appeared to be 2.5-fold more potent than virtual screening hit **5a**. Alteration of the *meta*-substitution pattern resulted in the complete abolition of inhibition for the *ortho* (**6g**) and *para* (**6h**) derivatives. After truncation of the connecting group to the biphenyl derivative (**6f**), only minor inhibition potency remained (*k*_2nd_ = 588 M^−1^min^−1^). On the other hand, with connector elongation to the benzyl ether (**6k**) or an amide derivate (**6m**), the inhibition was completely abolished.

Modifications of the terminal phenyl ring were well tolerated in the case of an exchange to an aliphatic cyclohexyl ring (**6c**, *k*_2nd_ = 6,593 M^−1^min^−1^) but resulted in a severe loss of inhibition when downsized to a methoxy group (**6e**, *k*_2nd_ = 882 M^−1^min^−1^). Other transformations such as amide inversion at the sulfonylpyrimidine ring (**6i**), rigidization to a benzofuran derivative (**6l**), or decyclization of the central phenyl ring (**6n**) resulted in complete loss of inhibition. For derivatives **6e,f**, two-step kinetics could not be confirmed because of a lacking *k*_obs_ saturation at higher inhibitor concentrations. The K_I_ values of these compounds were estimated to be significantly higher than 100 µM. In these cases, the inhibition potency (*k*_2nd_ value) was determined by Lineweaver-Burk linearization.

In addition to the modification of the recognition sequence, the chlorine substituent on the sulfonylpyrimidine ring was also varied and derivatives **7a–e** were synthesized (Table 3).

**TABLE 3:** Inhibition constants (K_I_, *k*_inact_, *k*_2nd_) of the compounds **7a–e** for *S. aureus* SrtA; n. i. = <20% inhibition at 100 µM.

Cpd.	Structure	K_I_ (µM)	*k*_inact_ (s^−1^)	*k*_2nd_ (M^−1^min^−1^)
7a	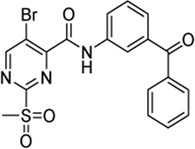	32.0 ± 5.07	0.01493 ± 0.00132	28,185 ± 2,117
7b	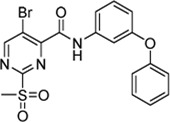	55.9 ± 11.6	0.01285 ± 0.00147	13,998 ± 1,462
7c	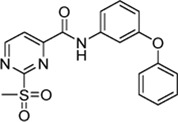	33.8 ± 11.2	0.00239 ± 0.00047	4,364 ± 596
7d	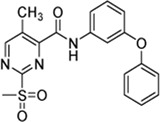	—	—	1,204 ± 139
7e	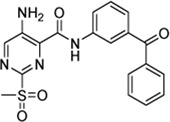	n. i.		

**FIGURE 4:**
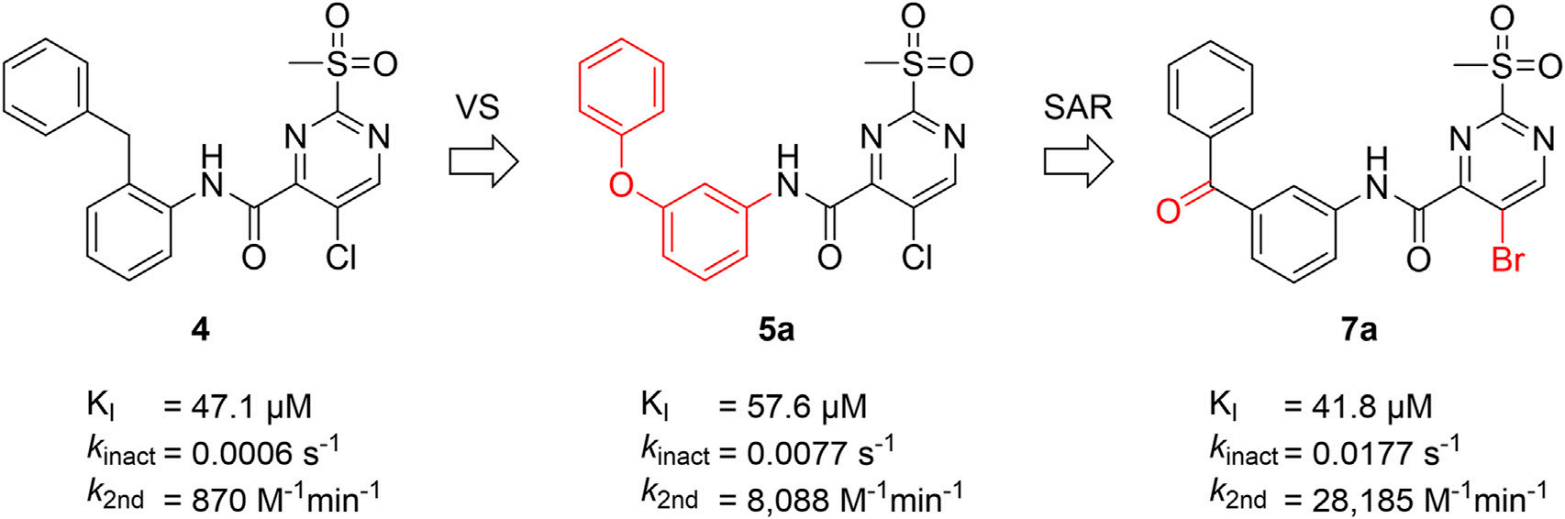
Summary of ligand optimization. Summary and inhibition data of the optimization strategy starting from literature compound **4** *via* virtual screening hit **5a** to yield lead structure **7a**.

A positive correlation was found between the intensity of the electron-withdrawing properties of the substituents and the inhibition potency: Br (**7b**) > Cl (**5a**) > H (**7c**) > CH_3_ (**7d**) >> NH_2_ (**7e**). The increase in inhibition was mainly caused by an increase in the *k*_inact_ value and was not due to affinity changes for the target (K_I_ value). The electronic effects of the substituents on the pyrimidine ring predominantly influenced the warhead reactivity, which can be quantified and is discussed by the analysis of the Hammett constants (Section 2.7). In summary, starting from virtual screening hit **5a**, two SAR transformations proved to be beneficial: Conversion of the aryl ether connecting group to the ketone derivative **6a** and the halogen exchange at the pyrimidine ring from chlorine to bromine derivative **7b**. Subsequently, the two modifications were combined and inhibitor **7a** was synthesized. Considering the *k*_2nd_ value, **7a** was the most potent irreversible inhibitor of this study (*k*_2nd_ = 28,185 M^−1^min^−1^). The optimization strategy is summarized in Figure 4.

### 2.3 Identification of Covalent Adducts by Mass Spectrometry

**FIGURE 5:**
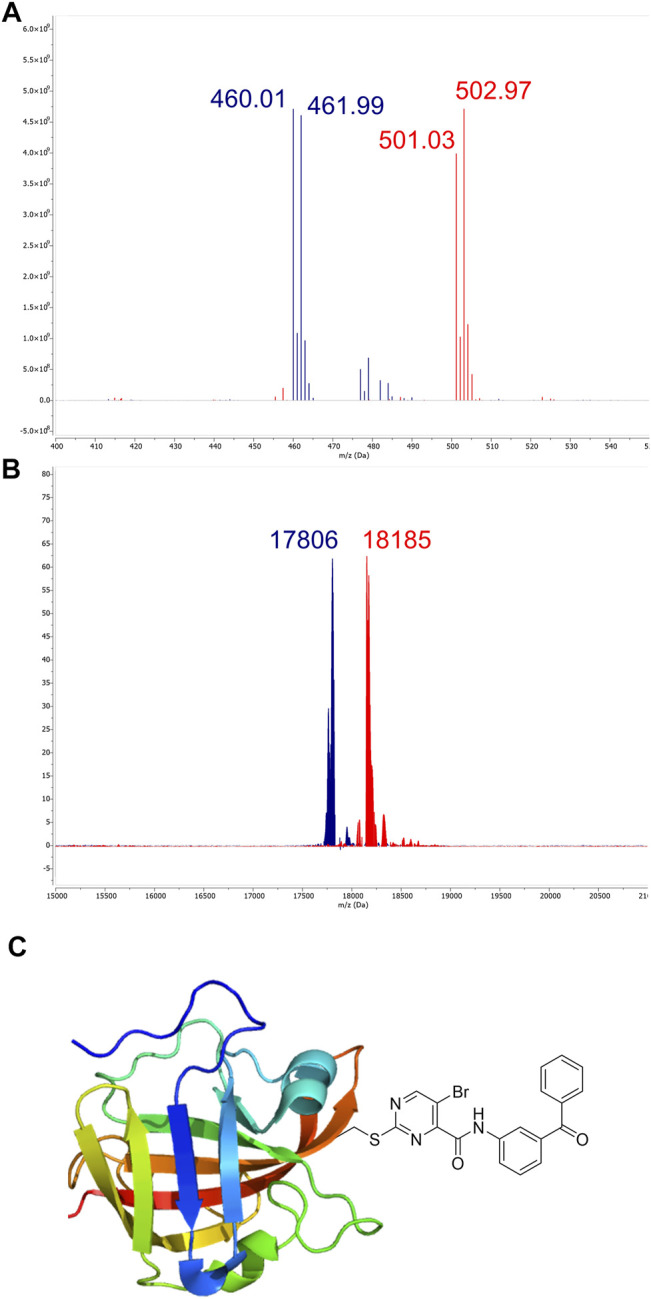
Identification of covalent adducts by mass spectrometry. **(A)** ESI-MS of compound **7a** (blue, m/z = 460.01) treated with free cysteine revealed the formation of a distinct adduct (red, m/z = 501.03) corresponding to the respective 2-pyrimidyl cysteine (see the mechanism in Panel 1). **(B)** MALDI-TOF-MS of SrtA (blue, m/z = 17,806) treated with compound **7a**. The peak at m/z = 18,185 (red) corresponds to the modification of the active site Cys^184^ by compound **7a**. **(C)** The predominant mode of SrtA inhibition is the transfer of the pyrimidyl-fragment to Cys^184^.

For electron-deficient heteroaromatic compounds, *S*_*N*_Ar reactions often occur at the aromatic carbon atom with the substituent representing the best leaving group (Rohrbach et al., 2019). For 2-sulfonylpyrimidines, this is generally the sulfonyl group (Buděšínský and Vavřina, 1972; Bauer et al., 2016; Förster et al., 2019). However, the scaffold used here also carried a halogen substituent which, depending on the reaction conditions, might represent the actual leaving group during organic synthesis (Buděšínský and Vavřina, 1972). Thus, we determined the point of nucleophilic thiol attack by means of ESI mass spectrometry between the reaction of compound **7a** and cysteine in phosphate buffered solution at pH 7.5. The reaction between compound **7a** (100 µM) and cysteine (100 µM) occurred quantitatively in less than 10 min to form the 2-pyrimidyl cysteine adduct (m/z = 501.03) as described by the mechanism in Figure 1. As expected, the isotope pattern specific for bromine (m/z = 502.97) was preserved for the adduct mass (Figure 5A). Similarly, for the chlorine-substituted compound **5c**, the analogous reaction adduct could be detected by LC/MS (Supplementary Figure S2). Accordingly, it was confirmed that under physiological conditions, the sulfonyl group was the leaving group and stable 2-pyrimidyl adducts were formed.

By using MALDI-TOF mass spectrometry, we aimed to determine the mode of inhibitory action in an enzymatic context. For the native SrtA, we found a protein mass at m/z = 17,806. Treatment with inhibitor **7a** resulted in quantitative conversion and showed a single mass peak at m/z = 18,185 (Figure 5B), which matches the corresponding 2-pyrimidyl adduct and proves the covalent inhibition mechanism (Figure 5C).

### 2.4 Protease Inhibition Selectivity

The sulfonylpyrimidines studied in this work were characterized as mild covalent inactivators of the SrtA enzyme (Table 3 and Figure 5) and exhibited a strong scaffold dependence for their inhibitory potency (Table 2). To investigate how these sulfonylpyrimidines affect the activity of other cysteine and serine proteases, a selectivity panel was constructed from a matrix of 6 diverse sulfonylpyrimidines and 6 different proteases. The compounds were assayed at a final compound concentration of 100 µM for the cysteine proteases human cathepsin B (hCatB), cathepsin L (hCatL), and *T. brucei* rhodesain (Rd). Furthermore, the serine proteases NS2B-NS3 from Zika virus (ZIKV), the homologous protease from Dengue virus (DENV), and the human protease urokinase plasminogen activator (uPA) were tested for their susceptibility. The results of the inhibition data are summarized in Table 4.

**TABLE 4:** Protease inhibition selectivity of a representative compound set. Compounds were tested at a final concentration of 100 µM inhibitor. The inhibition is expressed relative to the DMSO control in (%); n. i. = no inhibition at 100 µM.

Cpd	Structure	SrtA (%)	CatB (%)	CatL (%)	Rd	ZIKV (%)	DENV	uPA
5a	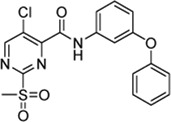	99.0	6.6	8.8	24.6%	6.4	n. i.	23.0%
5c	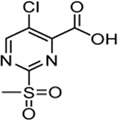	19.6	5.8	6.6	20.0%	7.7	n. i.	n. i.
6a	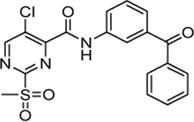	98.4	5.6	18.0	n. i.	18.6	5.1%	2.2%
6g	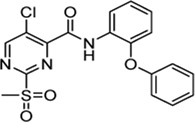	21.8	11.8	29.4	15.0%	18.3	2.6%	67.0%
6i	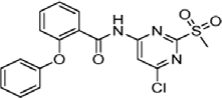	36.0	9.5	21.3	9.2%	29.3	15.0%	59.4%
7b	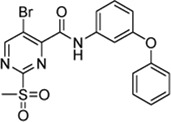	99.2	12.5	28.5	4.7%	18.3	5.2%	11.2%

Cathepsin B/L, rhodesain, and SrtA are all structurally related to papain-like proteases, thus, we used their relatedness to study the selectivity of our inhibitors (Bradshaw et al., 2015). The selected sulfonylpyrimidines appear to have good selectivity for SrtA, as the other three proteases were inhibited at most 29.4% by compound **6g**. A time-dependent inhibition, as with SrtA, could not be observed over the measurement duration of 30 min, which leads to the conclusion that sulfonylpyrimidines probably do not react covalently with cathepsin-related proteases.

A significant difference between cathepsin B/L, rhodesain, and SrtA is that in contrast to SrtA, the catalytically active cysteine is present in thiolate form in the first three proteases (Otto and Schirmeister, 1997; Ehmke et al., 2013; Klein et al., 2020b). This observation seems counterintuitive at first since the reversely protonated thiol of SrtA has a lower nucleophilicity than the thiolate in cathepsin-related proteases. The reaction of electrophiles such as sulfonylpyrimidines should react favorably with the thiolate as a stronger nucleophile (Zhang et al., 2012). A theoretical examination of these observations is provided later in this study through docking and quantum chemical calculations (Section 2.8).

The ZIKV NS2B/NS3 protease is a serine protease and contains two non-catalytic cysteine residues, thus as expected, only <30% inhibition at 100 µM inhibitor concentration was found. The related DENV NS2B/NS3 protease has no cysteine residues in its protein sequence and was inhibited by a maximum of only 15% (Li Y. et al., 2017). The urokinase plasminogen activator, which is also a serine protease, was inhibited by less than 25% by most compounds. Compound **6g**, however, showed 67% inhibition, but this inhibition was observed to be not time-dependent and thus presumably due to reversible binding.

### 2.5 Solvent Effects on SrtA Inhibition

**FIGURE 6:**
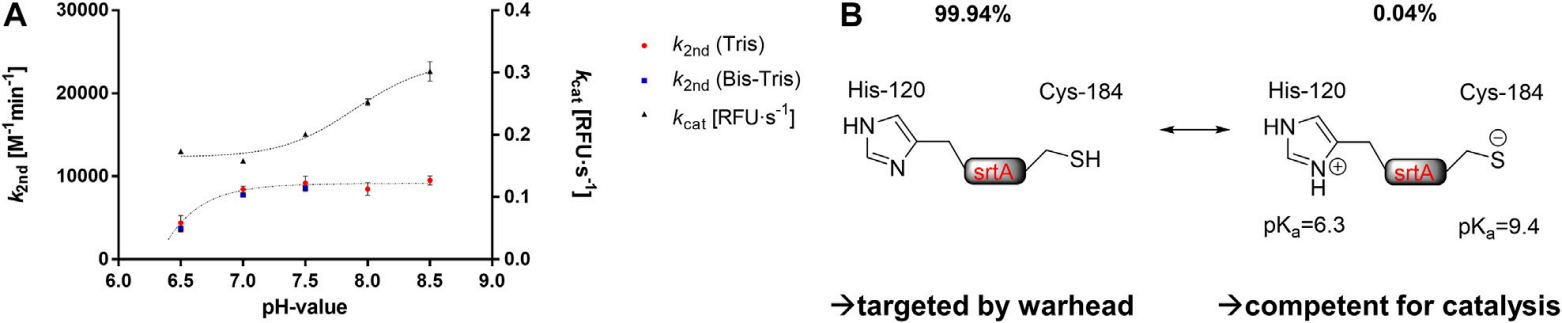
Buffer effects on SrtA inhibition. **(A)** pH- and buffer dependence of the enzymatic activity [*k*_cat_ (RFU·s^−1^)] and the inhibitory potency of compound **5a** [*k*_2nd_ (M^−1^min^−1^)]. **(B)** Hypothesis for the covalent inhibition mechanism by sulfonylpyrimidines. The catalytically competent form presumably differs from the warhead-targeted form in terms of the protonation of the catalytic dyad.

To investigate which thiol protomer (Cys-SH or Cys-S¯) of SrtA Cys^184^ is responsible for the reaction with sulfonylpyrimidines, enzyme assays were performed at different pH values and in different enzyme buffers. It is known that the catalytical Cys^184^ of SrtA is predominantly (99.94%) in the protonated thiol form under physiological conditions at pH 7.5 (van’t Hof et al., 2015). The pK_a_ value was previously determined to be 9.4 by NMR titration (Connolly et al., 2003). Thus, increasing the pH value of the enzyme solution above pH 7.5 resulted in a significant increase in the thiolate species content. This on one hand led to increased enzyme activity, on the other hand, made SrtA also more vulnerable for covalent modification by electrophiles such as Michael acceptors leading to an increase in the observed rate of irreversible enzyme inactivation (Connolly et al., 2003; Frankel et al., 2005; Schmohl et al., 2015; Barthels et al., 2020). Enzyme assays were performed at five pH values between 6.5 and 8.5 in Tris or Bis-Tris buffers within the possible buffer capacities. Both the relative enzyme activity *k*_cat_ (RFU·s^−1^) of the DMSO control and the inhibition potency *k*_2nd_ (M^−1^min^−1^) of compound **5a** were determined at each pH value (Figure 6A).

The observed activity increase of SrtA (*k*_cat_) at pH values above pH 7.5 is in agreement with the literature (Frankel et al., 2005; Barthels et al., 2020). However, the inhibitory potency of compound **5a** showed no significant differences at varying pH values between 7.0 and 8.5. This is contrary to the inactivation kinetics of known SrtA targeting warheads, which have been shown to react preferentially with the thiolate species (Connolly et al., 2003; Frankel et al., 2005; Schmohl et al., 2015; Barthels et al., 2020). We take this, in addition to the protease selectivity results (Table 4), as further evidence that sulfonylpyrimidines might react preferentially with protonated thiols in this proteinogenic context (Figure 6B). Albeit yet unclear, we aimed to elucidate the molecular background by *in vitro* and *in silico* methods. In addition to the cationic Tris and Bis-Tris buffers, inhibition experiments of SrtA by compound **5a** were also performed in the zwitterionic HEPES buffer at pH 7.5 (*k*_2nd_ = 7,956 M^−1^min^−1^). Hence, the inhibitory potency at pH 7.5 did not differ significantly between all three investigated buffers indicating no involvement of a buffer molecule for the reaction in an enzymatic context.

In contrast, when the pH value was lowered to 6.5, a substantial decrease in SrtA inhibitory potency was observed (pH 7.0: *k*_2nd_ = 8,436 M^−1^min^−1^; pH 6.5: *k*_2nd_ = 4,384 M^−1^min^−1^). However, the enzymatic activity (*k*_cat_) did not decrease as much with this pH change, which is in agreement with the literature reports (Zong et al., 2004). The reduction in inhibitory potency at pH < 7.0 must be due to other SrtA residues than Cys^184^ since the proportions of the thiol/thiolate ratio do not change significantly at pH 6.5–7.0. However, the pK_a_ value of the neighboring His^120^ is 6.3 leading to a significant protonation change when the pH is lowered from pH 7.0 to pH 6.5 (Connolly et al., 2003).

It is conceivable that the protonated imidazolinium form prevents the reaction of Cys^184^ with the sulfonylpyrimidine warhead. This would also hint at an explanation for the selectivity over the cysteine proteases with zwitterionic dyad (Table 4) since these have such an imidazolinium ion adjacent to their catalytic cysteine residue which might prevent reversible binding to the cathepsin B/L or rhodesain binding pockets (Mladenovic et al., 2007; Ehmke et al., 2013; Paasche et al., 2014).

### 2.6 In-Solution Cysteine Reactivity

Next, the reaction of sulfonylpyrimidines was studied kinetically in a non-proteinogenic context with the free amino acid cysteine as a nucleophile to gather additional evidence for the reaction mechanism of sulfonylpyrimidines with thiols.

**FIGURE 7:**
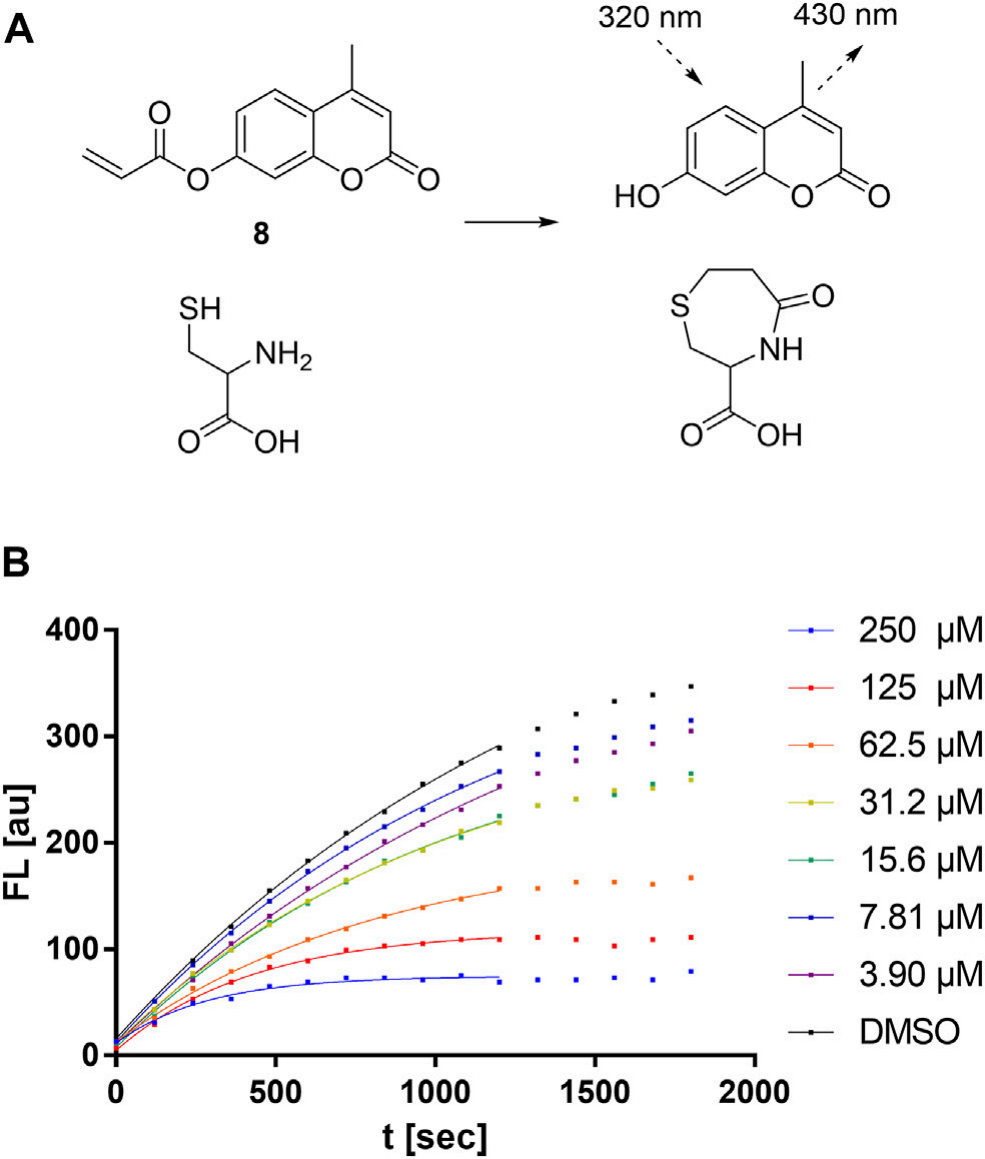
Cysteine reactivity assay. **(A)** Mechanism of the fluorescent probe **8** reacting with cysteine. **(B)** Cysteine reactivity assay with variable concentrations of compound **5a** showing hyperbolic substrate conversion plots. The fluorescence was recorded for 30 min every 30 s. For clarity, only every fourth data point is shown.

In order to study the kinetics of sulfonylpyrimidines reacting with free cysteine in solution, a strategy for a competitive fluorescence-based assay was adapted from previous works (Epps and Taylor, 2001; Sameshima et al., 2017). For this purpose, a novel cysteine-reactive probe **8** was synthesized, which, in terms of signal-to-noise ratio, was superior to the probes used in the previous cysteine assays (Zeng et al., 2017). The reaction of probe **8** with cysteine liberates 4-methylumbelliferone as a fluorometrically detectable product (Figure 7A), and thus, the fluorescence increase can be described by the second-order rate constant *k*_probe_ (Equation 1).

Upon the addition of competitive and irreversible cysteine modifiers such as sulfonylpyrimidines, the fluorescence increase of the probe reaction was hyperbolically attenuated (Figure 7B). According to the conceptual deduction described previously (Epps and Taylor, 2001; Sameshima et al., 2017), the experimental fluorescence curves can be analyzed by nonlinear regression as first-order reaction progress curves (Equation 2). The parameter *k*_obs_ describes the curvature corresponding to the sum of the competing kinetics (Equation 3). Under the assumptions that 1) the competitive reaction between sulfonylpyrimidines *vs.* probe with cysteine is started by simultaneous addition, and 2) sulfonylpyrimidines are added at a much higher concentration than cysteine (5 µM) and the probe (5 µM), the *k*_obs_ values depend substantially on the *k*_chem_ value. The compound-specific second-order inactivation parameter *k*_chem_ can be determined by linear regression from the *k*_obs_ values of the dilution series (Equation 4).

$$\frac{d\left[FL\right]}{dt}=k_{probe}\left[Cys\right]\left[probe\right]$$ (1)

$$FL=\frac{{\left[Cys\right]}_{total}\cdot k_{probe\;}\left[probe\right]}{k_{probe\;}\left[probe\right]\;+\;k_{chem\;}\left[sulfonylpyrimidine\right]}\left(1-e^{-k_{obs}\cdot t}\right)$$ (2)

$$k_{obs}=k_{chem\;}\left[sulfonylpyrimidine\right]+k_{probe\;}\left[probe\right]$$ (3)

$$k_{chem}=\frac{k_{obs}}{\left[sulfonylpyrimidine\right]}\;$$ (4)

The observed *k*_chem_ values of a comprehensive set of sulfonylpyrimidines are shown in Table 5. Bauer et al. have already determined a *k*_chem_ value of compound **5c** vs. glutathione (*k*_chem_ = 82 M^−1^min-^1^). Our determined *k*_chem_ for the reaction of the identical compound with cysteine is 63 M^−1^min^−1^ and thus, as expected, in the same order of magnitude. The determined *k*_chem_ values ranged from 6 to 104 M^−1^min^−1^, i e., they are 10–10,000 times lower than for the reactions of the reported Michael acceptors 1-penten-3-one, methyl propiolate, and methyl acrylate with GSH (Böhme et al., 2009). This confirms the previous reports that sulfonylpyrimidines are mild cysteine modifiers with potential application in a cellular context (Bauer et al., 2016; Li L. et al., 2017; Förster et al., 2019; Jaudzems et al., 2020).

**TABLE 5:** Kinetic constants (*k*_chem_ and *k*_2nd_) of the selected compound set investigated by the cysteine reactivity assay. Selectivity indices were calculated from *k*_2nd_/*k*_chem_.

Cpd.	Structure	Cysteine *k*_chem_ (M^−1^min^−1^)	SrtA *k*_2nd_ (M^−1^min^−1^)	S. I.
5a	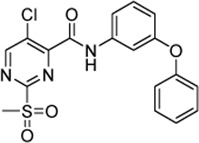	50.8 ± 16.6	8,088 ± 464	159
5c	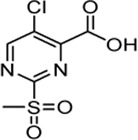	63.0 ± 2.01	0	—
6a	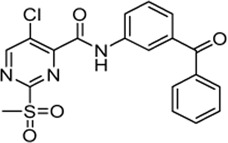	63.3 ± 8.37	20,785 ± 4,074	327
6i	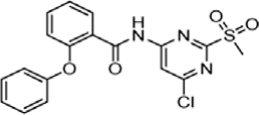	0	0	—
7a	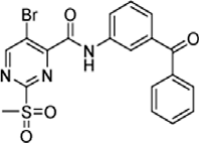	104.9 ± 22.5	28,185 ± 2,117	268
7b	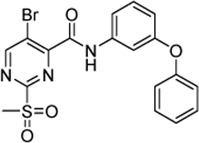	104.1 ± 19.4	13,998 ± 1,462	134
7c	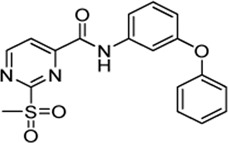	33.58 ± 2.93	4,364 ± 596	129
7d	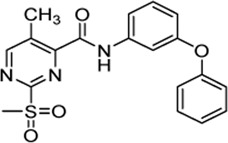	6.14 ± 1.71	1,204 ± 139	196
7e	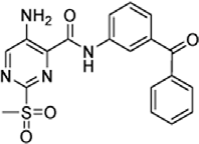	0	0	—

From the kinetic constants *k*_chem_ (cysteine assay) and *k*_2nd_ (SrtA inhibition), selectivity indices (S.I.) were calculated that compare how efficient the inactivation of SrtA was in comparison with the in-solution reactivity towards free cysteine (Table 5). It was found that the S.I. values do not depend on the halogen substituent, but rather on the recognition sequence: 3-benzoylaniline derivatives **6a** and **7a** were superior to 3-phenoxyaniline derivatives **5a** and **7b** in terms of the S.I. by about a factor of two. This is in agreement with findings from the enzymatic context that the recognition sequence mainly influences the affinity of binding (K_I_), with the halogen substituent influencing the reaction rate (*k*_inact_, *k*_chem_).

Furthermore, the influence of different solvents on the reaction of sulfonylpyrimidines with thiols in solution was investigated. No reactivity was observed between cysteine and **5a** or **5c** in water/DMSO mixtures. Likewise, no reactivity was detected between **5c** and 2-phenylethanethiol in chloroform. These observations are in agreement with the literature. In organic synthesis, such reactions required a base and elevated temperature (Sprague and Johnson, 1936; Buděšínský and Vavřina, 1972). However, between **5a** and cysteine, quantitative turnover (<10 min) was observed in phosphate-buffered solution during the preparation of ESI-MS experiments (Figure 5A) and cysteine reactivity assays (Figure 7). The buffer component appeared to be essential for the reaction in an aqueous solution.

In support of this qualitative statement, a quantitative estimation of the buffer-dependent reaction between sulfonylpyrimidines and cysteine was also performed: The cysteine reactivity assay of compound **5c** was performed in addition to the usage of phosphate buffer (*k*_chem_ = 63.0 ± 2.01 M^−1^min^−1^) also in bicarbonate buffered solution at an identical pH 7.5. The reaction rate in bicarbonate buffer appeared to be significantly reduced (*k*_chem_ = 21.9 ± 1.27 M^−1^min^−1^). Mechanistic participation of the buffer molecule is discussed during the quantum chemical investigations (Section 2.8).

### 2.7 Mechanistic Evaluation by Hammett Plot Analysis

**FIGURE 8:**
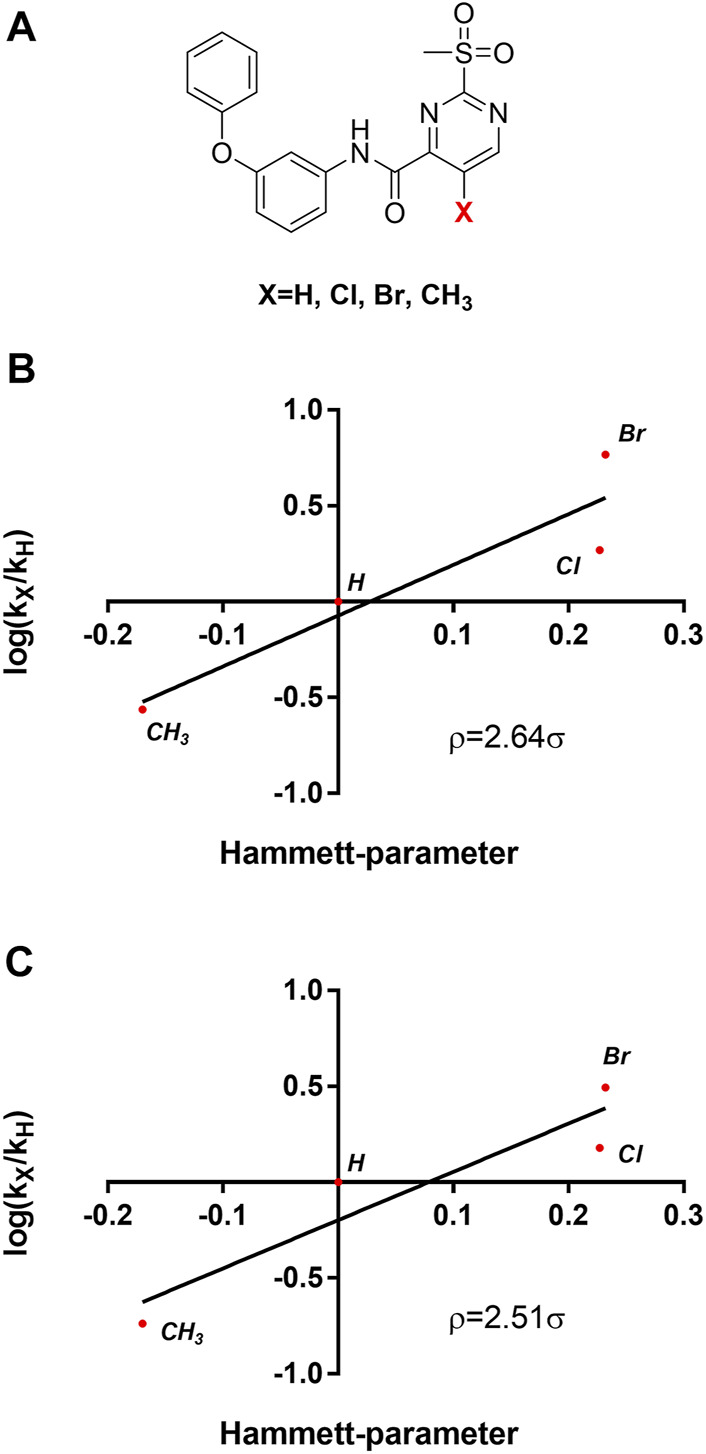
Mechanistic analysis by Hammett plots. **(A)** Substituent series based on compound **5a** used for kinetic characterization. **(B)** Hammett plot for the inactivation of SrtA by sulfonylpyrimidines. **(C)** Hammett plot for the reaction of sulfonylpyrimidines with cysteine in solution (phosphate buffer pH 7.5).

Table 3 shows that sulfonylpyrimidine derivatives, whose halogen substituent was varied, exhibited significantly different reaction rates in the inactivation of SrtA. The same was found for the cysteine reactivity assays (Table 5). If the pyrimidine ring is regarded as a pseudo-atom, according to Hammett’s theory, linear logarithmic relationships can be established between rate constants (M^−1^min^−1^) and Hammett’s substituent constants (σ), which allow qualitative mechanistic conclusions to be drawn (Hammett, 1937; Flynn, 1980). For this purpose, a substituent series based on compound **5a** (R = Cl, Br, H, CH_3_) was investigated (Figure 8A). Plotting the logarithmic-normalized second-order rate constants (*k*_2nd_, *k*_chem_) allowed us to generate Hammett plots for the reaction of sulfonylpyrimidines with SrtA (Figure 8B) and for the in-solution reaction with free cysteine (phosphate buffer pH 7.5, Figure 8C). From these, we found positive ρ values (slope) of 2.64 resp. 2.51σ, which are consistent with the rate-limiting nucleophilic attack (Kiemele et al., 2016), and strongly suggest a concerted *S*_*N*_Ar mechanism without stabilization of a Meisenheimer intermediate (Choi et al., 2009). The ρ-values obtained from enzymatic reactions and reactions with free cysteine are almost identical suggesting an analogous reaction mechanism for covalent modification of SrtA and free cysteine.

### 2.8 Theoretical Investigations

To gain insights into the mechanism of 2-sulfonylpyrimidine inhibitors in an enzymatic environment, QM/MM calculations would be desirable. However, the available X-ray structures of *S. aureus* SrtA differ strongly in the geometrical structure of their active site, and it is yet unclear which one represents the reactive conformation. Additionally, the enzyme is very flexible which makes it difficult to select appropriate conformations for the computation. Thus, unambiguous QM/MM computations were thought to be not available. Hence, we focused on the comparison of the warhead reaction in solution with the available experimental data. These findings were then applied to shed some light on the reaction of 2-sulfonylpyrimidines with SrtA. The reaction of inhibitor **5c** with methanethiol in (implicit) aqueous solution was selected as a model system. The relative orientation of the reactants together with the main reaction coordinate is depicted in Supplementary Figure S3. As the main reaction coordinate of the nucleophilic substitution of the sulfonyl group by the thiol (ate) we selected the distance between the sulfur center of thiol and the C-2 atom of the inhibitor (R (S_thiol_–C-2_inhibitor_)). To obtain single points of the corresponding reaction paths this distance was frozen while all other degrees of freedom were optimized. In the course of the calculations, only the R (S_thiol_–C-2_inhibitor_) distance was frozen but not the relative orientation of the two fragments to each other. Hence, the corresponding reaction path represents the minimum energy path (MEP) of the reaction. More details about the calculations are described in the experimental section.

First, the reactions of **5c** with methanethiol as well as methanethiolate were investigated. As shown in Supplementary Figure S4, the reaction with negatively charged methanethiolate occurs without any barrier, while the reaction with neutral methanethiol shows a barrier of 142 kJ/mol. Due to the high reaction barrier, a reaction of **5c** with a neutral thiol is not expected to take place. This is in line with the experimental findings that a reaction is not observed in organic solvents or neutral water/DMSO mixtures (Section 2.6) but only in a buffered medium. We also investigated if structures, which resemble the Meisenheimer complex, represent local minima on the potential surface. This is not the case as corresponding structures for the reaction with the thiol were found to be about 150 kJ/mol above the separated fragments. For the reaction with the thiolate, these were calculated to be more stable than the fragments but also do not represent local minima.

**FIGURE 9:**
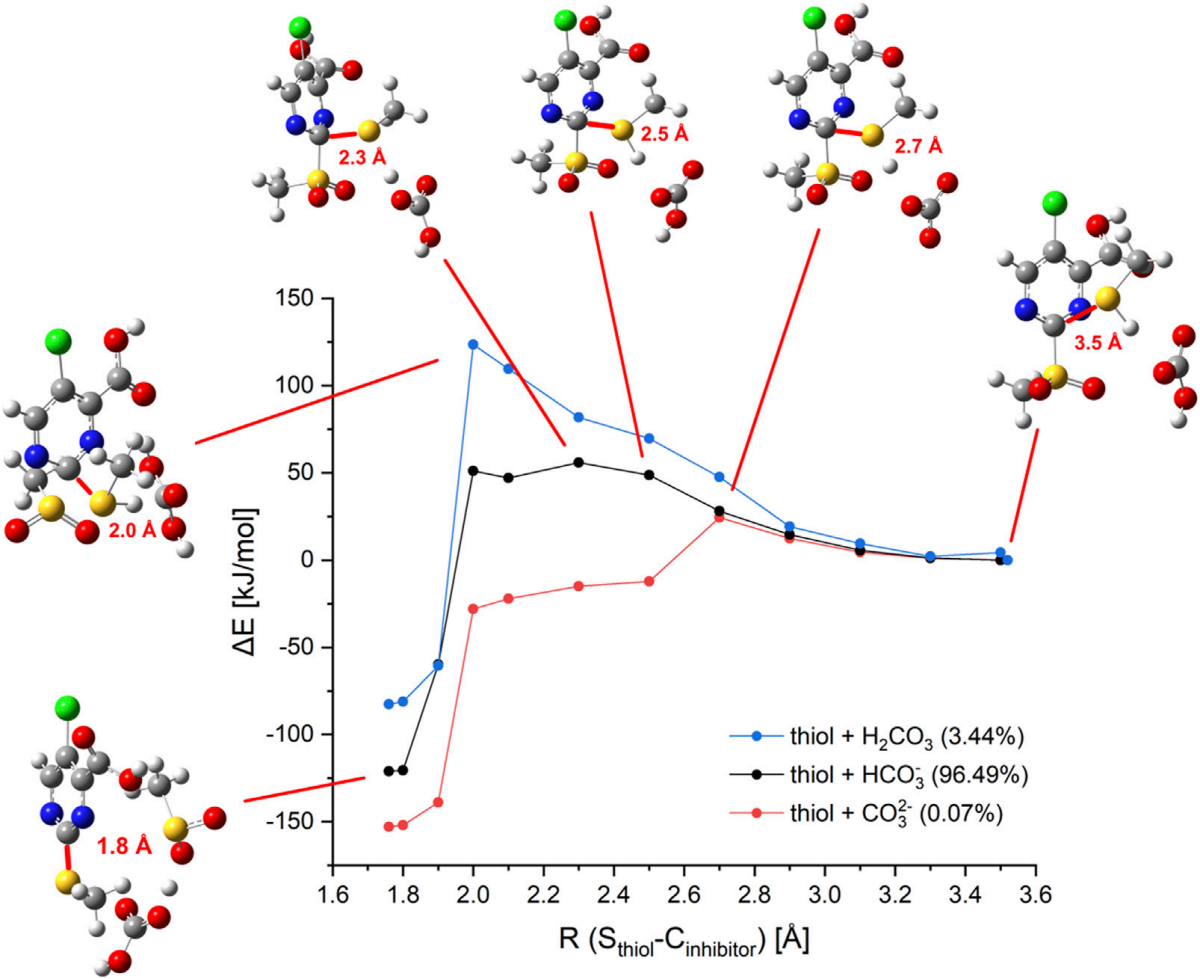
Computed reaction paths mediated by bicarbonate buffer. Energy profile of the calculated model reaction of inhibitor **5c** with methanethiol in presence of bicarbonate buffer species (and distribution of species at pH 7.5). Selected structures are shown in ball and stick representation with the reaction coordinate R (S_thiol_–C-2_inhibitor_) given in red.

To elucidate possible mechanisms of buffer-mediated reactions, we modeled reactions in which possible buffer molecules act as intermediate storage for the thiol proton before transferring it to the sulfonyl leaving group. At the investigated pH of 7.5, the majority of the bicarbonate buffer is composed of HCO_3_^¯^, but small fractions of CO_3_^2¯^ and H_2_CO_3_ are found as well. To include all these possibilities, we computed reaction paths for all protonation states of H_2_CO_3_. The results are depicted in Figure 9. Besides the reaction paths, Figure 9 depicts the variations in the geometrical arrangements for selected points along the reaction coordinate. Additional structures for the other paths can be found in Supplementary Table S1.

The reaction of methanethiol with HCO_3_^¯^ as buffer molecule shows an activation barrier of 56 kJ/mol which is significantly lower than the reaction barrier of the thiol without any buffer molecule (142 kJ/mol). The proton transfer to the buffer occurs at R (S_thiol_–C-2_inhibitor_) = 2.3 Å. This point represents the top of the barrier. For the thiol, the top of the barrier is at about R (S_thiol_–C-2_inhibitor_) = 2.0 Å. Neutral buffer molecules of H_2_CO_3_ are not able to bind the additional proton, hence no catalysis would be expected. Indeed, our computation predicted a reaction barrier of 124 kJ/mol, which lies only 20 kJ/mol below the barrier computed for unbuffered thiol (142 kJ/mol). CO_3_^2–^ as buffer molecule facilitates a fast reaction with a barrier height of merely 24 kJ/mol. For this species, the proton transfer occurs earlier in the reaction at R (S_thiol_–C-2_inhibitor_) = 2.5 Å. For the reaction catalyzed by HCO_3_^¯^ structures resembling the Meisenheimer complex are found to be about 50 kJ/mol higher in energy than the fragments, showing that no Meisenheimer complex is formed. These predictions that Meisenheimer complexes are energetically unfavorable supports the conclusion drawn by Hammett’s analysis (Section 2.7).

For further examination, the reaction paths for all phosphate buffer species were computed as well. The major component at pH 7.5 is HPO_4_^2–^, the computation yields an activation barrier of 31 kJ/mol, which is lower than that for HCO_3_^¯^ and comparable to the one found for CO_3_^2–^ (Supplementary Figure S6). This could be confirmed experimentally since a faster reaction was observed by using a phosphate buffer instead of a bicarbonate buffer (Section 2.6). The H_2_PO_4_^¯^ fraction of the phosphate buffer system shows a reaction barrier of 64 kJ/mol similar to the HCO_3_^¯^ mediated reaction.

**FIGURE 10:**
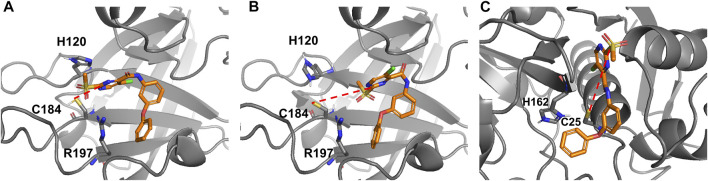
Docking poses of compound **5a** reversibly bound to SrtA and rhodesain. **(A)** Docking to a SrtA receptor with reversely protonated Cys/His dyad (pdb: 2kid). Distance (Cys^184^-SH/C-2) = 4.6 Å. **(B)** Docking to a SrtA receptor with an artificial zwitterionic Cys¯/His^+^ dyad (pdb: 2kid). Distance (Cys ^184^-S^-^/C-2) = 7.8 Å. **(C)** Docking to a rhodesain receptor (pdb: 2p7u). Distance (Cys^25^-S^-^/C-2) = 7.8 Å.

To determine whether the reaction can occur *via* the His^120^ residue in SrtA, the model reaction path was calculated with 4-methylimidazole as a conjugated base, yielding a barrier height of 64 kJ/mol. Consequently, the inhibition reaction in presence of histidine could lead to a reaction path analogous to the reaction mediated by bicarbonate or phosphate buffers. Our computations indicate that thiolates would react with 2-sulfonylpyrimidines without any barrier while the reaction with thiol requires a base as a catalyst. This stands in contrast to the experimental finding that 2-sulfonylpyrimidines inhibit a reversely protonated Cys/His dyad in enzymes like SrtA (as shown in Table 4), but not enzymes with zwitterionic Cys^¯^/His^+^ dyad as cathepsin-like proteases (Section 2.4). According to Zhang et al. and our model calculations, the nucleophilic reaction with a deprotonated cysteine residue should be preferred over the reaction with a neutral cysteine (Zhang et al., 2012). To address this question, we docked inhibitor **5a** to the SrtA receptors mimicking the zwitterionic and the neutral Cys/His dyads. The most advantageous docking poses are depicted in Figures 10A,B. For the neutral dyad, **5a** forms a quite stable complex blocking the active site. For the zwitterionic receptor, the inhibitor stays outside of the binding pocket with a distance (Cys^184^-SH/C-2) = 7.8 Å. A similar result is found for the docking pose of inhibitor **5a** in the zwitterionic cathepsin-like protease rhodesain (Figure 10C) which also shows an enlarged Cys^25^-S^¯^/C-2 distance of 7.8 Å.

Thus, the docking indicates that the possibility of a reaction does not result from the reactivity of the thiol or thiolate group but rather from the possibility to form an enzyme-inhibitor complex which can result in a covalent reaction. According to our findings for a zwitterionic dyad, the reaction would be very fast. However, it does not take place because the 2-sulfonylpyrimidine warhead does not fit into the active site due to unfavorable intermolecular interactions. For a neutral Cys/His dyad, the 2-sulfonylpyrimidine fits into the pocket, however, according to the experimental and theoretical findings, a base catalyst is needed that acts as a proton buffer. Our computations show that the histidine could be this catalyst if it is sufficiently close. This role of His ^120^ as a proton buffer is supported by the fact that the inhibition potency decreases between pH 7.0 and 6.5 for which His^120^ undergoes a significant protonation change (Connolly et al., 2003).

## 3 Conclusion

In summary, our investigations show that 2-sulfonylpyrimidines act as irreversible inhibitors for *S. aureus* SrtA possessing a neutral Cys/His dyad. Considerably weaker inhibition is found for enzymes with zwitterionic Cys¯/His^+^ dyads as found in cathepsin-like proteases. Inactivation measurements at higher pH values show no increase in the inhibitory potency of 2-sulfonylpyrimidines which, in this regard, stands in contrast to other known SrtA inhibitors. Measurements in solution show that no reaction takes place in a DMSO/water mixture. Only in presence of a buffer like bicarbonate or phosphate buffered solution (pH ∼7.5), a reaction was observed.

While the enzyme inactivation measurements indicate that 2-sulfonylpyrimidines react preferentially with protonated thiols, the investigations of in-solution cysteine reactivity indicate that this reaction is hampered. To get a better understanding of these conflicting data, we performed quantum chemical model calculations in water/DMSO, bicarbonate, and phosphate buffers. Our computations predict that thiolates react with 2-sulfonylpyrimidines without any barrier. This is not the case for a protonated thiol for which a very high barrier of 142 kJ/mol is computed. The barrier is considerably lowered for bicarbonate or phosphate buffer solutions because the conjugated bases, e.g., HCO_3_^¯^, CO_3_^2¯^, act as base-catalysts. The barriers drop because these bases induce a proton transfer from the thiol, thus increasing the nucleophilicity of the attacking agent. As expected, the decrease of the barrier heights correlates with the basicity of the catalyst. Additional computations show that imidazole can also act as a catalyst. It reduces the reaction barrier from 142 kJ/mol to about 64 kJ/mol.

The computations predict that a deprotonated cysteine should react considerably more efficiently with 2-sulfonylpyrimidines than protonated cysteine moieties. In contrast, our enzyme inhibition measurements only show an efficient inhibition of SrtA with neutral Cys/His dyads while enzymes possessing a zwitterionic Cys¯/His^+^ dyad in their reactive center are less affected. This contradiction is resolved by docking studies, which predict that 2-sulfonylpyrimidines only enter the active sites of enzymes with neutral Cys/His dyads. If the dyad of SrtA is artificially switched to a Cys¯/His^+^ dyad, the 2-sulfonylpyrimidine does not fit the active site. For rhodesain, which possesses a zwitterionic Cys¯/His^+^ dyad, the same results are found. Intermolecular interactions prevent the formation of an enzyme-inhibitor complex whose geometrical arrangements allow the reaction. As a catalyst for the reaction of 2-sulfonylpyrimidines with SrtA, the His^120^ moiety can act as a base catalyst. In line with the Hammett plots, our computations indicate that Meisenheimer complexes are energetically unfavorable for the energy surface of the *S*_*N*_Ar substitution reaction.

## 4 Materials and Methods

### 4.1 Synthesis

Synthesis protocols of all compounds with their analytical data and the spectral appendix are provided in the supporting information.

### 4.2 Protein Expression and Purification

Expression of the *S. aureus* SrtA was performed as described previously (Schmohl et al., 2017a). The pET23b expression construct was transformed into *E. coli* strain BL21 Gold (DE3) cells (Agilent Technologies, Santa Clara, California) and grown in LB medium with 100 µM ampicillin at 37 °C to an OD_600_ of ∼0.7. Expression was then induced with 1 mM isopropyl-d-thiogalactoside (IPTG) for 16 h at 20 °C. Harvested cells were resuspended in lysis buffer (20 mM Tris HCl, pH 6.9, 300 mM NaCl, 0.1% Triton X-100, RNase, DNase, lysozyme) and lysed by multiple cycles of sonication (Sonoplus, Bandelin, Berlin, Germany). After centrifugation (45 min at 15 krpm), the cleared lysate was subjected to IMAC (HisTrap HP 5 ml column, GE Healthcare, Chicago, Illinois) to isolate crude SrtA. For further purification, SrtA containing fractions were subsequently subjected to a gel-filtration step (HiLoad 16/60 Superdex 200 column, GE Healthcare) and eluted in the storage buffer (20 mM Tris-HCl, pH 7.50, 150 mM NaCl, 5 mM CaCl_2_). For storage at –80°C, the pure SrtA was concentrated, aliquoted and shock frozen in liquid nitrogen. Throughout all steps, protein concentrations were determined *via* absorbance at 280 nm and sample purity was assessed *via* sodium dodecyl sulfate-polyacrylamide gel electrophoresis (SDS-PAGE).

### 4.3 Inhibition of Sortase A

Inhibition assays of *S. aureus* SrtA transpeptidation reactions were performed as described previously (Barthels et al., 2020). Briefly, the recombinantly expressed SrtA (final concentration: 1 µM) was incubated in assay buffer (50 mM Tris, 150 mM NaCl, 5 mM CaCl_2_, pH 7.50) with 25 µM of the FRET-pair substrate Abz-LPETG-Dap (Dnp)-OH (Genscript, Piscataway, New Jersey) and 0.5 mM tetraglycine (Sigma Aldrich, St. Louis, Missouri). Inhibitors were added from DMSO stocks. Reactions were initiated by addition of SrtA and monitored for 30 min at 30°C in an Infinite M200 Pro plate reader with λ_ex_ 320 nm/λ_em_ 430 nm (Tecan, Männedorf, Switzerland). Three technical replicates were carried out for each inhibitor in black flat-bottom 96-well plates (Greiner bio-one, Kremsmünster, Austria). The enzyme kinetics of irreversible SrtA inactivation were analyzed as described previously (Barthels et al., 2020).

### 4.4 Protease Inhibition Selectivity

Fluorometric assays of the ZIKV/DENV NS2B/NS3 protease were performed as described previously (Maus et al., 2021). The assay was carried out in triplicates at 25°C in assay buffer (50 mM Tris, pH 9.0, 20% glycerol (v/v), and 1 mM CHAPS). 100 µM Boc-Gly-Arg-Arg-AMC (Bachem, Bubendorf, Switzerland) was used as a substrate. Fluorometric assays for cathepsin B, cathepsin L, and rhodesain (Calbiochem, Merck Millipore, Burlington, Massachusetts) were performed as described previously (Klein et al., 2020a). Cbz-Phe-Arg-AMC (Bachem, Bubendorf, Switzerland) was used as substrate (100 μM for cathepsin B, 6.5 μM for cathepsin L, and 10 µM for rhodesain). Fluorometric assays for urokinase plasminogen activator were performed as described previously (Angelini et al., 2012). 240 µM Cbz-Gly-Arg-Arg-AMC (Bachem, Bubendorf, Switzerland) was used as a substrate.

### 4.5 Mass Spectrometry

#### 4.5.1 ESI-MS

To a solution of 100 µM cysteine in phosphate-buffered saline (1×, pH 7.5), compound **5a** or **7a** (100 μM, 5% DMSO) was added and incubated for 10 min at room temperature. The negative labeling control was performed by mock treatment with DMSO. Samples were analyzed by LC/MS using an Agilent 1100 series HPLC system with an Agilent Poroshell 120 EC-C18 column (150 × 2.10 mm 4 μm; mobile phase: ACN/H2O 45:55 + 0.1% formic acid; flow rate: 0.4 ml/min) and electron spray ionization with the Agilent 1100 series LC/MSD Trap in positive ionization mode.

#### 4.5.2 MALDI-MS

An *S. aureus* SrtA stock solution was diluted in 100 μL assay buffer (50 mM Tris, 150 mM NaCl, 5 mM CaCl2, pH 7.50) to a final concentration of 10 µM. Compound **7a** was dissolved in DMSO and added to SrtA at a final concentration of 100 μM. The protein samples were allowed to react for 1 h at room temperature and subsequently, these were desalted using Zeba^TM^ Spin desalting columns (7 kDa MWCO, 0.5 ml; Thermo Scientific) according to the manufacturer’s instructions. For MALDI sample preparation, the desalted solution was mixed 1:1 with a matrix combination of equal amounts α-cyano-4-hydroxycinnamic acid (20 mg/ml dissolved in 70% acetonitrile and 30% of formic acid in water at a concentration of 5%) and 2,5-dihydroxybenzoic acid (20 mg/ml dissolved in 70% acetonitrile and 30% of trifluoroacetic acid in water at a concentration of 0.1%). The resulting mixture was allowed to dry slowly on the MALDI target prior to introduction into the mass spectrometer. The measurements were carried out on a rapiflex^TM^ MALDI-TOF/TOF mass spectrometer (Bruker Daltonik GmbH, Bremen, Germany). The instrument is equipped with a scanning smartbeam 10 kHz Nd:YAG laser at a wavelength of 355 nm and a 10 bit 5 GHz digitizer. The acceleration voltage was set to 20 kV and the mass spectra were recorded in positive ion linear mode. Calibration was done with the Bruker protein calibration standard II in a mass range from 10 to 70 kDa. Samples were measured at a laser power of 60% with random walk ionization across the sample spot.

### 4.6 Cysteine Reactivity Assay

Kinetic characterization of the reaction between sulfonylpyrimidines and cysteine was performed in degassed phosphate buffer (1×PBS, pH 7.5) or individual cases in bicarbonate buffer (20 mM NaHCO_3_, 100 mM NaCl, pH 7.5). Three technical replicates were carried out for each sulfonylpyrimidine in black flat-bottom 96-well plates. For this purpose, 50 µL buffered solution (including 5% (v/v) DMSO) of the respective sulfonylpyrimidine derivative (final: 250–3 µM) and the cysteine reactive probe **8** (final: 5 µM) were premixed. Reactions were initiated by the addition of 50 µL cysteine (final: 5 µM), dissolved in the respective buffer, and monitored for 30 min at 30°C in an Infinite M200 Pro plate reader with λ_ex_ 380 nm/λ_em_ 460 nm. To calculate *k*_obs_ and *k*_chem_ values, fluorescence curves were fitted using the same non-linear regression as for *in vitro* inhibition of SrtA (Barthels et al., 2020).

### 4.7 Molecular Modeling

JChem for Office (Excel) was used for the virtual synthesis of an in-house docking library. JChem for Office 20.18.0, 2020, ChemAxon (http://www.chemaxon.com) (Bode, 2004). For this purpose, a database of 438 in-house available amine building blocks was amide-coupled *in silico* with the sulfonylpyrimidine carboxylic acid **5c**. The resulting product structures were exported in the SDF file format. For virtual screening of this compound library, a Glide docking protocol was conducted within the Schrödinger Maestro 2020.04 worksuite (Friesner et al., 2004). The protein structures pdb: 2kid (frame 1, SrtA) and 2p7u (rhodesain) were downloaded from the Protein Databank (PDB). Before docking, the alkylated active-site Cys^184^ (SrtA) or Cys^25^ (rhodesain) were untethered and reprotonated. Receptor preparation was performed using the automated binding site, protonation, and energy minimization routine within Maestro “Protein preparation” and “Receptor grid generation.” Ligands were energy minimized using the “LigPrep” routine. The docking protocol was performed under default parameters with extra precision (XP) settings. Subsequently, predicted binding modes were analyzed to select the most promising candidate compounds for wet-lab synthesis. Ranking of the virtual screening output was performed according to the total docking score and in a second step according to the predicted binding mode (i.e., positioning and distance of the warhead relative to the Cys^184^).

### 4.8 Quantum Chemical Methods

All calculations were carried out with the Gaussian16 program (Frisch et al., 2020). The DFT functional B3LYP (Vosko et al., 1980; Lee et al., 1988; Becke, 1993) with Grimme’s D3 correction (Grimme et al., 2010) and the Pople basis set 6-31+G (d,p) were employed. Implicit solvation in water was included with the SMD solvation method (Marenich et al., 2009). The geometry of the reactants was optimized to obtain the starting structures. Subsequently, a scan was performed in which the distance of the methanethiol (ate) to the attacking carbon atom of the inhibitor (C-2) was gradually shortened. Reaction profiles were calculated for the reaction of the inhibitor with methanethiolate, methanethiol, and with methanethiol in presence of a buffer molecule. As buffer molecules, all species of the bicarbonate buffer and phosphate buffer species, respectively, were considered. For the reaction of the thiolate anion with the inhibitor, the dihedral angle between the thiolate carbon, thiolate sulfur, C-2 and C-5 of the inhibitor was frozen, because otherwise the thiolate was not oriented towards the attacking carbon. However, for the distances R (S_thiolate_-C-2_inhibitor_) = 1.8/1.9 Å the restraint was removed to ensure the correct product geometry. Reactants and products were confirmed by calculations without restraints and frequency calculations.

## Data Availability

The original contributions presented in the study are included in the article/Supplementary Material, further inquiries can be directed to the corresponding author.
