# Supplementary material for: 2-Sulfonylpyrimidines as Privileged Warheads for the Development of S. aureus Sortase A Inhibitors
Source: Front Mol Biosci. 2022 Jan 3;8:804970. doi: 10.3389/fmolb.2021.804970 (PMC8763382; doi:10.3389/fmolb.2021.804970)
Supplement: Supplementary file 1 [file DataSheet1.PDF]

Supplementary Material

1 General synthetic procedures

All reagents and solvents were of reagent/analytical grade quality and purchased from *Sigma-Aldrich*, *BLDpharmatech*, or *FisherScientific*. Chemicals were used without further purification. Solvents were purified by distillation and desiccated by standard methods if necessary. ^{1}H and ^{13}C spectra were recorded on a *Bruker* Fourier 300 using DMSO-d_6 or $CDCl_3$ as solvent. Chemical shifts δ are given in parts per million (ppm) using residual proton peaks of the solvent as internal standard (^{1}H / ^{13}C: DMSO: 2.50 / 39.52 ppm, $CHCl_3$: 7.26 / 77.16 ppm). Mass spectra were obtained by LC-MS consisting of an 1100 series HPLC system from *Agilent* with an *Agilent* Poroshell 120 EC-C_{18} 150 x 2.10 mm, 4 μm column. The detection wavelength was 254 nm. The molecular mass was detected by an *Agilent* 1100 series LC/MSD Trap with electron spray ionization (ESI) in positive mode. Column chromatography was performed with silica gel (0.06–0.02 mm) obtained from *Carl Roth*. All reactions were monitored by thin-layer chromatography using *Macherey-Nagel* ALUGRAM Xtra SIL G/UV254 silica gel 60 plates for detection at 254 nm. Melting points (uncorrected) were determined in open capillaries using a Schorpp MPM-H3 melting point device.

2 Synthesis of precursor compounds (1a–o, 2c–d)

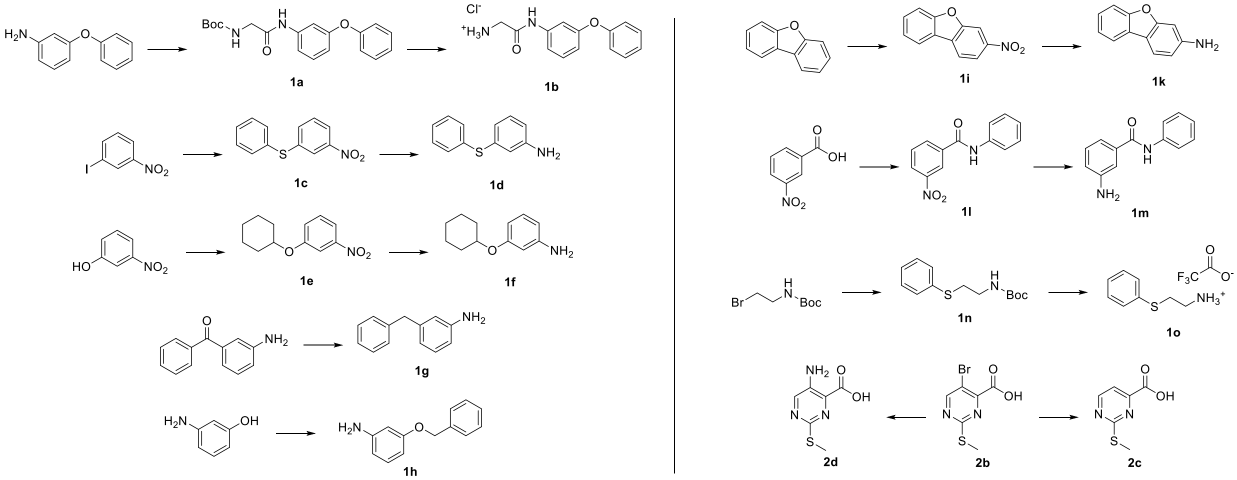

***Tert*-butyl *N*-{[(3-phenoxyphenyl)carbamoyl]methyl}carbamate (1a)**

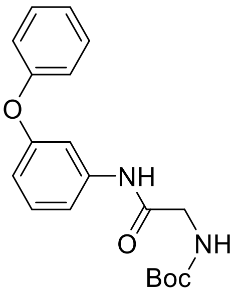

A solution of 3-phenoxyaniline (370 mg, 2.00 mmol), Boc-glycine (350 mg, 2.00 mmol), TBTU (642 mg, 2.00 mmol), and DIPEA (1047 µL, 6.00 mmol) in ethyl acetate (20 mL) was stirred for 16 h at room temperature. The organic phase was extract with $NaHCO_3$ sat., HCl (1 M) and filtered through a pad of silica. Evaporation of the solvent yielded the title compound **1a** as an orange solid (509 mg, 74%); mp: 122 °C; ^{1}H NMR (300 MHz, DMSO-d_6) δ= 9.99 (s, 1H), 7.46 – 7.35 (m, 2H), 7.35 – 7.24 (m, 3H), 7.20 – 7.10 (m, 1H), 7.07 – 6.95 (m, 3H), 6.75 – 6.64 (m, 1H), 3.68 (d, J = 6.1 Hz, 2H), 1.37 (s, 9H); ^{13}C NMR (75 MHz, DMSO-d_6) δ= 168.8, 157.5, 156.8, 156.3, 140.9, 130.5, 130.5 (2C), 124.0, 119.3 (2C), 114.2, 113.6, 109.3, 78.5, 44.2, 28.6 (3C); FT-IR (neat): ṽ [cm^{-1}]= 1671, 1589, 1533, 1485, 1440, 1366, 1285, 1220, 1159, 1057, 954, 896, 861, 757, 687.

[(3-Phenoxyphenyl)carbamoyl]methanamine hydrochloride (1b)

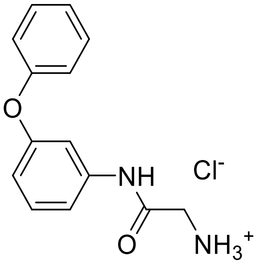

Boc-protected amide **1a** (171 mg, 0.50 mmol) was dissolved in tetrahydrofuran (5 mL). Concentrated hydrochloric acid (5 mL) was added dropwise and the mixture was stirred for 1 h at room temperature. The solvent was evaporated, and the residual was lyophilized from ACN/H_2O (1:1, 5 mL) to give the title compound **1b** as a colourless solid (112 mg, 80%). mp: 235 °C; ^{1}H NMR (300 MHz, DMSO-d_6) δ= 10.83 (s, 1H), 8.55 – 8.01 (m, 3H), 7.47 – 7.27 (m, 5H), 7.16 (t, J = 7.4 Hz, 1H), 7.03 (d, J = 8.0 Hz, 2H), 6.79 – 6.70 (m, 1H), 3.75 (q, J = 5.5 Hz, 2H); ^{13}C NMR (75 MHz, DMSO-d_6) δ= 165.3, 157.7, 156.6, 140.2, 130.7, 130.5 (2C), 124.2, 119.5 (2C), 114.2, 114.0, 109.2, 41.4; FT-IR (neat): ṽ [cm^{-1}]= 3040, 2832, 1679, 1594, 1557, 1478, 1445, 1286, 1246, 1211, 1165, 1130, 967, 938, 895, 882, 818, 790, 768, 743, 688,.

(3-Nitrophenyl)(phenyl)sulfane (1c)

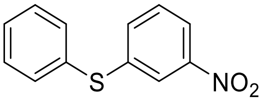

A suspension of 1-iodo-3-nitrobenzene (461 mg, 1.85 mmol), K_2CO_3 (415 mg, 3.00 mmol), thiophenol (234 µL, 2.30 mmol) and copper(I)iodide (145 mg, 0.76 mmol) in dimethylsulfoxide (5 mL) was thoroughly degassed with argon and transferred to an oxygen-free reflux apparatus. Under argon atmosphere, the reaction mixture was heated for 16 h to 140 °C. Subsequently, the solvent was distilled off and the residual was resolved in ethyl acetate. The organic phase was extract with $NaHCO_3$ sat. and HCl (1 M). Purification by column chromatography (CH/EA 20:1) afforded the title compound **1c** as an orange oil (178 mg, 41%); ^{1}H NMR (300 MHz, $CDCl_3$) δ= 8.06 – 7.95 (m, 2H), 7.54 – 7.36 (m, 7H); ^{13}C NMR (75 MHz, $CDCl_3$) δ= 148.8, 140.7, 134.3, 133.5 (2C), 132.2, 130.0 (2C), 129.8, 129.0, 123.2, 121.0; FT-IR (neat): ṽ [cm^{-1}]= 1522, 1475, 1463, 1439, 1344, 1125, 1066, 875, 799, 749, 728, 689, 667.

3-(Phenylthio)aniline (1d)

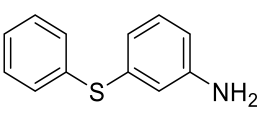

To a solution of nitroarene **1c** (173 mg, 0.75 mmol) in ethyl acetate/methanol (10+1 mL), tin(II)chloride (903 mg, 4.00 mmol) and HCl (1 mL, 1 M) were added. The reaction mixture was stirred for 2 h at 60 °C and subsequently quenched by the addition of $NaHCO_3$ sat. (5 mL). Extraction with ethyl acetate and filtration through a pad of silica afforded the title compound **1d** as a yellowish oil (108 mg, 71%); ^{1}H NMR (300 MHz, $CDCl_3$) δ= 7.28 – 7.19 (m, 2H), 7.18 – 7.07 (m, 3H), 6.93 (t, *J* = 7.9 Hz, 1H), 6.65 – 6.56 (m, 1H), 6.50 (t, *J* = 2.0 Hz, 1H), 6.38 (dd, *J* = 7.9, 2.3 Hz, 1H), 3.44 (s, 2H); ^{13}C NMR (75 MHz, $CDCl_3$) δ= 147.2, 136.3, 135.7, 131.0 (2C), 129.9, 129.1 (2C), 126.9, 120.9, 117.1, 113.9; FT-IR (neat): ṽ [cm^{-1}]= 3365, 1616, 1589, 1525, 1476, 1437, 1299, 1264, 1164, 1075, 1023, 991, 887, 857, 773, 739, 685.

1-(Cyclohexyloxy)-3-nitrobenzene (1e)

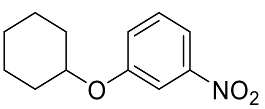

To an ice-cooled solution of 3-nitrophenol (278 mg, 2.00 mmol), cyclohexanol (832 µL, 8.00 mmol) and triphenylphosphine (1049 mg, 4.00 mmol) in tetrahydrofuran (10 mL) diisopropyl azodicarboxylate (785 µL, 4.00 mmol) was added. The reaction mixture was stirred under argon atmosphere for 168 h at room temperature. Purification by column chromatography (CH/EA 10:1) afforded the title compound **1e** as a yellowish oil (224 mg, 50%); ^{1}H NMR (300 MHz, $CDCl_3$) δ= 7.82 – 7.73 (m, 1H), 7.71 (t, *J* = 2.3 Hz, 1H), 7.39 (t, *J* = 8.2 Hz, 1H), 7.23 – 7.14 (m, 1H), 4.47 – 4.21 (m, 1H), 2.06 – 1.92 (m, 2H), 1.87 – 1.73 (m, 2H), 1.67 – 1.49 (m, 3H), 1.46 – 1.32 (m, 3H); ^{13}C NMR (75 MHz, $CDCl_3$) δ= 158.5, 149.3, 130.0, 122.9, 115.5, 110.2, 76.2, 74.4, 31.5, 25.5, 23.6, 21.7; FT-IR (neat): ṽ [cm^{-1}]= 2935, 2859, 1773, 1616, 1578, 1525, 1478, 1449, 1346, 1284, 1237, 1094, 1043, 1021, 999, 975, 892, 845, 811, 794, 736, 673.

3-(Cyclohexyloxy)aniline (1f)

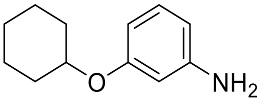

To a solution of nitroarene **1e** (177 mg, 0.80 mmol) in tetrahydrofuran (10 mL), Pd/C (20 mg) was added. The reaction mixture was stirred for 16 h under H_2-atmosphere (15 psi) at room temperature. Subsequently, the catalyst was filtered off and the solvent was removed *in vacuo* to afford the title compound **1f** as an orange oil (150 mg, 98%); ^{1}H NMR (300 MHz, $CDCl_3$) δ= 6.97 – 6.85 (m, 1H), 6.26 – 6.19 (m, 1H), 6.18 – 6.09 (m, 2H), 4.14 – 4.01 (m, 1H), 3.59 (s, 2H), 1.95 – 1.81 (m, 2H), 1.76 – 1.60 (m, 2H), 1.52 – 1.11 (m, 6H); ^{13}C NMR (75 MHz, $CDCl_3$) δ= 158.4, 147.3, 129.5, 107.3, 105.6, 102.8, 74.7, 31.4, 29.8, 25.1, 23.3, 21.4; FT-IR (neat): ṽ [cm^{-1}]= 2932, 2855, 1721, 1596, 1492, 1448, 1372, 1288, 1231, 1181, 1148, 1107, 1046, 1021, 984, 928, 888, 843, 762, 687.

3-Benzylaniline (1g)

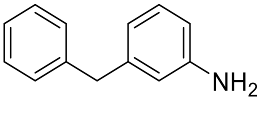

To a solution of 3-benzoylaniline (296 mg, 1.50 mmol) in tetrahydrofuran (10 mL), trifluoroacetic acid (1 mL) and Pd/C (30 mg) were added. The reaction mixture was stirred for 5 h under H_2-atmosphere (80 psi) at room temperature.

Subsequently, the catalyst was filtered off and the solvent was removed *in vacuo*. The residual was suspended in $NaHCO_3$ sat. and extracted twice with ethyl acetate. The organic phase was filtered through a pad of silica and evaporated to afford the title compound **1g** as a brownish oil (270 mg, 98%); ^{1}H NMR (300 MHz, $CDCl_3$) δ= 7.44 – 7.17 (m, 7H), 6.64 (d, *J* = 7.6 Hz, 1H), 6.59 – 6.51 (m, 1H), 3.92 (s, 2H), 3.47 (s, 2H); ^{13}C NMR (75 MHz, $CDCl_3$) δ= 146.4, 142.4, 141.2, 129.4, 129.0 (2C), 128.5 (2C), 126.1, 119.5, 115.9, 113.1, 42.0; FT-IR (neat): ṽ [cm^{-1}]= 3025, 2916, 1673, 1599, 1492, 1451, 1288, 1200, 1135, 1073, 1029, 995, 764, 724, 694.

3-(Benzyloxy)aniline (1h)

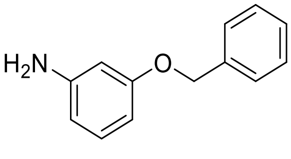

The title compound **1h** was synthesized by a procedure according to Tröster et al. 3-aminophenol (546 mg, 5.00 mmol) and KO*t*Bu (673 mg, 6.00 mmol) were solved in dimethylformamide (5 mL). Benzyl bromide (593 µL, 5.00 mmol) was added dropwise and the reaction mixture was stirred for 16 h at room temperature. Subsequently, the mixture was diluted with water (50 mL) and extracted thrice with ethyl acetate. The combined organic phases were washed with $NaHCO_3$ sat. and brine. Filtration through a pad of silica and evaporation of the solvent yielded the title compound **1h** as a beige solid (713 mg, 71%); mp: 62 °C; ^{1}H NMR (300 MHz, DMSO-*d*$_6$) δ= 7.55 – 7.28 (m, 5H), 7.13 – 6.97 (m, 1H), 6.46 – 6.37 (m, 1H), 6.36 – 6.27 (m, 2H), 5.03 (s, 2H), 3.65 (s, 2H); ^{13}C NMR (75 MHz, DMSO-*d*$_6$) δ= 160.1, 147.9, 137.3, 130.2, 128.6, 127.9, 127.5, 108.3, 105.0, 102.1, 69.9; FT-IR (neat): ṽ [cm^{-1}]= 1492, 1453, 1371, 1336, 1294, 1218, 1186, 1176, 1162, 1012, 989, 937, 911, 842, 758, 739, 689.

3-Nitrodibenzo[*b,d*]furan (1i)

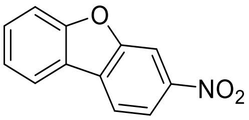

The title compound **1i** was synthesized by a procedure according to Li et al. Dibenzo[*b,d*]furan (505 mg, 3.00 mmol) was suspended in trifluoroacetic acid (1.2 mL) and cooled to –20 °C. Red fuming nitric acid (145 µL, 1.17 mmol) was carefully mixed with trifluoroacetic acid (1.0 mL) and the nitrating agent was added dropwise to the reaction. Subsequently, the reaction mixture was warmed to room temperature and stirred for 3 h until completion of the conversion. The precipitated product was isolated by filtration, washed with water and dried by lyophilization. The title compound **1i** was yielded as a beige solid (588 mg, 91%); mp: 179 °C; ^{1}H NMR (300 MHz, $CDCl_3$) δ= 8.44 (d, *J* = 2.0 Hz, 1H), 8.28 (dd, *J* = 8.5, 2.0 Hz, 1H), 8.09 – 7.99 (m, 2H), 7.70 – 7.53 (m, 2H), 7.50 – 7.39 (m, 1H); ^{13}C NMR (75 MHz, $CDCl_3$) δ= 158.4, 155.1, 130.3, 129.7, 126.6, 123.9, 121.9, 120.6, 118.6, 112.4, 111.7, 108.1; FT-IR (neat): ṽ [cm^{-1}]= 3102, 1629, 1522, 1457, 1421, 1359, 1340, 1263, 1231, 1197, 1107, 1061, 1018, 929, 871, 846, 820, 758, 733, 719, 688.

Dibenzo[*b,d*]furan-3-amine (1k)

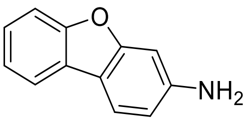

To a solution of nitroarene **1i** (320 mg, 1.50 mmol) in ethyl acetate/dichloromethane (10+10 mL), Pd/C (30 mg) was added. The reaction mixture was stirred for 6 h under H_2-atmosphere (60 psi) at room temperature. Subsequently, the catalyst was filtered off and the solvent was removed *in vacuo*

to afford the title compound **1k** as a yellowish solid (259 mg, 94%); mp: 89 °C; ^{1}H NMR (300 MHz, $CDCl_3$) δ= 7.85 – 7.77 (m, 1H), 7.71 (d, *J* = 8.2 Hz, 1H), 7.54 – 7.43 (m, 1H), 7.41 – 7.23 (m, 2H), 6.87 (d, *J* = 2.0 Hz, 1H), 6.71 (dd, *J* = 8.2, 2.0 Hz, 1H), 3.83 (s, 2H); ^{13}C NMR (75 MHz, $CDCl_3$) δ= 158.0, 156.0, 146.8, 125.2, 124.9, 122.6, 121.3, 119.4, 115.7, 111.3, 111.2, 97.6; FT-IR (neat): ṽ [cm^{-1}]= 3364, 1635, 1602, 1507, 1456, 1354, 1303, 1273, 1186, 1149, 1127, 1014, 940, 844, 810, 777, 747, 720.

3-Nitro-*N*-phenylbenzamide (1l)

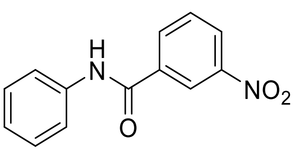

A solution of aniline (159 µL, 1.75 mmol), 3-nitrobenzoic acid (292 mg, 1.75 mmol), TBTU (562 mg, 1.75 mmol) and DIPEA (916 µL, 5.25 mmol) in ethyl acetate (20 mL) was stirred for 16 h at room temperature. The organic phase was extract with $NaHCO_3$ sat., HCl (1 M), and filtered through a pad of silica. Evaporation of the solvent yielded the title compound **1l** as an orange solid (344 mg, 81%); mp: 149 °C; ^{1}H NMR (300 MHz, $CDCl_3$) δ= 8.69 (t, *J* = 2.0 Hz, 1H), 8.43 – 8.32 (m, 1H), 8.31 – 8.20 (m, 1H), 8.19 – 8.08 (m, 1H), 7.73 – 7.58 (m, 3H), 7.44 – 7.30 (m, 2H), 7.23 – 7.11 (m, 1H); ^{13}C NMR (75 MHz, $CDCl_3$) δ= 163.5, 148.3, 137.4, 136.7, 133.5, 130.2, 129.3 (2C), 126.4, 125.4, 122.0, 120.7 (2C); FT-IR (neat): ṽ [cm^{-1}]= 3322, 1648, 1597, 1525, 1495, 1444, 1345, 1326, 1258, 1135, 1073, 1002, 914, 813, 752, 715, 689, 667.

3-Amino-*N*-phenylbenzamide (1m)

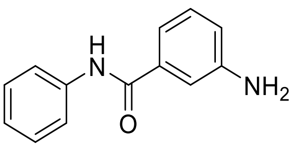

To a solution of nitroarene **1l** (291 mg, 1.20 mmol) in ethyl acetate (10 mL), Pd/C (30 mg) was added. The reaction mixture was stirred for 16 h under H_2-atmosphere (60 psi) at room temperature. Subsequently, the catalyst was filtered off and the solvent was removed *in vacuo* to afford the title compound **1m** as a beige solid (230 mg, 90%); mp: 124 °C; ^{1}H NMR (300 MHz, $CDCl_3$) δ= 7.95 (s, 1H), 7.68 – 7.57 (m, 2H), 7.39 – 7.30 (m, 2H), 7.23 – 7.08 (m, 4H), 6.84 – 6.75 (m, 1H), 3.82 (s, 2H); ^{13}C NMR (75 MHz, $CDCl_3$) δ= 166.2, 147.1, 138.1, 136.2, 129.7, 129.1 (2C), 124.5, 120.3 (2C), 118.3, 116.4, 113.9; FT-IR (neat): ṽ [cm^{-1}]= 3363, 1638, 1619, 1592, 1517, 1489, 1456, 1435, 1321, 1241, 1175, 1077, 994, 905, 880, 792, 750, 688.

Tert-butyl (2-(phenylthio)ethyl)carbamate (1n)

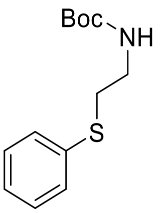

tert-butyl *N*-(2-bromoethyl)carbamate (448 mg, 2.00 mmol), thiophenol (204 µL, 2.00 mmol), DIPEA (1.0 mL, 6.00 mmol) and potassium iodide (332 mg, 2.00 mmol) were suspended in acetonitrile (5 mL) and stirred for 16 h at room temperature under argon atmosphere. Subsequently, the solvent was evaporated and the residual was resolved in ethyl acetate (10 mL). The organic phase was extract with $NaHCO_3$ sat., HCl (1 M), and filtered through a pad of silica. Evaporation of the solvent yielded the title compound **1n** as a colourless oil (414 mg, 81%); ^{1}H NMR (300 MHz, $CDCl_3$) δ= 7.40 – 7.32 (m, 2H), 7.31 – 7.23 (m, 2H), 7.21 – 7.11 (m, 1H), 3.31 (q, *J* = 6.5 Hz, 2H), 3.02 (t, *J* = 6.5 Hz, 2H), 1.43 (s, 9H); ^{13}C NMR (75 MHz, $CDCl_3$) δ= 155.3, 134.8, 129.2 (2C), 128.5 (2C), 125.9, 78.9,

39.2, 33.5, 27.9 (3C); FT-IR (neat): ṽ [cm^{-1}]= 3315, 2915, 1687, 1639, 1506, 1478, 1364, 1334, 1249, 1160, 1088, 1025, 947, 889, 736, 690.

2-(Phenylthio)ethan-1-amine trifluoroacetate (1o)

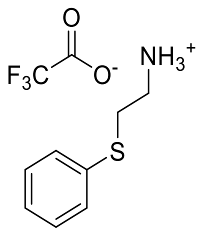

The Boc-protected amine **1n** (304 mg, 1.20 mmol) was suspended in dichloromethane (1.0 mL). Subsequently, trifluoroacetic acid (1.0 mL) was added and the reaction was stirred for 4 h at room temperature. Evaporation of the solvent and lyophilization from acetonitrile/water (1:1) yielded the title compound **1o** as a colourless oil (320 mg, 99%); ^{1}H NMR (300 MHz, DMSO-*d6*) δ= 8.02 (s, 3H), 7.46 – 7.30 (m, 4H), 7.29 – 7.18 (m, 1H), 3.25 – 3.12 (m, 2H), 3.03 – 2.90 (m, 2H); ^{13}C NMR (75 MHz, DMSO-*d6*) δ= 158.5 (d, *J* = 34.1 Hz), 134.2, 129.4 (2C), 128.8 (2C), 126.5, 38.2, 29.5; FT-IR (neat): ṽ [cm^{-1}]= 2903, 1669, 1475, 1439, 1328, 1134, 1024, 889, 840, 797, 740, 723, 690.

2-(Methylthio)pyrimidine-4-carboxylic acid (2c)

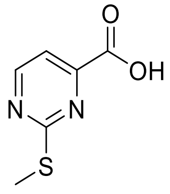

The title compound **2c** was synthesized by a procedure according to Arukwe et al. 5-bromo-2-(methylsulfanyl)pyrimidine-4-carboxylic acid **2a** (400 mg, 1.60 mmol) was solved in anhydrous tetrahydrofuran (15 mL) and cooled to –78 °C. A solution of *n*-butyllithium (1.3 mL, 2.5 M in hexanes) was added dropwise under argon atmosphere. The reaction mixture was stirred for 2 h at –78 °C and subsequently quenched by the addition of HCl (2 mL, 1 M). The mixture was warmed to room temperature and stirred for additional 30 min. The solvent was evaporated and the residual was extracted twice from dichloromethane/water. The combined organic layers were filtered through a pad of silica. Evaporation of the solvent yielded the title compound **2c** as a yellowish solid (57 mg, 20%); mp: 198 °C; ^{1}H NMR (300 MHz, DMSO-*d6*) δ= 8.86 (d, *J* = 4.9 Hz, 1H), 7.64 (d, *J* = 4.9 Hz, 1H), 2.55 (s, 3H); ^{13}C NMR (75 MHz, DMSO-*d6*) δ= 172.2, 164.9, 159.9, 156.1, 115.9, 13.6; FT-IR (neat): ṽ [cm^{-1}]= 1670, 1588, 1512, 1484, 1404, 1355, 1321, 1225, 1160, 1125, 1058, 966, 855, 766, 737, 693, 668.

5-Amino-2-(methylthio)pyrimidine-4-carboxylic acid (2d)

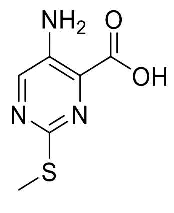

The title compound **2d** was synthesized by a procedure according to Grant et al. 5-bromo-2-(methylsulfanyl)pyrimidine-4-carboxylic acid **2a** (623 mg, 2.50 mmol) and copper(II)sulfate (24 mg, 0.15 mmol) were solved in NH_3 aq. (5 mL, 25%). The reaction mixture was heated in a sealed tube for 4 h to 100 °C. The cooled reaction mixture was adjusted to pH=1 with conc. HCl and the precipitate was isolated by filtration. The solid residual was washed with water and dichloromethane. After drying, the title compound **2d** was obtained as a yellow solid (195 mg, 42%); mp: 190 °C; ^{1}H NMR (300 MHz, DMSO-*d6*) δ= 8.44 (s, 1H), 2.47 (s, 3H); ^{13}C NMR (75 MHz, DMSO-*d6*) δ= 167.7, 154.8, 150.2, 140.6, 131.5, 13.6; FT-IR (neat): ṽ [cm^{-1}]= 3439, 3327, 2436, 1865, 1656, 1603, 1566, 1463, 1408, 1320, 1290, 1211, 1136, 985, 947, 905, 787, 736, 696, 663.

3 Synthesis of 2-(methylthio)pyrimidine-4-carboxamides (3a–z)

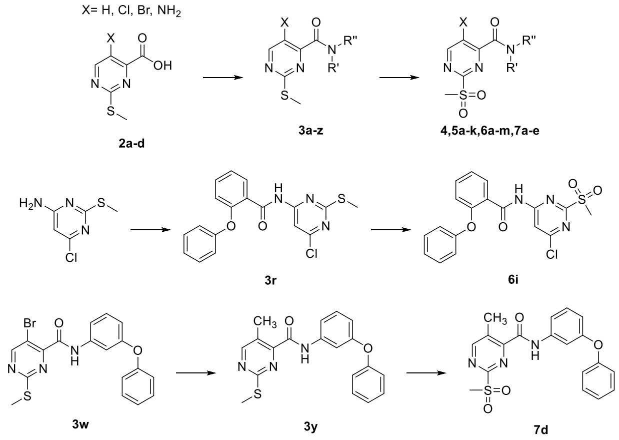

General procedure (I) for the synthesis of 2-(methylthio)pyrimidine-4-carboxamides

The respective pyrimidinecarboxylic acid (1.0 eq.), the corresponding amine component (1.0 eq.), TBTU (1.0 eq.) and DIPEA (3.0 eq.) were suspended in ethyl acetate and stirred for 24 h at room temperature. The organic phase was extracted with $NaHCO_3$ sat., HCl (1 M), and filtered through a pad of silica. Evaporation of the solvent yielded the corresponding 2-(methylthio)pyrimidine-4-carboxamides (**3a**–**z**), which were used without further purification.

N-(2-benzylphenyl)-5-chloro-2-(methylthio)pyrimidine-4-carboxamide (3a)

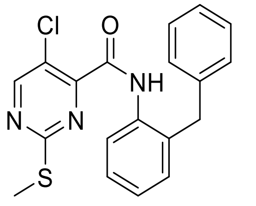

According to the general procedure (I), the title compound **3a** was obtained from **2a** and 2-benzylaniline as a yellowish solid (80 mg, 30%); mp: 150 °C; ^{1}H NMR (300 MHz, $CDCl_3$) δ= 9.45 (s, 1H), 8.67 (s, 1H), 8.20 (d, J = 7.7 Hz, 1H), 7.43 – 7.16 (m, 8H), 4.10 (s, 2H), 2.47 (s, 3H); ^{13}C NMR (75 MHz, $CDCl_3$) δ= 170.0, 160.8, 159.3, 151.5, 138.7, 135.1, 131.3, 131.0, 129.0 (2C), 128.6 (2C), 127.8, 126.8, 125.8, 125.3, 123.1, 38.2, 14.6; FT-IR (neat): ṽ [cm^{-1}]= 3345, 1702, 1585, 1547, 1529, 1511, 1473, 1453, 1434, 1397, 1315, 1299, 1214, 1154, 1072, 1046, 962, 754, 726, 694, 672, 655.

5-Chloro-2-(methylthio)-*N*-(3-phenoxyphenyl)pyrimidine-4-carboxamide (3b)

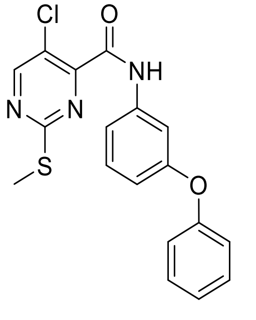

According to the general procedure (I), the title compound **3b** was obtained from **2a** and 3-phenoxyaniline as a yellow solid (170 mg, 91%); mp: 148 °C; ^{1}H NMR (300 MHz, DMSO-d_6) δ= 10.89 (s, 1H), 8.94 (s, 1H), 7.51 – 7.33 (m, 6H), 7.17 (t, J = 7.4 Hz, 1H), 7.07 (d, J = 8.0 Hz, 2H), 6.82 (dd, J = 8.0, 2.3 Hz, 1H), 2.57 (s, 3H); ^{13}C NMR (75 MHz, DMSO-d_6) δ= 169.9, 161.2, 158.5, 157.4, 157.2, 156.1, 139.4, 130.4, 130.1 (2C), 123.8, 122.1, 119.0 (2C), 114.4, 109.4, 14.0; FT-IR (neat): ṽ [cm^{-1}]= 3367, 1693, 1589, 1534, 1509, 1485, 1418, 1394, 1312, 1270, 1213, 1175, 1157, 1050, 964, 865, 809, 787, 773, 734, 687, 675.

5-Chloro-2-(methylthio)-*N*-(2-oxo-2-((3-phenoxyphenyl)amino)ethyl)pyrimidine-4-carboxamide (3c)

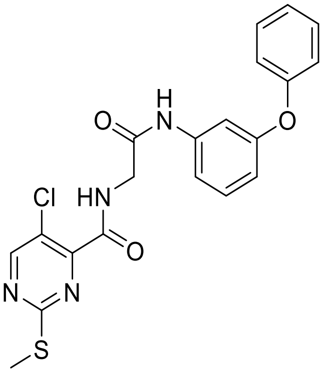

According to the general procedure (I), the title compound **3c** was obtained from **2a** and **1b** as a colourless solid (112 mg, 52%); mp: 193 °C; ^{1}H NMR (300 MHz, DMSO-d_6) δ= 10.19 (s, 1H), 9.08 (t, *J* = 5.8 Hz, 1H), 8.88 (s, 1H), 7.47 – 7.35 (m, 2H), 7.35 – 7.26 (m, 3H), 7.21 – 7.09 (m, 1H), 7.08 – 6.98 (m, 2H), 6.79 – 6.66 (m, 1H), 4.08 (d, *J* = 5.8 Hz, 2H), 2.57 (s, 3H); ^{13}C NMR (75 MHz, DMSO-d_6) δ= 169.4, 166.8, 162.6, 158.9, 157.1, 156.3, 156.0, 140.2, 130.2, 130.0 (2C), 123.6, 122.9, 118.9 (2C), 113.8, 113.3, 108.8, 42.7, 14.0; FT-IR (neat): ṽ [cm^{-1}]= 3321, 1700, 1668, 1609, 1594, 1536, 1506, 1480, 1419, 1388, 1312, 1257, 1216, 1171, 1149, 947, 886, 795, 756, 693, 663.

5-Chloro-*N*-(4'-cyano-[1,1'-biphenyl]-4-yl)-2-(methylthio)pyrimidine-4-carboxamide (3d)

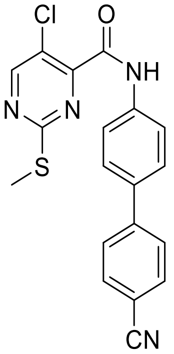

According to the general procedure (I), the title compound **3d** was obtained from **2a** and 4'-amino-[1,1'-biphenyl]-4-carbonitrile as a yellowish solid (115 mg, 60%); mp: 225 °C; ^{1}H NMR (300 MHz, DMSO-d_6) δ= 11.01 (s, 1H), 8.97 (s, 1H), 7.97 – 7.77 (m, 8H), 2.59 (s, 3H); ^{13}C NMR (75 MHz, DMSO-d_6) δ= 169.9, 161.2, 158.5, 157.4, 143.8, 138.5, 134.1, 132.8 (2C), 127.7 (2C), 127.1 (2C), 122.2, 120.2 (2C), 118.9, 109.7, 14.1; FT-IR (neat): ṽ [cm^{-1}]= 3339, 2222, 1678, 1603, 1590, 1550, 1532, 1501, 1419, 1399, 1311, 1217, 1183, 1154, 1050, 962, 809, 741, 719, 707, 679, 656.

***Tert*-butyl 5-(5-chloro-2-(methylthio)pyrimidine-4-carboxamido)-1*H*-indazole-1-carboxylate (3e)**

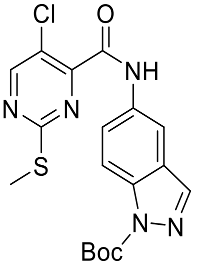

According to the general procedure (I), the title compound **3e** was obtained from **2a** and *tert*-butyl 5-amino-1*H*-indazole-1-carboxylate as an orange solid (200 mg, 95%); mp: 187 °C; ^{1}H NMR (300 MHz, $CDCl_3$) δ= 9.76 (s, 1H), 8.70 (s, 1H), 8.48 – 8.37 (m, 1H), 8.24 – 8.10 (m, 2H), 7.61 (dd, *J* = 9.0, 2.1 Hz, 1H), 2.65 (s, 3H), 1.73 (s, 9H); ^{13}C NMR (75 MHz, $CDCl_3$) δ= 169.6, 160.6, 158.9, 150.7, 148.7, 139.2, 136.8, 132.6, 126.0, 125.0, 121.8, 114.8, 111.3, 84.8, 27.8 (3C), 14.4; FT-IR (neat): ṽ [cm^{-1}]= 1734, 1686, 1602, 1546, 1517, 1431, 1385, 1349, 1305, 1289, 1245, 1214, 1159, 1146, 1030, 896, 873, 844, 829, 792, 764, 669.

5-Chloro-2-(methylthio)-*N*-(4-(*p*-tolyl)thiazol-2-yl)pyrimidine-4-carboxamide (3f)

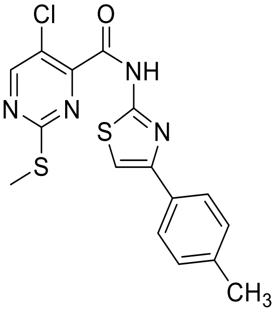

According to the general procedure (I), the title compound **3f** was obtained from **2a** and 4-(*p*-tolyl)thiazol-2-amine as a yellowish solid (80 mg, 42%); mp: 162 °C; ^{1}H NMR (300 MHz, DMSO-*d*$_6$) δ= 13.12 (s, 1H), 8.98 (s, 1H), 7.82 (d, *J* = 8.1 Hz, 2H), 7.71 – 7.61 (m, 2H), 7.00 (s, 1H), 2.60 (s, 3H), 2.33 (s, 3H); ^{13}C NMR (75 MHz, DMSO-*d*$_6$) δ= 168.8, 158.6, 155.8, 137.5, 131.2, 129.3, 129.2 (2C), 125.7, 125.5 (2C), 122.6, 108.5, 100.9, 20.8, 14.0; FT-IR (neat): ṽ [cm^{-1}]= 1688, 1627, 1537, 1518, 1434, 1380, 1334, 1321, 1306, 1268, 1206, 1182, 1150, 1131, 1063, 1040, 820, 794, 753, 730, 708, 676.

(5-Chloro-2-(methylthio)pyrimidin-4-yl)(3,4-dihydroisoquinolin-2(1*H*)-yl)methanone (3g)

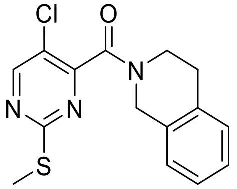

According to the general procedure (I), the title compound **3g** was obtained from **2a** and 1,2,3,4-tetrahydroisoquinoline as a beige solid (151 mg, 94%); mp: 141 °C; ^{1}H NMR (300 MHz, $CDCl_3$) δ= 8.58 (d, *J* = 3.7 Hz, 1H), 7.32 – 6.90 (m, 4H), 5.03 – 4.35 (m, 2H), 4.08 – 3.45 (m, 2H), 2.97 (dt, *J* = 26.9, 6.0 Hz, 2H), 2.56 (d, *J* = 7.0 Hz, 3H); ^{13}C NMR (75 MHz, $CDCl_3$) δ= 171.5, 163.4, 163.2, 159.4, 157.3, 134.4, 133.6, 132.0, 131.7, 129.1, 128.8, 127.3, 126.9, 126.8, 126.7, 126.0, 122.6, 47.9, 44.2, 44.0, 39.9, 30.0, 29.5, 28.3, 14.7; FT-IR (neat): ṽ [cm^{-1}]= 3342, 1696, 1593, 1569, 1522, 1474, 1434, 1386, 1333, 1306, 1261, 1247, 1207, 1148, 1117, 1043, 986, 943, 885, 862, 770, 737, 686, 665.

5-Chloro-2-(methylthio)-*N*-(pentan-3-yl)pyrimidine-4-carboxamide (3h)

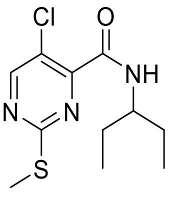

According to the general procedure (I), the title compound **3h** was obtained from **2a** and pentan-3-amine as a colourless solid (123 mg, 89%); mp: 125 °C; ^{1}H NMR (300 MHz, $CDCl_3$) δ= 8.62 (s, 1H), 7.30 (d, *J* = 9.2 Hz, 1H), 4.04 – 3.77 (m, 1H), 2.57 (s, 3H), 1.76 – 1.39 (m, 4H), 0.95 (t, *J* = 7.4 Hz, 6H); ^{13}C NMR (75 MHz, $CDCl_3$) δ= 169.8, 161.5, 160.4, 152.5, 125.0, 52.6, 27.4 (2C), 14.7, 10.4 (2C); FT-IR (neat): ṽ [cm^{-1}]= 2922, 1647, 1546, 1526, 1464, 1445, 1386, 1361, 1332, 1308, 1284, 1250, 1197, 1105, 1039, 929, 841, 814, 780, 753, 731, 657.

***N,N*-dibenzyl-5-chloro-2-(methylthio)pyrimidine-4-carboxamide (3i)**

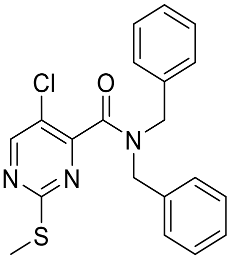

According to the general procedure (I), the title compound **3i** was obtained from **2a** and *N,N*-dibenzylamine as a beige solid (190 mg, 99%); mp: 120 °C; ^{1}H NMR (300 MHz, $CDCl_3$) δ= 8.98 (s, 1H), 7.91 – 7.58 (m, 10H), 5.14 (s, 2H), 4.67 (s, 2H), 2.97 (s, 3H); ^{13}C NMR (75 MHz, $CDCl_3$) δ= 171.4, 164.9, 159.4, 157.3, 135.8, 135.0, 129.1, 128.9 (2C), 128.6 (2C), 128.3 (2C), 127.9 (2C), 126.5, 122.6, 50.6, 46.6, 14.6; FT-IR (neat): ṽ [cm^{-1}]= 1645, 1529, 1494, 1473, 1452, 1439, 1417, 1386, 1366, 1355, 1334, 1304, 1263, 1201, 1166, 1025, 1009, 959, 784, 743, 729, 699, 665.

N-(3-benzoylphenyl)-5-chloro-2-(methylthio)pyrimidine-4-carboxamide (3k)

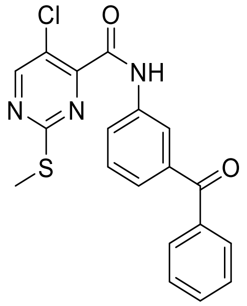

According to the general procedure (I), the title compound **3k** was obtained from **2a** and 3-benzoylaniline as a beige solid (237 mg, 88%); mp: 158 °C; ^{1}H NMR (300 MHz, $CDCl_3$) δ= 9.72 (s, 1H), 8.70 (s, 1H), 8.23 – 8.10 (m, 1H), 7.98 (t, J = 1.9 Hz, 1H), 7.89 – 7.76 (m, 2H), 7.67 – 7.42 (m, 5H), 2.64 (s, 3H); ^{13}C NMR (75 MHz, $CDCl_3$) δ= 196.1, 170.2, 161.1, 159.4, 151.0, 138.7, 137.3, 137.2, 132.8, 130.2 (2C), 129.3, 128.5 (2C), 126.8, 125.4, 123.9, 121.1, 14.8; FT-IR (neat): ṽ [cm^{-1}]= 3323, 1687, 1648, 1593, 1532, 1509, 1483, 1415, 1395, 1317, 1215, 1157, 1125, 1055, 968, 893, 856, 814, 787, 719, 707, 693, 669.

5-Chloro-N-(3-(cyclohexyloxy)phenyl)-2-(methylthio)pyrimidine-4-carboxamide (3l)

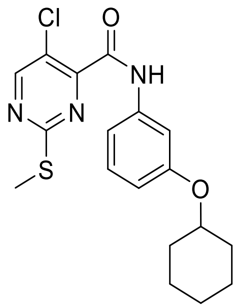

According to the general procedure (I), the title compound **3l** was obtained from **2a** and **1f** as a reddish oil (220 mg, 97%); ^{1}H NMR (300 MHz, $CDCl_3$) δ= 9.55 (s, 1H), 8.61 (s, 1H), 7.40 (t, J = 2.2 Hz, 1H), 7.24 – 7.08 (m, 2H), 6.78 – 6.62 (m, 1H), 4.34 – 4.19 (m, 1H), 2.57 (s, 3H), 2.04 – 1.85 (m, 2H), 1.84 – 1.67 (m, 2H), 1.57 – 1.14 (m, 6H); ^{13}C NMR (75 MHz, $CDCl_3$) δ= 169.8, 160.5, 159.1, 158.4, 151.6, 138.0, 129.7, 125.0, 112.8, 112.0, 107.9, 75.3, 38.5, 31.7, 25.6, 23.5, 21.9, 14.6; FT-IR (neat): ṽ [cm^{-1}]= 3338, 2937, 1691, 1593, 1536, 1513, 1442, 1393, 1310, 1273, 1213, 1153, 1046, 1000, 985, 941, 867, 767, 736, 684, 670.

N-(3-benzylphenyl)-5-chloro-2-(methylthio)pyrimidine-4-carboxamide (3m)

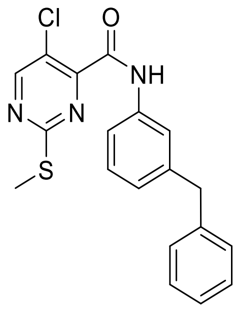

According to the general procedure (I), the title compound **3m** was obtained from **2a** and **1g** as a beige solid (138 mg, 53%); mp: 153 °C; ^{1}H NMR (300 MHz, $CDCl_3$) δ= 9.97 (s, 1H), 9.13 (s, 1H), 8.01 (d, J = 8.3 Hz, 2H), 7.77 – 7.58 (m, 7H), 7.46 (d, J = 7.3 Hz, 1H), 4.45 (s, 2H), 3.07 (s, 3H); ^{13}C NMR (75 MHz, $CDCl_3$) δ= 169.9, 161.0, 159.1, 151.4, 142.5, 140.8, 137.1, 129.3, 129.0 (2C), 128.6 (2C), 126.3, 125.9, 125.4, 120.5, 117.9, 42.0, 14.8; FT-IR (neat): ṽ [cm^{-1}]= 3350, 1688, 1609, 1535, 1514, 1489, 1452, 1420, 1393, 1307, 1214, 1153, 1050, 883, 810, 784, 759, 734, 699, 661.

5-Chloro-N-(3-methoxyphenyl)-2-(methylthio)pyrimidine-4-carboxamide (3n)

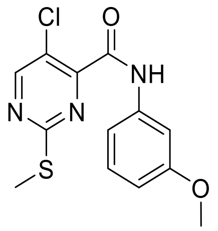

According to the general procedure (I), the title compound **3n** was obtained from **2a** and 3-methoxyaniline as a beige solid (185 mg, 85%); mp: 151 °C; ^{1}H NMR (300 MHz, $CDCl_3$) δ= 9.98 (s, 1H), 9.08 (s, 1H), 7.90 (t, J = 2.3 Hz, 1H), 7.71 – 7.61 (m, 1H), 7.57 – 7.50 (m, 1H), 7.13 (dd, J = 8.2, 2.5 Hz, 1H), 4.24 (s, 3H), 3.03 (s, 3H); ^{13}C NMR (75 MHz, $CDCl_3$) δ= 170.0, 161.0, 160.4, 159.1, 151.3, 138.2, 129.9, 125.3, 112.2, 111.3, 105.5, 55.5, 14.7; FT-IR (neat): ṽ [cm^{-1}]= 3318, 1691, 1595, 1540, 1515, 1469, 1454, 1430, 1385, 1336, 1287, 1206, 1180, 1158, 1084, 1036, 958, 855, 798, 770, 737, 688, 675.

N-([1,1'-biphenyl]-3-yl)-5-chloro-2-(methylthio)pyrimidine-4-carboxamide (3o)

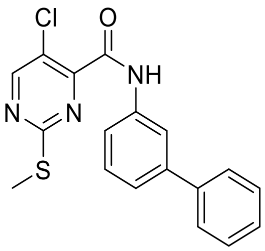

According to the general procedure (I), the title compound **3o** was obtained from **2a** and [1,1'-biphenyl]-3-amine as a beige solid (225 mg, 90%); mp: 189 °C; ^{1}H NMR (300 MHz, $CDCl_3$) δ= 9.67 (s, 1H), 8.70 (s, 1H), 8.00 (t, *J* = 1.9 Hz, 1H), 7.76 – 7.57 (m, 3H), 7.50 – 7.32 (m, 5H), 2.65 (s, 3H); ^{13}C NMR (75 MHz, $CDCl_3$) δ= 170.0, 161.0, 159.2, 151.3, 142.5, 140.6, 137.4, 129.6, 128.9 (2C), 127.7, 127.4 (2C), 125.4, 124.0, 118.8, 14.8; FT-IR (neat): ṽ [cm^{-1}]= 3367, 1691, 1601, 1537, 1504, 1449, 1406, 1392, 1315, 1214, 1153, 963, 868, 820, 802, 757, 738, 697, 673.

5-Chloro-2-(methylthio)-N-(2-phenoxyphenyl)pyrimidine-4-carboxamide (3p)

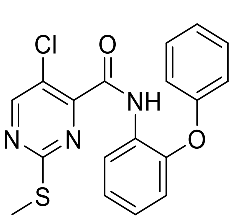

According to the general procedure (I), the title compound **3p** was obtained from **2a** and 2-phenoxyaniline as a yellowish solid (191 mg, 73%); mp: 126 °C; ^{1}H NMR (300 MHz, $CDCl_3$) δ= 10.50 (s, 1H), 8.70 – 8.61 (m, 2H), 7.44 – 7.30 (m, 2H), 7.23 – 7.01 (m, 5H), 6.93 (dd, *J* = 8.1, 1.5 Hz, 1H), 2.39 (s, 3H); ^{13}C NMR (75 MHz, $CDCl_3$) δ= 170.1, 161.1, 159.1, 156.6, 151.1, 146.1, 130.1 (2C), 129.3, 125.4, 125.0, 124.5, 124.1, 120.5, 118.4 (2C), 118.2, 14.6; FT-IR (neat): ṽ [cm^{-1}]= 3313, 1697, 1588, 1541, 1516, 1491, 1449, 1383, 1333, 1302, 1248, 1205, 1100, 1046, 876, 787, 745, 693, 675.

5-Chloro-2-(methylthio)-N-(4-phenoxyphenyl)pyrimidine-4-carboxamide (3q)

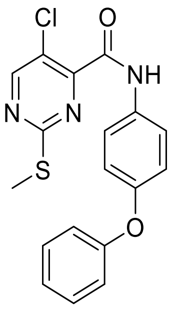

According to the general procedure (I), the title compound **3q** was obtained from **2a** and 4-phenoxyaniline as a yellowish solid (221 mg, 84%); mp: 181 °C; ^{1}H NMR (300 MHz, $CDCl_3$) δ= 9.56 (s, 1H), 8.69 (s, 1H), 7.75 – 7.62 (m, 2H), 7.40 – 7.29 (m, 2H), 7.17 – 6.96 (m, 5H), 2.64 (s, 3H); ^{13}C NMR (75 MHz, $CDCl_3$) δ= 170.0, 161.0, 159.0, 157.4, 154.3, 151.4, 132.4 (2C), 129.9, 125.4, 123.4, 121.7 (2C), 119.7 (2C), 118.7 (2C), 14.7; FT-IR (neat): ṽ [cm^{-1}]= 3352, 1684, 1595, 1508, 1488, 1394, 1316, 1249, 1212, 1155, 1105, 1047, 959, 849, 813, 798, 738, 687.

N-(6-chloro-2-(methylthio)pyrimidin-4-yl)-2-phenoxybenzamide (3r)

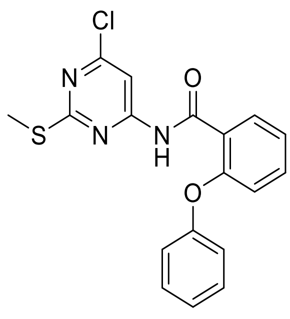

To a solution of 2-phenoxybenzoic acid (214 mg, 1.00 mmol) in toluene (1 mL), thionyl chloride (725 µL, 10.0 mmol) was added. The mixture was refluxed for 1 h and evaporated *in vacuo*. Subsequently, the isolated acyl chloride was added dropwise to a solution of 4-amino-6-chloro-2-(methylthio)pyrimidine (176 mg, 1.00 mmol) and DIPEA (524 µL, 3.00 mmol) in dichloromethane (5 mL). The reaction mixture was stirred for 168 h at room temperature, evaporated, and resolved in ethyl acetate. Extraction with $NaHCO_3$ sat. and purification by silica column

chromatography (CH/EA 10:1 + 1% TFA) afforded the title compound **3r** as a yellowish solid (22 mg, 5.9%); mp: 148 °C; ^{1}H NMR (300 MHz, DMSO-d_6) δ= 10.24 (s, 1H), 8.25 (dd, J = 7.9, 1.8 Hz, 1H), 8.07 (s, 1H), 7.52 – 7.37 (m, 3H), 7.31 – 7.20 (m, 2H), 7.16 – 7.08 (m, 2H), 6.95 – 6.88 (m, 1H), 2.46 (s, 3H); ^{13}C NMR (75 MHz, DMSO-d_6) δ= 172.8, 164.3, 162.5, 158.6, 156.2, 155.3, 134.9, 133.1, 130.9, 125.8, 124.5, 120.1, 118.9, 118.4, 105.8, 28.5; FT-IR (neat): ṽ [cm^{-1}]= 3252, 2903, 1657, 1544, 1500, 1458, 1415, 1359, 1307, 1207, 1167, 1112, 1020, 778, 755, 710, 665.

N-(3-(benzyloxy)phenyl)-5-chloro-2-(methylthio)pyrimidine-4-carboxamide (3s)

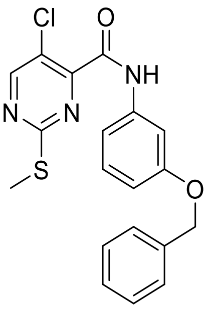

According to the general procedure (I), the title compound **3s** was obtained from **2a** and **1h** as a beige solid (188 mg, 97%); mp: 62 °C; ^{1}H NMR (300 MHz, $CDCl_3$) δ= 9.51 (s, 1H), 8.62 – 8.56 (m, 1H), 7.52 (t, J = 2.2 Hz, 1H), 7.40 – 7.04 (m, 7H), 6.72 (dd, J = 8.2, 2.5, Hz, 1H), 5.01 (s, 2H), 2.54 (s, 3H); ^{13}C NMR (75 MHz, $CDCl_3$) δ= 170.0, 161.0, 159.5, 159.1, 151.3, 138.2, 136.8, 129.9, 128.6 (2C), 128.1, 127.6 (2C), 125.3, 112.4, 112.0, 106.5, 70.2, 14.7; FT-IR (neat): ṽ [cm^{-1}]= 3367, 1692, 1599, 1536, 1511, 1493, 1454, 1418, 1393, 1314, 1247, 1213, 1193, 1156, 1083, 1050, 1009, 992, 922, 835, 786, 736, 700, 684, 673.

5-Chloro-*N*-(dibenzo[*b,d*]furan-1-yl)-2-(methylthio)pyrimidine-4-carboxamide (3t)

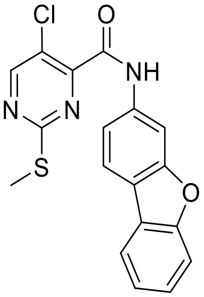

According to the general procedure (I), the title compound **3t** was obtained from **2a** and **1k** as a beige solid (214 mg, 82%); mp: 195 °C; ^{1}H NMR (300 MHz, DMSO-d_6) δ= 10.37 – 10.16 (m, 1H), 8.73 – 8.57 (m, 1H), 8.32 – 8.16 (m, 1H), 7.99 – 7.76 (m, 2H), 7.61 – 7.19 (m, 4H), 2.59 (s, 3H); ^{13}C NMR (75 MHz, DMSO-d_6) δ= 170.0, 156.1, 155.5, 126.2, 123.4, 123.3, 122.3, 122.1, 120.2, 120.1, 119.7, 114.9, 114.9, 111.1, 110.9, 103.1, 14.0; FT-IR (neat): ṽ [cm^{-1}]= 3333, 1687, 1597, 1513, 1453, 1419, 1391, 1310, 1212, 1047, 960, 854, 824, 771, 751, 737, 722, 673.

5-Chloro-2-(methylthio)-*N*-(3-(phenylcarbamoyl)phenyl)pyrimidine-4-carboxamide (3u)

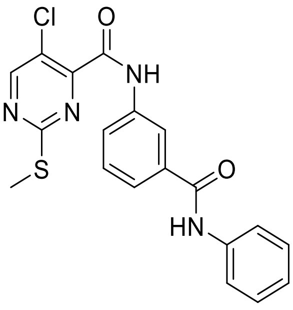

According to the general procedure (I), the title compound **3u** was obtained from **2a** and **1m** as a colourless solid (162 mg, 58%); mp: 168 °C; ^{1}H NMR (300 MHz, $CDCl_3$) δ= 11.04 (s, 1H), 10.33 (s, 1H), 8.97 (s, 1H), 8.30 – 8.12 (m, 1H), 7.97 – 7.86 (m, 1H), 7.84 – 7.70 (m, 3H), 7.56 (t, J = 7.9 Hz, 1H), 7.36 (t, J = 7.9 Hz, 2H), 7.21 – 6.98 (m, 1H), 2.60 (s, 3H); ^{13}C NMR (75 MHz, $CDCl_3$) δ= 169.9, 165.3, 161.3, 158.6, 157.4, 139.0, 138.0, 136.0, 129.1, 128.6 (2C), 123.7, 123.5, 122.7, 122.2, 120.3 (2C), 119.3, 14.1; FT-IR (neat): ṽ [cm^{-1}]= 3356, 1690, 1651, 1597, 1531, 1510, 1483, 1442, 1416, 1394, 1314, 1218, 1177, 1153, 1049, 963, 895, 810, 789, 751, 688, 667.

N-(3-benzoylphenyl)-5-bromo-2-(methylthio)pyrimidine-4-carboxamide (3v)

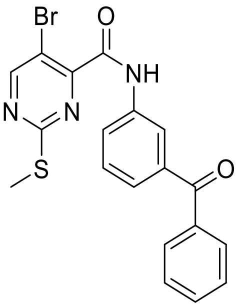

According to the general procedure (I), the title compound **3v** was obtained from **2b** and 3-benzoylaniline as a yellowish solid (276 mg, 92%); mp: 165 °C; ^{1}H NMR (300 MHz, $CDCl_3$) δ= 11.06 (s, 1H), 9.02 (s, 1H), 8.12 (t, J = 1.8 Hz, 1H), 8.03 – 7.95 (m, 1H), 7.80 – 7.74 (m, 2H), 7.73 – 7.66 (m, 1H), 7.64 – 7.49 (m, 4H), 2.57 (s, 3H); ^{13}C NMR (75 MHz, $CDCl_3$) δ= 195.4, 170.4, 162.1, 160.7, 159.1, 138.1, 137.7, 136.8, 132.8, 129.6 (2C), 129.4, 128.6 (2C), 125.7, 123.6, 120.6, 110.9, 14.0; FT-IR (neat): ṽ [cm^{-1}]= 3324, 1691, 1646, 1594, 1528, 1505, 1484, 1447, 1414, 1393, 1313, 1295, 1259, 1212, 1177, 1155, 1026, 999, 968, 893, 863, 810, 787, 719, 706, 693, 671.

5-Bromo-2-(methylthio)-N-(3-phenoxyphenyl)pyrimidine-4-carboxamide (3w)

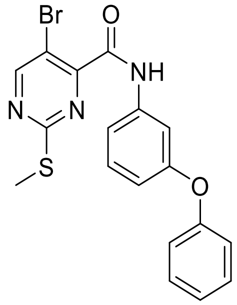

According to the general procedure (I), the title compound **3w** was obtained from **2b** and 3-phenoxyaniline as a yellowish solid (250 mg, 85%); mp: 136 °C; ^{1}H NMR (300 MHz, $CDCl_3$) δ= 9.61 (s, 1H), 8.81 (s, 1H), 7.54 – 7.41 (m, 2H), 7.39 – 7.29 (m, 3H), 7.16 – 7.08 (m, 1H), 7.06 – 6.99 (m, 2H), 6.90 – 6.75 (m, 1H), 2.61 (s, 3H); ^{13}C NMR (75 MHz, $CDCl_3$) δ= 170.7, 163.4, 159.4, 157.9, 157.0, 153.0, 138.3, 130.2, 129.8 (2C), 123.5, 119.0 (2C), 115.5, 114.9, 112.5, 110.9, 14.6; FT-IR (neat): ṽ [cm^{-1}]= 3365, 1693, 1589, 1530, 1509, 1484, 1418, 1393, 1308, 1250, 1212, 1174, 1157, 1027, 964, 915, 865, 806, 786, 772, 734, 687, 674.

2-(Methylthio)-N-(3-phenoxyphenyl)pyrimidine-4-carboxamide (3x)

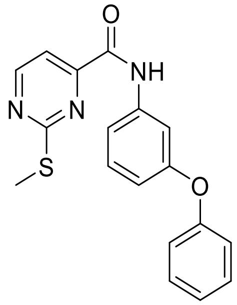

According to the general procedure (I), the title compound **3x** was obtained from **2c** and 3-phenoxyaniline as a beige solid (89 mg, 87%); mp: 117 °C; ^{1}H NMR (300 MHz, $CDCl_3$) δ= 10.19 – 10.02 (m, 1H), 9.20 (d, J = 5.0 Hz, 1H), 8.23 (d, J = 5.0 Hz, 1H), 7.92 – 7.84 (m, 2H), 7.82 – 7.71 (m, 3H), 7.59 – 7.51 (m, 1H), 7.50 – 7.43 (m, 2H), 7.30 – 7.16 (m, 1H), 3.05 (s, 3H); ^{13}C NMR (75 MHz, $CDCl_3$) δ= 172.4, 160.1, 159.8, 158.1, 156.9, 156.5, 138.3, 130.3, 129.9 (2C), 123.6, 119.2 (2C), 115.3, 114.8, 113.5, 110.6, 14.4; FT-IR (neat): ṽ [cm^{-1}]= 3328, 1691, 1669, 1587, 1513, 1486, 1445, 1415, 1320, 1218, 1155, 1124, 1056, 1022, 968, 859, 762, 738, 692.

5-Methyl-2-(methylthio)-*N*-(3-phenoxyphenyl)pyrimidine-4-carboxamide (3y)

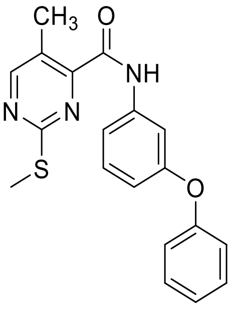

A solution of 2-(methylthio)pyrimidine **3w** (125 mg, 0.30 mmol) and $Pd(PPh)_3$ (35 mg, 0.03 mmol) in anhydrous tetrahydrofuran (5 mL) was thoroughly degassed with argon and transferred to an argon-flushed sealed tube. Trimethylaluminium (1.2 mL, 2 M in toluene) was added via septum and the reaction mixture was heated for 2 h to 70 °C. The reaction was quenched by the addition of water (1 mL) and evaporation of the volatiles. Purification by silica column chromatography (CH/EA 5:1) afforded the title compound **3y** as a colourless solid (35 mg, 33%); mp: 112 °C; ^{1}H NMR (300 MHz, $CDCl_3$) δ= 9.88 (s, 1H), 8.54 (s, 1H), 7.51 – 7.41 (m, 2H), 7.40 – 7.28 (m, 3H), 7.16 – 7.07 (m, 1H), 7.07 – 7.00 (m, 2H), 6.85 – 6.73 (m, 1H), 2.66 (s, 3H), 2.61 (s, 3H); ^{13}C NMR (75 MHz, $CDCl_3$) δ= 169.1, 162.6, 161.8, 157.9, 157.1, 152.8, 138.7, 130.2, 129.9 (2C), 126.6, 123.5, 119.0 (2C), 115.2, 114.8, 110.8, 16.8, 14.3; FT-IR (neat): ṽ [cm^{-1}]= 3314, 2970, 1697, 1640, 1588, 1525, 1448, 1387, 1361, 1278, 1250, 1170, 1149, 1065, 1041, 968, 918, 897, 873, 762, 720, 688.

5-Amino-*N*-(3-benzoylphenyl)-2-(methylthio)pyrimidine-4-carboxamide (3z)

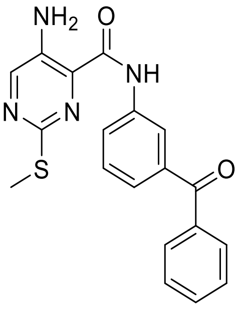

According to the general procedure (I), the title compound **3z** was obtained from **2d** and 3-benzoylaniline as a yellow solid (73 mg, 50%); mp: 178 °C; ^{1}H NMR (300 MHz, DMSO-d_6) δ= 10.43 (s, 1H), 8.28 (t, J = 1.9 Hz, 2H), 8.09 – 7.97 (m, 1H), 7.85 – 7.46 (m, 7H), 6.74 – 6.58 (m, 2H), 2.57 (s, 3H); ^{13}C NMR (75 MHz, DMSO-d_6) δ= 195.6, 164.8, 154.7, 150.4, 139.8, 138.0, 137.5, 137.0, 132.8, 132.8, 129.7 (2C), 129.0, 128.6 (2C), 125.4, 124.8, 121.4, 13.8; FT-IR (neat): ṽ [cm^{-1}]= 3443, 3324, 1678, 1644, 1585, 1527, 1436, 1410, 1317, 1273, 1237, 1206, 1141, 1107, 985, 921, 806, 783, 710, 683, 664.

4 Synthesis of 2-(methylsulfonyl)pyrimidines (4, 5a–k, 6a–n, 7a–e)

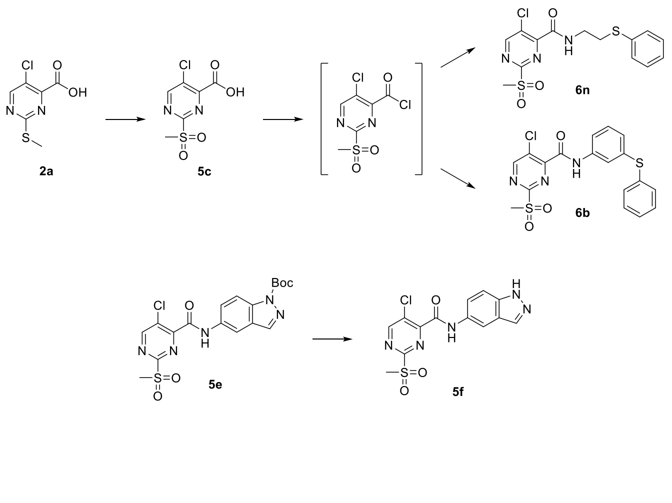

General procedure (II) for the synthesis of 2-(methylsulfonyl)pyrimidines

2-(methylsulfonyl)pyrimidines were synthesized according to a procedure by Webb. To a solution of the respective 2-(methylthio)pyrimidine **3a–z** (1.0 eq.) in tetrahydrofuran/water (1:1) potassium peroxymonosulfate (3.0 eq., OXONE®) was added and the mixture was stirred for 16 h at room temperature. The solvent was evaporated and the residual was extracted from dichloromethane/water. Purification by silica column chromatography (isocratic, CH/EA 5:1–2:1) yielded the corresponding 2-(methylsulfonyl)pyrimidines (**4, 5a–k, 6a–m, 7a–e**)

N-(2-benzylphenyl)-5-chloro-2-(methylsulfonyl)pyrimidine-4-carboxamide (4)

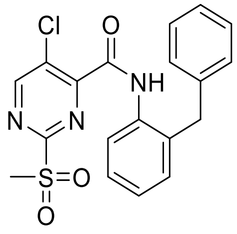

According to the general procedure (II), the title compound **4** was obtained from **3a** as a yellowish solid (52 mg, 86%); mp: 250 °C; ^{1}H NMR (300 MHz, $CDCl_3$) δ= 9.26 (s, 1H), 8.94 (s, 1H), 8.03 (d, *J* = 8.0 Hz, 1H), 7.39 – 7.00 (m, 8H), 4.03 (s, 2H), 3.19 (s, 3H); ^{13}C NMR (75 MHz, $CDCl_3$) δ= 162.0, 160.1, 158.0, 153.7, 138.6, 134.5, 133.6, 132.1, 131.2, 129.0 (2C), 128.6 (2C), 127.8, 126.8, 126.4, 123.4, 39.7, 38.2; FT-IR (neat): ṽ [cm^{-1}]= 3380, 1737, 1692, 1584, 1522, 1465, 1451, 1396, 1308, 1178, 1145, 1131, 1091, 1057, 963, 917, 787, 761, 731, 712, 696, 670; ESI-MS *m/z*: [M+H]$^{+}$ 402.15 (100%), 403.37 (4.82%), 404.12 (33.88%); purity (HPLC)= 95.6%.

5-Chloro-2-(methylsulfonyl)-*N*-(3-phenoxyphenyl)pyrimidine-4-carboxamide (5a)

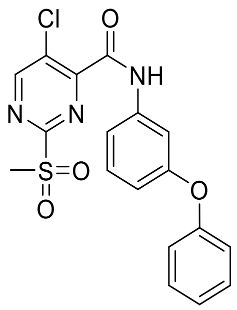

According to the general procedure (II), the title compound **5a** was obtained from **3b** as a yellowish solid (34 mg, 49%); mp: 156 °C; ^{1}H NMR (300 MHz, DMSO-d_6) δ= 11.05 (s, 1H), 9.44 (s, 1H), 7.51 – 7.35 (m, 5H), 7.18 (t, *J* = 7.6 Hz, 1H), 7.08 (d, *J* = 7.6 Hz, 2H), 6.85 (dt, *J* = 7.6, 2.0 Hz, 1H), 3.49 (s, 3H); ^{13}C NMR (75 MHz, DMSO-d_6) δ= 162.8, 160.3, 158.3, 157.3, 156.1, 139.1, 130.6, 130.1 (2C), 129.9, 123.9, 119.1 (2C), 114.7, 114.6, 109.5, 39.4; FT-IR (neat): ṽ [cm^{-1}]= 3332, 1692, 1610, 1588, 1529, 1484, 1429, 1392, 1319, 1302, 1261, 1208, 1183, 1157, 1136, 1059, 1023, 973, 912, 870, 809, 787, 777, 733, 690, 671; ESI-MS *m/z*: [M+H]$^{+}$ 404.08 (100%), 405.28 (6.56%), 406.09 (33.7%); purity (HPLC)= 95.4%.

5-Chloro-2-(methylsulfonyl)-*N*-(2-oxo-2-((3-phenoxyphenyl)amino)ethyl)pyrimidine-4-carboxamide (5b)

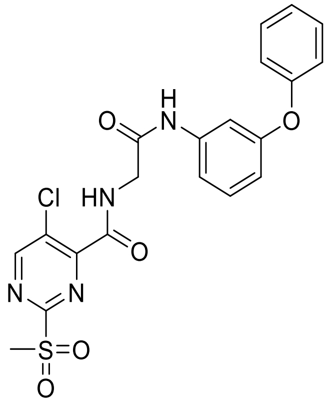

According to the general procedure (II), the title compound **5b** was obtained from **3c** as a colourless solid (50 mg, 60%); mp: 91 °C; ^{1}H NMR (300 MHz, DMSO-d_6) δ= 10.24 (s, 1H), 9.36 (s, 1H), 9.28 (t, J = 5.8 Hz, 1H), 7.45 – 7.36 (m, 2H), 7.35 – 7.29 (m, 3H), 7.21 – 7.11 (m, 1H), 7.07 – 6.99 (m, 2H), 6.77 – 6.68 (m, 1H), 4.14 (d, J = 5.8 Hz, 2H), 3.48 (s, 3H); ^{13}C NMR (75 MHz, DMSO-d_6) δ= 166.6, 162.5, 161.9, 160.5, 157.2, 156.3, 140.2, 130.5, 130.2, 130.0 (2C), 123.6, 118.9 (2C), 113.8, 113.3, 108.8, 42.8, 39.4; FT-IR (neat): ṽ [cm^{-1}]= 3340, 2361, 1674, 1589, 1508, 1486, 1440, 1315, 1216, 1163, 1133, 1006, 961, 874, 767, 689, 664; ESI-MS *m/z*: [M+H]$^{+}$ 461.28 (100%), 463.19 (62.9%), 464.22 (3.48%); purity (HPLC)= 95.3%.

5-Chloro-2-(methylsulfonyl)pyrimidine-4-carboxylic acid (5c)

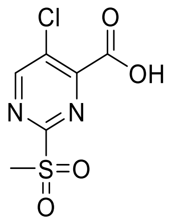

According to the general procedure (II), the title compound **5c** was obtained from **2a** as a beige solid (70 mg, 98%); mp: 180 °C; ^{1}H NMR (300 MHz, DMSO-d_6) δ= 9.38 (s, 1H), 3.44 (s, 3H); ^{13}C NMR (75 MHz, DMSO-d_6) δ= 163.5, 162.8, 160.4, 156.8, 129.6, 39.4; FT-IR (neat): ṽ [cm^{-1}]= 2930, 1728, 1568, 1433, 1395, 1306, 1277, 1231, 1193, 1158, 1141, 1065, 1036, 973, 937, 896, 811, 774, 749, 732, 657; ESI-MS *m/z*: [M+H]$^{+}$ 236.92 (100%), 237.91 (10.7%), 238.92 (38.09%); purity (HPLC)= 95.7%.

5-Chloro-*N*-(4'-cyano-[1,1'-biphenyl]-4-yl)-2-(methylsulfonyl)pyrimidine-4-carboxamide (5d)

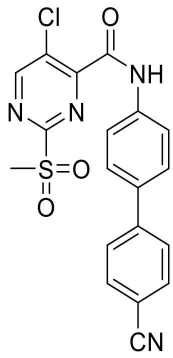

According to the general procedure (II), the title compound **5d** was obtained from **3d** as a yellow solid (38 mg, 54%); mp: 267 °C; ^{1}H NMR (300 MHz, DMSO-d_6) δ= 11.17 (s, 1H), 9.47 (s, 1H), 7.96 – 7.87 (m, 4H), 7.85 (s, 4H), 3.52 (s, 3H); ^{13}C NMR (75 MHz, DMSO-d_6) δ= 162.8, 160.4, 158.3, 143.8, 138.2, 134.4, 132.9 (2C), 130.0, 127.8 (2C), 127.2 (2C), 120.4 (2C), 118.9, 109.8, 39.4; FT-IR (neat): ṽ [cm^{-1}]= 2227, 1688, 1605, 1590, 1525, 1509, 1420, 1398, 1315, 1229, 1187, 1146, 1128, 1058, 974, 949, 819, 800, 780, 764, 741, 676; ESI-MS *m/z*: [M+H]$^{+}$ 413.07 (100%), 414.14 (15.8%), 415.06 (60.82%); purity (HPLC)= 95.3%.

***Tert*-butyl 5-(5-chloro-2-(methylsulfonyl)pyrimidine-4-carboxamido)-1*H*-indazole-1-carboxylate (5e)**

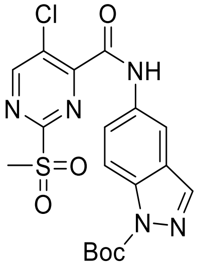

According to the general procedure (II), the title compound **5e** was obtained from **3e** as a yellow solid (83 mg, 76%); mp: 180 °C; ^{1}H NMR (300 MHz, $CDCl_3$) δ= 9.82 (s, 1H), 9.04 (s, 1H), 8.35 (d, *J* = 2.1 Hz, 1H), 8.21 – 8.11 (m, 2H), 7.64 (dd, *J* = 8.9, 2.1 Hz, 1H), 3.41 (s, 3H), 1.72 (s, 9H); ^{13}C NMR (75 MHz, $CDCl_3$) δ= 162.3, 161.9, 158.2, 154.0, 149.1, 139.6, 137.4, 133.6, 132.6, 126.2, 122.6, 115.3, 112.4, 85.3, 39.7, 28.2 (3C); FT-IR (neat): ṽ [cm^{-1}]= 1737, 1693, 1508, 1393, 1354, 1318, 1289, 1243, 1152, 1131, 1029, 980, 902, 848, 819, 758, 686.

5-Chloro-*N*-(1*H*-indazol-5-yl)-2-(methylsulfonyl)pyrimidine-4-carboxamide (5f)

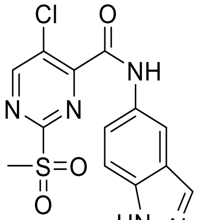

The Boc-protected 2-(methylsulfonyl)pyrimidine **5e** (42 mg, 0.10 mmol) was suspended in dichloromethane (3.0 mL). Subsequently, trifluoroacetic acid (1.5 mL) was added and the reaction mixture was stirred for 16 h at room temperature. Evaporation of the solvent and lyophilization from acetonitrile/water (1:1) yielded the title compound **5f** as a yellow solid (35 mg, 99%); mp: 231 °C; ^{1}H NMR (300 MHz, DMSO-*d*$_6$) δ= 13.12 (s, 1H), 10.99 (s, 1H), 9.45 (s, 1H), 8.28 – 8.22 (m, 1H), 8.11 (s, 1H), 7.63 – 7.47 (m, 2H), 3.51 (s, 3H); ^{13}C NMR (75 MHz, DMSO-*d*$_6$) δ= 162.8, 160.2, 160.1, 158.8, 130.6, 130.0, 129.8, 122.6, 120.3, 111.0, 106.3, 99.9, 39.3; FT-IR (neat): ṽ [cm^{-1}]= 3307, 3012, 2929, 1687, 1587, 1536, 1501, 1455, 1404, 1312, 1292, 1257, 1218, 1187, 1132, 1067, 1052, 965, 940, 894, 855, 798, 753, 730, 665; ESI-MS *m/z*: [M+H]$^+$ 352.06 (100%), 353.05 (15.16%), 354.04 (34.78%); purity (HPLC)= 99.5%.

5-Chloro-2-(methylsulfonyl)-*N*-(4-(*p*-tolyl)thiazol-2-yl)pyrimidine-4-carboxamide (5g)

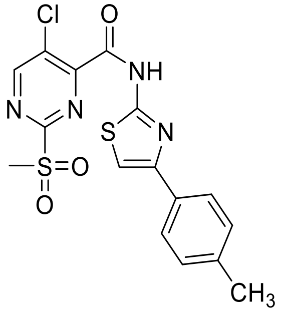

According to the general procedure (II), the title compound **5g** was obtained from **3f** as a yellow solid (18 mg, 33%); mp: 280 °C; ^{1}H NMR (300 MHz, DMSO-*d*$_6$) δ= 13.22 (s, 1H), 9.43 (d, *J* = 29.4 Hz, 1H), 7.82 (d, *J* = 7.9 Hz, 2H), 7.76 (d, *J* = 3.2 Hz, 1H), 7.26 (d, *J* = 7.9 Hz, 2H), 3.53 (s, 3H), 2.33 (s, 3H); ^{13}C NMR (75 MHz, DMSO-*d*$_6$) δ= 171.4, 162.7, 160.7, 156.3, 137.4, 131.2, 131.1, 130.6, 129.3 (2C), 128.5, 125.7 (2C), 108.8, 39.8, 20.8; FT-IR (neat): ṽ [cm^{-1}]= 3384, 1701, 1534, 1445, 1391, 1323, 1303, 1184, 1143, 1067, 1054, 962, 932, 824, 750, 737, 710, 674; ESI-MS *m/z*: [M+H]$^+$ 409.14 (100%), 410.13 (20.03%), 411.12 (42.91%); purity (HPLC)= 97.7%.

(5-Chloro-2-(methylsulfonyl)pyrimidin-4-yl)(3,4-dihydroisoquinolin-2(1*H*)-yl)methanone (5h)

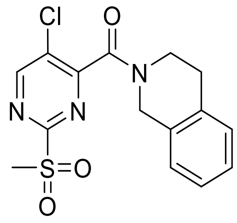

According to the general procedure (II), the title compound **5h** was obtained from **3g** as a yellowish solid (20 mg, 28%); mp: 218 °C; ^{1}H NMR (300 MHz, DMSO-d_6) δ= 9.50 – 9.25 (m, 1H), 7.39 – 7.09 (m, 4H), 4.95 – 4.40 (m, 2H), 3.97 – 3.49 (m, 2H), 3.42 (d, *J* = 8.5 Hz, 3H), 3.01 – 2.74 (m, 2H); ^{13}C NMR (75 MHz, DMSO-d_6) δ= 163.2, 161.5, 161.3, 159.8, 159.8, 159.7, 134.0, 133.7, 132.5, 131.9, 129.6, 128.7, 128.6, 126.8, 126.6, 126.5, 126.4, 126.2, 126.0, 46.8, 43.3, 39.9, 28.6, 27.4; FT-IR (neat): ṽ [cm^{-1}]= 2925, 1644, 1483, 1459, 1402, 1372, 1311, 1255, 1188, 1134, 1066, 1044, 964, 927, 814, 794, 770, 757, 714; ESI-MS *m/z*: [M+H]$^+$ 352.10 (100%), 353.08 (13.95%), 354.07 (31.77%); purity (HPLC)= 96.5%.

5-Chloro-2-(methylsulfonyl)-*N*-(pentan-3-yl)pyrimidine-4-carboxamide (5i)

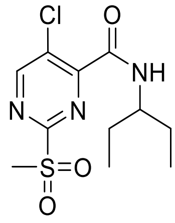

According to the general procedure (II), the title compound **5i** was obtained from **3h** as a yellowish solid (55 mg, 71%); mp: 146 °C; ^{1}H NMR (300 MHz, $CDCl_3$) δ= 8.99 (s, 1H), 7.32 (d, *J* = 9.2 Hz, 1H), 4.08 – 3.86 (m, 1H), 3.38 (s, 3H), 1.82 – 1.46 (m, 4H), 0.96 (t, *J* = 7.4 Hz, 6H); ^{13}C NMR (75 MHz, $CDCl_3$) δ= 162.4, 161.5, 160.2, 154.7, 133.2, 53.3, 39.7, 27.4 (2C), 10.5 (2C); FT-IR (neat): ṽ [cm^{-1}]= 3268, 2966, 1650, 1533, 1459, 1413, 1318, 1197, 1125, 1060, 969, 954, 926, 782, 715, 676; ESI-MS *m/z*: [M+H]$^+$ 306.07 (100%), 307.07 (9.57%), 308.07 (40.32%); purity (HPLC)= 99.3%.

***N,N*-dibenzyl-5-chloro-2-(methylsulfonyl)pyrimidine-4-carboxamide (5k)**

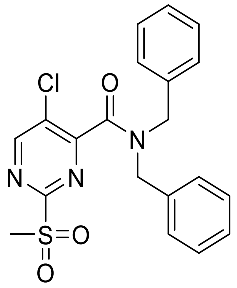

According to the general procedure (II), the title compound **5k** was obtained from **3i** as a yellowish solid (74 mg, 89%); mp: 68 °C; ^{1}H NMR (300 MHz, $CDCl_3$) δ= 8.87 (s, 1H), 7.45 – 7.24 (m, 8H), 7.18 – 7.10 (m, 2H), 4.74 (s, 2H), 4.24 (s, 2H), 3.23 (s, 3H); ^{13}C NMR (75 MHz, $CDCl_3$) δ= 163.4, 160.8, 158.9, 135.4, 134.5, 131.0, 129.0 (2C), 129.0 (2C), 128.7 (2C), 128.4, 128.2, 127.7 (2C), 50.9, 47.7, 39.4; FT-IR (neat): ṽ [cm^{-1}]= 2926, 1769, 1646, 1553, 1495, 1473, 1452, 1394, 1317, 1184, 1132, 1080, 1061, 1029, 1001, 958, 796, 747, 715, 697; ESI-MS *m/z*: [M+H]$^+$ 438.04 (100%), 439.03 (19.28%), 440.02 (32.13%); purity (HPLC)= 96.9%.

N-(4-benzoylphenyl)-5-chloro-2-(methylsulfonyl)pyrimidine-4-carboxamide (6a)

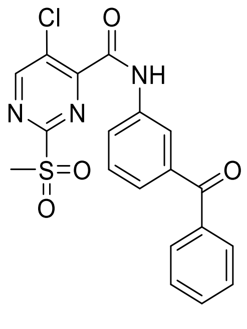

According to the general procedure (II), the title compound **6a** was obtained from **3k** as a yellowish solid (80 mg, 64%); mp: 175 °C; ^{1}H NMR (300 MHz, DMSO-d_6) δ= 11.24 (s, 1H), 9.46 (s, 1H), 8.19 – 8.07 (m, 1H), 8.02 (d, J = 8.0 Hz, 1H), 7.81 – 7.74 (m, 2H), 7.73 – 7.53 (m, 5H), 3.51 (s, 3H); ^{13}C NMR (75 MHz, DMSO-d_6) δ= 194.3, 162.8, 160.5, 160.4, 158.0, 137.8, 137.7, 136.8, 132.8, 130.0, 129.6 (2C), 129.5, 128.6 (2C), 126.0, 123.8, 121.4, 39.4; FT-IR (neat): ṽ [cm^{-1}]= 3303, 1677, 1650, 1588, 1533, 1513, 1482, 1401, 1323, 1292, 1225, 1135, 1058, 962, 904, 854, 758, 721, 707, 694, 669; ESI-MS *m/z*: [M+H]$^{+}$ 416.04 (100%), 417.05 (22.14%), 418.03 (36.43%); purity (HPLC)= 99.2%.

5-Chloro-2-(methylsulfonyl)-N-(3-(phenylthio)phenyl)pyrimidine-4-carboxamide (6b)

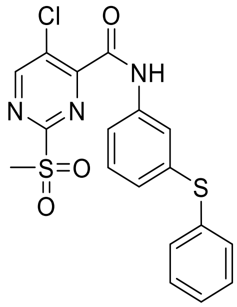

At 0 °C, 5-chloro-2-(methylsulfonyl)pyrimidine-4-carboxylic acid **5c** (106 mg, 0.45 mmol) and single drop of dimethylformamide were added stepwise to thionyl chloride (580 µL, 8.00 mmol). The mixture was refluxed for 1 h and evaporated *in vacuo.* Subsequently, the isolated acyl chloride was added dropwise to a solution of the arylamine **1d** (81 mg, 0.40 mmol) and triethylamine (168 µL, 1.20 mmol) in dichloromethane (10 mL). The reaction mixture was stirred for 16 h at room temperature, evaporated and resolved in ethyl acetate. The organic phase was extracted with $NaHCO_3$ sat. and HCl (1 M). Purification by silica column chromatography (CH/EA 2:1) afforded the title compound **6b** as a yellow solid (66 mg, 39%); mp: 135 °C; ^{1}H NMR (300 MHz, $CDCl_3$) δ= 9.51 (s, 1H), 8.94 (s, 1H), 7.61 – 7.49 (m, 2H), 7.38 – 7.12 (m, 6H), 7.08 – 6.94 (m, 1H), 3.29 (s, 3H); ^{13}C NMR (75 MHz, $CDCl_3$) δ= 162.2, 161.8, 158.1, 154.2, 137.7, 137.3, 134.8, 133.5, 131.9 (2C), 129.9, 129.4 (2C), 127.6, 127.5, 122.1, 119.0, 39.7; FT-IR (neat): ṽ [cm^{-1}]= 3329, 1689, 1592, 1523, 1513, 1478, 1438, 1407, 1386, 1319, 1301, 1226, 1181, 1151, 1135, 1081, 1059, 1025, 972, 881, 858, 821, 780, 769, 745, 684, 665; ESI-MS *m/z*: [M+H]$^{+}$ 420.14 (100%), 421.08 (11.65%), 422.12 (34.38%); purity (HPLC)= 95.4%.

5-Chloro-N-(3-(cyclohexyloxy)phenyl)-2-(methylsulfonyl)pyrimidine-4-carboxamide (6c)

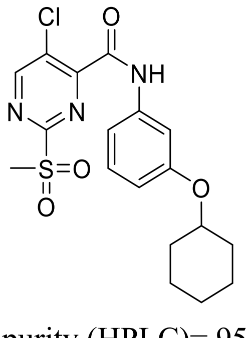

According to the general procedure (II), the title compound **6c** was obtained from **3l** as a yellowish solid (46 mg, 37%); mp: 144 °C; ^{1}H NMR (300 MHz, $CDCl_3$) δ= 9.40 (s, 1H), 8.97 (s, 1H), 7.33 (t, J = 2.2 Hz, 1H), 7.25 – 7.08 (m, 2H), 6.74 – 6.64 (m, 1H), 4.30 – 4.16 (m, 1H), 3.34 (s, 3H), 2.01 – 1.81 (m, 2H), 1.81 – 1.62 (m, 2H), 1.57 – 1.14 (m, 6H); ^{13}C NMR (75 MHz, $CDCl_3$) δ= 162.3, 162.0, 158.6, 157.8, 154.2, 137.6, 133.6, 130.0, 113.7, 112.6, 108.4, 75.6, 39.8, 31.8 (2C), 25.7, 23.7 (2C); FT-IR (neat): ṽ [cm^{-1}]= 3344, 2935, 1702, 1604, 1540, 1495, 1422, 1389, 1325, 1274, 1205, 1146, 1130, 1049, 1020, 979, 850, 785, 765, 730, 690, 669; ESI-MS *m/z*: [M+Na]$^{+}$ 432.15 (100%), 433.12 (15.81%), 434.14 (44.39%); purity (HPLC)= 95.6%.

N-(3-benzylphenyl)-5-chloro-2-(methylsulfonyl)pyrimidine-4-carboxamide (6d)

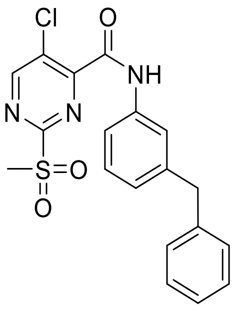

According to the general procedure (II), the title compound **6d** was obtained from **3m** as a yellow solid (88 mg, 87%); mp: 148 °C; ^{1}H NMR (300 MHz, $CDCl_3$) δ= 9.53 (s, 1H), 9.01 (s, 1H), 7.65 – 7.49 (m, 2H), 7.36 – 7.24 (m, 3H), 7.24 – 7.14 (m, 3H), 7.09 – 6.99 (m, 1H), 4.00 (s, 2H), 3.38 (s, 3H); ^{13}C NMR (75 MHz, $CDCl_3$) δ= 162.2, 161.8, 160.0, 158.0, 154.3, 142.5, 140.7, 136.7, 133.4, 129.3, 129.0 (2C), 128.6 (2C), 126.4, 126.3, 120.9, 118.3, 41.9, 39.7; FT-IR (neat): ṽ [cm^{-1}]= 3336, 1686, 1593, 1534, 1494, 1443, 1393, 1305, 1189, 1145, 1058, 972, 898, 820, 785, 761, 732, 695, 669; ESI-MS *m/z*: [M+H]$^{+}$ 402.13 (100%), 403.18 (17.85%), 404.17 (32.18%); purity (HPLC)= 98.4%.

5-Chloro-N-(3-methoxyphenyl)-2-(methylsulfonyl)pyrimidine-4-carboxamide (6e)

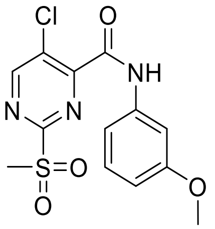

According to the general procedure (II), the title compound **6e** was obtained from **3n** as a yellow solid (113 mg, 86%); mp: 185 °C; ^{1}H NMR (300 MHz, DMSO-d_6) δ= 10.97 (s, 1H), 9.44 (s, 1H), 7.37 – 7.21 (m, 3H), 6.84 – 6.74 (m, 1H), 3.77 (s, 3H), 3.50 (s, 3H); ^{13}C NMR (75 MHz, DMSO-d_6) δ= 172.0, 162.8, 160.3, 159.7, 158.6, 138.8, 130.0, 129.9, 112.1, 110.4, 105.7, 55.1, 39.5; FT-IR (neat): ṽ [cm^{-1}]= 3339, 1687, 1596, 1531, 1454, 1432, 1392, 1307, 1292, 1268, 1212, 1178, 1161, 1147, 1058, 1044, 971, 865, 818, 766, 738, 690, 668; ESI-MS *m/z*: [M+H]$^{+}$ 342.08 (100%), 343.10 (22.39%), 344.09 (29.87%); purity (HPLC)= 98.1%.

N-([1,1'-biphenyl]-3-yl)-5-chloro-2-(methylsulfonyl)pyrimidine-4-carboxamide (6f)

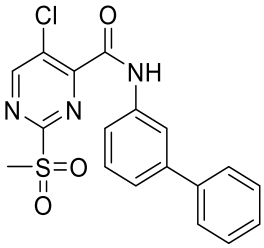

According to the general procedure (II), the title compound **6f** was obtained from **3o** as a yellowish solid (122 mg, 78%); mp: 164 °C; ^{1}H NMR (300 MHz, DMSO-d_6) δ= 11.10 (s, 1H), 9.46 (s, 1H), 8.04 – 7.97 (m, 1H), 7.77 – 7.61 (m, 3H), 7.57 – 7.46 (m, 4H), 7.45 – 7.27 (m, 1H), 3.52 (s, 3H); ^{13}C NMR (75 MHz, DMSO-d_6) δ= 162.8, 160.3 (2C), 158.4, 141.2, 139.7, 138.2, 130.0, 129.8, 129.0 (2C), 127.8, 126.7 (2C), 123.3, 119.0, 118.2, 39.4; FT-IR (neat): ṽ [cm^{-1}]= 3289, 1675, 1593, 1576, 1539, 1500, 1453, 1422, 1400, 1350, 1310, 1219, 1153, 1136, 1121, 1055, 967, 948, 881, 793, 751, 691, 668; ESI-MS *m/z*: [M+H]$^{+}$ 388.12 (100%), 389.18 (27.86%), 390.15 (24.35%); purity (HPLC)= 97.6%.

5-Chloro-2-(methylsulfonyl)-N-(2-phenoxyphenyl)pyrimidine-4-carboxamide (6g)

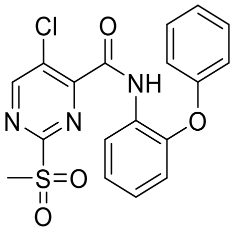

According to the general procedure (II), the title compound **6g** was obtained from **3p** as a yellowish solid (57 mg, 47%); mp: 177 °C; ^{1}H NMR (300 MHz, $CDCl_3$) δ= 10.17 (s, 1H), 9.06 (s, 1H), 8.59 (dd, J = 8.0, 1.7 Hz, 1H), 7.40 – 7.31 (m, 2H), 7.25 – 7.09 (m, 3H), 7.09 – 7.03 (m, 2H), 6.99 (dd, J = 8.0, 1.7 Hz, 1H), 3.21 (s, 3H); ^{13}C NMR (75 MHz, $CDCl_3$) δ= 162.6, 162.4, 157.6, 156.4, 153.0, 146.1, 133.4, 130.2 (2C), 128.8, 125.7, 124.6, 124.1, 120.8, 118.7 (2C), 118.2, 39.3; FT-IR (neat): ṽ [cm^{-1}]= 3360, 1705, 1602, 1588, 1517, 1447, 1414, 1389, 1311, 1249,

1210, 1174, 1142, 1102, 1054, 1029, 967, 873, 849, 802, 784, 767, 748, 694, 669; ESI-MS *m/z*: $[M+H]^+$ 404.14 (100%), 405.13 (19.81%), 406.13 (40.53%); purity (HPLC)= 97.5%.

5-Chloro-2-(methylsulfonyl)-*N*-(4-phenoxyphenyl)pyrimidine-4-carboxamide (6h)

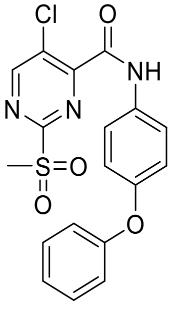

According to the general procedure (II), the title compound **6h** was obtained from **3q** as a yellowish solid (75 mg, 61%); mp: 195 °C; ^1H NMR (300 MHz, $CDCl_3$) δ= 9.61 (s, 1H), 9.03 (s, 1H), 7.78 – 7.61 (m, 2H), 7.34 (t, *J* = 7.7 Hz, 2H), 7.11 (t, *J* = 7.4 Hz, 1H), 7.06 – 6.96 (m, 4H), 3.40 (s, 3H); ^{13}C NMR (75 MHz, $CDCl_3$) δ= 162.3, 161.9, 157.9, 157.2, 154.8, 154.2, 133.6, 131.9, 129.9 (2C), 123.5, 122.2 (2C), 119.5 (2C), 118.9 (2C), 39.8; FT-IR (neat): ṽ [cm^{-1}]= 3297, 1669, 1587, 1513, 1483, 1403, 1323, 1254, 1227, 1160, 1125, 1058, 1023, 967, 927, 876, 855, 840, 766, 734, 693, 669; ESI-MS *m/z*: $[M+H]^+$ 404.14 (100%), 405.17 (21.05%), 406.16 (36.77%); purity (HPLC)= 96.6%.

N-(6-chloro-2-(methylsulfonyl)pyrimidin-4-yl)-2-phenoxybenzamide (6i)

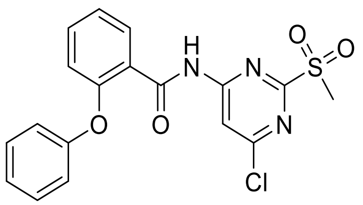

According to the general procedure (II), the title compound **6i** was obtained from **3r** as a colourless solid (14 mg, 57%); mp: 184 °C; ^1H NMR (300 MHz, $CDCl_3$) δ= 10.48 (s, 1H), 8.54 (s, 1H), 8.17 (dd, *J* = 7.9, 1.8 Hz, 1H), 7.46 – 7.30 (m, 3H), 7.23 – 7.13 (m, 2H), 7.10 – 7.01 (m, 2H), 6.82 (dd, *J* = 8.4, 1.1 Hz, 1H), 3.21 (s, 3H); ^{13}C NMR (75 MHz, $CDCl_3$) δ= 165.2, 164.2, 163.8, 159.7, 156.3, 154.5, 135.1, 132.8, 130.6 (2C), 125.7, 124.0, 121.5, 120.1 (2C), 118.2, 112.7, 39.0; FT-IR (neat): ṽ [cm^{-1}]= 3310, 1686, 1600, 1537, 1479, 1450, 1342, 1320, 1289, 1243, 1207, 1140, 1111, 1093, 975, 906, 863, 785, 770, 748, 708, 687; ESI-MS *m/z*: $[M+H]^+$ 404.12 (100%), 405.12 (23.57%), 406.13 (33.48%); purity (HPLC)= 99.0%.

N-(3-(benzyloxy)phenyl)-5-chloro-2-(methylsulfonyl)pyrimidine-4-carboxamide (6k)

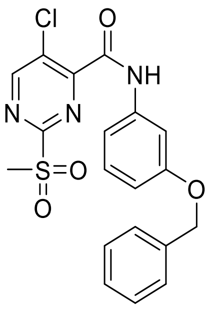

According to the general procedure (II), the title compound **6k** was obtained from **3s** as a yellowish solid (66 mg, 68%); mp: 154 °C; ^1H NMR (300 MHz, $CDCl_3$) δ= 9.52 (s, 1H), 8.94 (s, 1H), 7.45 (t, *J* = 2.2 Hz, 1H), 7.39 – 7.08 (m, 7H), 6.80 – 6.70 (m, 1H), 5.00 (s, 2H), 3.31 (s, 3H); ^{13}C NMR (75 MHz, $CDCl_3$) δ= 162.2, 161.9, 159.5, 158.0, 154.1, 137.7, 136.8, 133.5, 130.0, 128.7 (2C), 128.1, 127.6 (2C), 112.9, 112.6, 106.9, 70.2, 39.7; FT-IR (neat): ṽ [cm^{-1}]= 3282, 1675, 1596, 1536, 1441, 1402, 1382, 1351, 1310, 1277, 1202, 1121, 1053, 1027, 954, 871, 850, 767, 728, 690, 668; ESI-MS *m/z*: $[M+H]^+$ 418.15 (100%), 419.17 (30.79%), 421.16 (13.05%); purity (HPLC)= 98.7%.

5-Chloro-*N*-(dibenzo[*b*,*d*]furan-3-yl)-2-(methylsulfonyl)pyrimidine-4-carboxamide (6l)

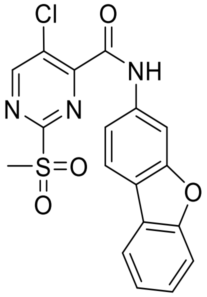

According to the general procedure (II), the title compound **6l** was obtained from **3t** as a yellow solid (68 mg, 42%); mp: 250 °C; ^{1}H NMR (300 MHz, DMSO-d_6) δ= 11.31 (s, 1H), 9.48 (s, 1H), 8.24 – 8.08 (m, 3H), 7.81 – 7.33 (m, 4H), 3.53 (s, 3H); ^{13}C NMR (75 MHz, DMSO-d_6) δ= 162.8, 160.3, 158.3, 155.9, 155.6, 137.2, 133.3, 130.0, 127.2, 123.3, 123.3, 121.5, 120.8, 120.3, 115.5, 111.6, 103.0, 39.5; FT-IR (neat): ṽ [cm^{-1}]= 1692, 1600, 1530, 1455, 1423, 1397, 1306, 1181, 1141, 1116, 1054, 962, 846, 831, 776, 752, 739, 719, 677, 654; ESI-MS *m/z*: [M+H]$^+$ 402.09 (100%), 403.07 (19.92%), 404.09 (44.97%); purity (HPLC)= 95.0%.

5-Chloro-2-(methylsulfonyl)-*N*-(3-(phenylcarbamoyl)phenyl)pyrimidine-4-carboxamide (6m)

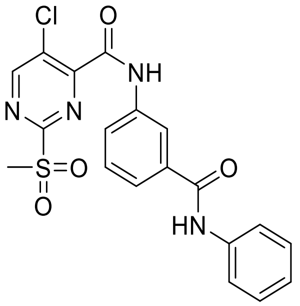

According to the general procedure (II), the title compound **6m** was obtained from **3u** as a yellowish solid (120 mg, 92%); mp: 237 °C; ^{1}H NMR (300 MHz, DMSO-d_6) δ= 11.20 (s, 1H), 10.35 (s, 1H), 9.47 (s, 1H), 8.21 (d, J = 2.0 Hz, 1H), 7.93 (d, J = 8.2 Hz, 1H), 7.83 – 7.73 (m, 3H), 7.59 (t, J = 7.9 Hz, 1H), 7.36 (t, J = 7.9 Hz, 2H), 7.11 (t, J = 7.4 Hz, 1H), 3.52 (s, 3H); ^{13}C NMR (75 MHz, DMSO-d_6) δ= 165.2, 162.8, 160.4, 160.4, 158.2, 139.0, 137.7, 136.0, 130.0, 129.2, 128.6 (2C), 123.8, 123.7, 122.9, 120.3 (2C), 119.5, 40.0; FT-IR (neat): ṽ [cm^{-1}]= 3370, 1683, 1655, 1598, 1531, 1484, 1442, 1420, 1394, 1324, 1257, 1212, 1177, 1148, 1128, 1054, 972, 896, 867, 833, 809, 770, 750, 728, 666; ESI-MS *m/z*: [M+H]$^+$ 431.15 (100%), 432.16 (24.92%), 433.15 (37.69%); purity (HPLC)= 95.0%.

5-Chloro-2-(methylsulfonyl)-*N*-(2-(phenylthio)ethyl)pyrimidine-4-carboxamide (6n)

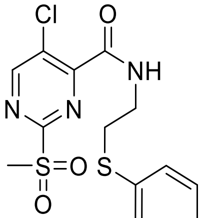

At 0 °C, 5-chloro-2-(methylsulfonyl)pyrimidine-4-carboxylic acid **5c** (130 mg, 0.55 mmol) and single drop of dimethylformamide were added stepwise to oxalyl chloride (428 µL, 5.00 mmol). The mixture was refluxed for 1 h and evaporated *in vacuo*. Subsequently, the isolated acyl chloride was added dropwise to a solution of the amine **1o** (134 mg, 0.50 mmol) and triethylamine (168 µL, 1.20 mmol) in dichloromethane (5 mL). The reaction mixture was stirred for 16 h at room temperature, evaporated and resolved in ethyl acetate. The organic phase was extracted with $NaHCO_3$ sat. and HCl (1 M). Purification by silica column chromatography (CH/EA 2:1) afforded the title compound **6n** as an orange oil (50 mg, 26%);^{1}H NMR (300 MHz, $CDCl_3$) δ= 8.90 (s, 1H), 8.07 – 7.99 (m, 1H), 7.37 – 7.25 (m, 2H), 7.25 – 7.14 (m, 2H), 7.14 – 7.02 (m, 1H), 3.59 (q, J = 6.6 Hz, 2H), 3.29 (s, 3H), 3.10 (t, J = 6.6 Hz, 2H); ^{13}C NMR (75 MHz, $CDCl_3$) δ= 162.2, 161.5, 160.4, 153.9, 134.7, 133.0, 130.0 (2C), 129.2 (2C), 126.7, 39.6, 39.2, 32.9; FT-IR (neat): ṽ [cm^{-1}]= 3299, 1667, 1531, 1515, 1477, 1439, 1404, 1317, 1284, 1209, 1177, 1131, 1073, 1033, 955, 831, 791, 764, 739, 687, 667; ESI-MS *m/z*: [M+H]$^+$ 372.02 (100%), 373.08 (18.01%), 374.01 (50.99%); purity (HPLC)= 96.0%.

N-(4-benzoylphenyl)-5-bromo-2-(methylsulfonyl)pyrimidine-4-carboxamide (7a)

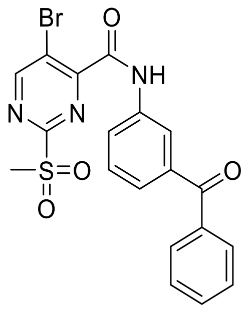

According to the general procedure (II), the title compound **7a** was obtained from **3v** as a yellowish solid (91 mg, 65%); mp: 174 °C; ^{1}H NMR (300 MHz, DMSO-*d*$_6$) δ= 11.23 (s, 1H), 9.53 (s, 1H), 8.11 (s, 1H), 8.00 (d, *J* = 7.9 Hz, 1H), 7.86 – 7.45 (m, 7H), 3.49 (s, 3H); ^{13}C NMR (75 MHz, DMSO-*d*$_6$) δ= 195.3, 163.4, 162.6, 161.3, 160.1, 137.9, 137.8, 136.8, 132.8, 129.6 (2C), 129.5, 128.6 (2C), 126.0, 123.7, 120.7, 119.5, 39.4; FT-IR (neat): ṽ [cm^{-1}]= 1691, 1652, 1589, 1530, 1483, 1434, 1396, 1311, 1177, 1133, 1035, 961, 860, 785, 762, 723, 709, 683, 668; ESI-MS *m/z*: [M+H]$^+$ 460.01 (100%), 461.01 (23.12%), 461.99 (97.81%); purity (HPLC)= 99.7%.

5-Bromo-2-(methylsulfonyl)-*N*-(3-phenoxyphenyl)pyrimidine-4-carboxamide (7b)

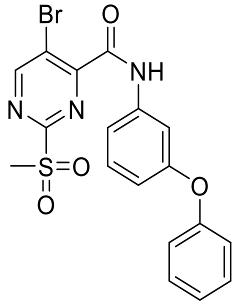

According to the general procedure (II), the title compound **7b** was obtained from **3w** as a yellowish solid (90 mg, 66%); mp: 153 °C; ^{1}H NMR (300 MHz, $CDCl_3$) δ= 9.56 (s, 1H), 9.27 – 9.13 (m, 1H), 7.49 – 7.40 (m, 2H), 7.39 – 7.29 (m, 3H), 7.16 – 7.07 (m, 1H), 7.07 – 6.97 (m, 2H), 6.91 – 6.78 (m, 1H), 3.39 (s, 3H); ^{13}C NMR (75 MHz, $CDCl_3$) δ= 164.7, 163.1, 158.3, 158.1, 156.9, 155.1, 137.9, 130.3, 129.9 (2C), 123.7, 121.8, 119.2 (2C), 116.0, 115.3, 111.3, 39.7; FT-IR (neat): ṽ [cm^{-1}]= 3330, 1691, 1669, 1611, 1587, 1507, 1483, 1420, 1319, 1302, 1256, 1210, 1182, 1156, 1126, 1057, 1036, 970, 912, 856, 839, 802, 767, 733, 691, 669; ESI-MS *m/z*: [M+H]$^+$ 448.12 (100%), 449.09 (22.43%), 450.37 (21.65%); purity (HPLC)= 97.2%.

2-(Methylsulfonyl)-*N*-(4-phenoxyphenyl)pyrimidine-4-carboxamide (7c)

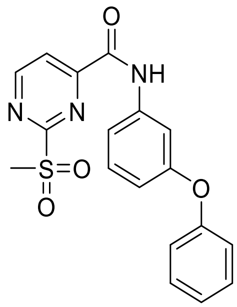

According to the general procedure (II), the title compound **7c** was obtained from **3x** as a yellowish solid (38 mg, 51%); mp: 149 °C; ^{1}H NMR (300 MHz, $CDCl_3$) δ= 9.76 (s, 1H), 9.13 (d, *J* = 5.0 Hz, 1H), 8.39 (d, *J* = 5.0 Hz, 1H), 7.51 (t, *J* = 2.2 Hz, 1H), 7.48 – 7.42 (m, 1H), 7.40 – 7.28 (m, 3H), 7.15 – 7.08 (m, 1H), 7.06 – 6.98 (m, 2H), 6.87 – 6.76 (m, 1H), 3.41 (s, 3H); ^{13}C NMR (75 MHz, $CDCl_3$) δ= 165.2, 160.8, 158.7, 158.7, 158.1, 156.7, 137.8, 130.3, 129.9 (2C), 123.8, 121.4, 119.2 (2C), 115.8, 115.2, 111.0, 39.5; FT-IR (neat): ṽ [cm^{-1}]= 3373, 1701, 1597, 1529, 1492, 1445, 1353, 1310, 1278, 1226, 1186, 1154, 1131, 975, 880, 797, 755, 697, 686, 672; ESI-MS *m/z*: [M+H]$^+$ 370.09 (100%), 371.10 (23.95%), 372.08 (4.81%); purity (HPLC)= 97.8%.

5-Methyl-2-(methylsulfonyl)-*N*-(4-phenoxyphenyl)pyrimidine-4-carboxamide (7d)

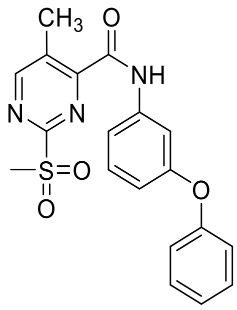

According to the general procedure (II), the title compound **7d** was obtained from **3y** as a yellow solid (54 mg, 70%); mp: 164 °C; ^{1}H NMR (300 MHz, DMSO-d_6) δ= 10.79 (d, J = 18.0 Hz, 1H), 9.21 – 9.07 (m, 1H), 7.68 – 7.47 (m, 3H), 7.46 – 7.36 (m, 2H), 7.22 – 7.12 (m, 1H), 7.06 (d, J = 7.7 Hz, 2H), 6.90 – 6.80 (m, 1H), 3.56 (d, J = 38.9 Hz, 3H), 2.53 (s, 3H); ^{13}C NMR (75 MHz, DMSO-d_6) δ= 162.8, 162.4, 162.2, 157.1, 156.3, 139.4, 132.7, 131.5, 131.4, 130.1 (2C), 128.8, 128.7, 123.7, 118.9 (2C), 115.0, 114.6, 110.1, 39.5, 15.5; FT-IR (neat): ṽ [cm^{-1}]= 3376, 2931, 1687, 1612, 1593, 1532, 1486, 1458, 1423, 1304, 1259, 1213, 1189, 1150, 1121, 970, 909, 873, 774, 742, 722, 688, 673, 657; ESI-MS *m/z*: [M+Na]$^+$ 406.08 (100%), 407.04 (23.79%), 408.04 (6.54%); purity (HPLC)= 99.1%.

5-Amino-*N*-(4-benzoylphenyl)-2-(methylsulfonyl)pyrimidine-4-carboxamide (7e)

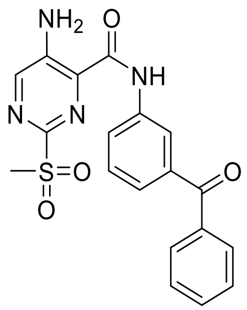

According to the general procedure (II), the title compound **7e** was obtained from **3z** as a beige solid (12 mg, 23%); mp: 280 °C; ^{1}H NMR (300 MHz, DMSO-d_6) δ= 9.73 (s, 1H), 7.78 (s, 1H), 7.47 – 7.36 (m, 1H), 7.29 – 7.16 (m, 1H), 6.97 – 6.90 (m, 2H), 6.89 – 6.82 (m, 1H), 6.80 – 6.63 (m, 6H), 2.64 (s, 3H); ^{13}C NMR (75 MHz, DMSO-d_6) δ= 195.5, 164.1, 150.7, 149.0, 144.4, 137.7, 137.5, 136.9, 132.8, 130.8, 129.6 (2C), 128.9, 128.6 (2C), 125.7, 125.2, 121.9, 39.6; FT-IR (neat): ṽ [cm^{-1}]= 3414, 3307, 1678, 1650, 1605, 1528, 1481, 1427, 1318, 1294, 1213, 1122, 982, 972, 890, 833, 807, 789, 775, 717, 687, 674, 660; ESI-MS *m/z*: [M+Na]$^+$ 419.08 (100%), 420.08 (23.26%), 421.11 (6.31%); purity (HPLC)= 99.5%.

5 Cysteine-reactive probe

4-Methyl-2-oxo-2*H*-chromen-7-yl acrylate (8)

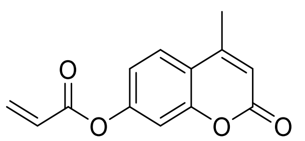

The title compound **8** was synthesized by a procedure according to Zeng et al. To a solution of 4-methylumbelliferone (88 mg, 0.50 mmol) in dichloromethane (10 mL), acryloyl chloride (72 µL, 0.90 mmol) and DIPEA (87 µL, 0.50 mmol) were added dropwise. The reaction mixture was stirred for 16 h at room temperature and extracted with $NaHCO_3$ sat. and HCl (1 M). Purification by silica column chromatography (CH/EA 5:1) afforded the title compound **8** as a colourless solid (20 mg, 17%); mp: 159 °C; ^{1}H NMR (300 MHz, DMSO-d_6) δ= 7.83 (d, J = 8.6 Hz, 1H), 7.35 (d, J = 2.2 Hz, 1H), 7.24 (dt, J = 8.6, 2.2 Hz, 1H), 6.59 (dd, J = 17.2, 1.6 Hz, 1H), 6.51 – 6.35 (m, 2H), 6.20 (dd, J = 10.1, 1.6 Hz, 1H), 2.45 (s, 3H); ^{13}C NMR (75 MHz, DMSO-d_6) δ= 163.6, 159.6, 153.5, 152.9, 152.6, 134.3, 127.3, 126.4, 118.3, 117.6, 113.8, 110.1, 18.1; FT-IR (neat): ṽ [cm^{-1}]= 1730, 1614, 1386, 1371, 1292, 1263, 1159, 1074, 1066, 1019, 985, 970, 903, 875, 845, 824, 803, 794, 750, 706, 671; ESI-MS *m/z*: [M+H]$^+$ 230.81 (100%), 231.35 (10.35%), 232.26 (3.31%); purity (HPLC)= 99.9%.

6 Strategy to design the chimeric sulfonylpyrimidine inhibitors 5b,c

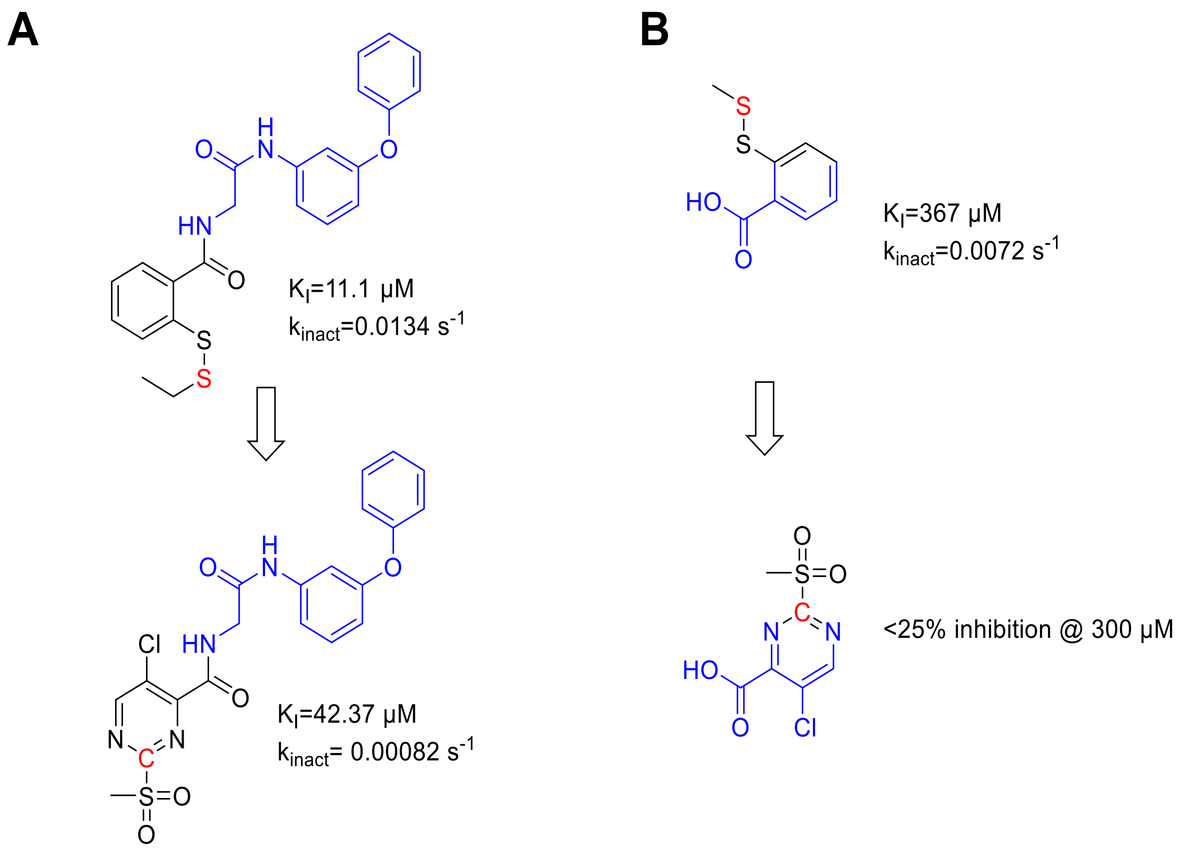

Figure 1: Scheme of the design strategy to derive the chimeric sulfonylpyrimidines 5b,c from previously published SrtA inhibitors (Barthels et al., 2020; doi: 10.1002/cmdc.201900687). (A) The previously published disulfanylbenzamide (original manuscript compound numbering: **7a**) was used to design the chimeric inhibitor **5b**. The electrophilic center of the warhead chemotype is highlighted in red. The preserved sequence of the recognition motif (3-PhOPh-NH-Glycine) is marked in blue. (**B**) The previously published disulfide-substituted benzoic acid (original manuscript compound numbering: **3a**) was used to design the chimeric fragment inhibitor **5c**. The electrophilic center of the warhead chemotype is highlighted in red. The preserved sequence of the recognition motif (aromatic carboxylic acid) is marked in blue.

7 Mass spectrometry data supporting the cysteine reactivity experiments

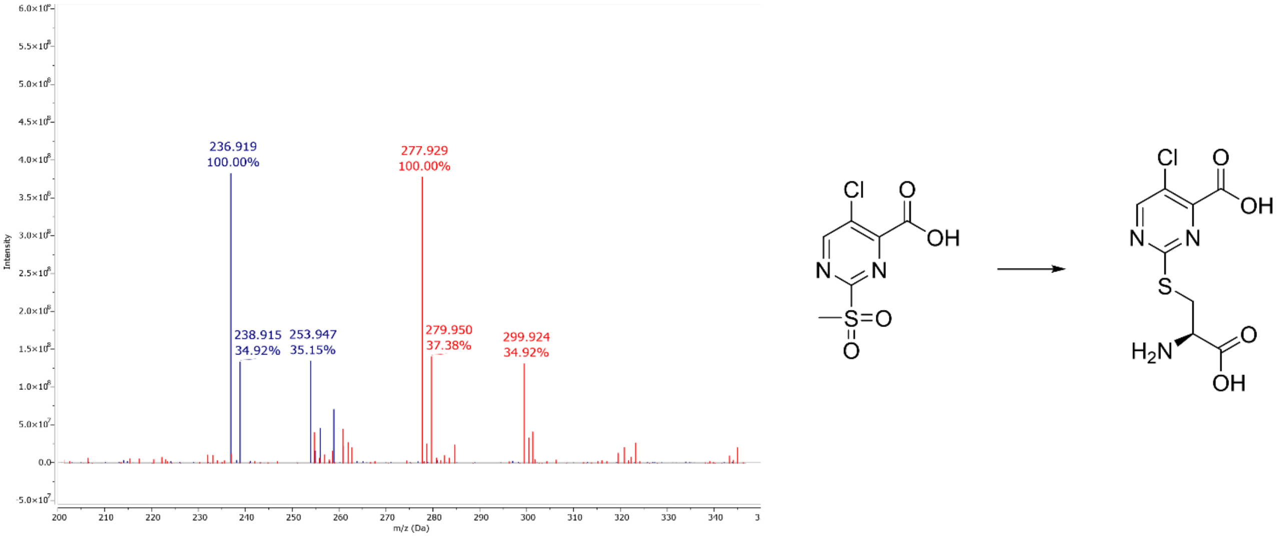

Figure 2: ESI-MS of compound 5c (blue, m/z=236.91) treated with free cysteine revealed the formation of a distinct adduct (red, m/z=277.92) corresponding to the respective 2-pyrimidyl cysteine.

8 Quantum mechanical calculations

To investigate the reaction in solution, the model reaction of inhibitor **5c** with methanethiol was chosen. The reaction coordinate is labeled with $R(S_{thiol}\text{-}C_{inhibitor})$ and describes the distance between the thiol(ate) sulfur atom and the C-2 atom of the inhibitor (Figure 3).

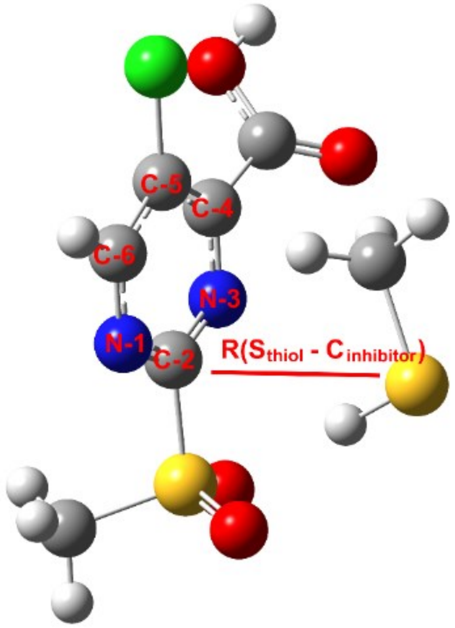

Figure 3: Selected model for QM calculations between inhibitor 5c and methanethiol.

Computed reaction paths of inhibitor **5c** with methanethiol or methanethiolate are shown in Figure 4.

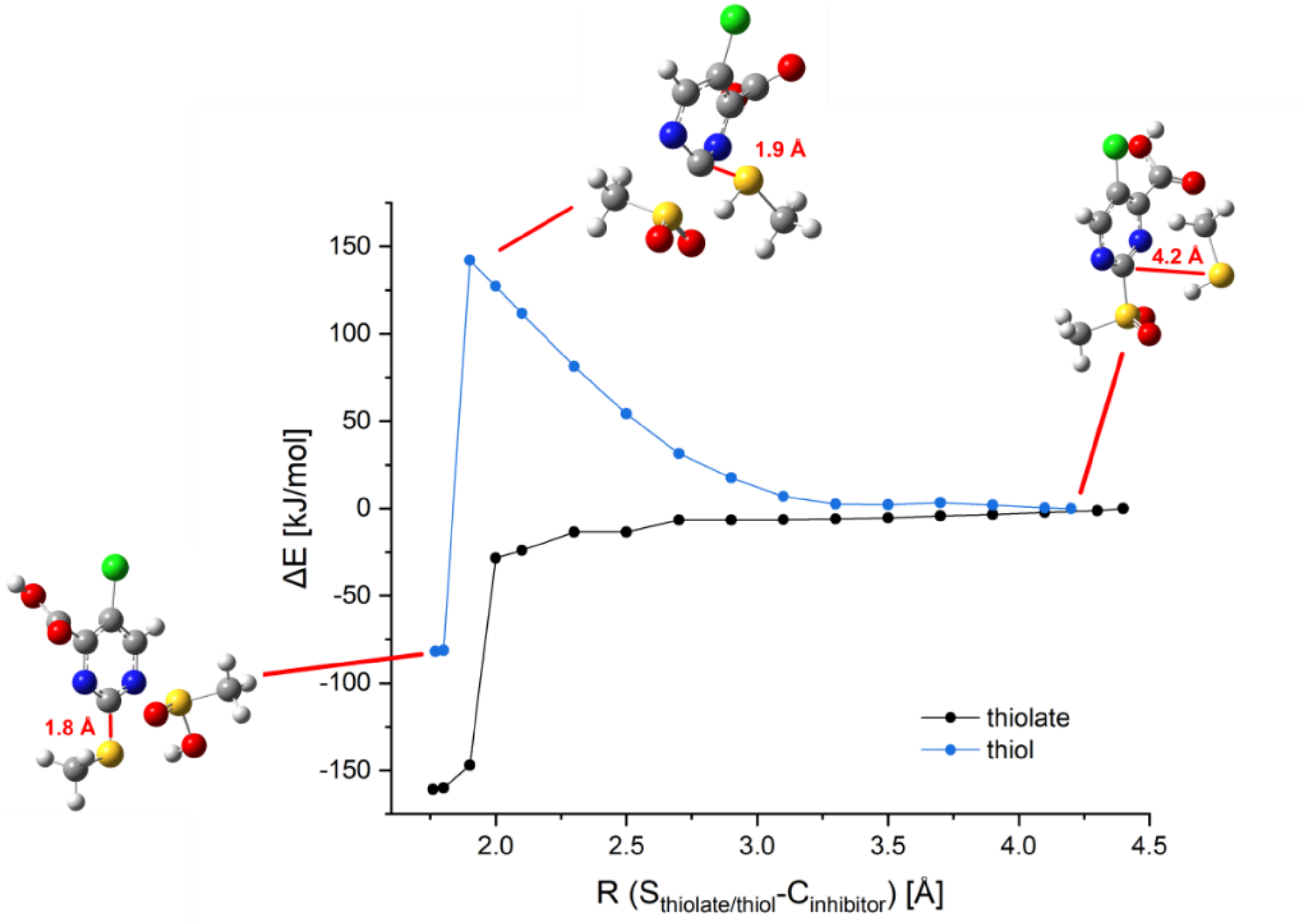

Figure 4: Computed reaction paths with thiolate and thiol: Energy profile of the calculated model reaction of inhibitor **5c** with methanethiolate or methanethiol.

Calculated reaction paths of inhibitor **5c** with methanethiol in the presence of all possible bicarbonate and phosphate species are shown in Figure 5 and Figure 6.

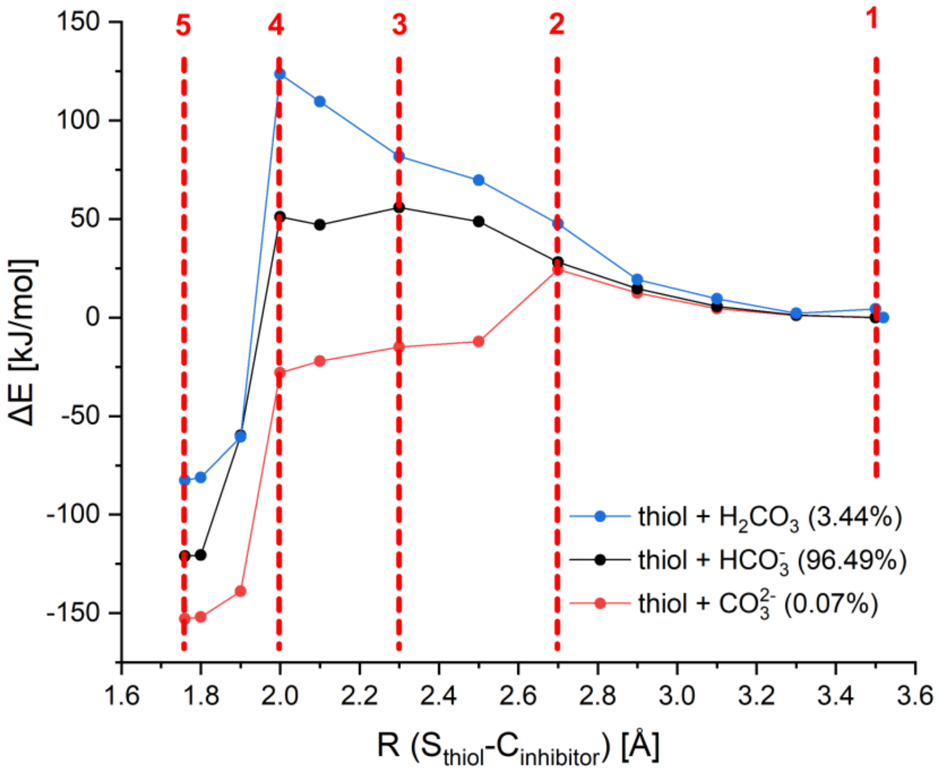

Figure 5: Computed reaction paths mediated by bicarbonate buffer: Energy profile of the calculated model reaction between inhibitor **5c** and methanethiol in the presence of bicarbonate buffer species, the dashed red lines indicated the structures depicted in the following Table 1.

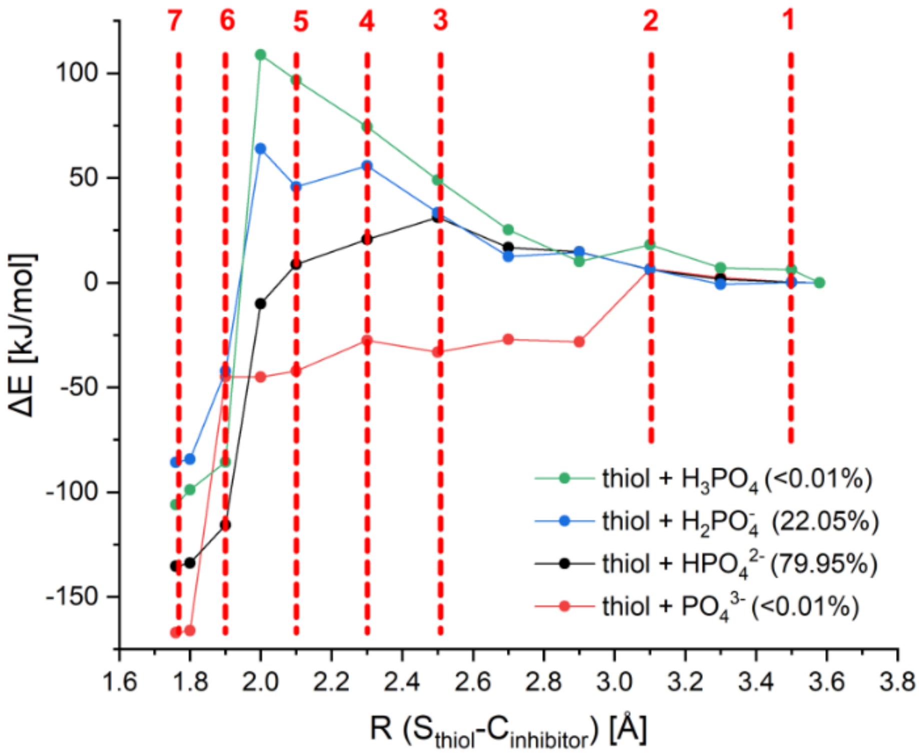

Figure 6: Computed reaction paths mediated by phosphate buffer: Energy profile of the calculated model reaction between inhibitor **5c** and methanethiol in the presence of phosphate buffer species, the dashed red lines indicated the structures depicted in the following Table 2.

Table 1: Selected structures from the calculated reaction paths of inhibitor **5c** and all bicarbonate buffer species.

	**R (S_{thiol}-C_{inh}) [Å]**	**thiol + H_2CO_3**	**thiol + HCO_3^-**	**thiol + CO_3^{2-}**
**1**	**3.5**	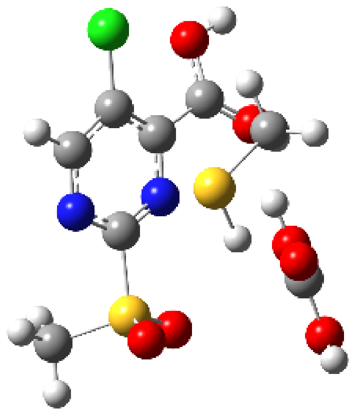	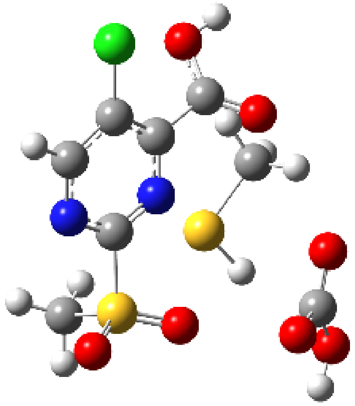	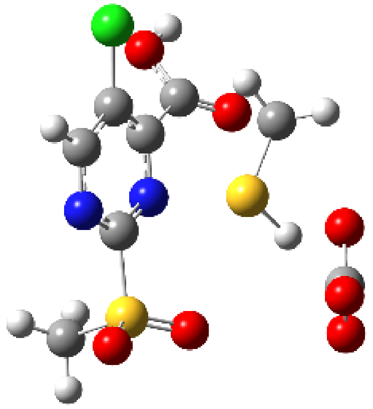
**2**	**2.7**	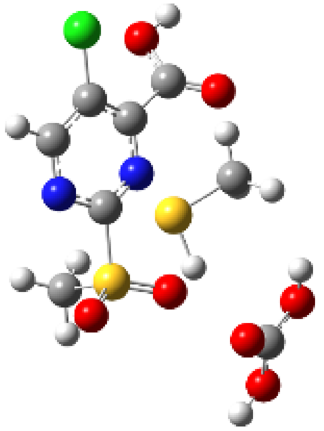	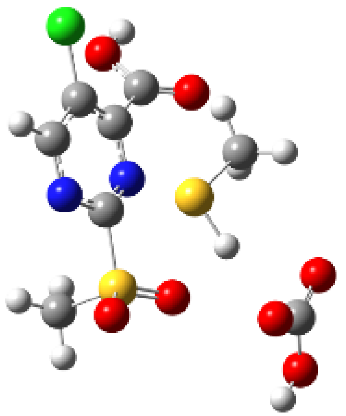	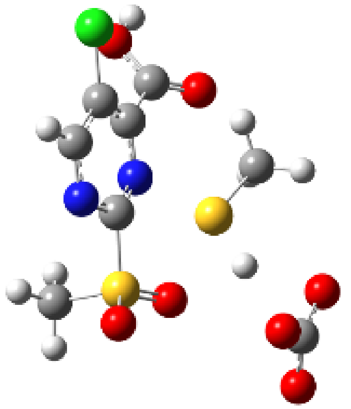

**3**	**2.3**	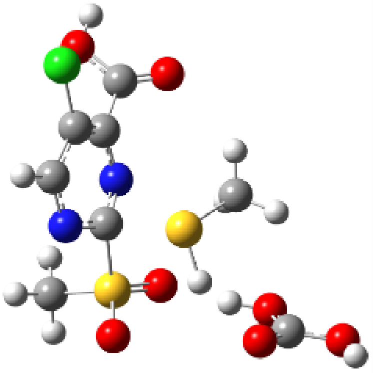	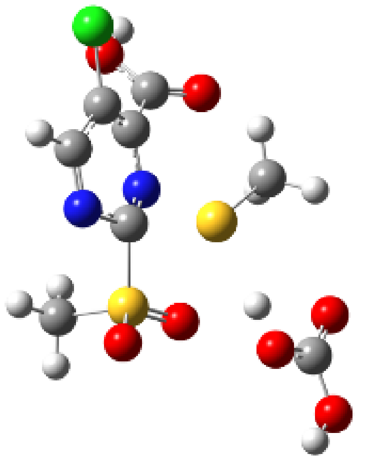	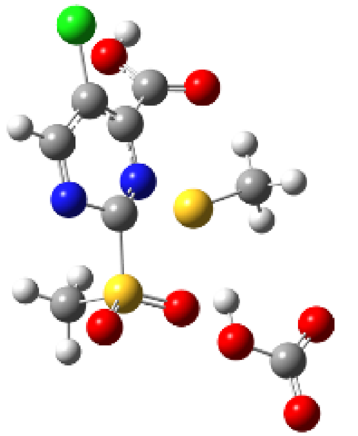
**4**	**2.0**	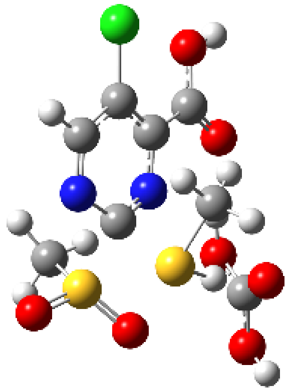	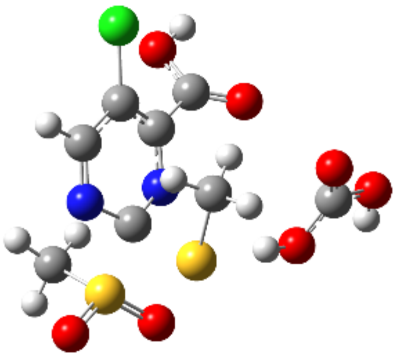	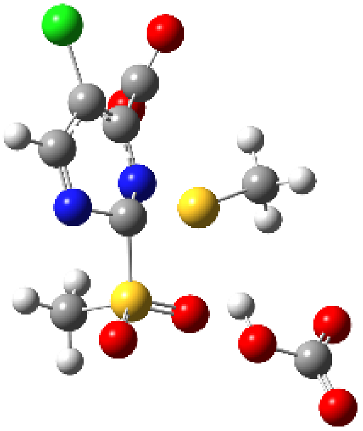

5	1.76	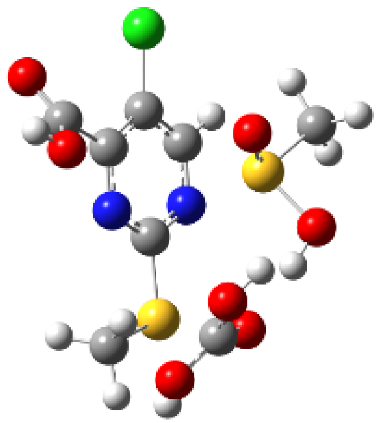	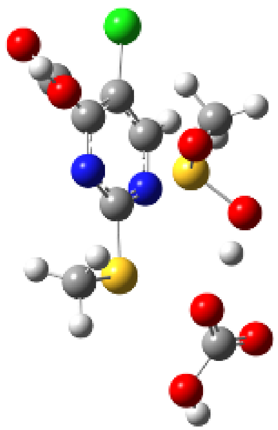	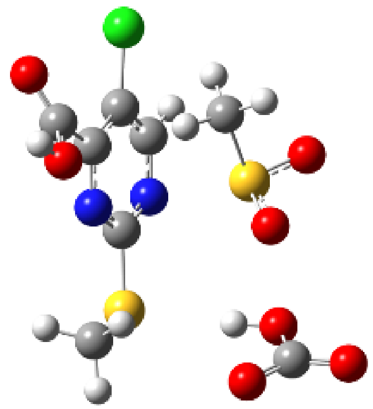

Table 2: Selected structures from the calculated reaction paths of inhibitor **5c** and all phosphate buffer species.

	R (S_{thiol}-C_{inh}) [Å]	thiol + H_3PO_4	thiol + $H_2PO_4^-$	thiol + HPO_4^{2-}	thiol + PO_4^{3-}
1	3.5	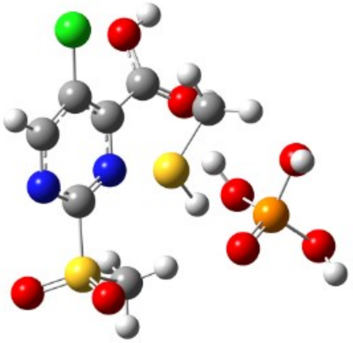	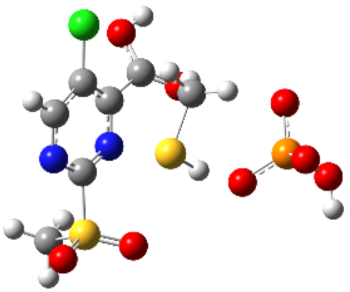	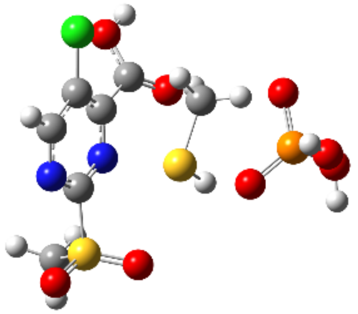	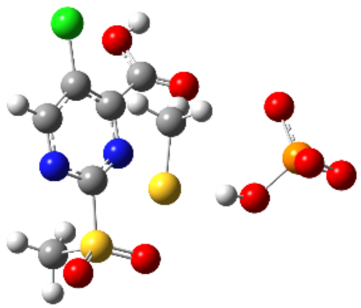

**2**	**2.9**	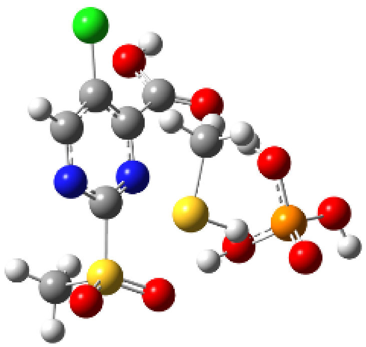	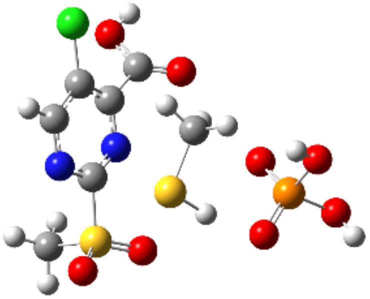	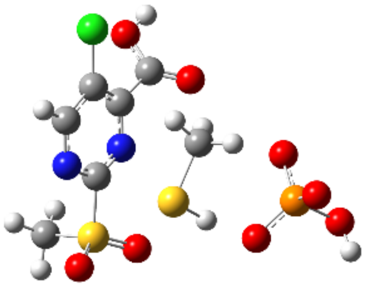	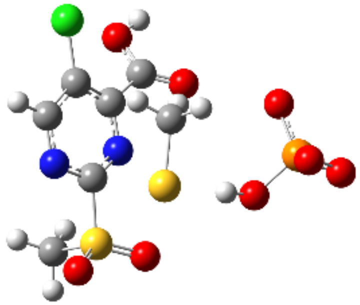
**3**	**2.5**	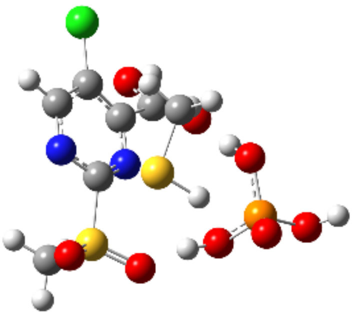	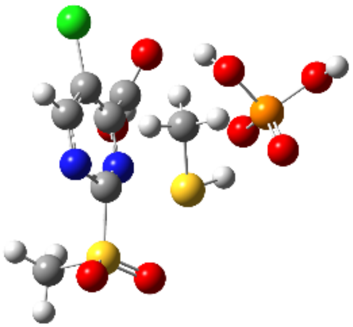	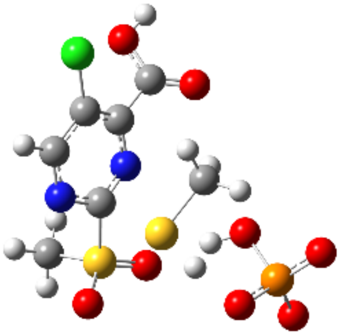	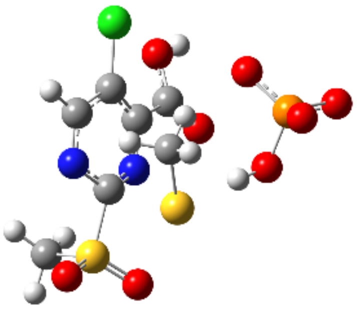
**4**	**2.3**	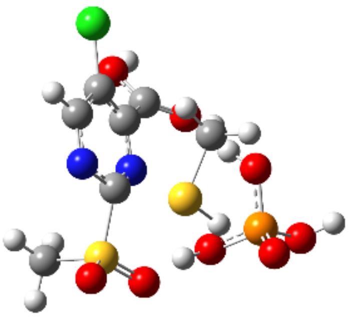	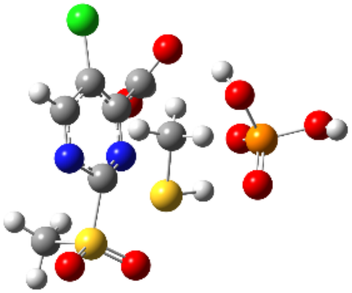	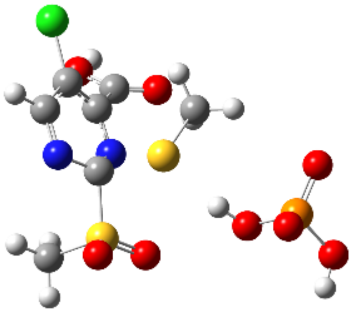	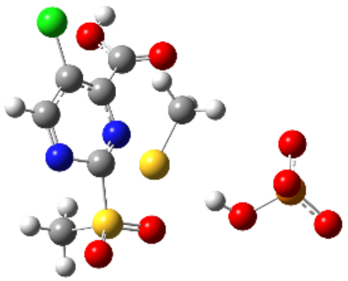

**5**	**2.1**	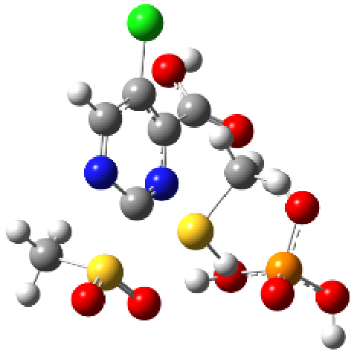	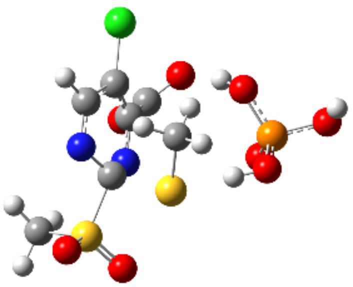	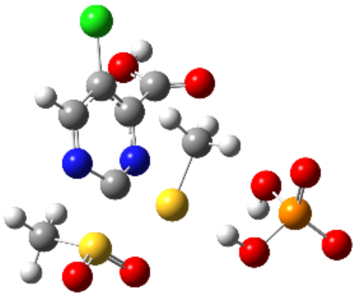	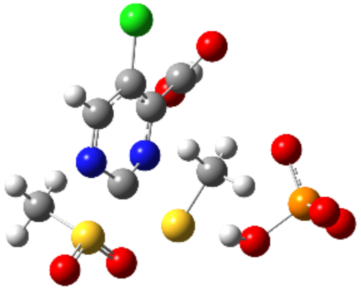
**6**	**1.9**	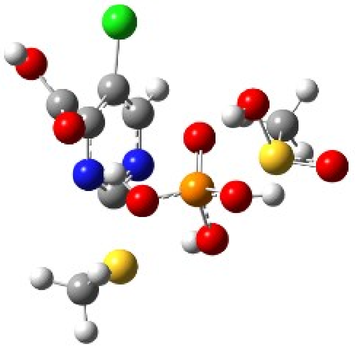	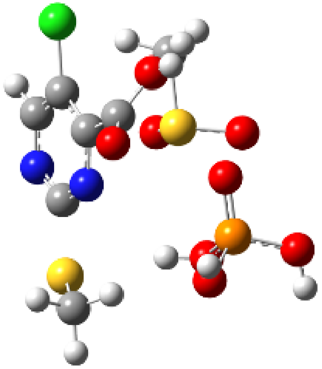	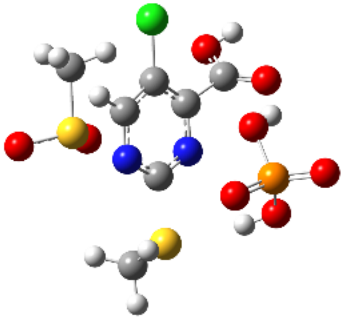	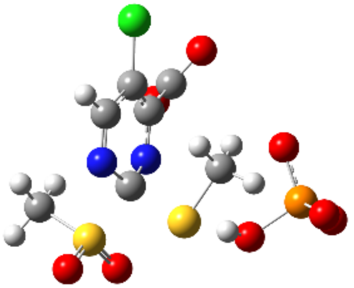
**7**	**1.76**	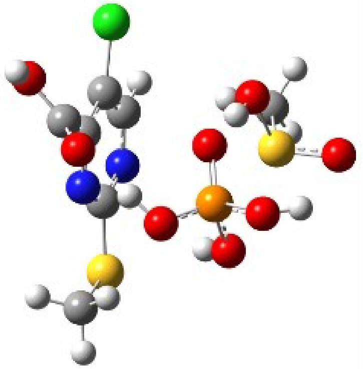	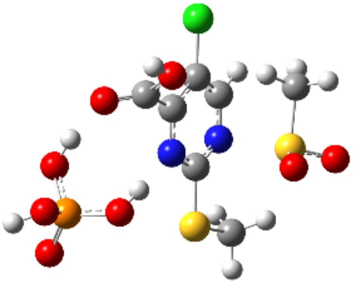	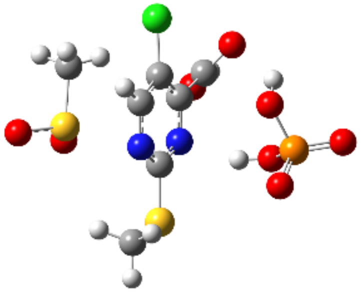	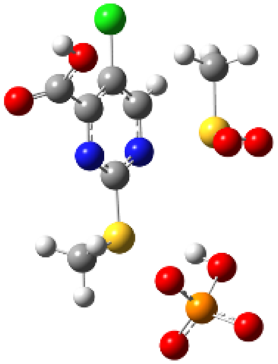

The reaction path of inhibitor **5c** with methanethiol in the presence of 4-methylimidazole is shown in Figure 7.

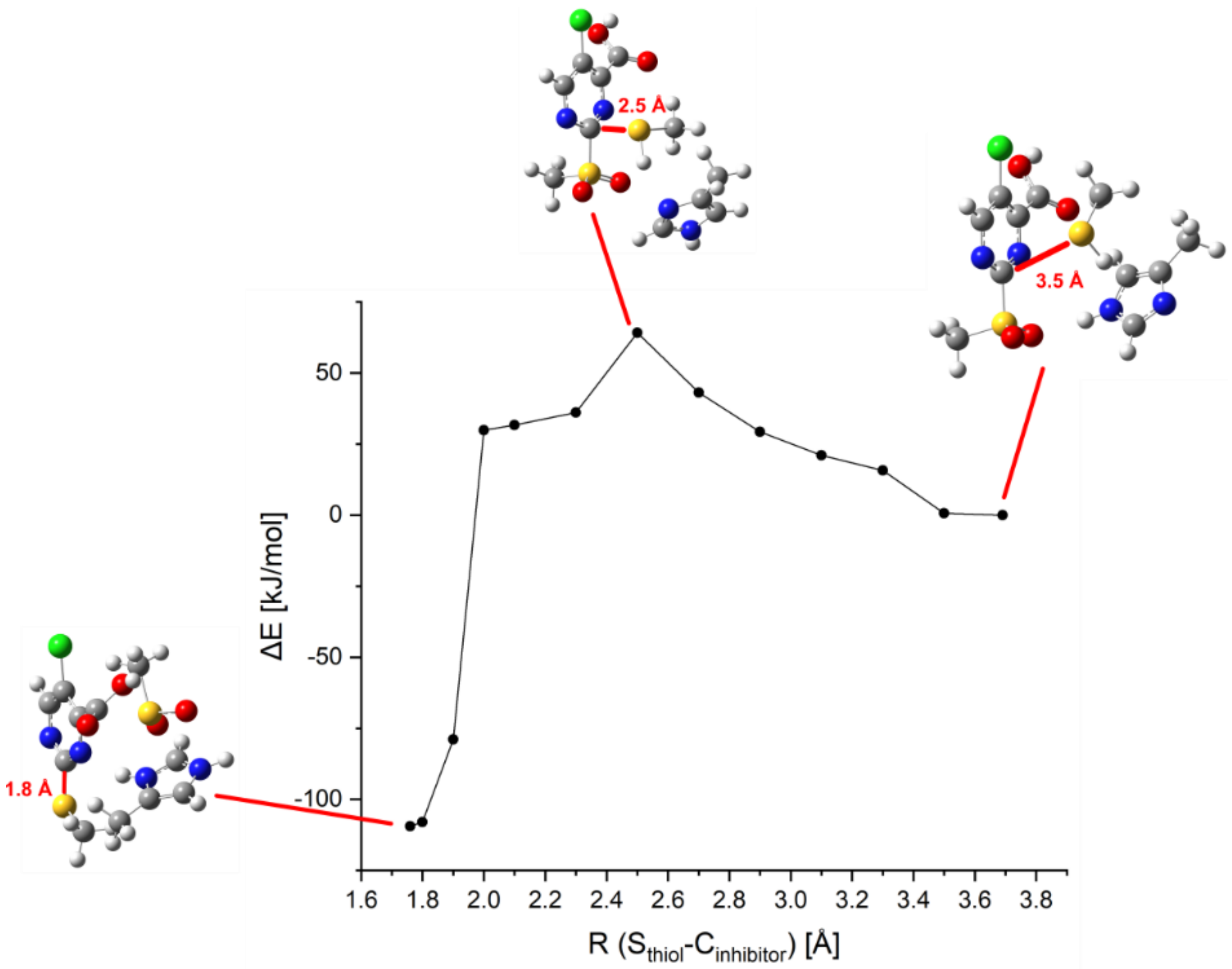

Figure 7: Computed reaction paths mediated by 4-methylimidazole: Energy profile of the calculated model reaction between inhibitor **5c** and methanethiol in the presence of 4-methylimidazole.

In the following Table 3 an overview of all computed barrier heights, product energies, and distances R(S_{thiol}-C-$2_{inhibitor}$), at which the proton is transferred, is shown.

Table 3: Computed model reactions of inhibitor 5c: Reaction barrier, product energy, and distances R(S_{thiol}-C-$2_{inhibitor}$), at which the proton is transferred, of the calculated model reaction between inhibitor **5c** and methanethiol(ate).

	**thiolate**	**thiol**	**thiol + HCO_3^-**	**thiol + H_2CO_3**	**thiol + CO_3^{2-}**	**thiol + HPO_4^{2-}**	**thiol + $H_2PO_4^-$**	**thiol + H_3PO_4**	**thiol + PO_4^{3-}**	**thiol + 4-Me-imidazol**
barrier [kJ/mol]	–	142	56	123	24	31	64	109	7	64
product [kJ/mol]	–161	–82	–121	–82	–153	–135	–86	–106	–167	–110
proton transfer (R_{S-C-2}) [Å]	–	1.8	2.3	1.9	2.5	2.3	2.1	1.9	2.9	2.3

9 References

Arukwe, J., Undheim, K., Skjetne, T., and Darzynkiewicz, E. (1986). Lithiation in the Synthesis of 5-Pyrimidinyl Ketones. *Acta Chem. Scand.* 40b, 588–592. doi:10.3891/acta.chem.scand.40b-0588.

Barthels, F., Marincola, G., Marciniak, T., Konhäuser, M., Hammerschmidt, S., Bierlmeier, J., et al. (2020). Asymmetric Disulfanylbenzamides as Irreversible and Selective Inhibitors of Staphylococcus aureus Sortase A. *ChemMedChem* 15, 839–850. doi:10.1002/cmdc.201900687.

Grant, G. A., Seemann, C. V., and Winthrop, S. O. (1956). Basic Esters Of Substituted Pyrimidine-4-Carboxylic Acids. *Can. J. Chem.* 34, 1444–1446. doi:10.1139/v56-185.

Li, W., Li, J., Wu, Y., Wu, J., Hotchandani, R., Cunningham, K., et al. (2009). A Selective Matrix Metalloprotease 12 Inhibitor for Potential Treatment of Chronic Obstructive Pulmonary Disease (COPD): Discovery of (*S*)-2-(8-(Methoxycarbonylamino)dibenzo[*b* , *d*]furan-3-sulfonamido)-3-methylbutanoic acid (MMP408). *J. Med. Chem.* 52, 1799–1802. doi:10.1021/jm900093d.

Tröster, A., Bauer, A., Jandl, C., and Bach, T. (2019). Enantioselective Visible-Light-Mediated Formation of 3-Cyclopropylquinolones by Triplet-Sensitized Deracemization. *Angew. Chem. Int. Ed.* 58, 3538–3541. doi:10.1002/anie.201814193.

Webb, K. S. (1994). A mild, inexpensive and practical oxidation of sulfides. *Tetrahedron Lett.* 35, 3457–3460. doi:10.1016/S0040-4039(00)73209-6.

Zeng, R.-F., Lan, J.-S., Li, X.-D., Liang, H.-F., Liao, Y., Lu, Y.-J., et al. (2017). A Fluorescent Coumarin-Based Probe for the Fast Detection of Cysteine with Live Cell Application. *Molecules* 22, 1618. doi:10.3390/molecules22101618.

4

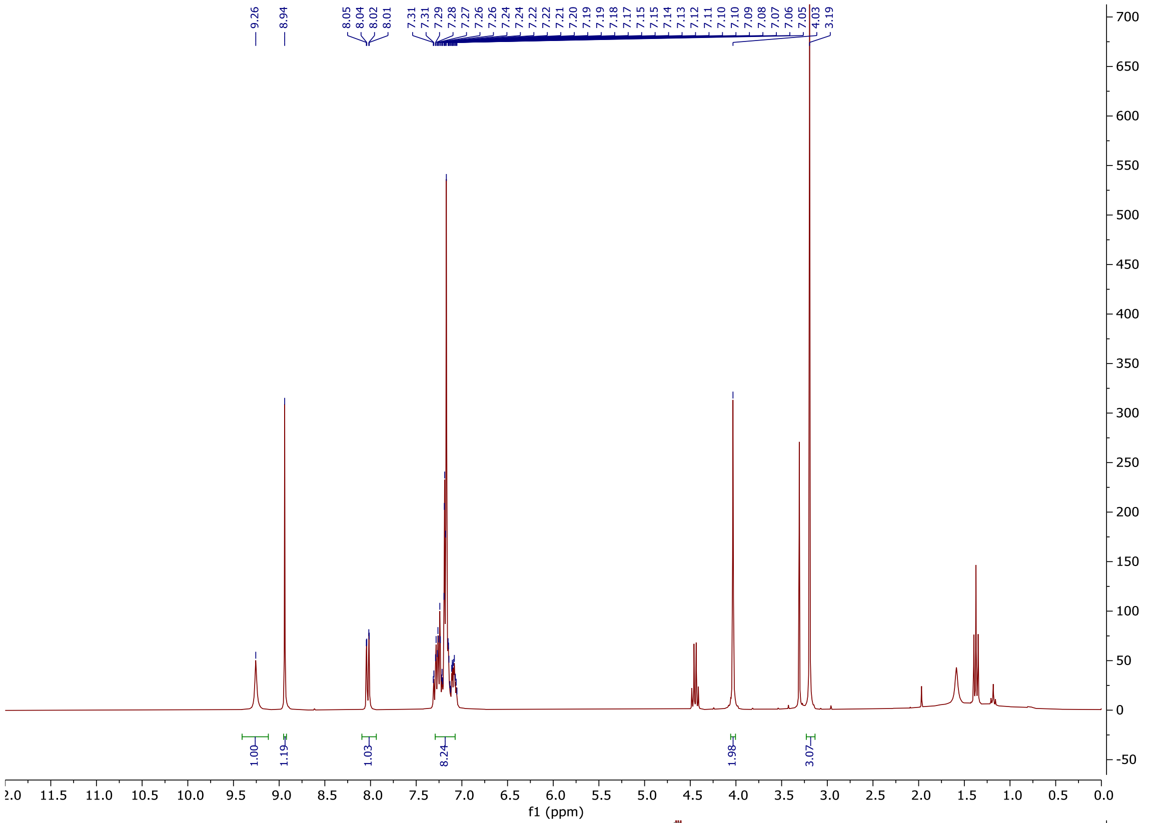
9.26
8.94
8.05
8.04
8.02
8.01
7.31
7.31
7.29
7.28
7.27
7.26
7.26
7.24
7.24
7.22
7.22
7.21
7.20
7.19
7.19
7.18
7.17
7.15
7.15
7.14
7.13
7.12
7.11
7.10
7.10
7.09
7.08
7.07
7.06
7.05
4.03
3.19
1.00
1.19
1.03
8.24
1.98
3.07
f1 (ppm)

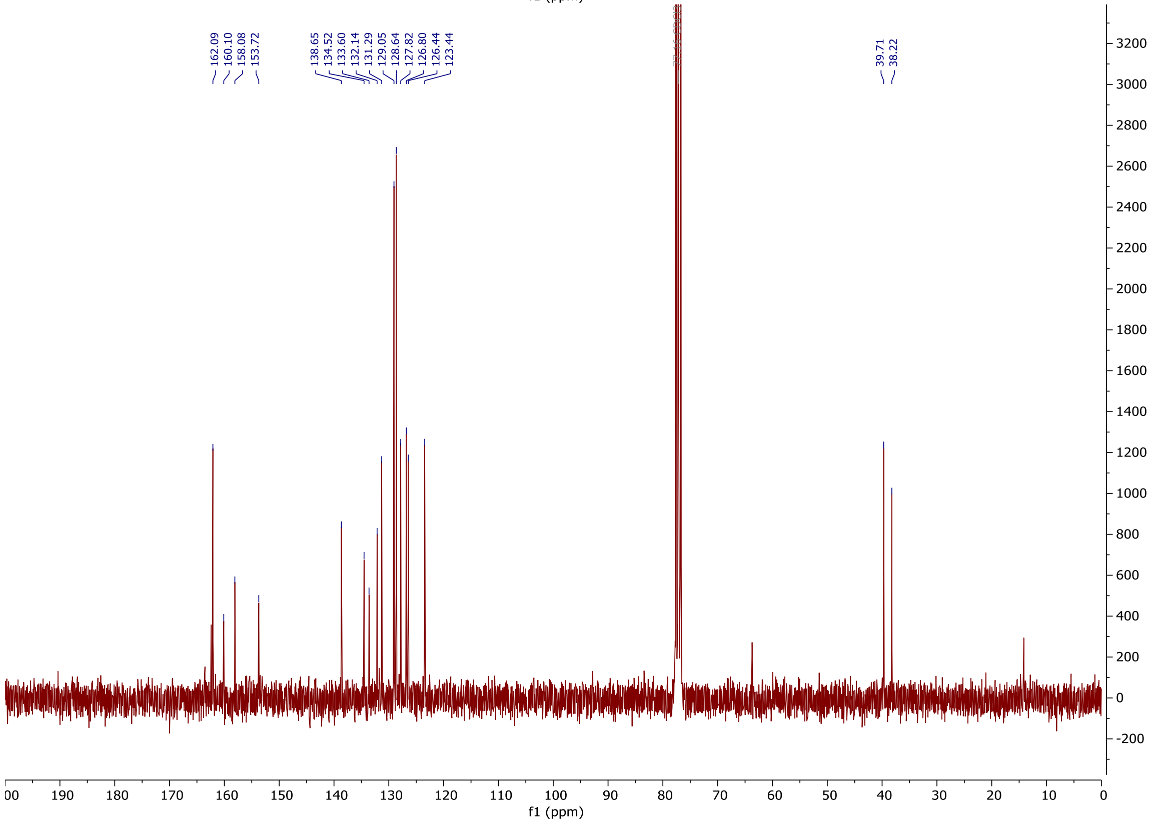
162.09
160.10
158.08
153.72
138.65
134.52
133.60
132.14
131.29
129.05
128.64
127.82
126.80
126.44
123.44
39.71
38.22
f1 (ppm)

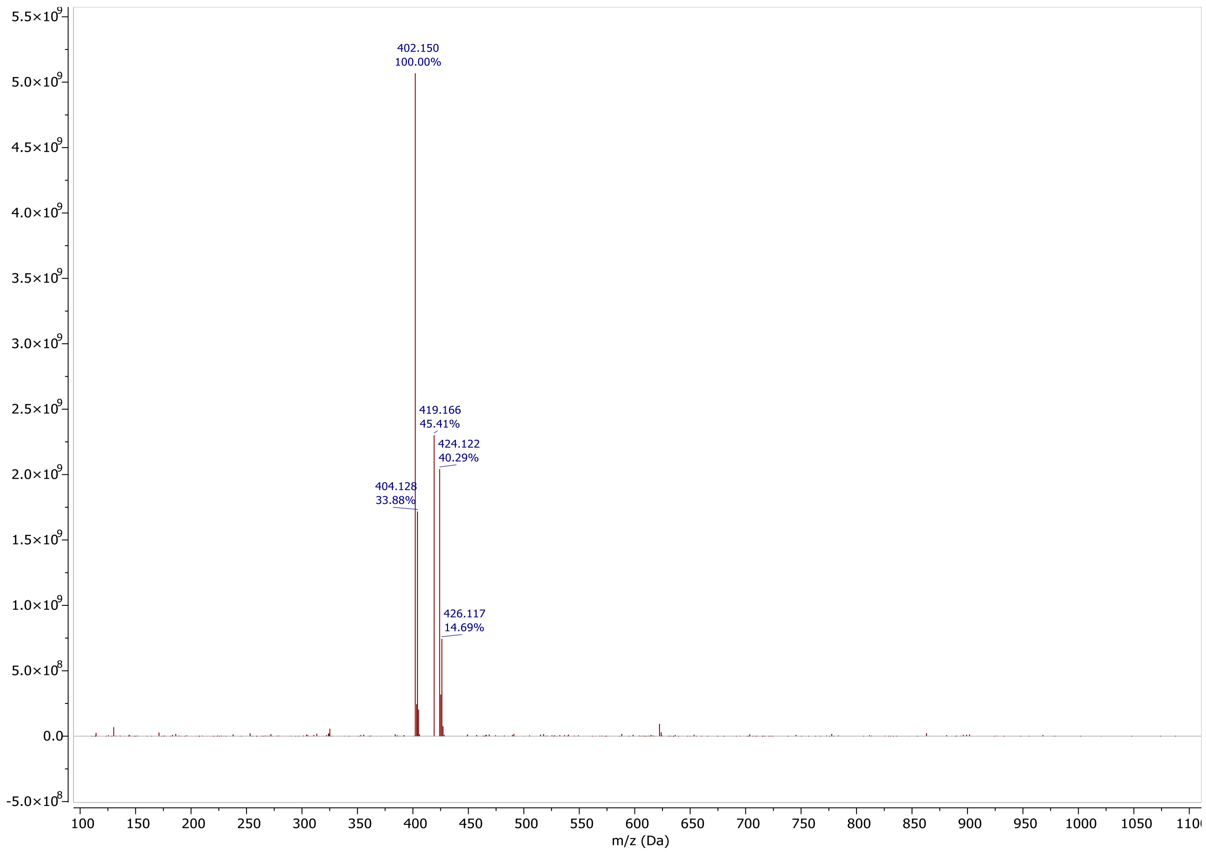
402.150
100.00%
419.166
45.41%
424.122
40.29%
404.128
33.88%
426.117
14.69%
m/z (Da)

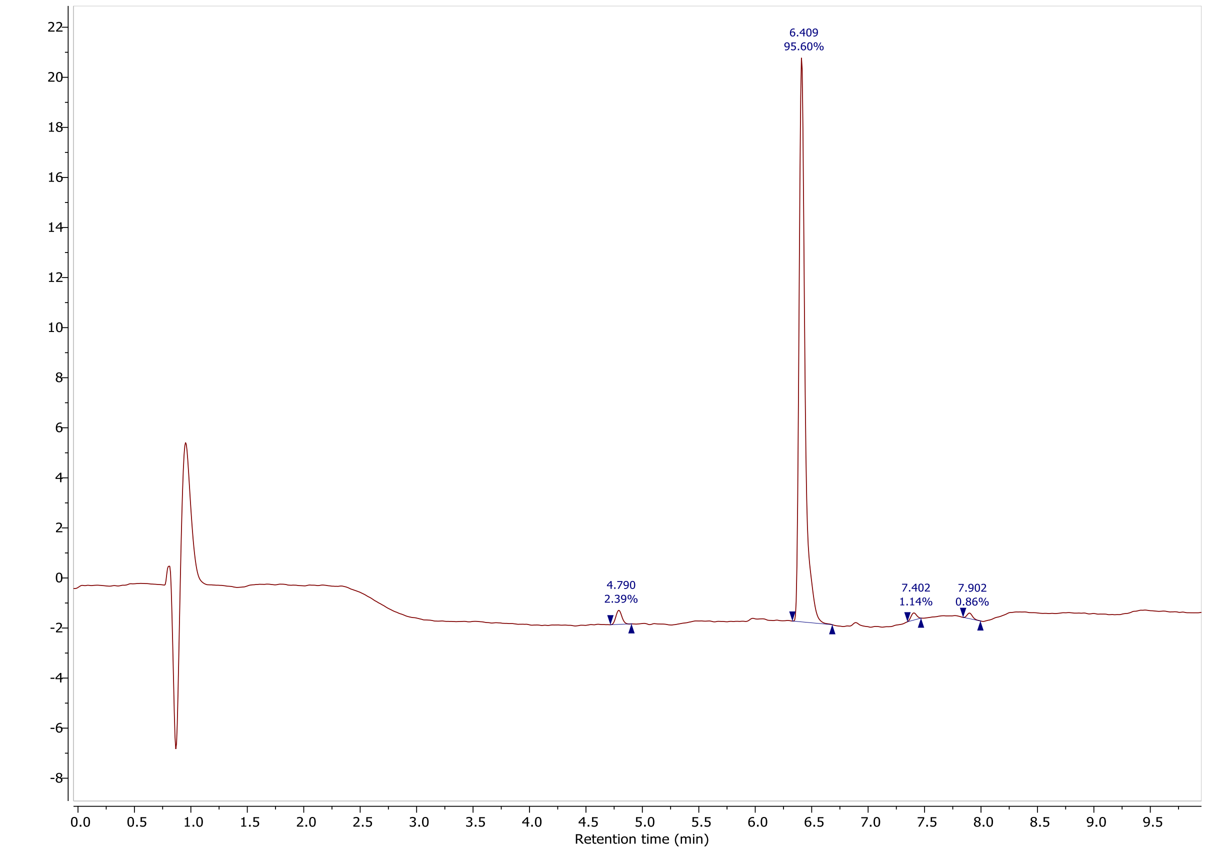
6.409
95.60%
4.790
2.39%
7.402
1.14%
7.902
0.86%
Retention time (min)

5a

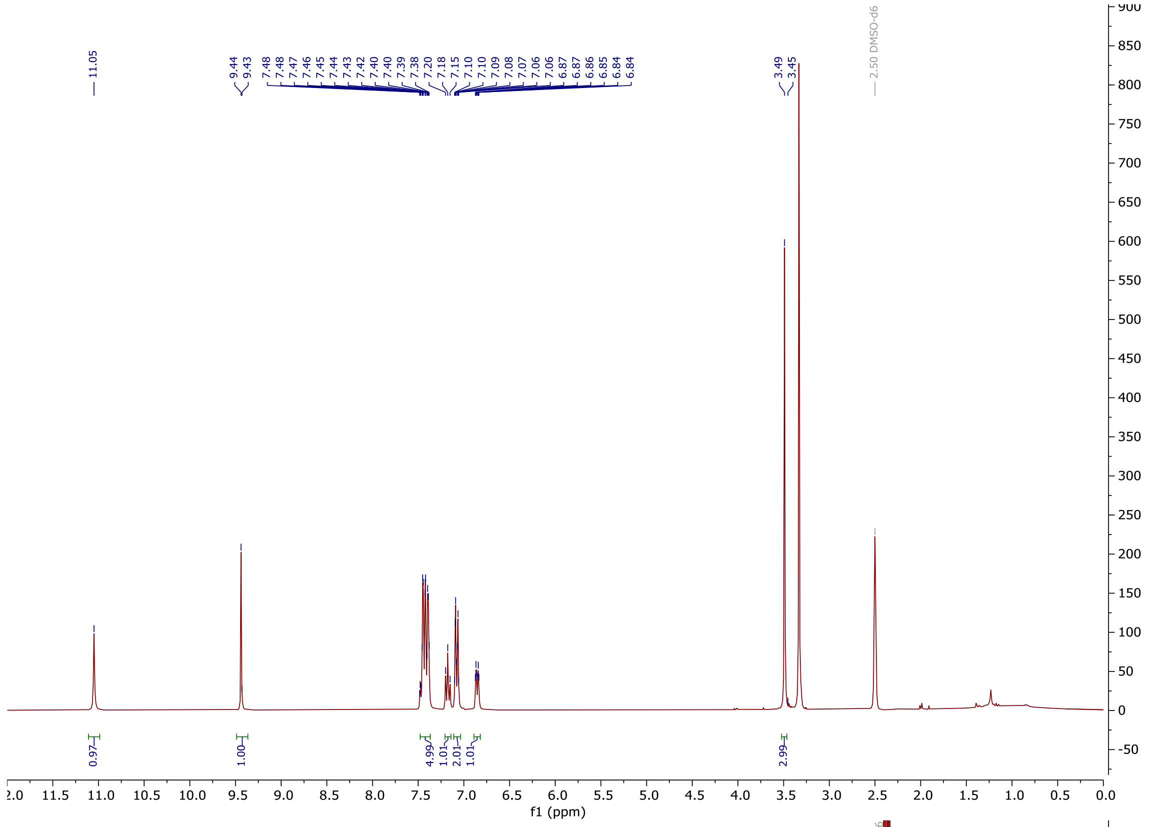
11.05
9.44
9.43
7.48
7.48
7.47
7.46
7.45
7.44
7.43
7.42
7.40
7.40
7.39
7.38
7.20
7.18
7.15
7.10
7.10
7.09
7.08
7.07
7.06
7.06
6.87
6.87
6.86
6.85
6.84
6.84
3.49
3.45
2.50 DMSO-d6
0.97
1.00
4.99
1.01
2.01
1.01
2.99
f1 (ppm)

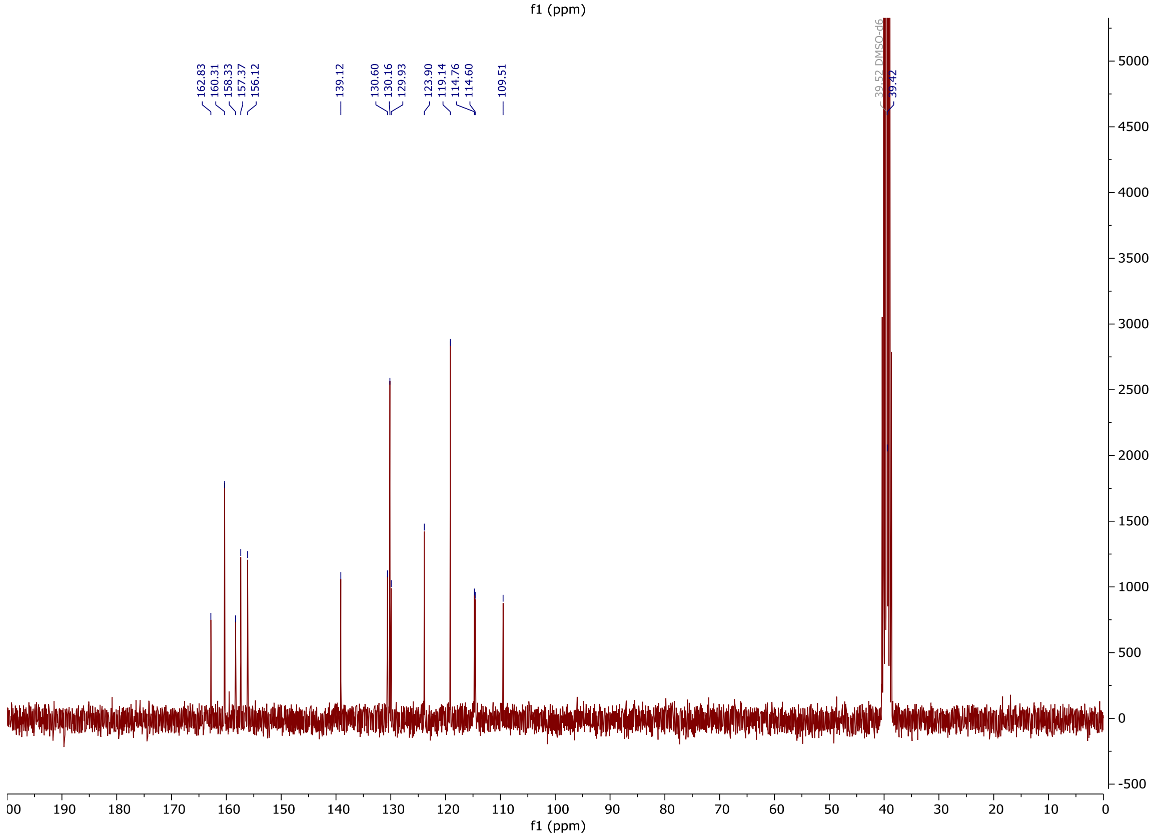
162.83
160.31
158.33
157.37
156.12
139.12
130.60
130.16
129.93
123.90
119.14
114.76
114.60
109.51
39.52 DMSO-d6
39.42
f1 (ppm)

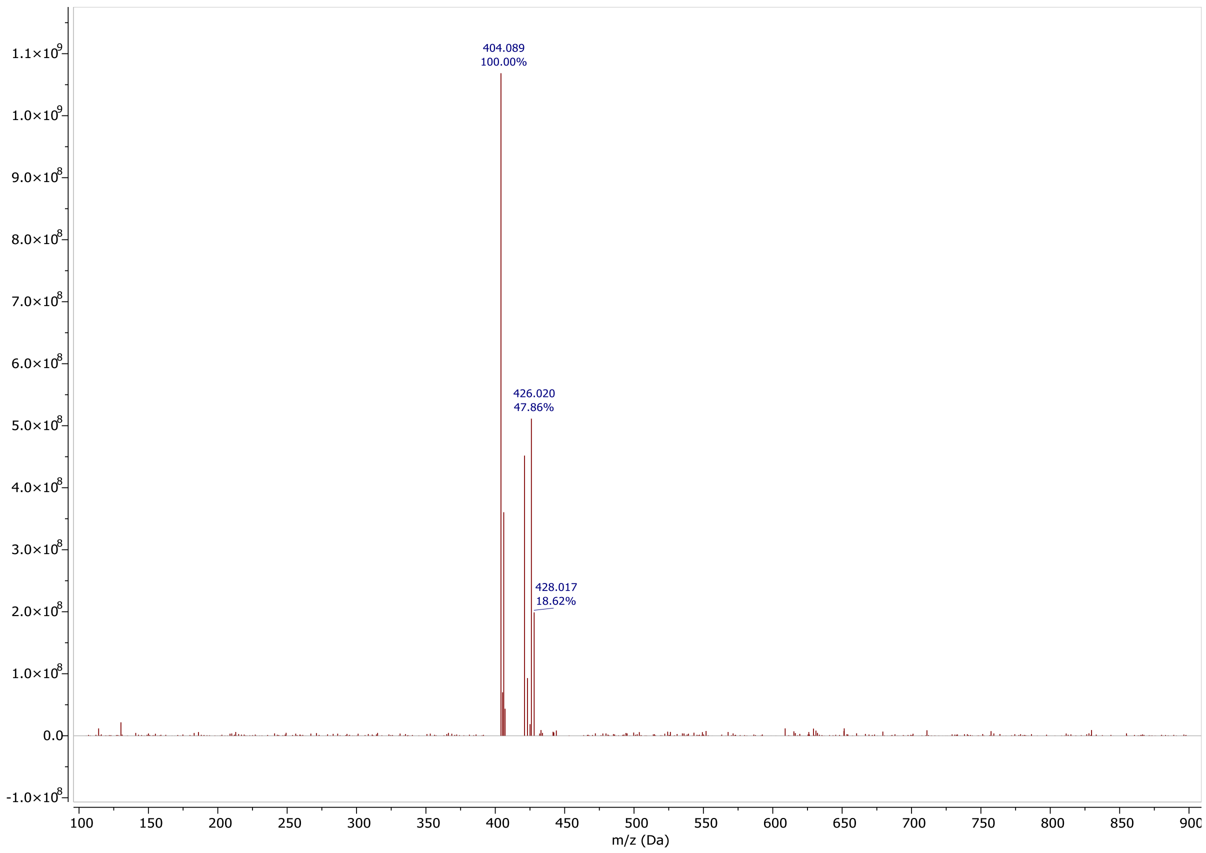
404.089
100.00%
426.020
47.86%
428.017
18.62%
m/z (Da)

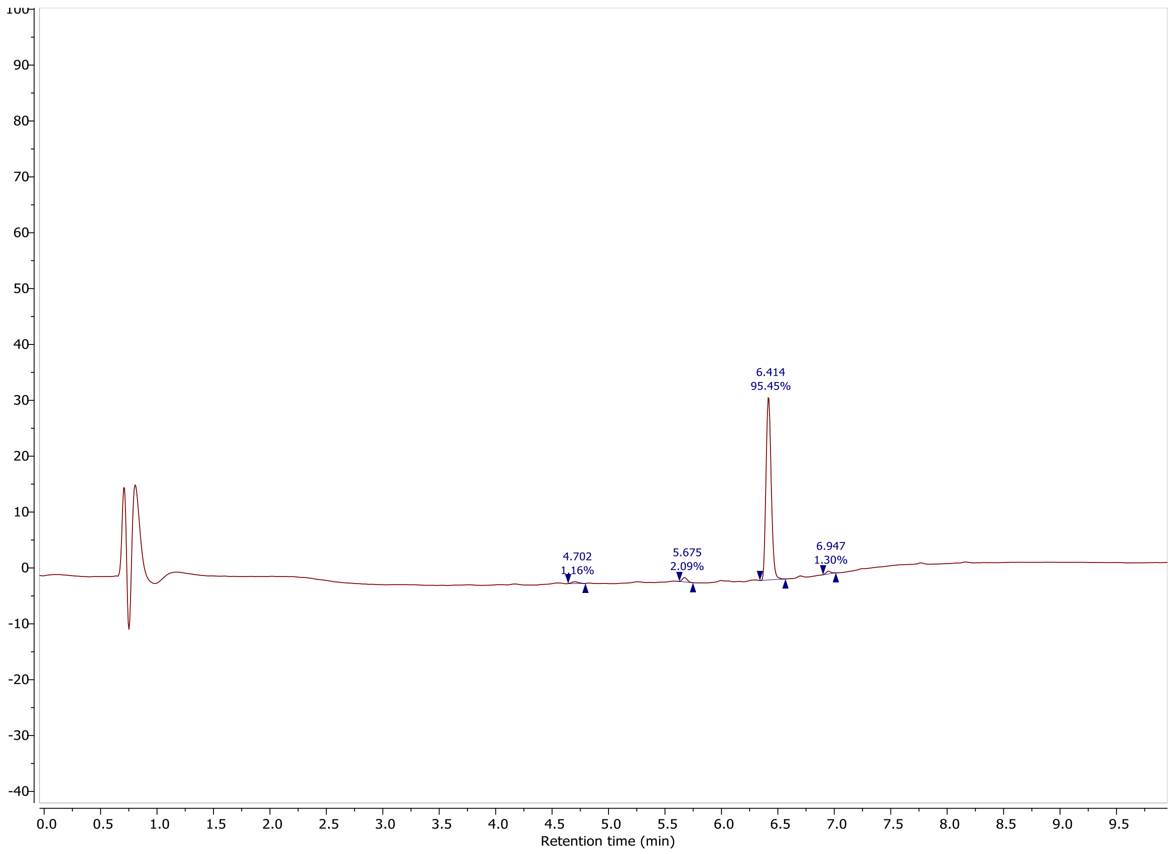
6.414
95.45%
4.702
1.16%
5.675
2.09%
6.947
1.30%
Retention time (min)

5b

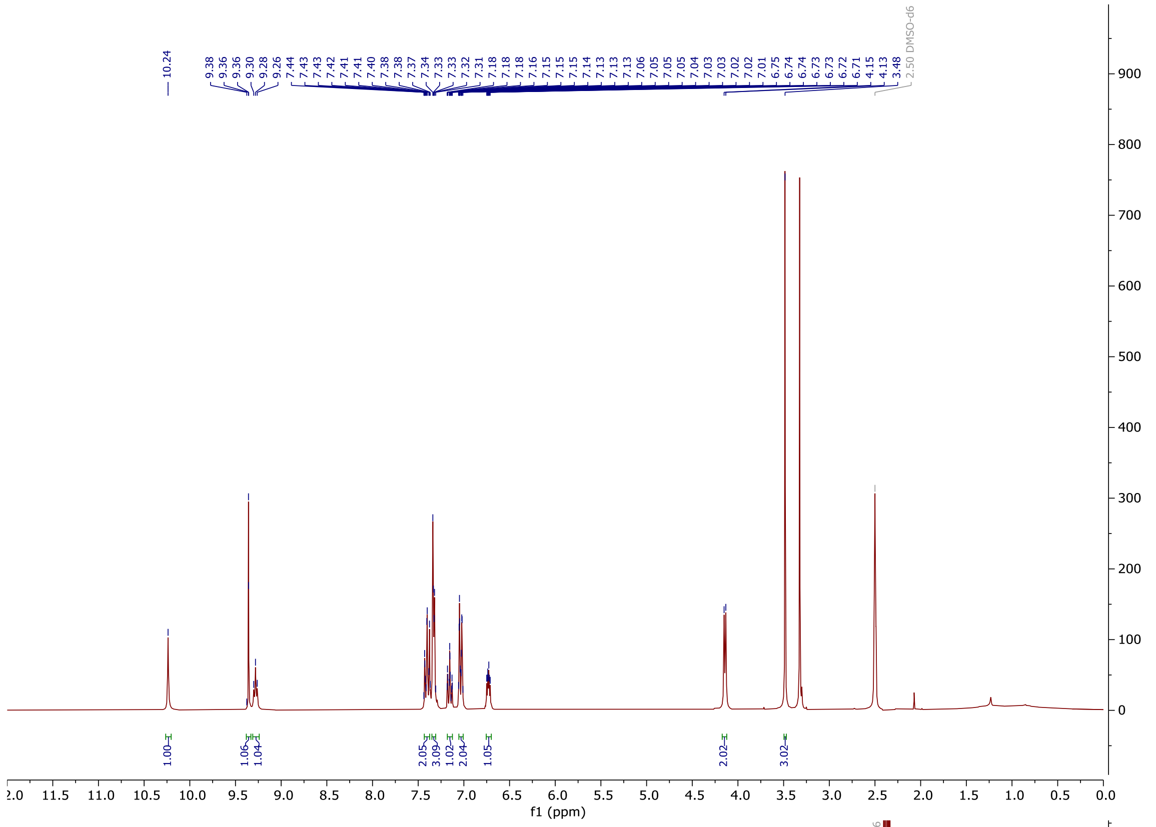
10.24
9.38
9.36
9.36
9.30
9.28
9.26
7.44
7.43
7.43
7.42
7.41
7.41
7.40
7.38
7.38
7.37
7.34
7.33
7.33
7.32
7.31
7.18
7.18
7.18
7.16
7.15
7.15
7.15
7.14
7.13
7.13
7.13
7.06
7.05
7.05
7.05
7.04
7.03
7.03
7.02
7.02
7.01
6.75
6.74
6.74
6.73
6.73
6.72
6.71
4.15
4.13
3.48
2.50 DMSO-d6
1.00
1.06
1.04
2.05
3.09
1.02
2.04
1.05
2.02
3.02
f1 (ppm)

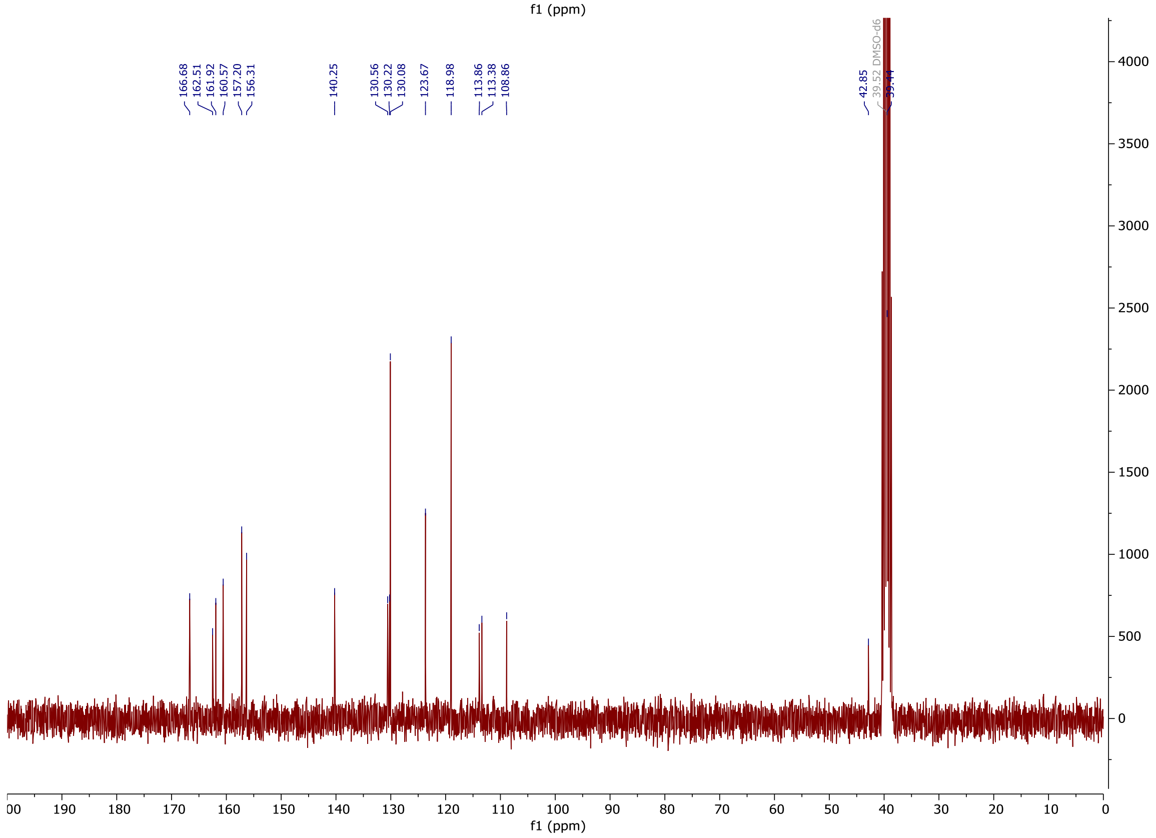
166.68
162.51
161.92
160.57
157.20
156.31
140.25
130.56
130.22
130.08
123.67
118.98
113.86
113.38
108.86
42.85
39.52 DMSO-d6
f1 (ppm)

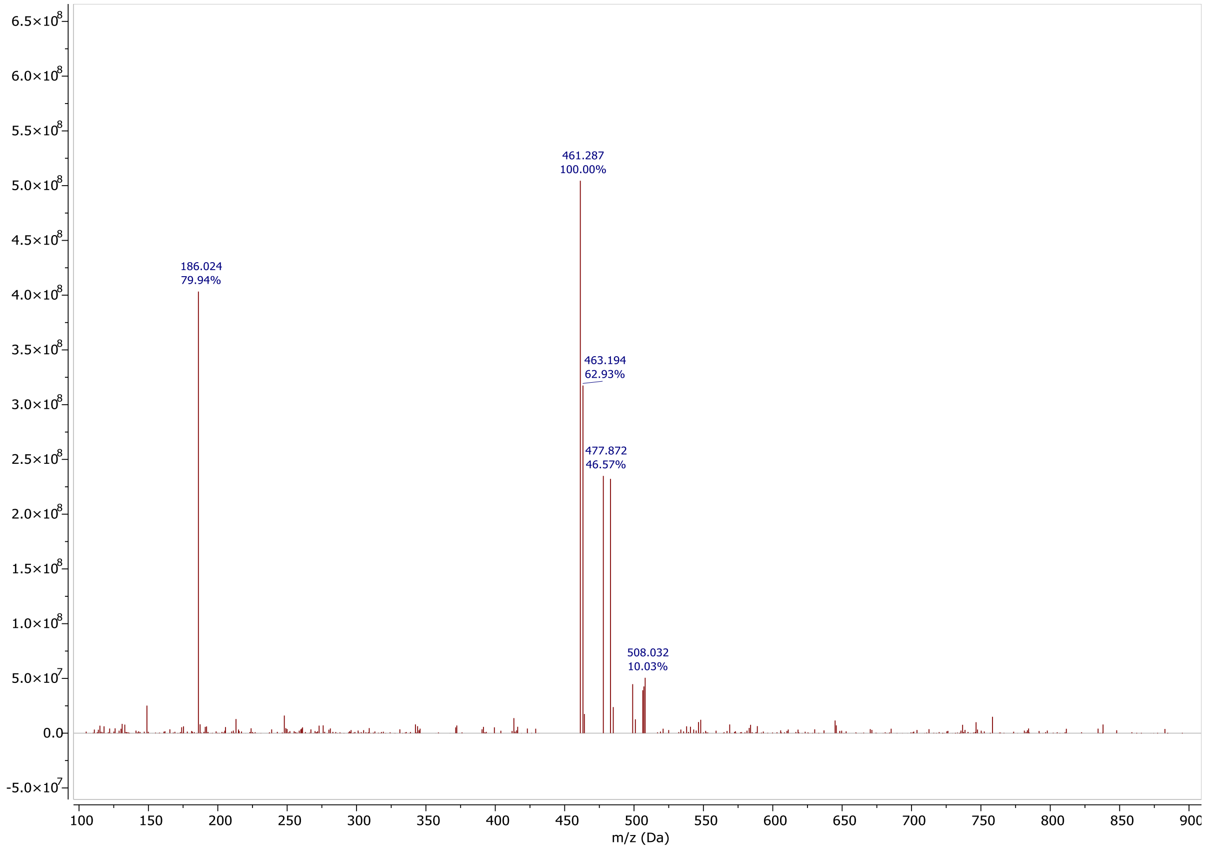
461.287
100.00%
186.024
79.94%
463.194
62.93%
477.872
46.57%
508.032
10.03%
m/z (Da)

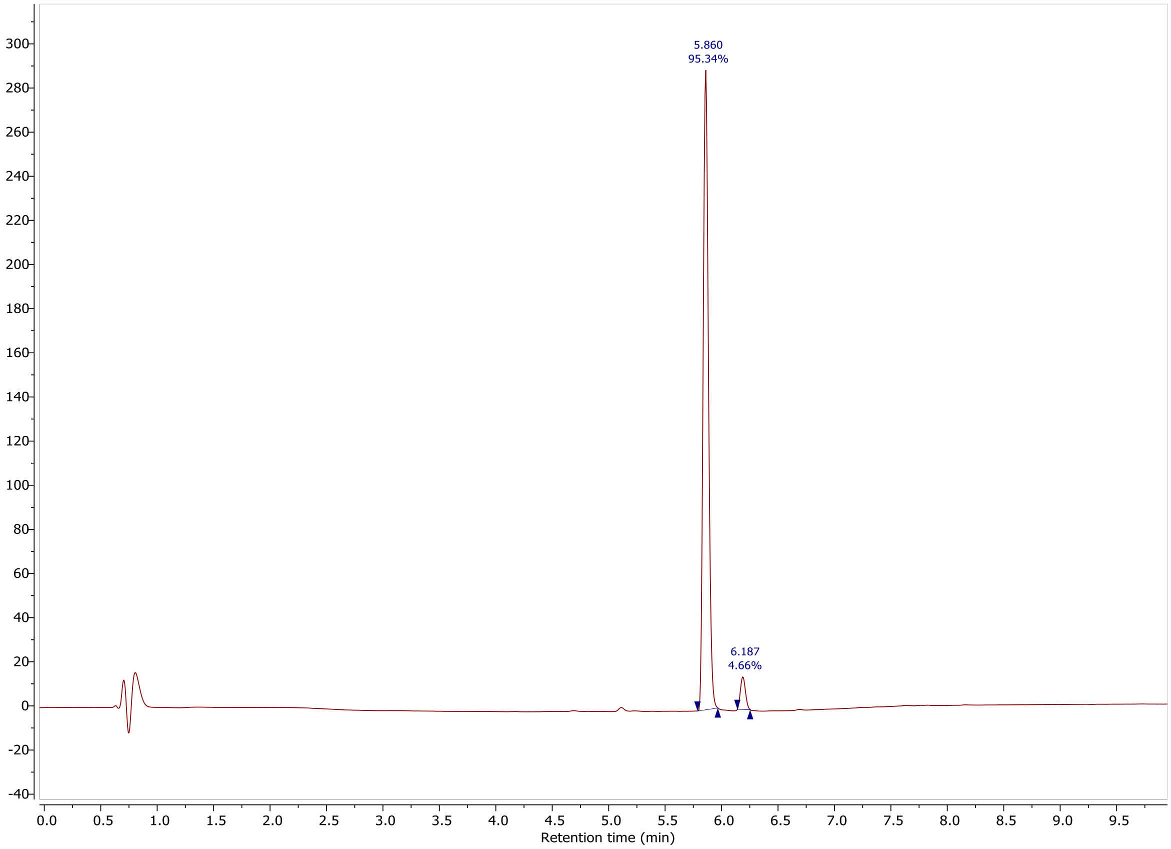
5.860
95.34%
6.187
4.66%
Retention time (min)

5c

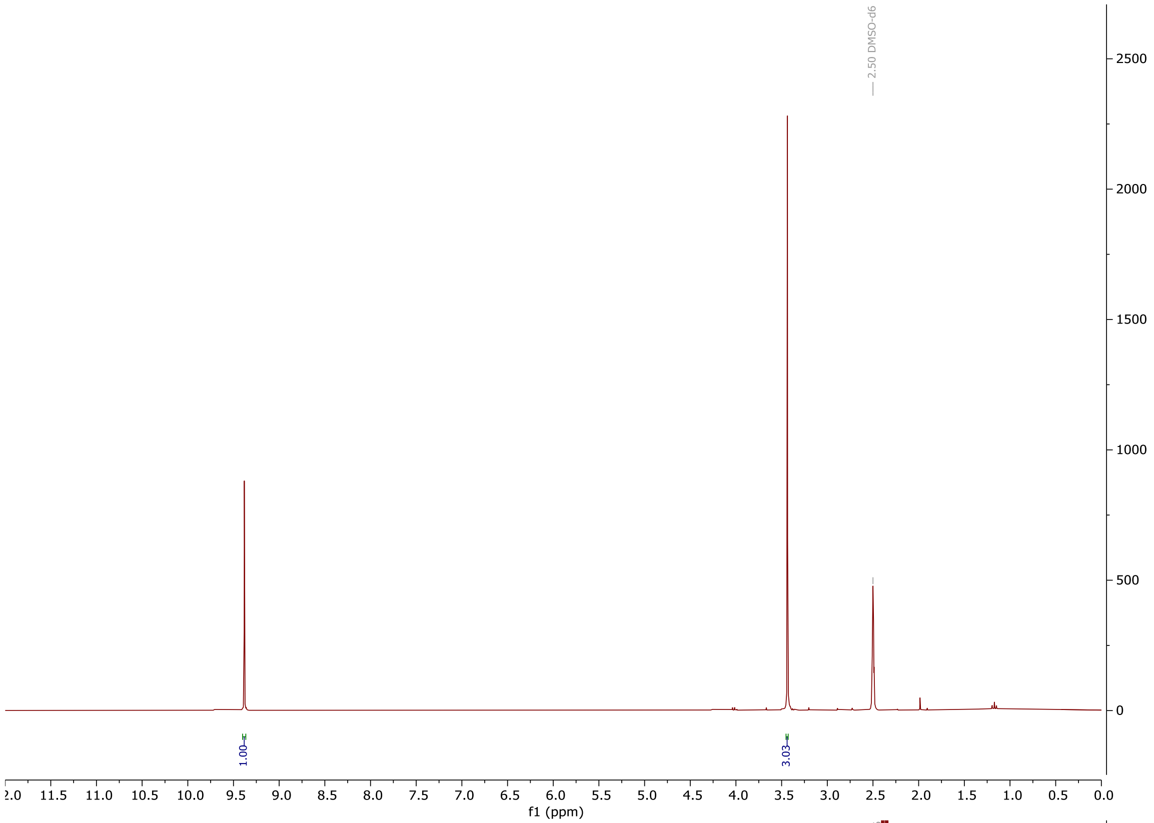
2.50 DMSO-d6
1.00
3.03
f1 (ppm)

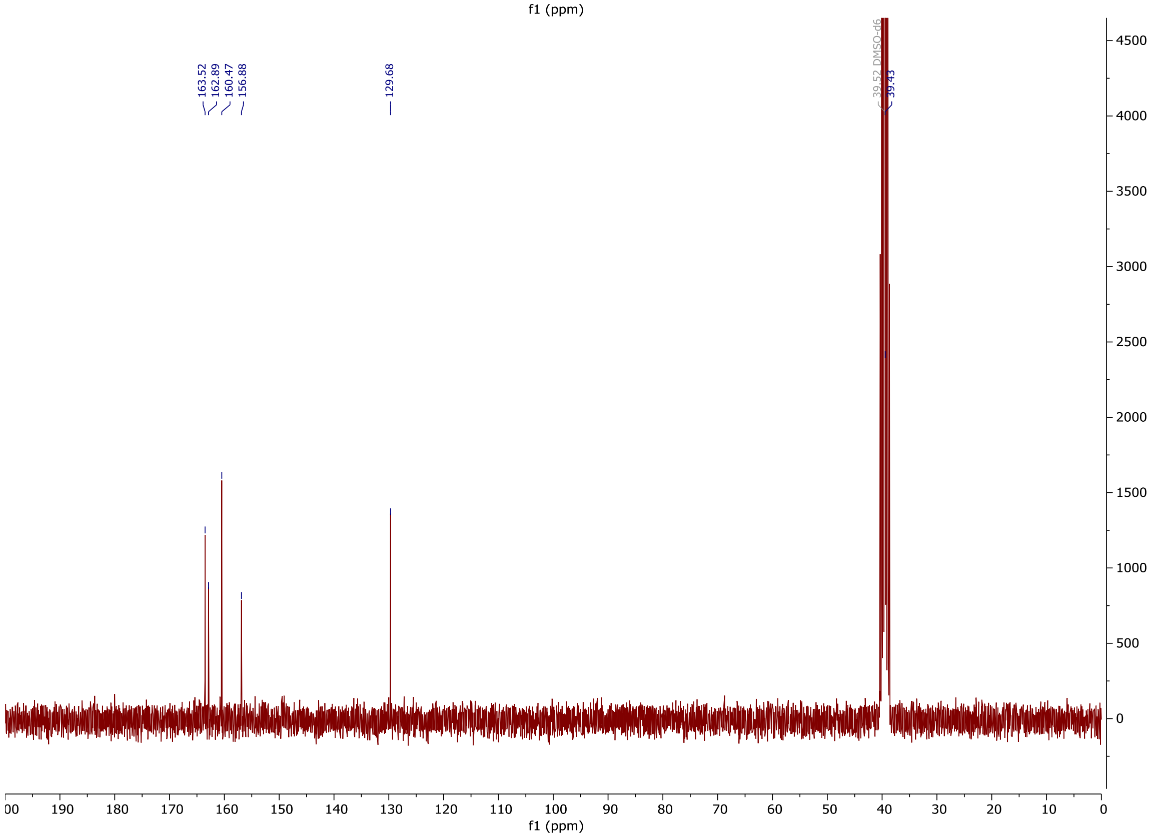
163.52
162.89
160.47
156.88
129.68
39.52 DMSO-d6
39.43
f1 (ppm)

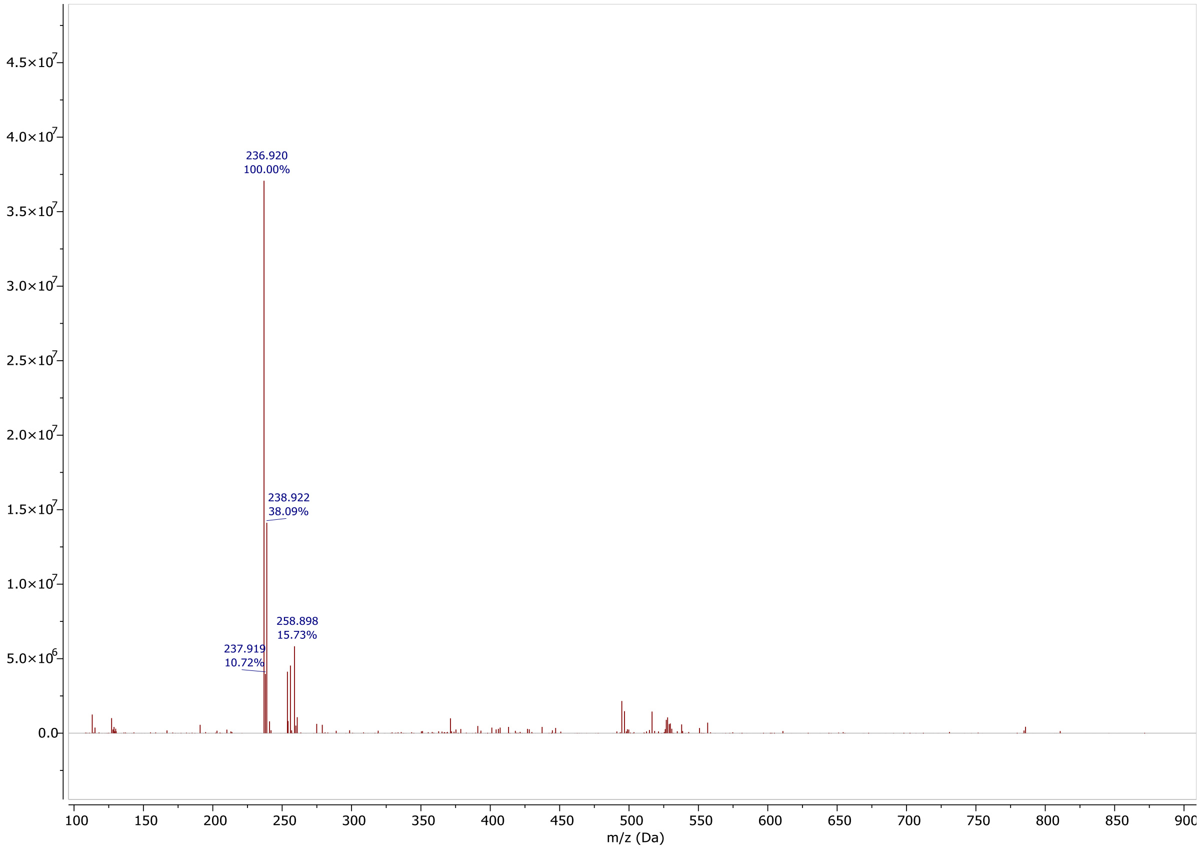
236.920
100.00%
238.922
38.09%
258.898
15.73%
237.919
10.72%
m/z (Da)

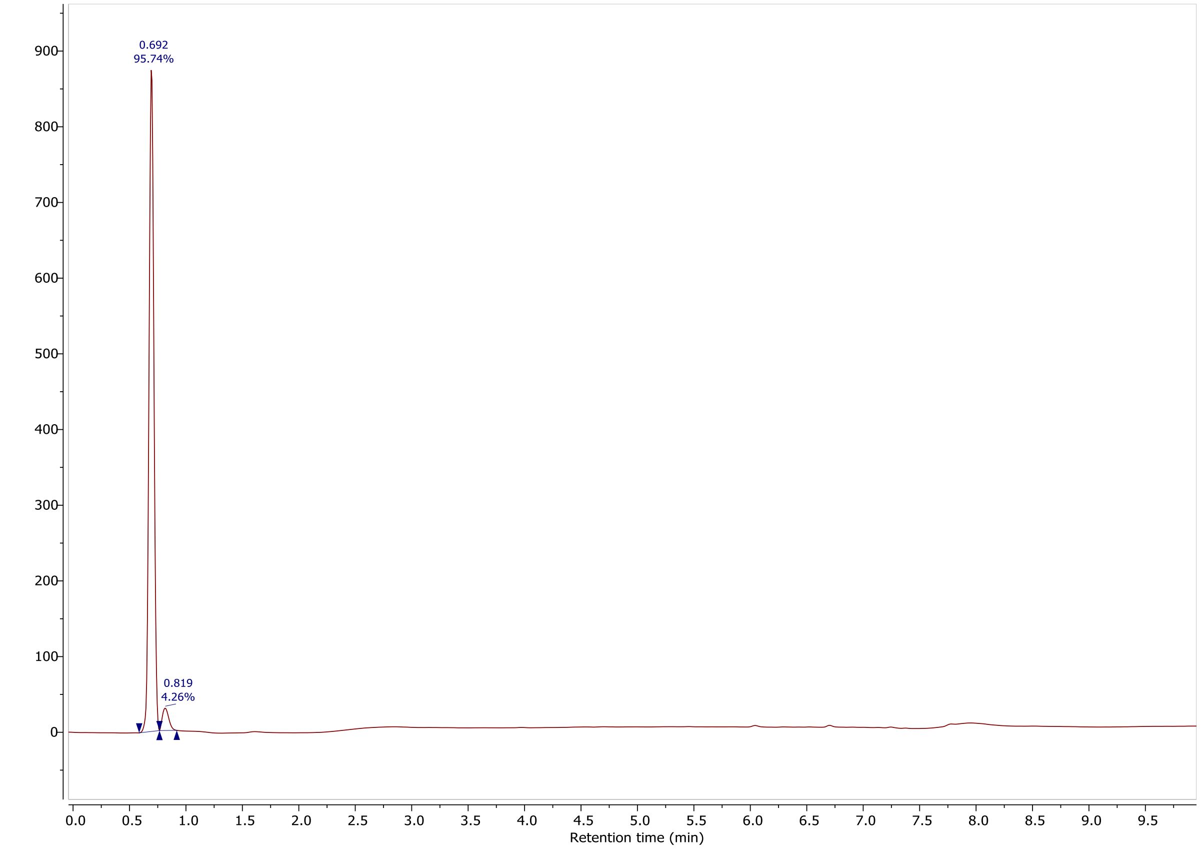
0.692
95.74%
0.819
4.26%
Retention time (min)

5d

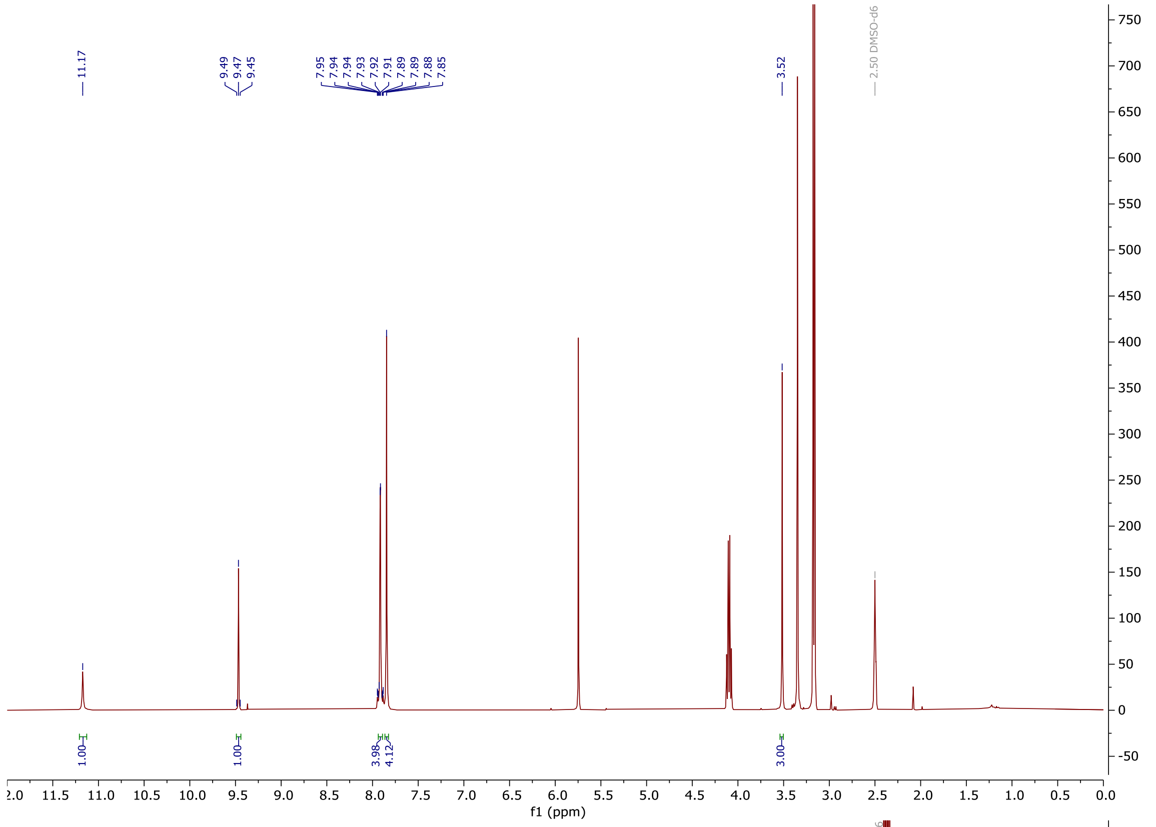
11.17
9.49
9.47
9.45
7.95
7.94
7.94
7.93
7.92
7.91
7.89
7.89
7.88
7.85
3.52
2.50 DMSO-d6
1.00
1.00
3.98
4.12
3.00
f1 (ppm)

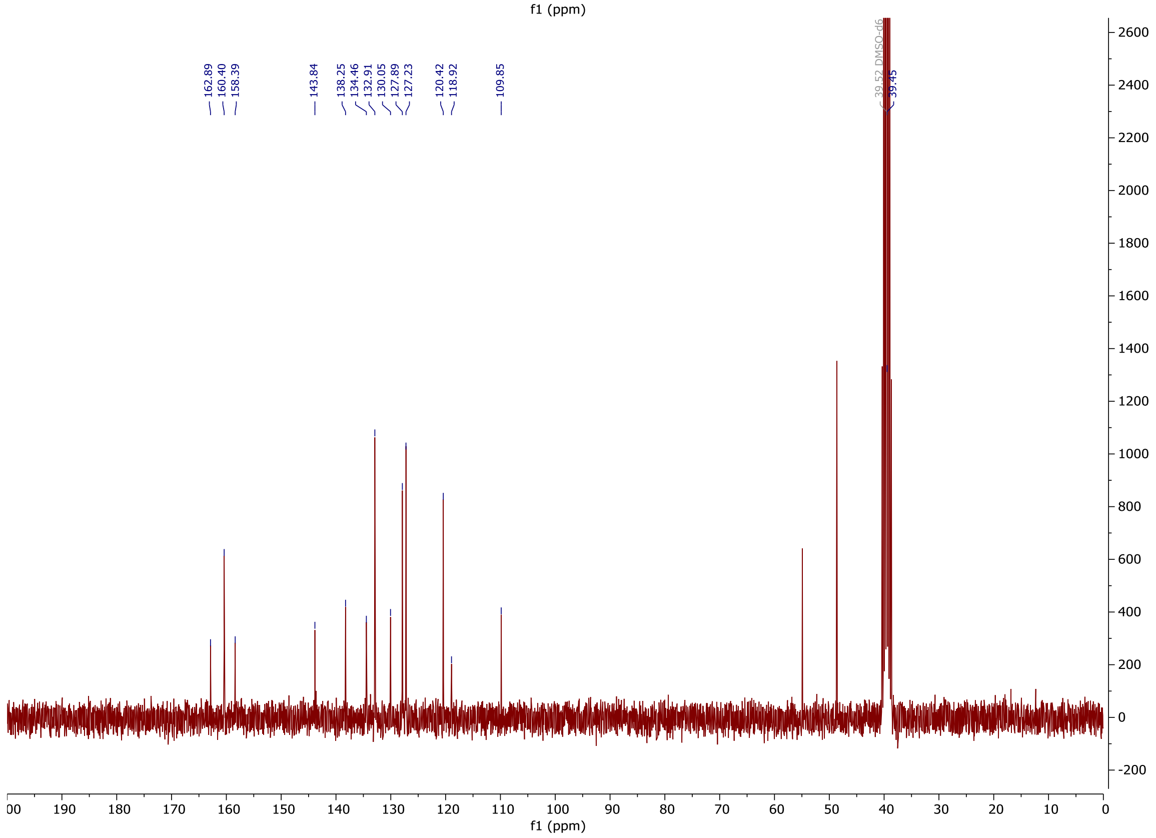
162.89
160.40
158.39
143.84
138.25
134.46
132.91
130.05
127.89
127.23
120.42
118.92
109.85
39.52 DMSO-d6
39.45
f1 (ppm)

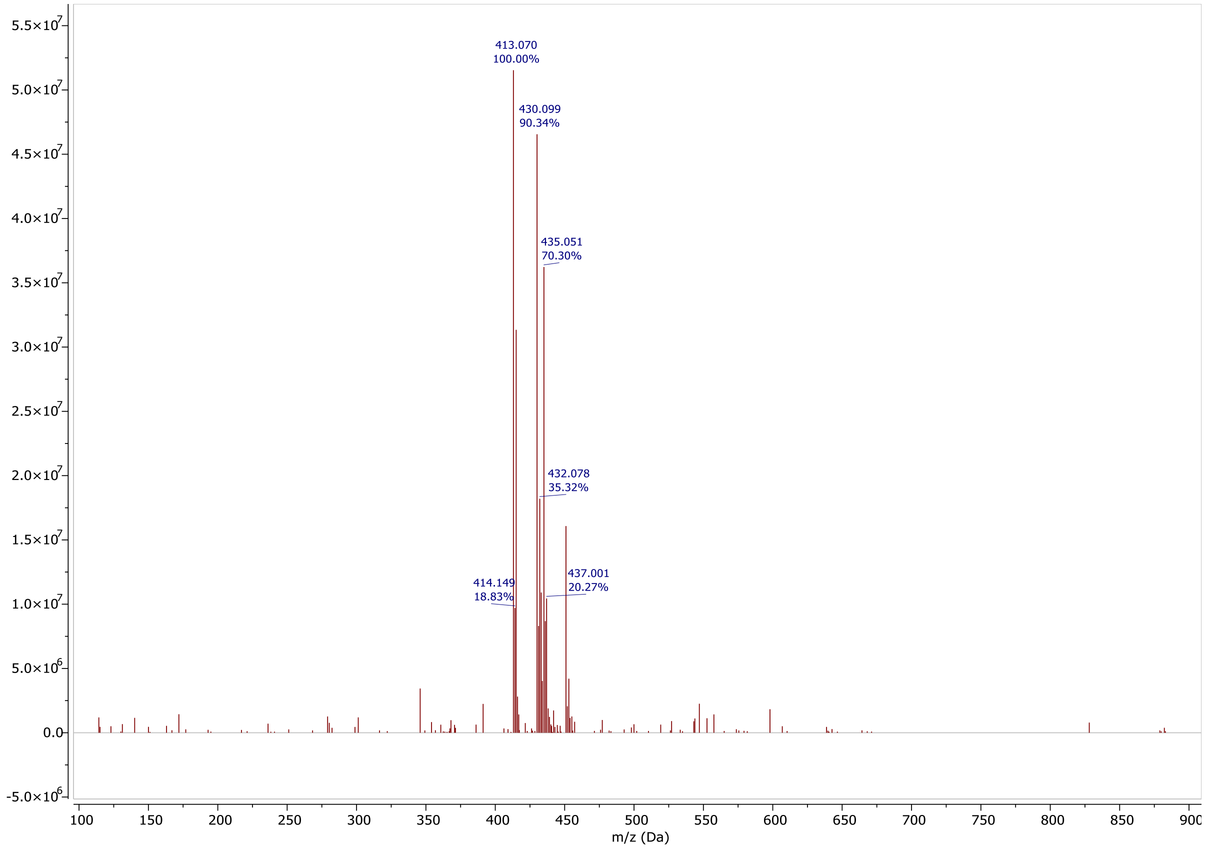

413.070
100.00%
430.099
90.34%
435.051
70.30%
432.078
35.32%
414.149
18.83%
437.001
20.27%
m/z (Da)

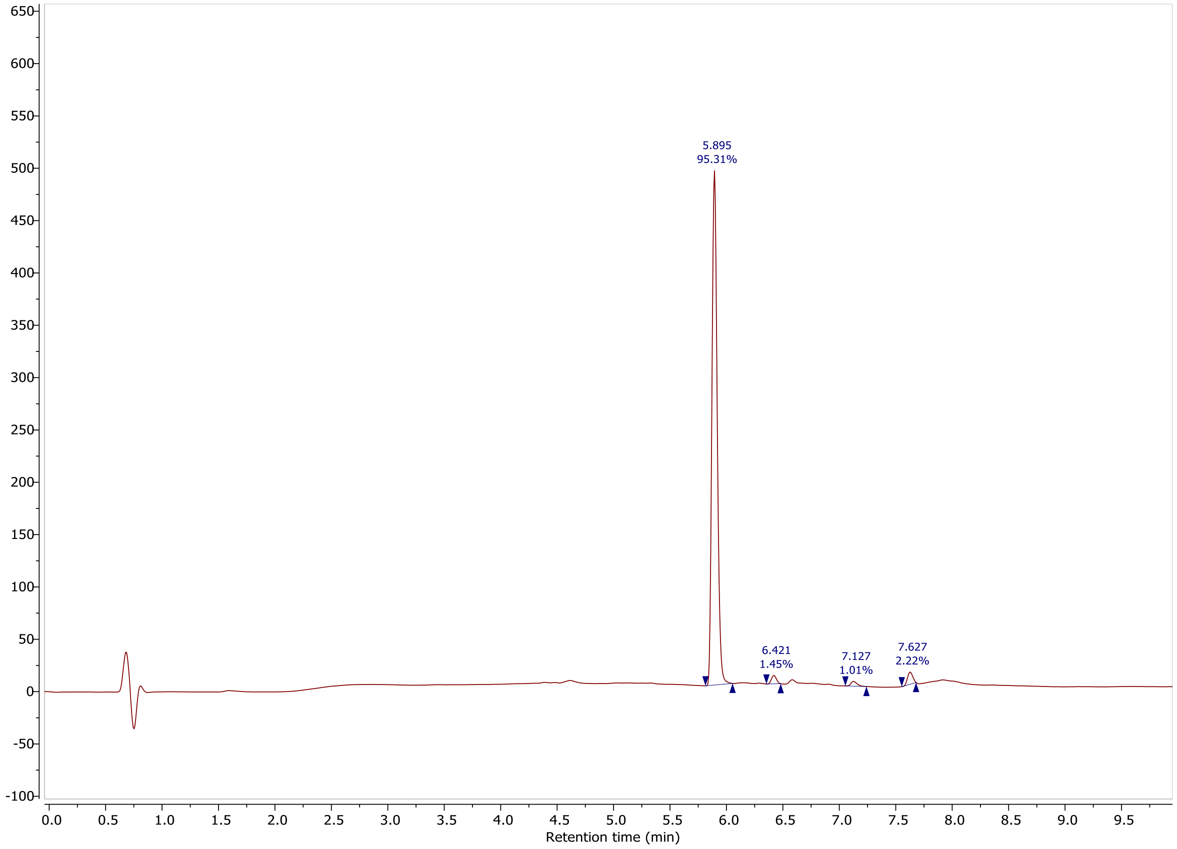

5.895
95.31%
6.421
1.45%
7.127
1.01%
7.627
2.22%
Retention time (min)

5f

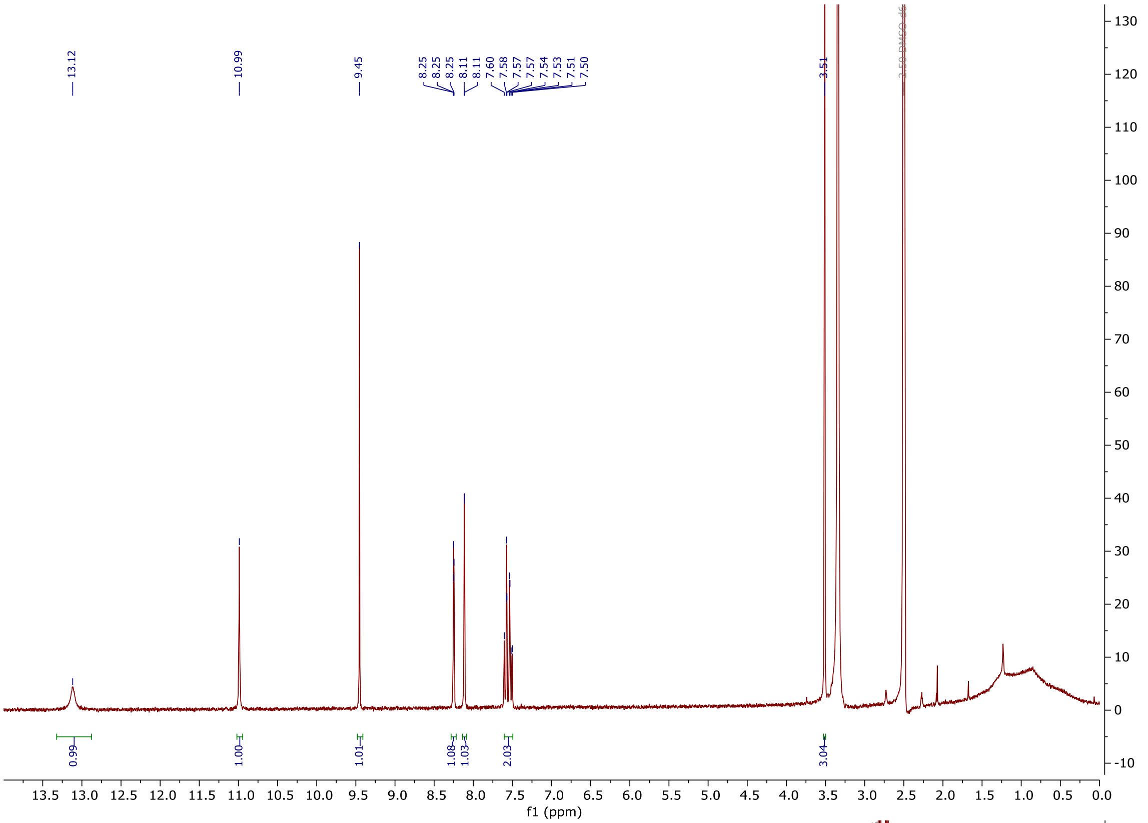
13.12
10.99
9.45
8.25
8.25
8.25
8.11
8.11
7.60
7.58
7.57
7.57
7.54
7.53
7.51
7.50
3.51
2.50 DMSO-d6
0.99
1.00
1.01
1.08
1.03
2.03
3.04
f1 (ppm)

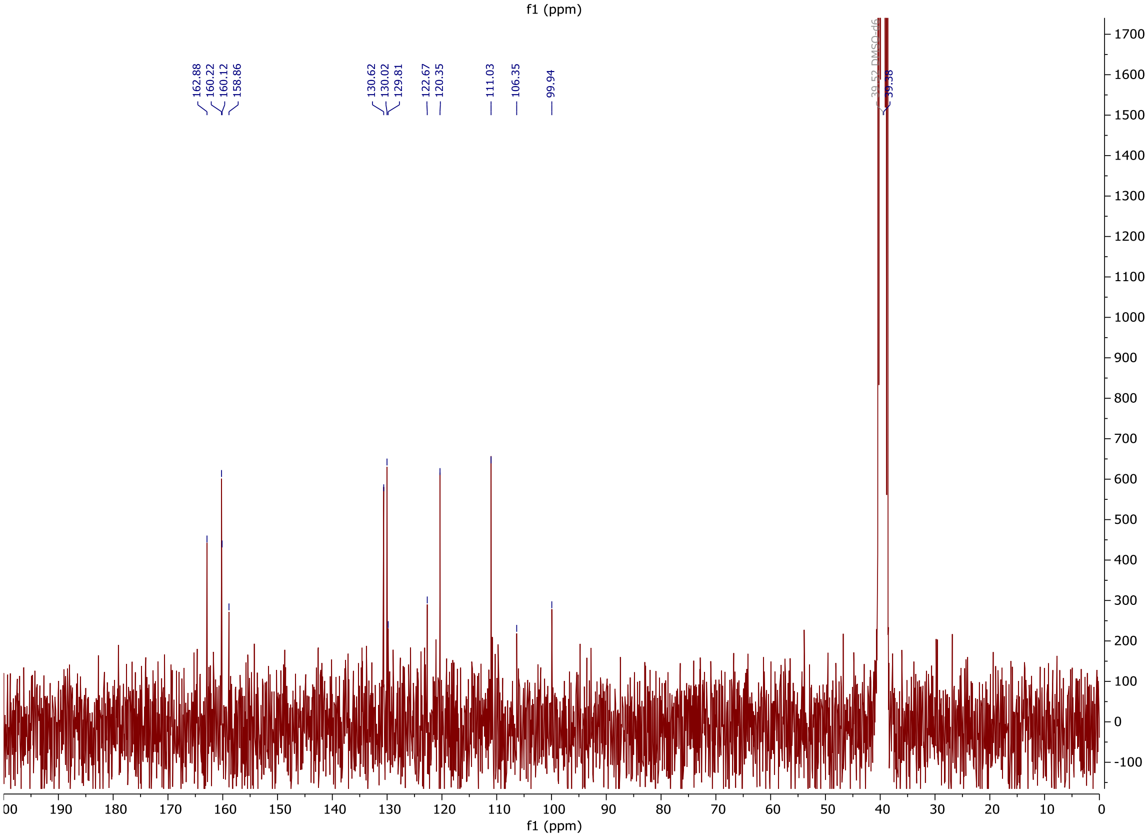
162.88
160.22
160.12
158.86
130.62
130.02
129.81
122.67
120.35
111.03
106.35
99.94
39.52 DMSO-d6
39.38
f1 (ppm)

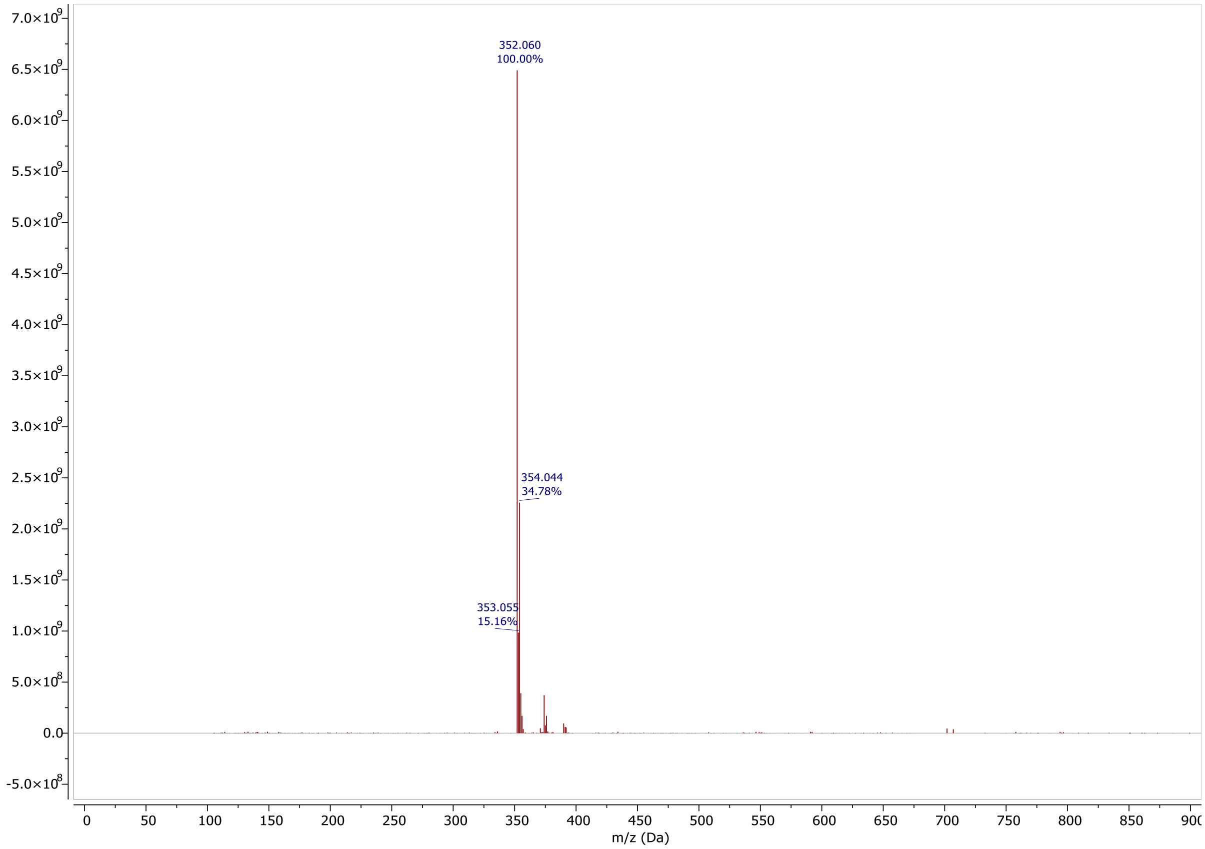
352.060
100.00%
354.044
34.78%
353.055
15.16%
m/z (Da)

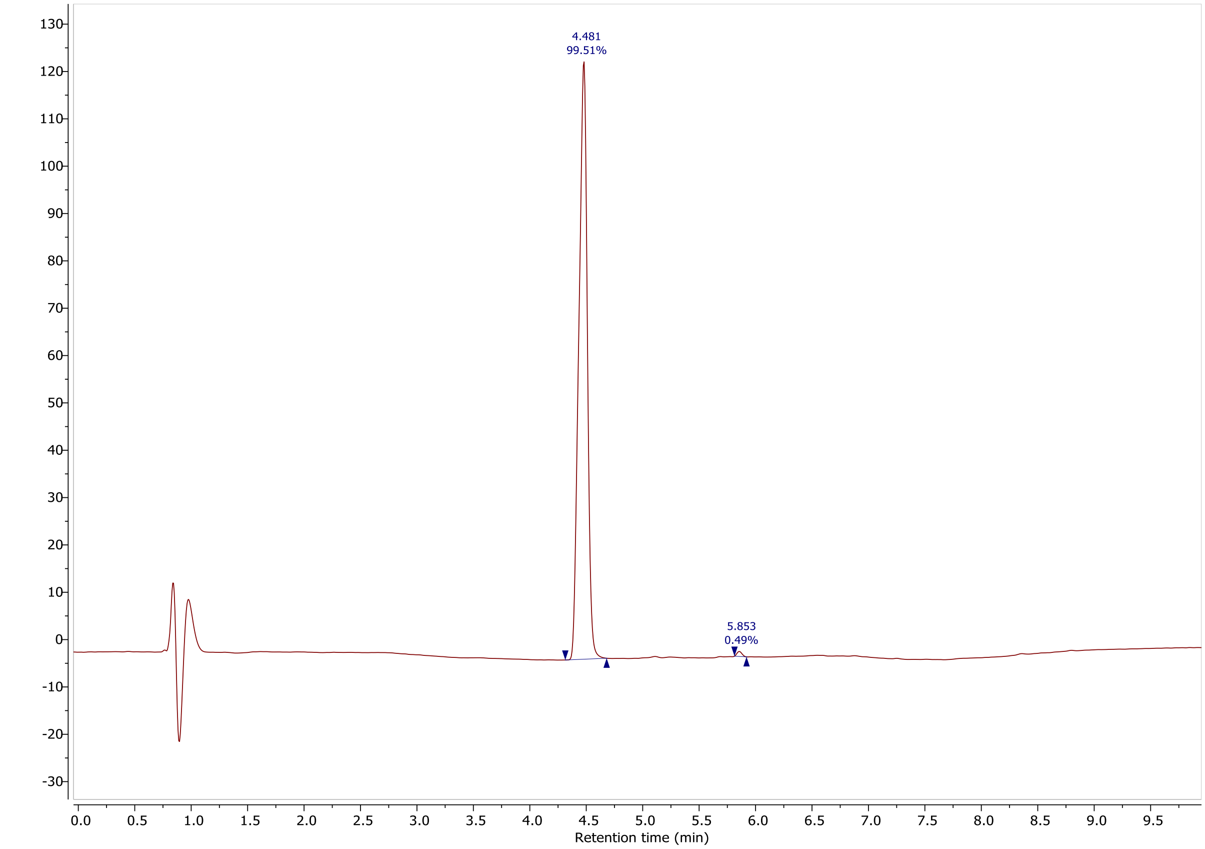
4.481
99.51%
5.853
0.49%
Retention time (min)

5g

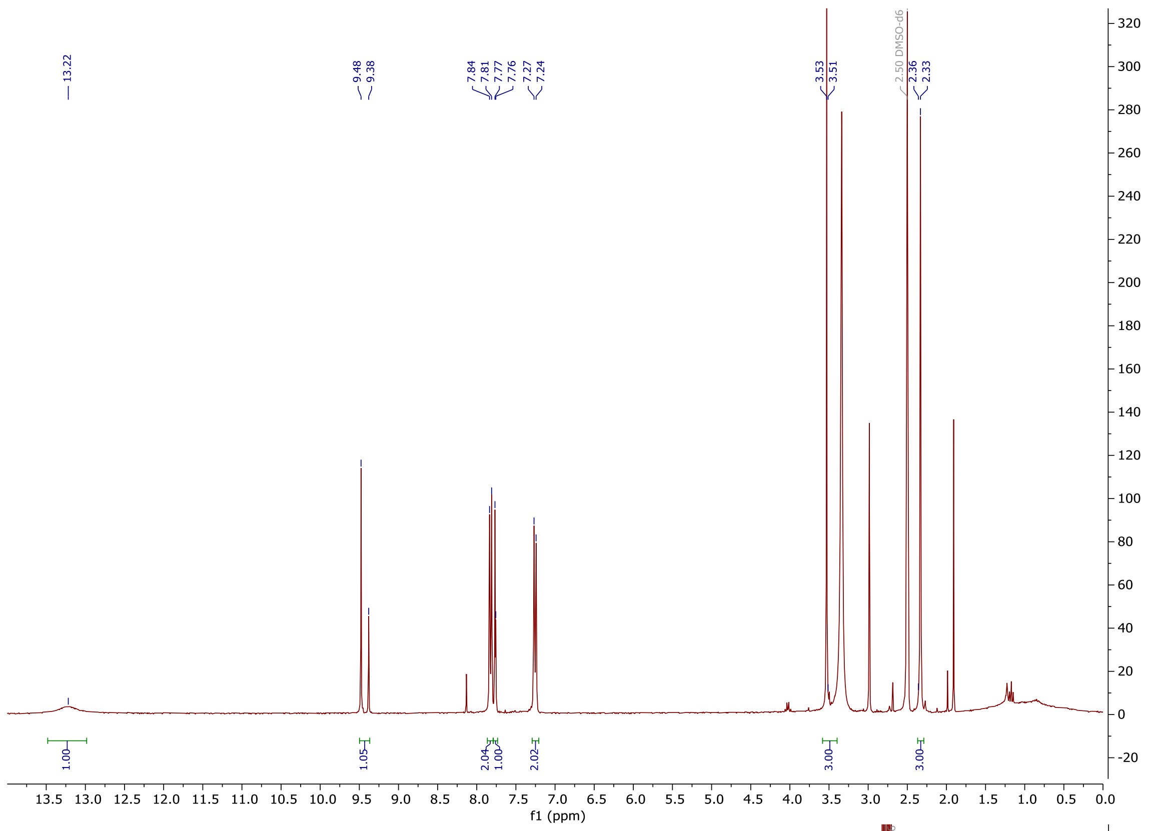
13.22
9.48
9.38
7.84
7.81
7.77
7.76
7.27
7.24
3.53
3.51
2.50 DMSO-d6
2.36
2.33
1.00
1.05
2.04
1.00
2.02
3.00
3.00
f1 (ppm)

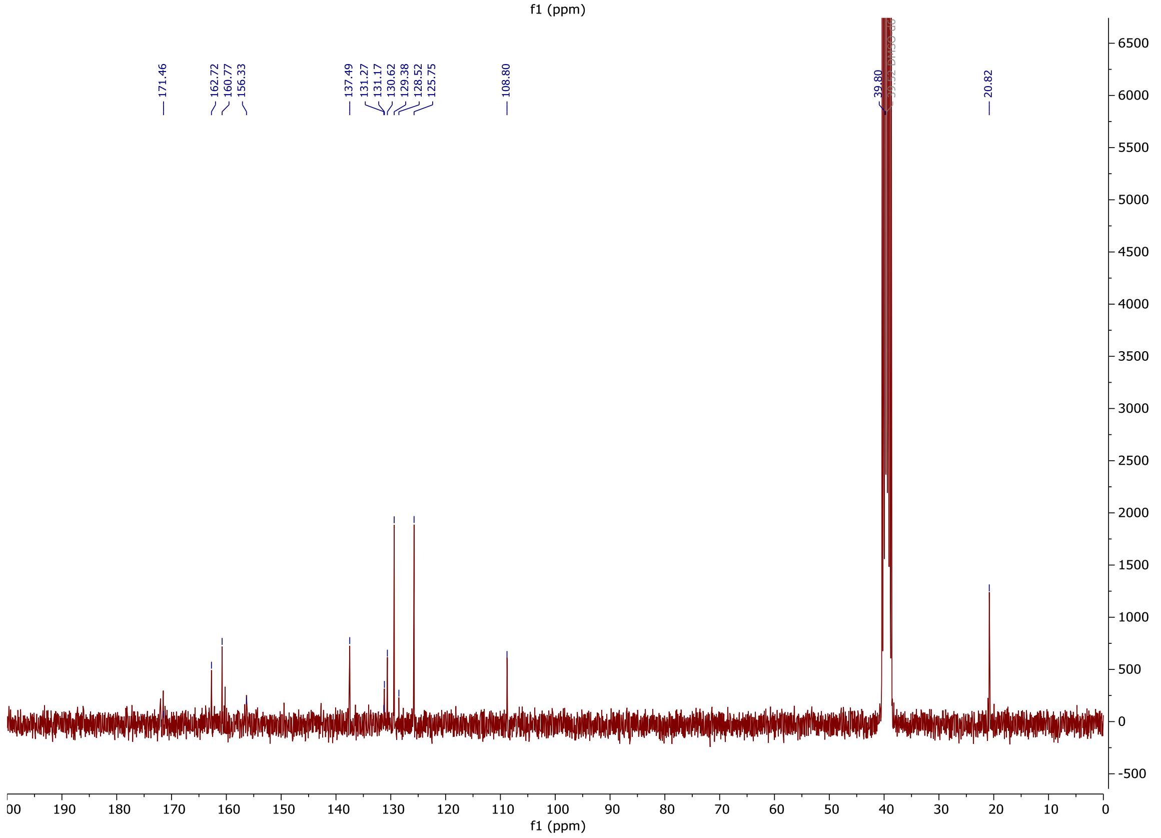
171.46
162.72
160.77
156.33
137.49
131.27
131.17
130.62
129.38
128.52
125.75
108.80
39.80
20.82
f1 (ppm)

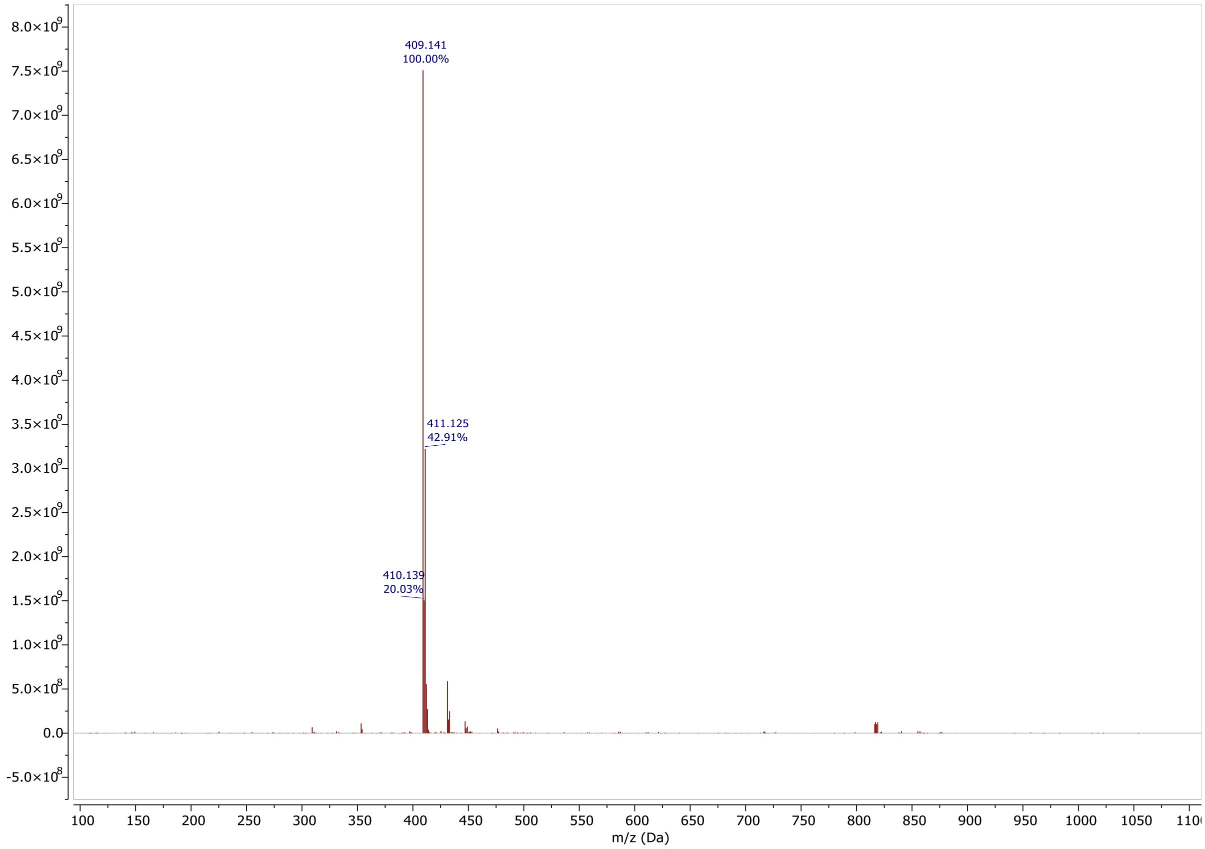
409.141
100.00%
411.125
42.91%
410.139
20.03%
m/z (Da)

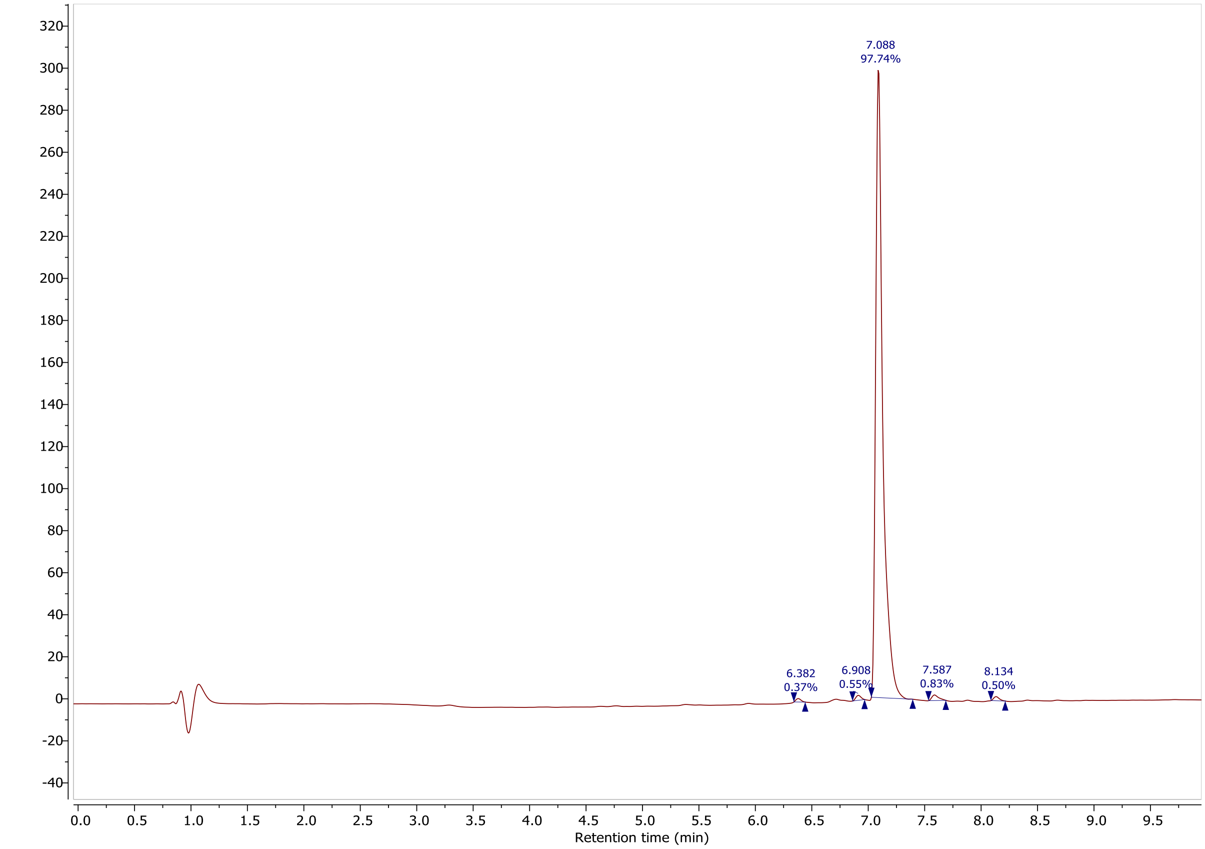
7.088
97.74%
6.382
0.37%
6.908
0.55%
7.587
0.83%
8.134
0.50%
Retention time (min)

5h

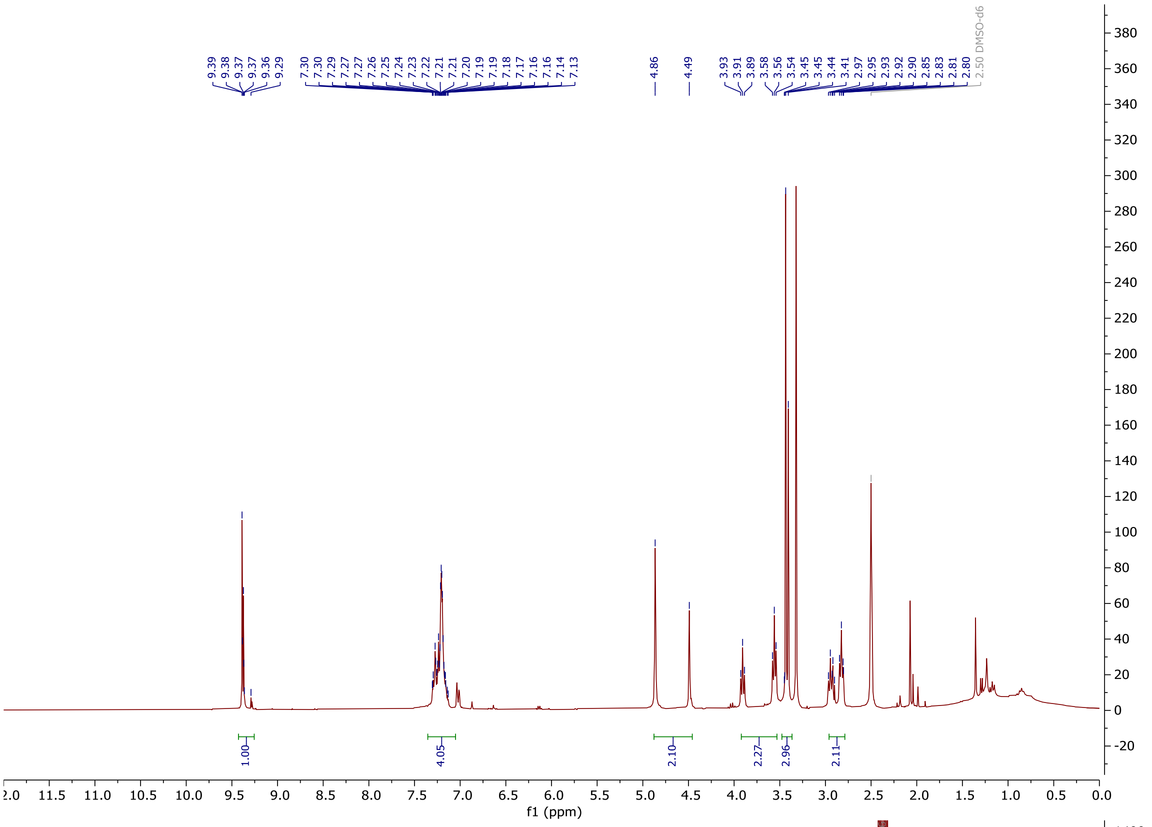
9.39
9.38
9.37
9.37
9.36
9.29
7.30
7.30
7.29
7.27
7.27
7.26
7.25
7.24
7.23
7.22
7.21
7.21
7.20
7.19
7.19
7.18
7.17
7.16
7.16
7.14
7.13
4.86
4.49
3.93
3.91
3.89
3.58
3.56
3.54
3.45
3.45
3.44
3.41
2.97
2.95
2.93
2.92
2.90
2.85
2.83
2.81
2.80
2.50 DMSO-d6
1.00
4.05
2.10
2.27
2.96
2.11
f1 (ppm)

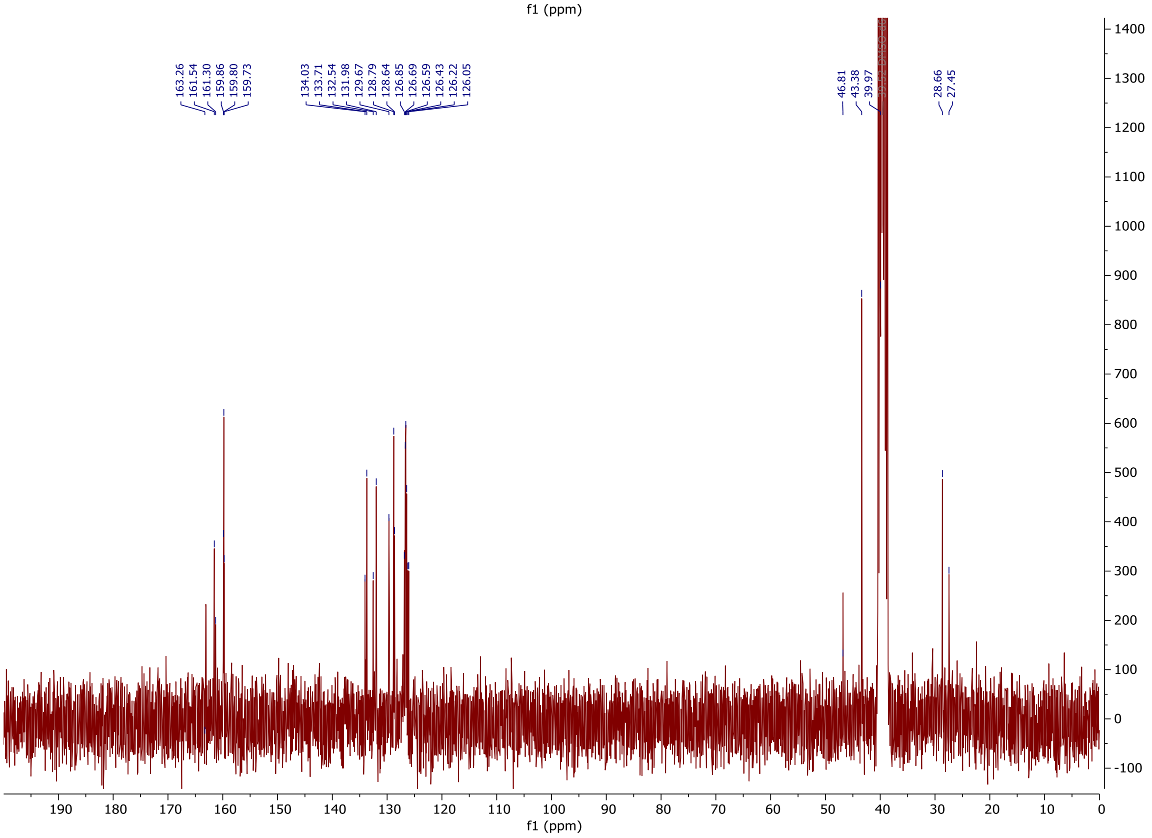
163.26
161.54
161.30
159.86
159.80
159.73
134.03
133.71
132.54
131.98
129.67
128.79
128.64
126.85
126.69
126.59
126.43
126.22
126.05
46.81
43.38
39.97
28.66
27.45
f1 (ppm)

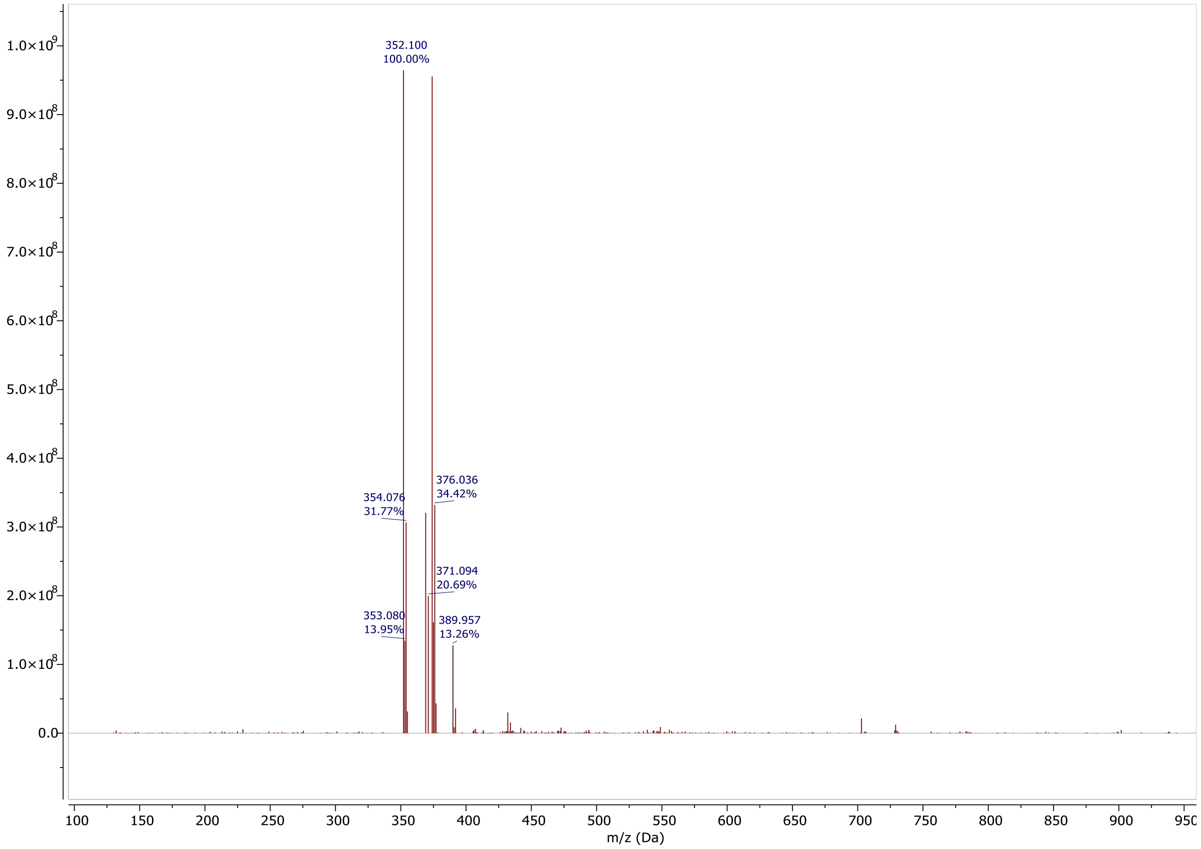
352.100
100.00%
354.076
31.77%
376.036
34.42%
371.094
20.69%
353.080
13.95%
389.957
13.26%
m/z (Da)

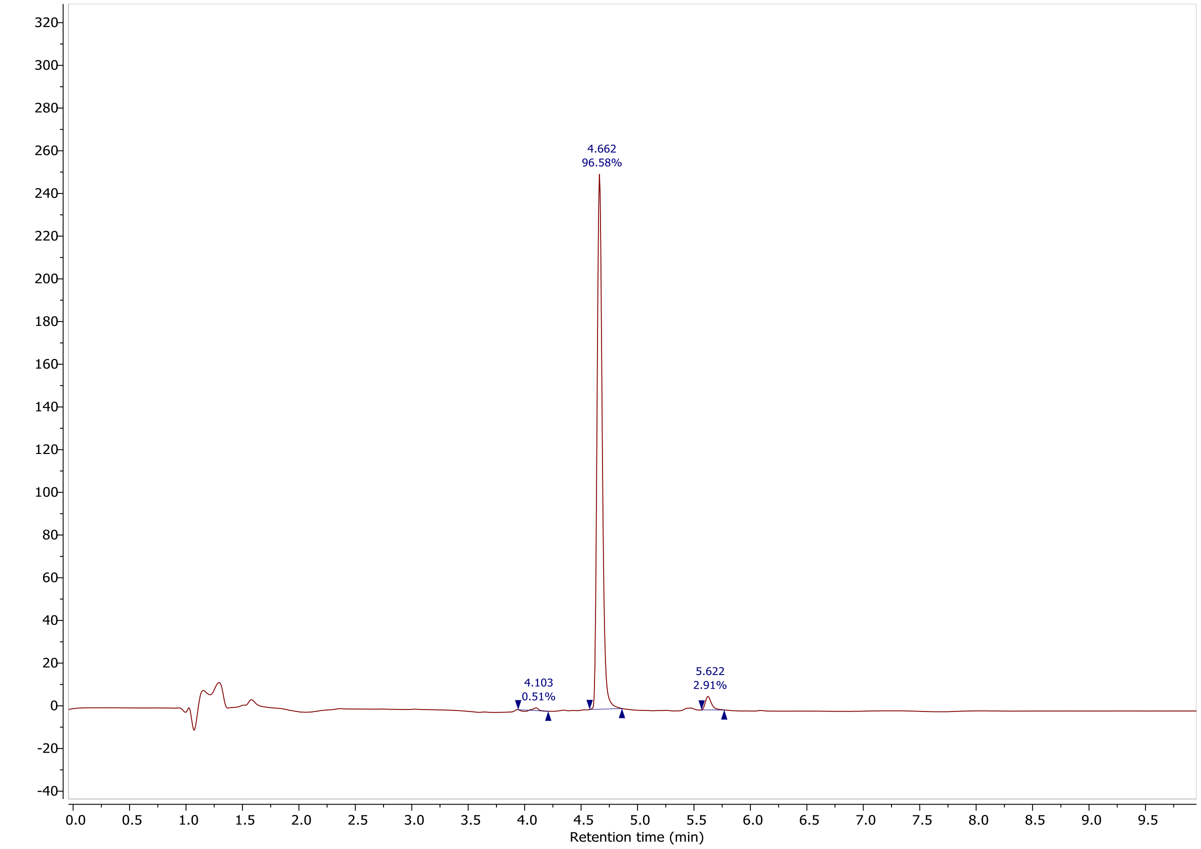
4.662
96.58%
4.103
0.51%
5.622
2.91%
Retention time (min)

5i

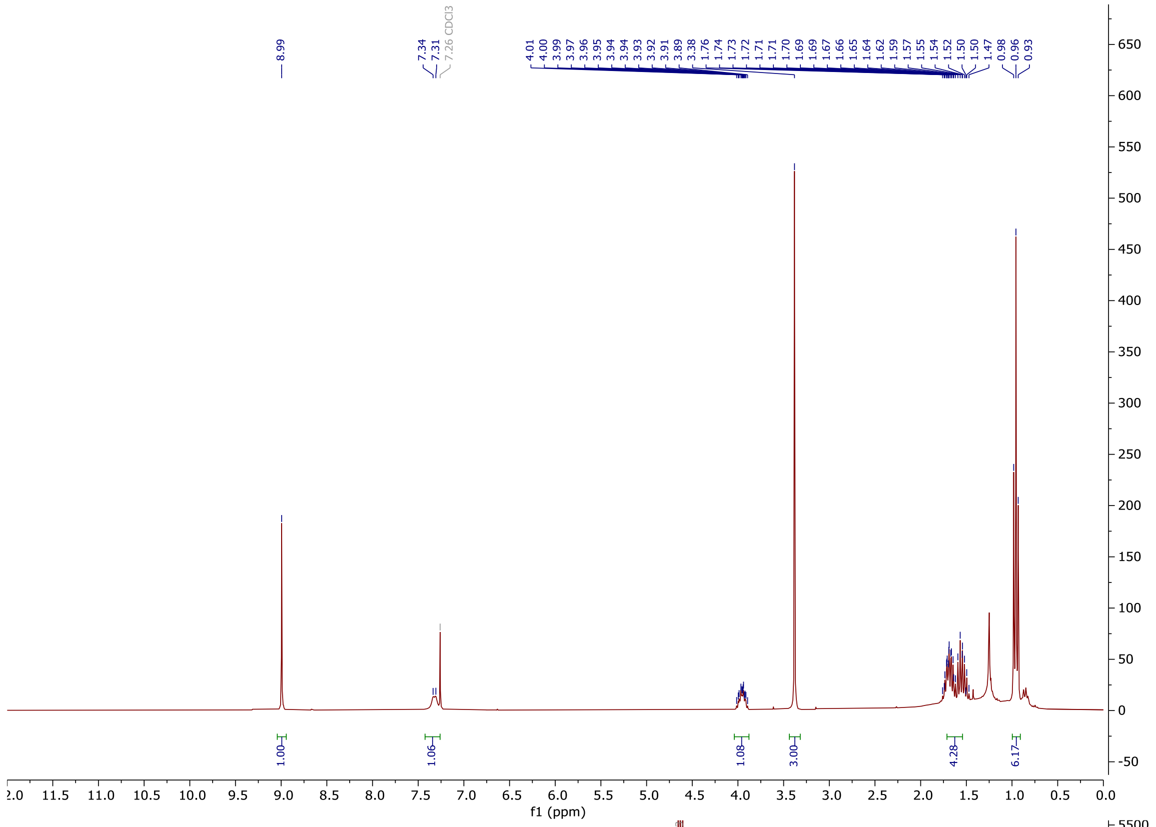

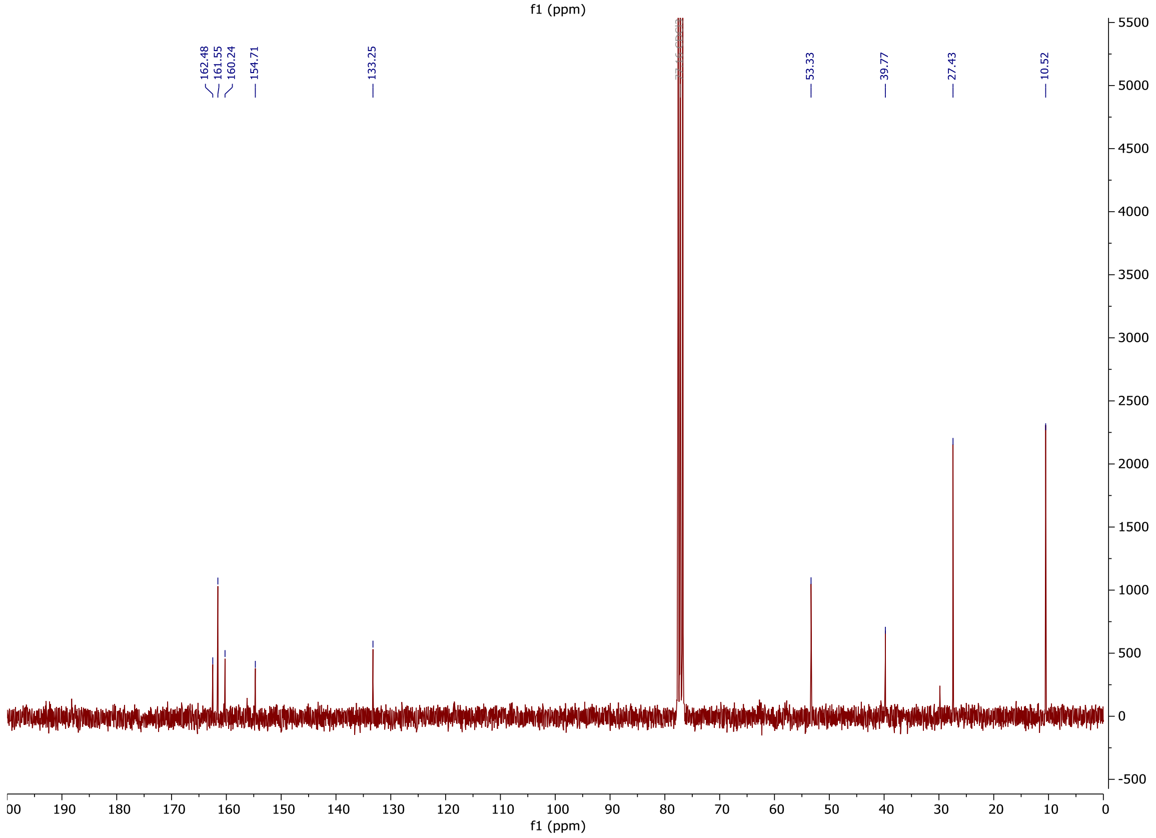

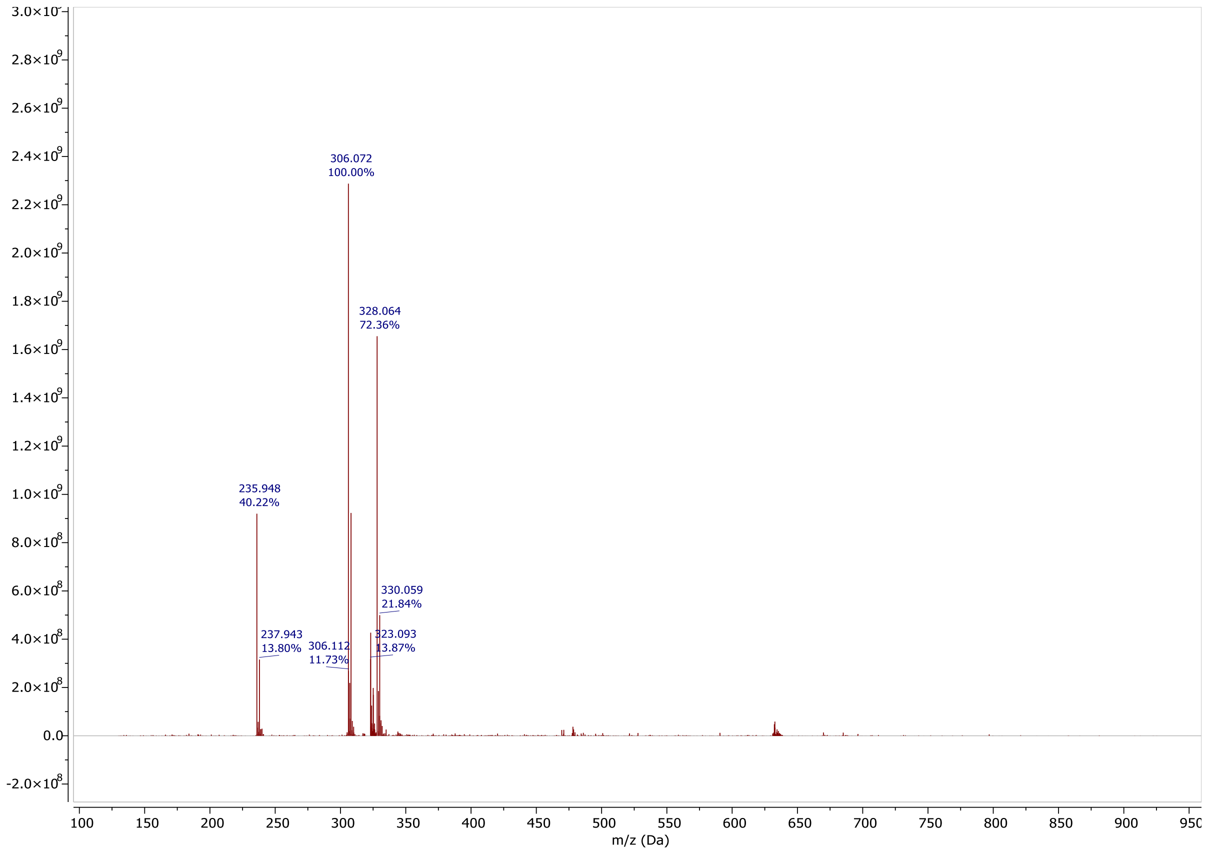
306.072
100.00%
328.064
72.36%
235.948
40.22%
330.059
21.84%
237.943
13.80%
306.112
11.73%
323.093
13.87%
m/z (Da)

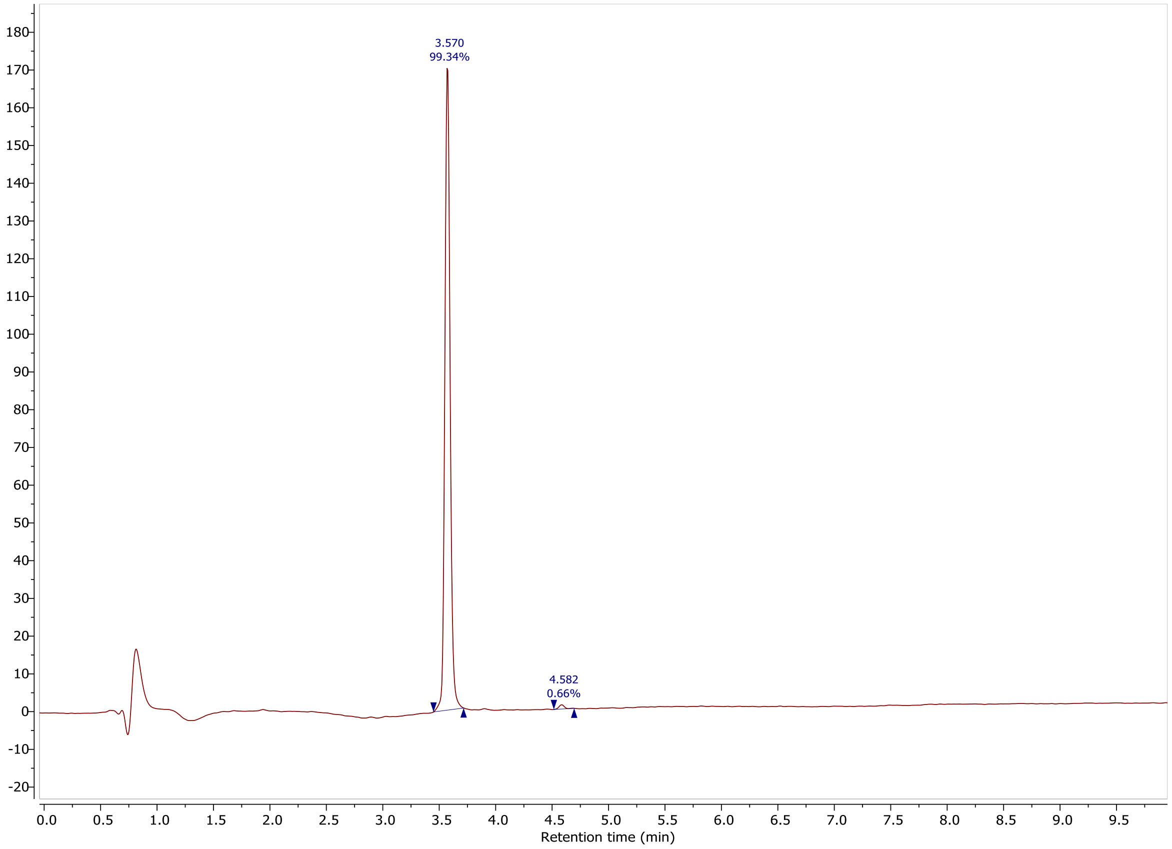
3.570
99.34%
4.582
0.66%
Retention time (min)

5k

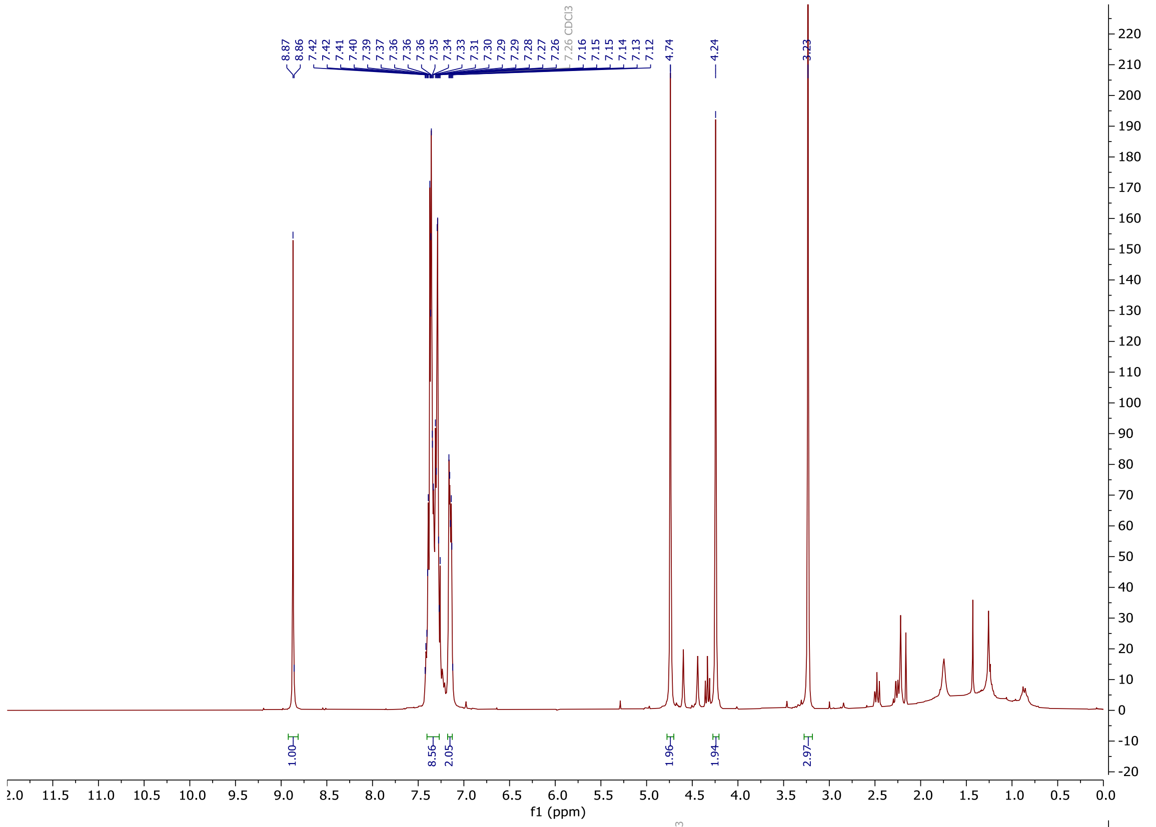
8.87
8.86
7.42
7.42
7.41
7.40
7.39
7.37
7.36
7.36
7.36
7.35
7.34
7.33
7.31
7.30
7.29
7.29
7.28
7.27
7.26
7.26 CDCl3
7.16
7.15
7.15
7.14
7.13
7.12
4.74
4.24
3.23
1.00
8.56
2.05
1.96
1.94
2.97
f1 (ppm)

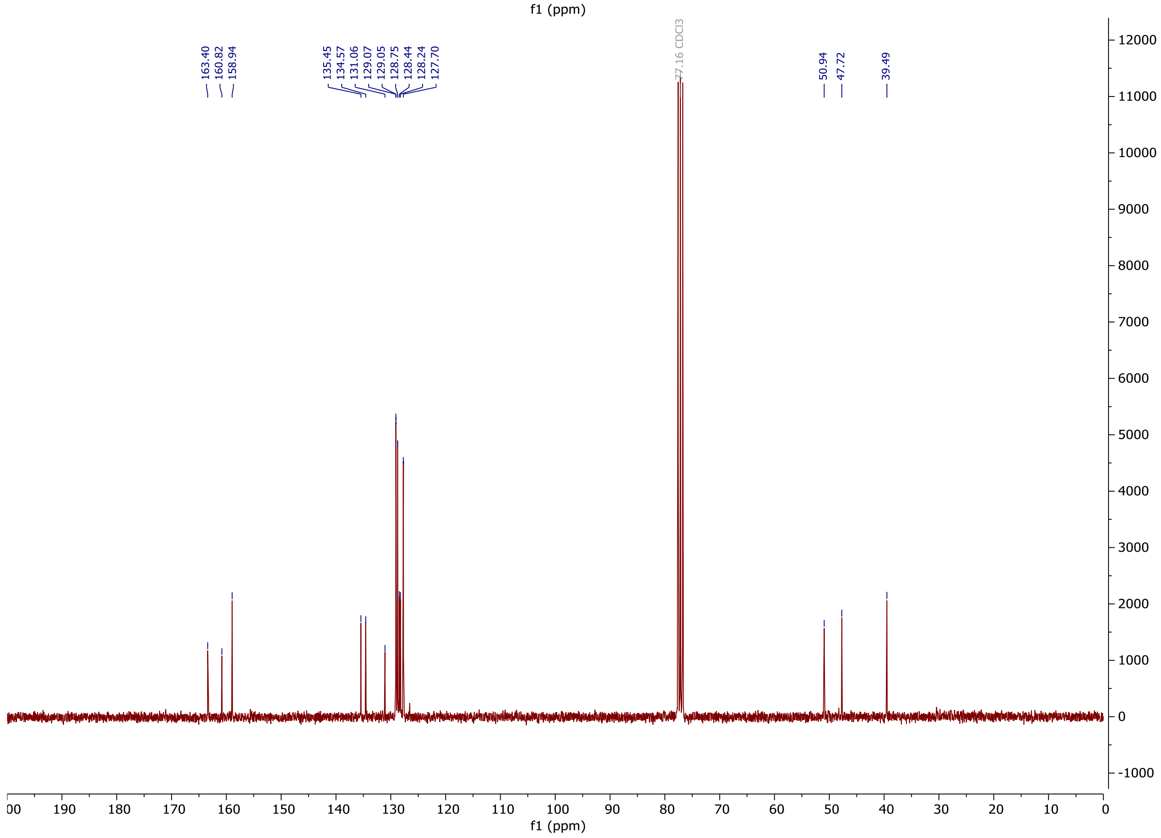
163.40
160.82
158.94
135.45
134.57
131.06
129.07
129.05
128.75
128.44
128.24
127.70
77.16 CDCl3
50.94
47.72
39.49
f1 (ppm)

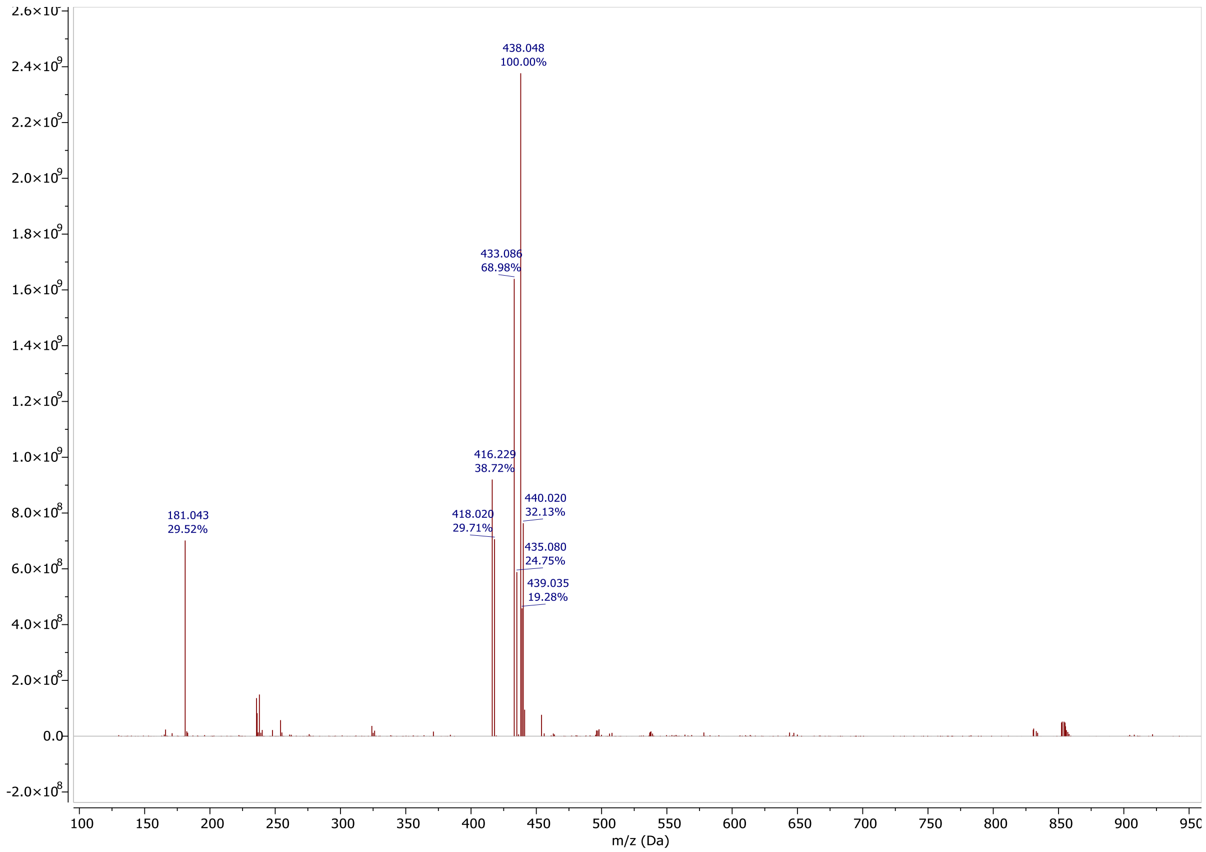
438.048
100.00%
433.086
68.98%
416.229
38.72%
181.043
29.52%
418.020
29.71%
440.020
32.13%
435.080
24.75%
439.035
19.28%
m/z (Da)

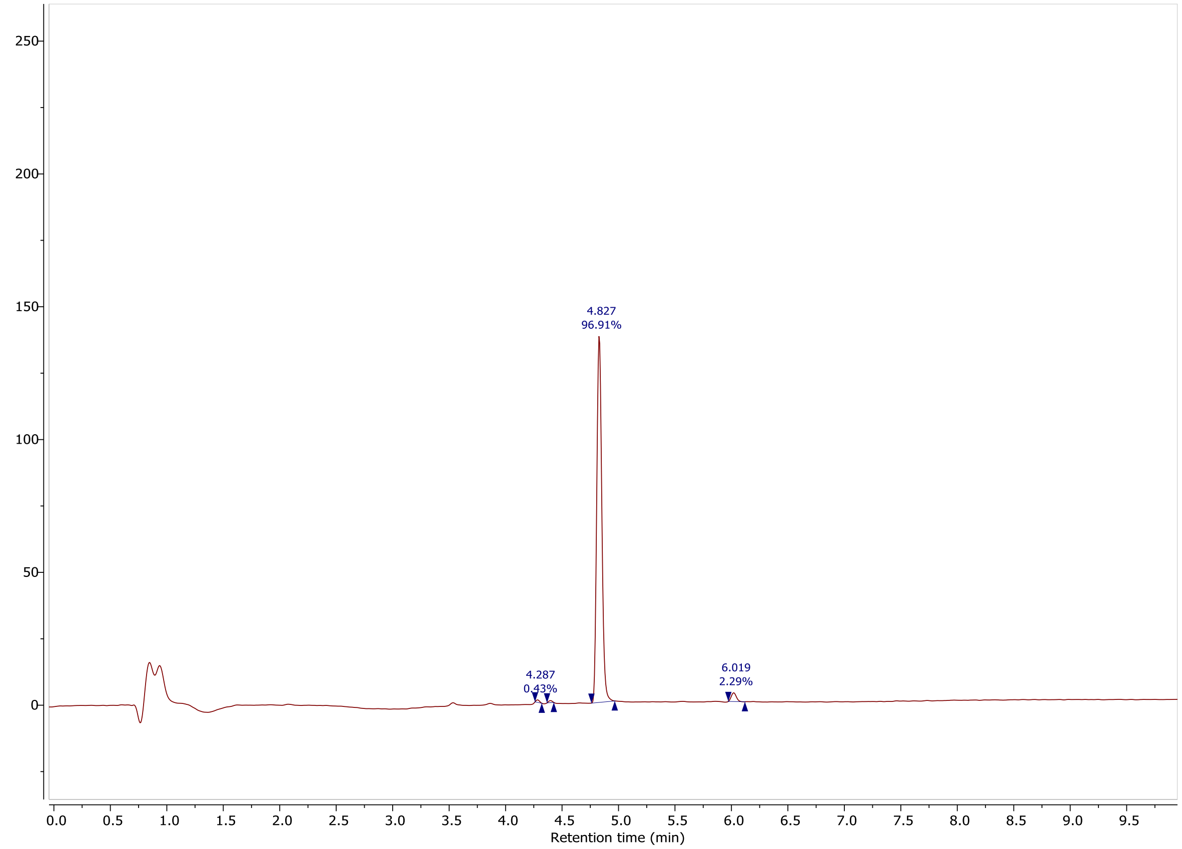
4.827
96.91%
4.287
0.43%
6.019
2.29%
Retention time (min)

6a

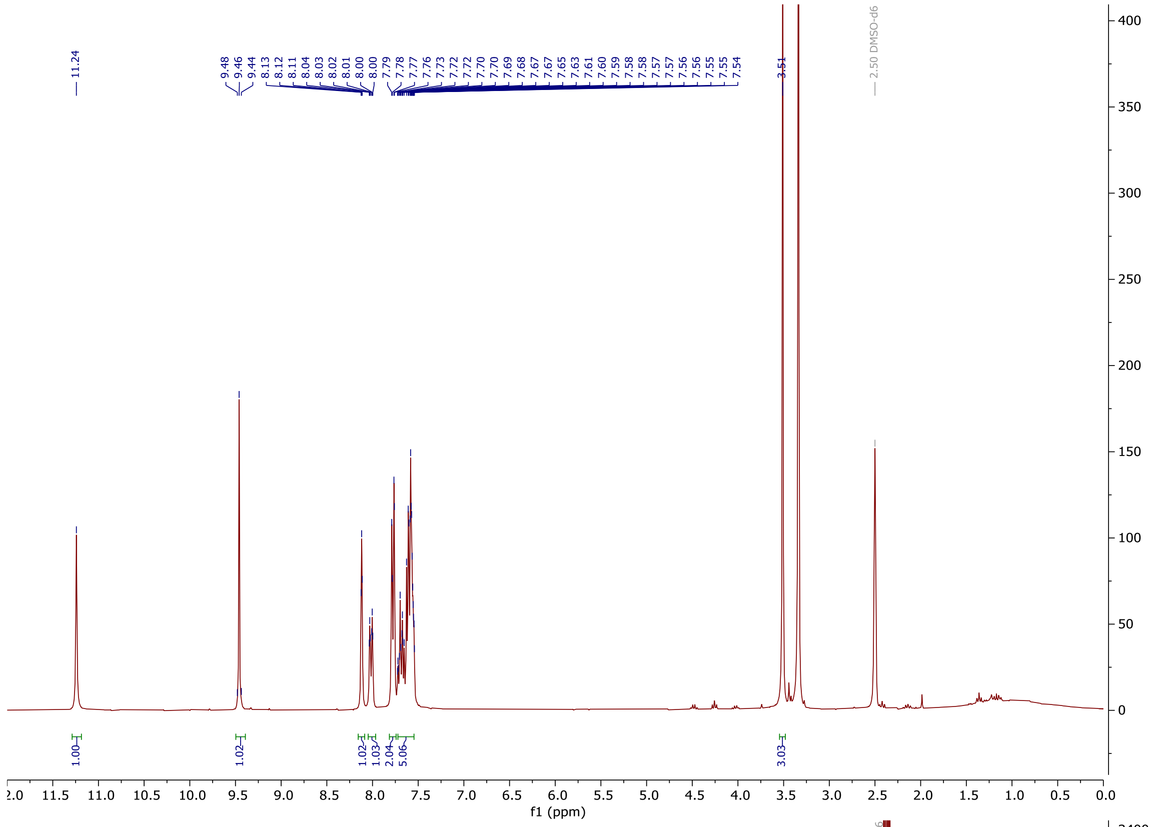

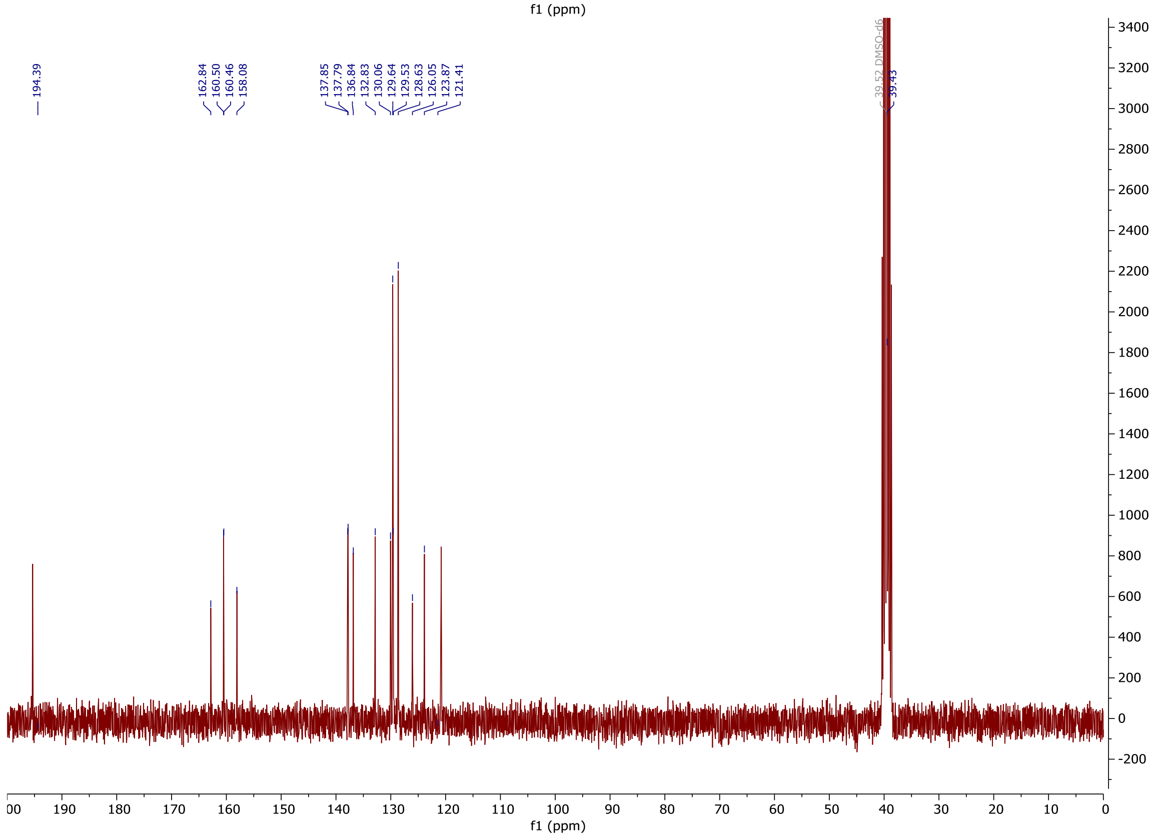

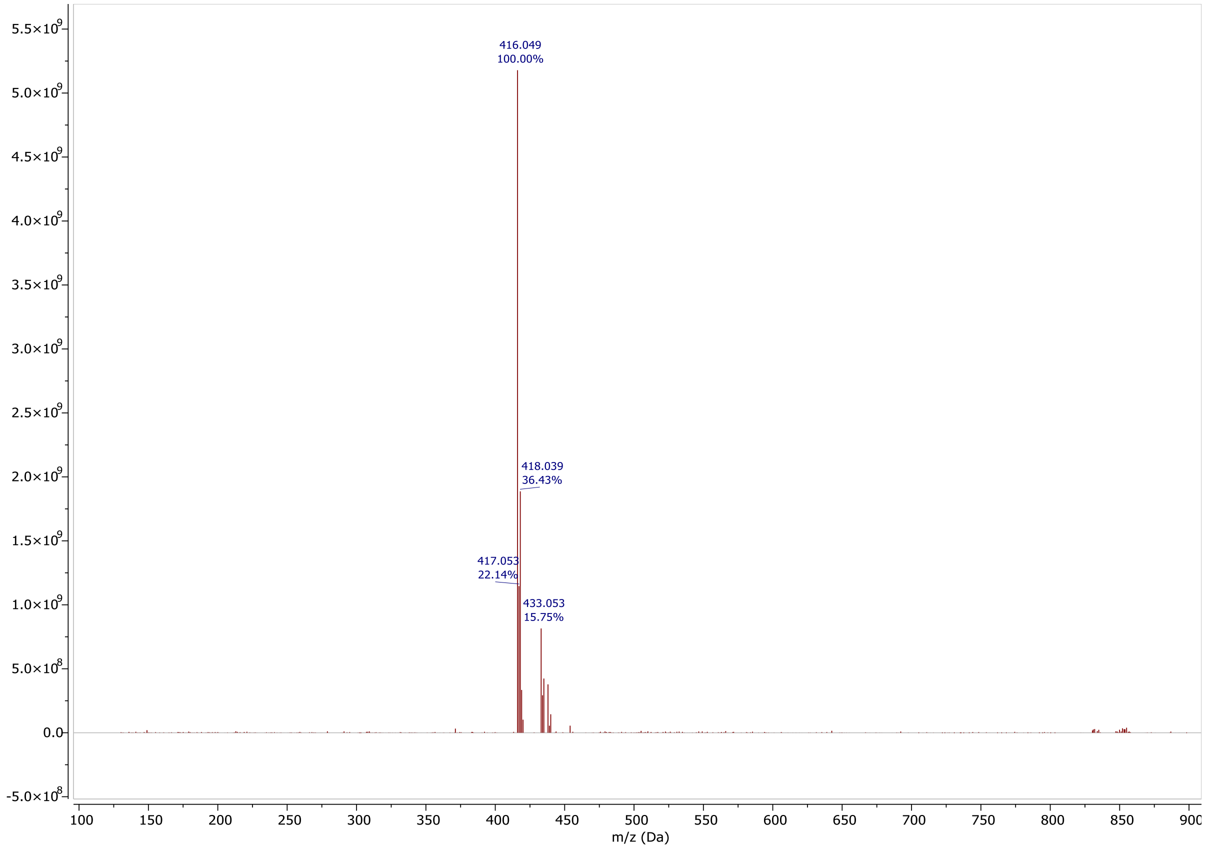
416.049
100.00%
418.039
36.43%
417.053
22.14%
433.053
15.75%
5.5×10^9
5.0×10^9
4.5×10^9
4.0×10^9
3.5×10^9
3.0×10^9
2.5×10^9
2.0×10^9
1.5×10^9
1.0×10^9
5.0×10^8
0.0
-5.0×10^8
100
150
200
250
300
350
400
450
500
550
600
650
700
750
800
850
900
m/z (Da)

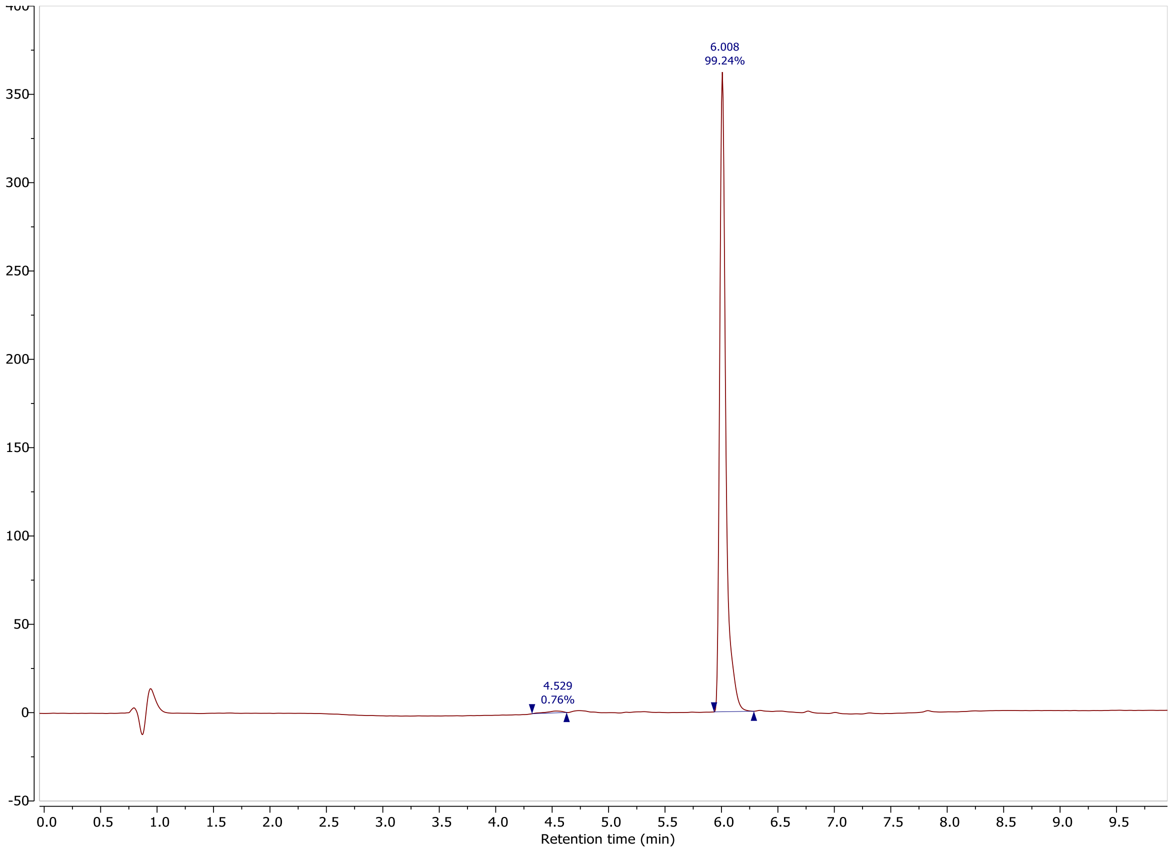
6.008
99.24%
4.529
0.76%
400
350
300
250
200
150
100
50
0
-50
0.0
0.5
1.0
1.5
2.0
2.5
3.0
3.5
4.0
4.5
5.0
5.5
6.0
6.5
7.0
7.5
8.0
8.5
9.0
9.5
Retention time (min)

6b

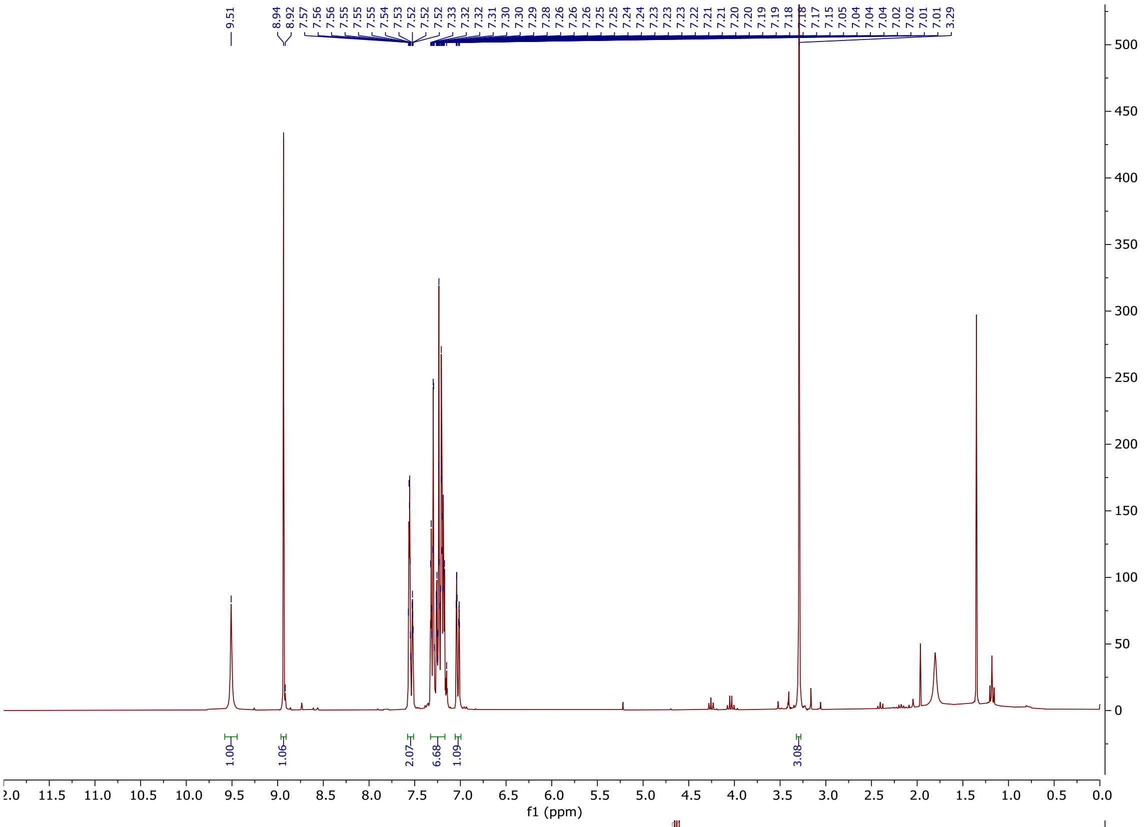
9.51
8.94
8.92
7.57
7.56
7.56
7.55
7.55
7.55
7.55
7.54
7.53
7.52
7.52
7.52
7.33
7.32
7.32
7.31
7.30
7.30
7.29
7.28
7.26
7.26
7.25
7.25
7.24
7.24
7.23
7.23
7.23
7.23
7.22
7.21
7.21
7.20
7.20
7.19
7.19
7.18
7.18
7.17
7.15
7.05
7.04
7.04
7.02
7.01
7.01
3.29
1.00
1.06
2.07
6.68
1.09
3.08
f1 (ppm)

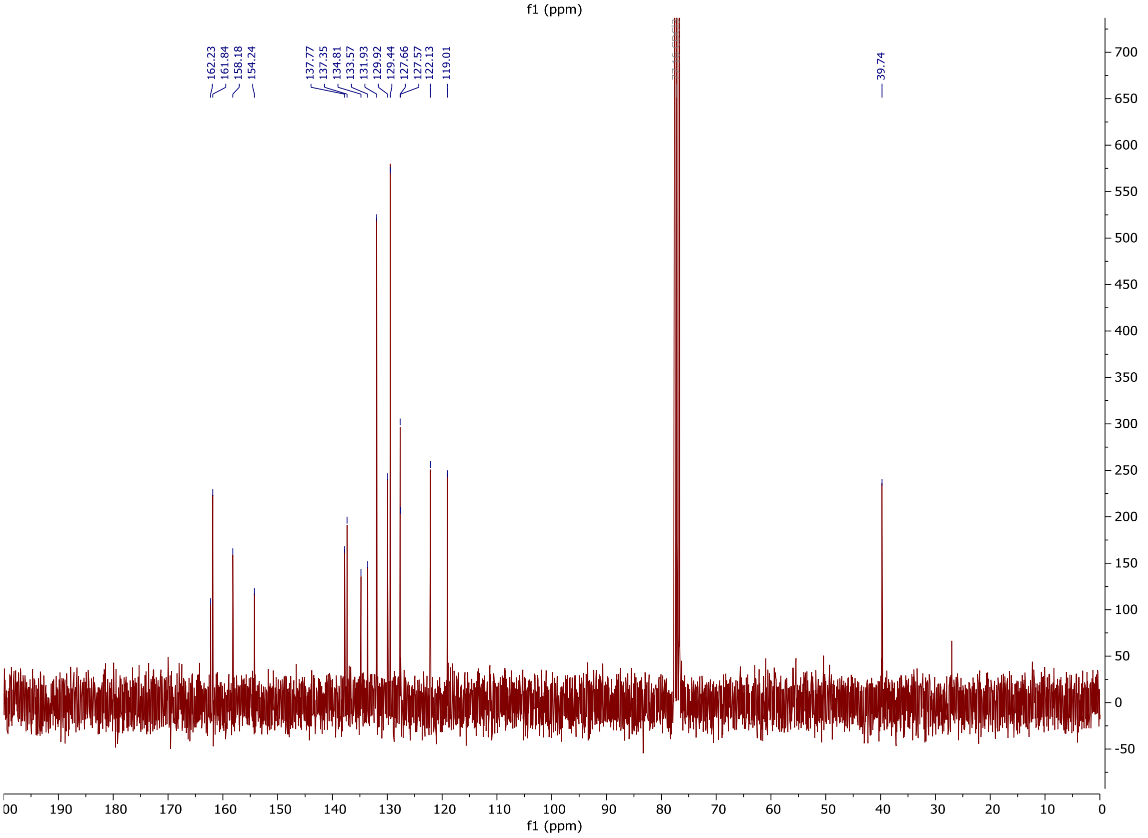
162.23
161.84
158.18
154.24
137.77
137.35
134.81
133.57
131.93
129.92
129.44
127.66
127.57
122.13
119.01
39.74
f1 (ppm)

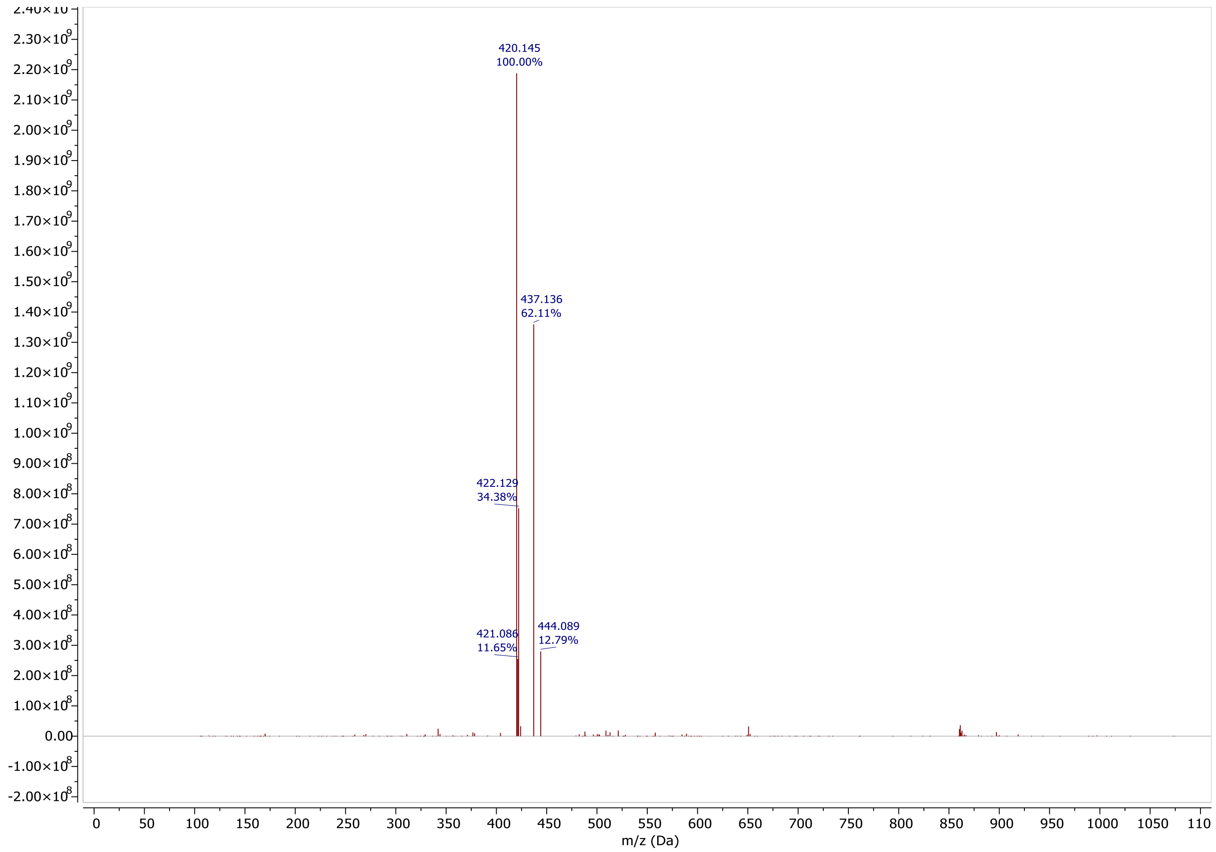
420.145
100.00%
437.136
62.11%
422.129
34.38%
421.086
11.65%
444.089
12.79%
m/z (Da)

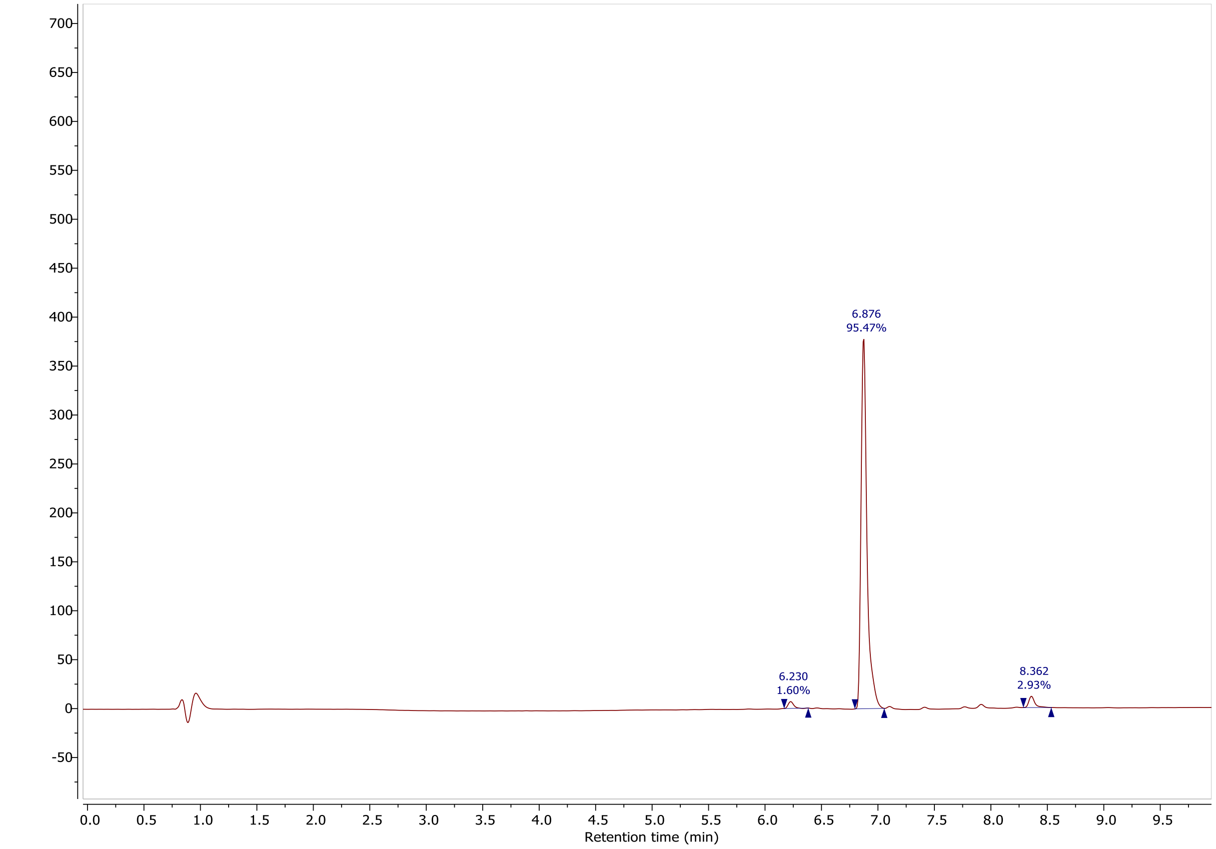
6.876
95.47%
6.230
1.60%
8.362
2.93%
Retention time (min)

6c

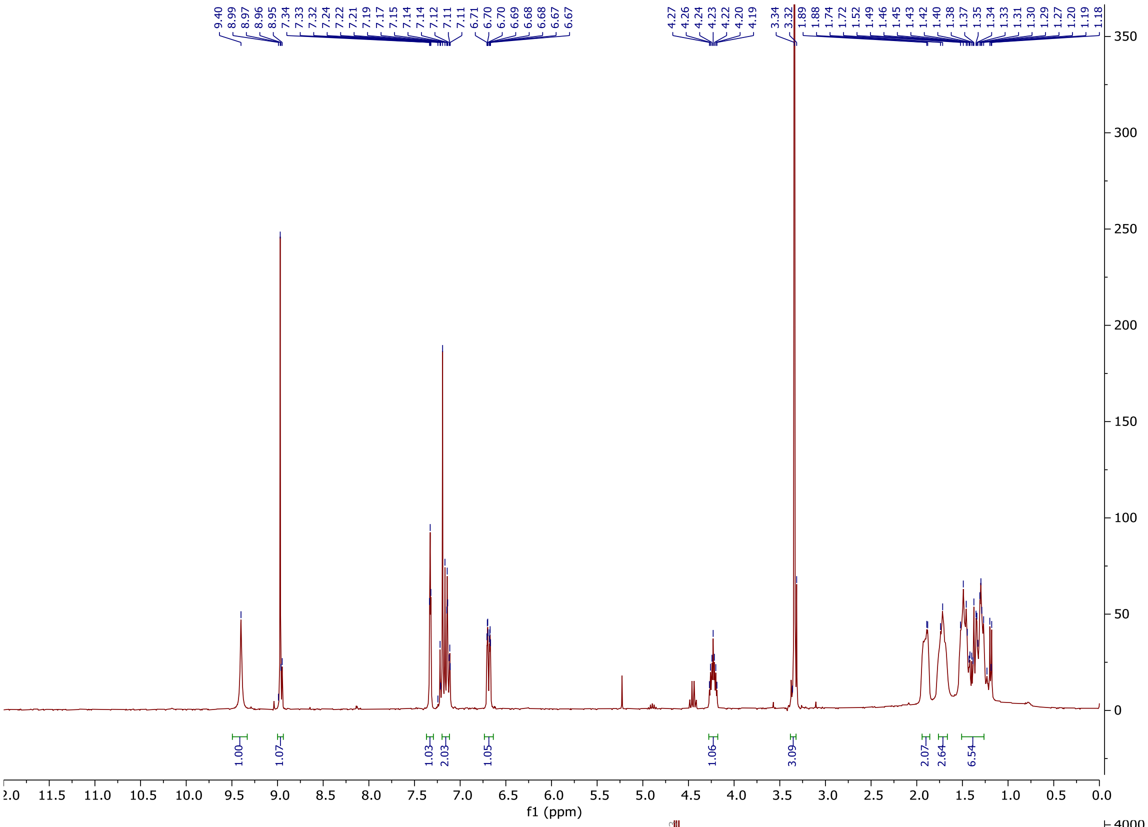
9.40
8.99
8.97
8.96
8.95
7.34
7.33
7.32
7.24
7.22
7.21
7.19
7.17
7.15
7.14
7.14
7.12
7.11
7.11
6.71
6.70
6.70
6.69
6.68
6.68
6.67
6.67
4.27
4.26
4.24
4.23
4.22
4.20
4.19
3.34
3.32
1.89
1.88
1.74
1.72
1.52
1.49
1.46
1.45
1.43
1.42
1.40
1.38
1.37
1.35
1.34
1.33
1.31
1.30
1.29
1.27
1.20
1.19
1.18
1.00
1.07
1.03
2.03
1.05
1.06
3.09
2.07
2.64
6.54
f1 (ppm)

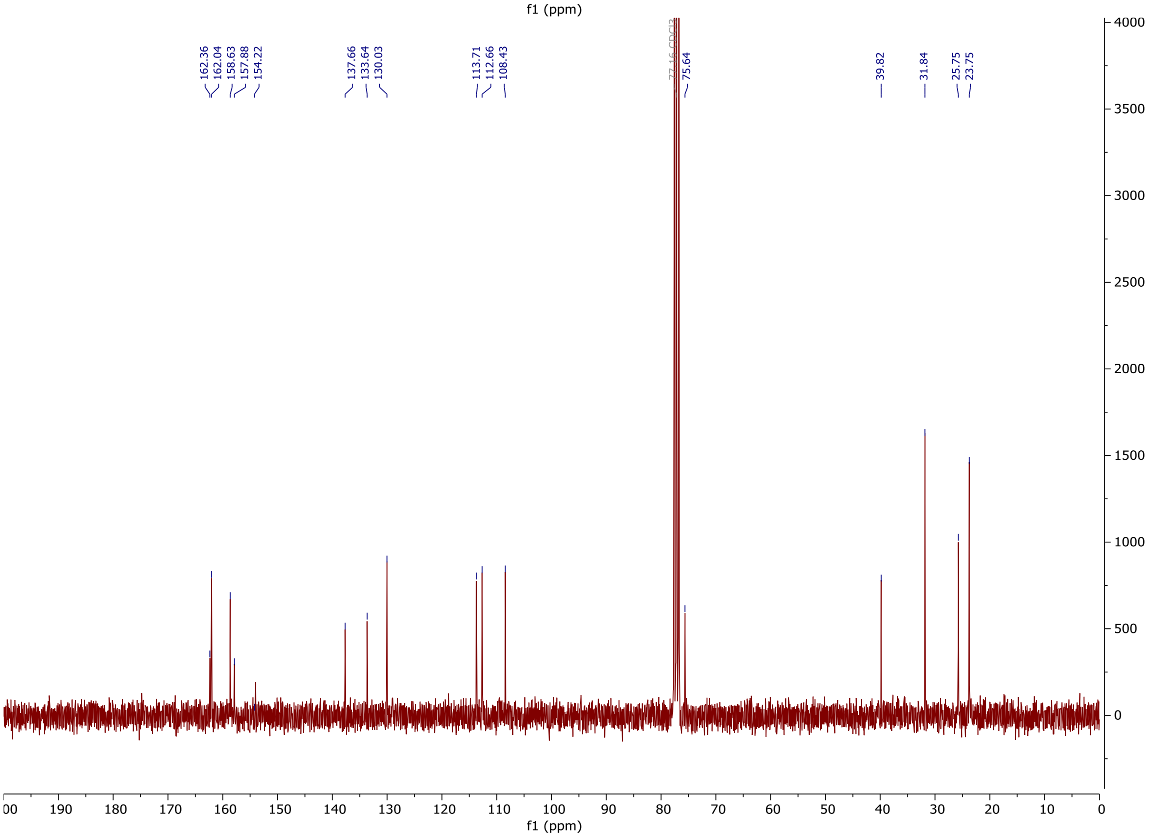
162.36
162.04
158.63
157.88
154.22
137.66
133.64
130.03
113.71
112.66
108.43
75.64
39.82
31.84
25.75
23.75
f1 (ppm)

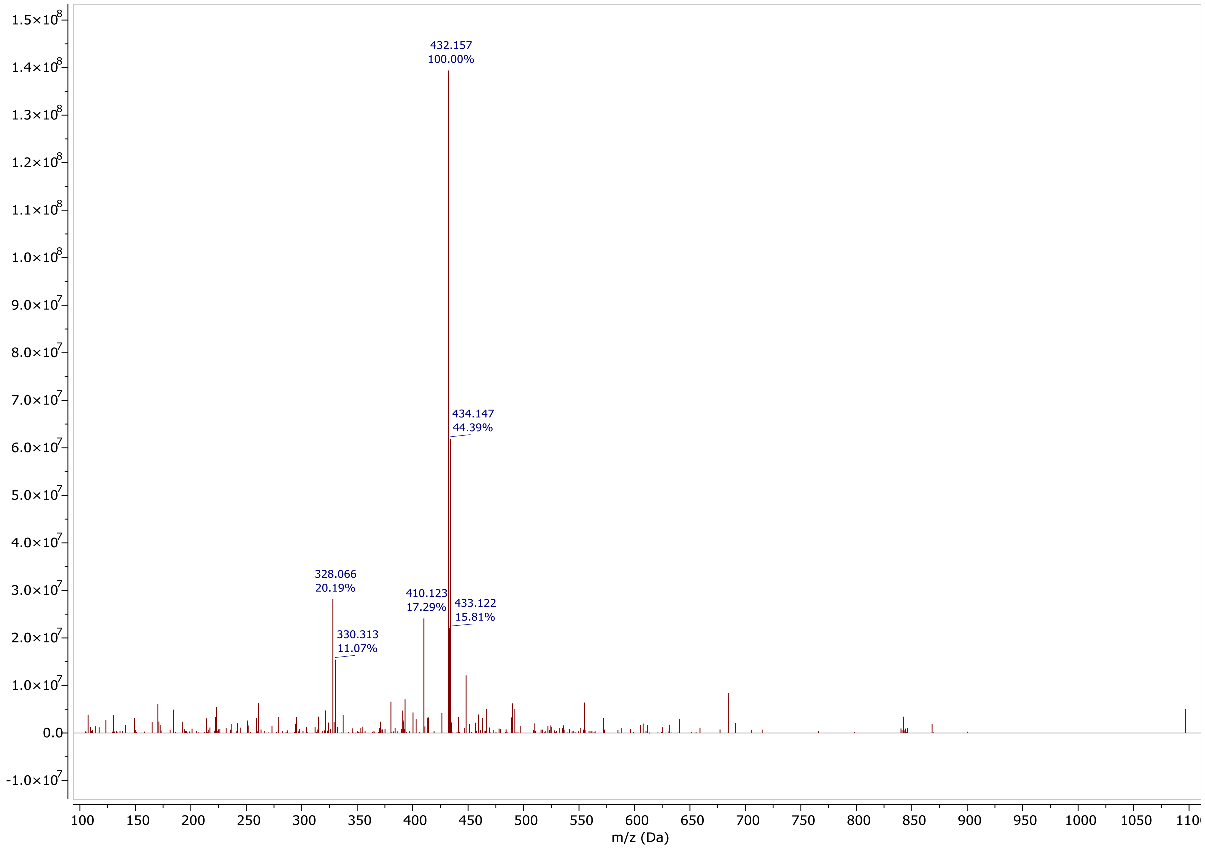
432.157
100.00%
434.147
44.39%
328.066
20.19%
410.123
17.29%
433.122
15.81%
330.313
11.07%
m/z (Da)

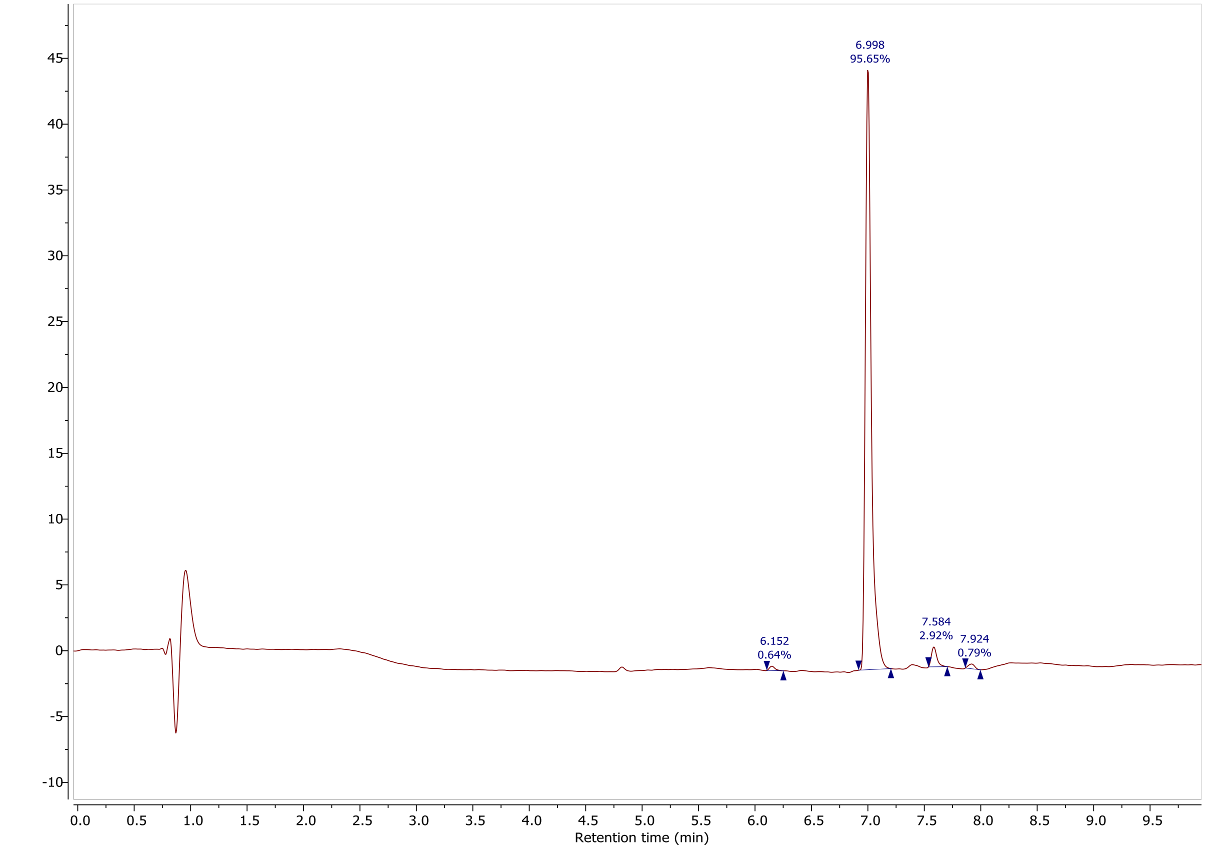
6.998
95.65%
6.152
0.64%
7.584
2.92%
7.924
0.79%
Retention time (min)

6d

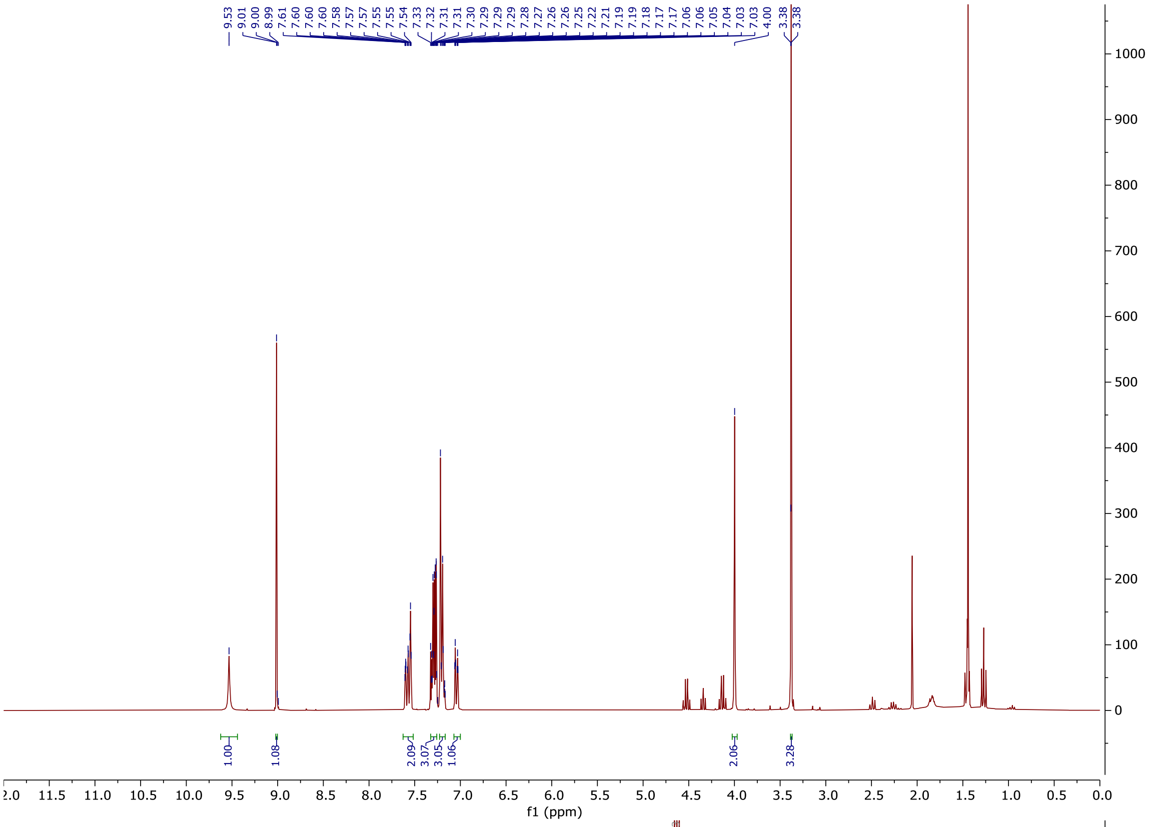
9.53
9.01
9.00
8.99
7.61
7.60
7.60
7.58
7.57
7.57
7.55
7.55
7.54
7.33
7.32
7.31
7.31
7.30
7.29
7.29
7.29
7.28
7.27
7.26
7.26
7.25
7.22
7.21
7.19
7.19
7.18
7.17
7.17
7.06
7.06
7.05
7.04
7.03
7.03
4.00
3.38
3.38
1.00
1.08
2.09
3.07
3.05
1.06
2.06
3.28
f1 (ppm)

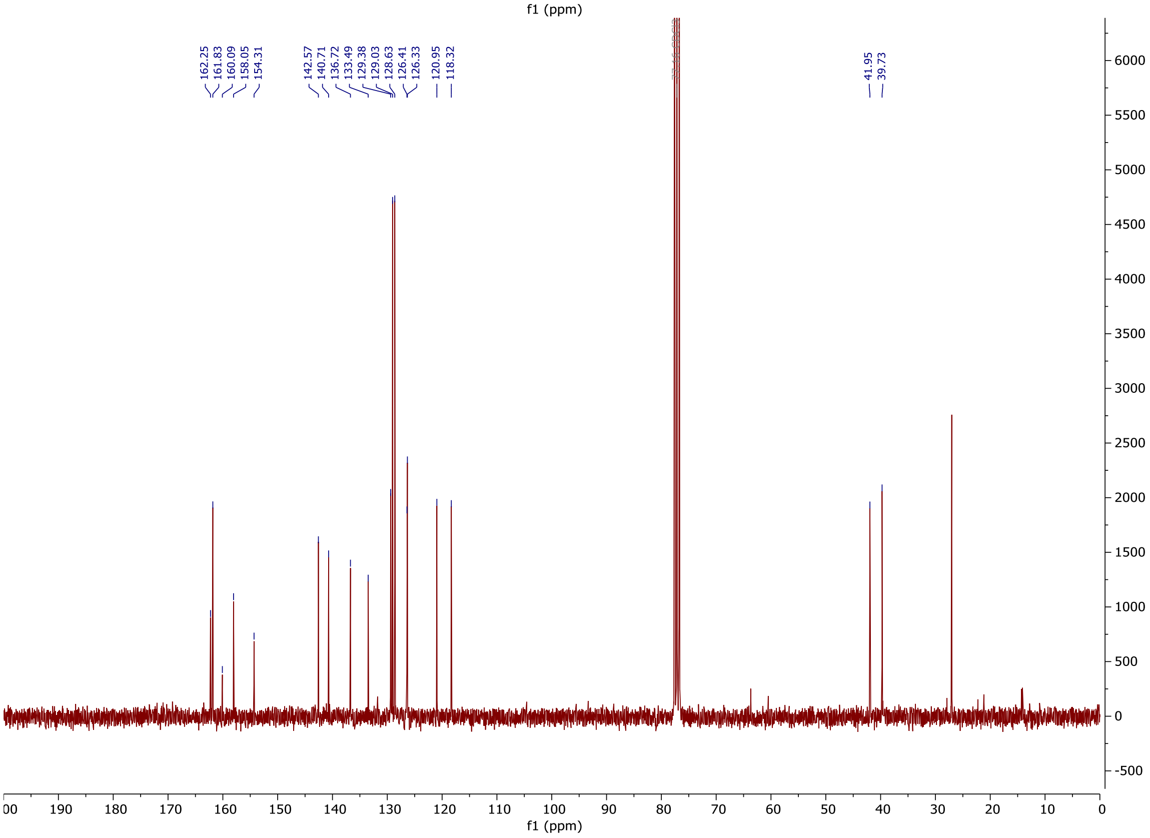
162.25
161.83
160.09
158.05
154.31
142.57
140.71
136.72
133.49
129.38
129.03
128.63
126.41
126.33
120.95
118.32
41.95
39.73
f1 (ppm)

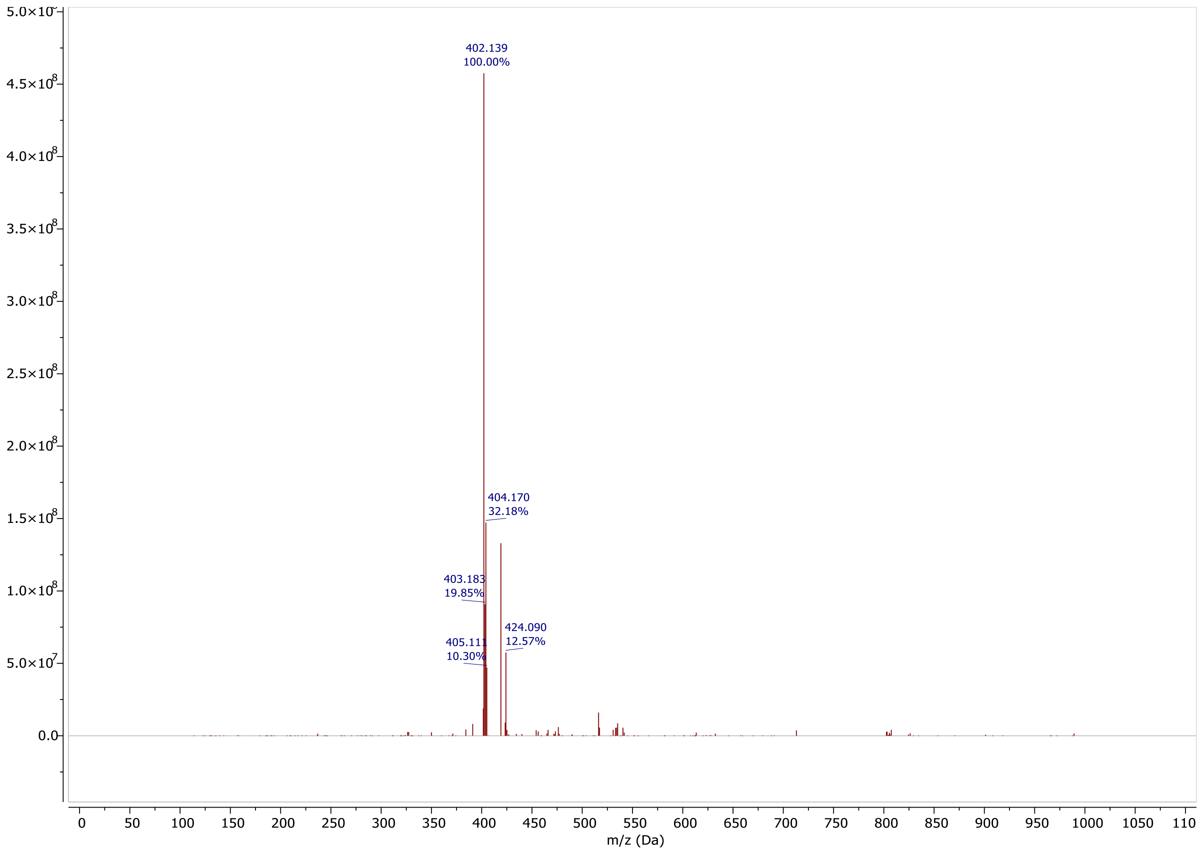
402.139
100.00%
404.170
32.18%
403.183
19.85%
424.090
12.57%
405.111
10.30%
m/z (Da)

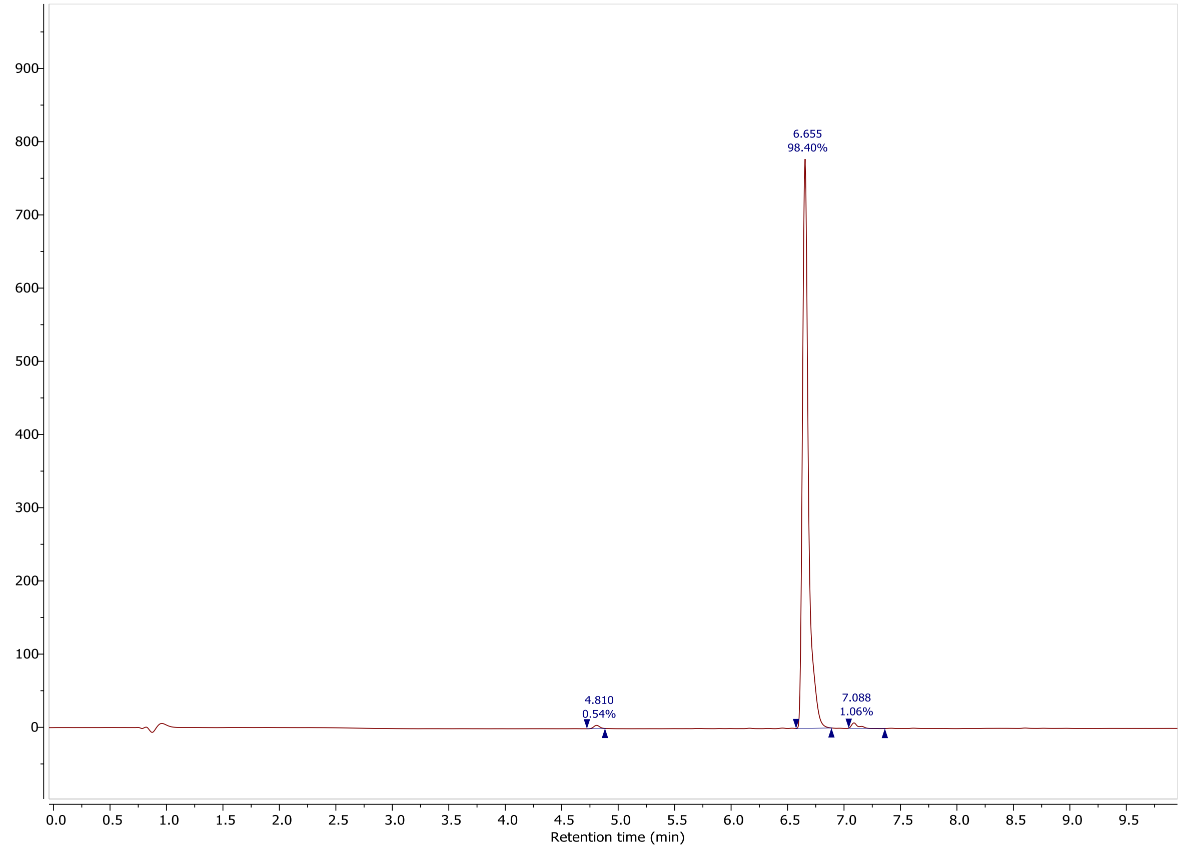
6.655
98.40%
4.810
0.54%
7.088
1.06%
Retention time (min)

6e

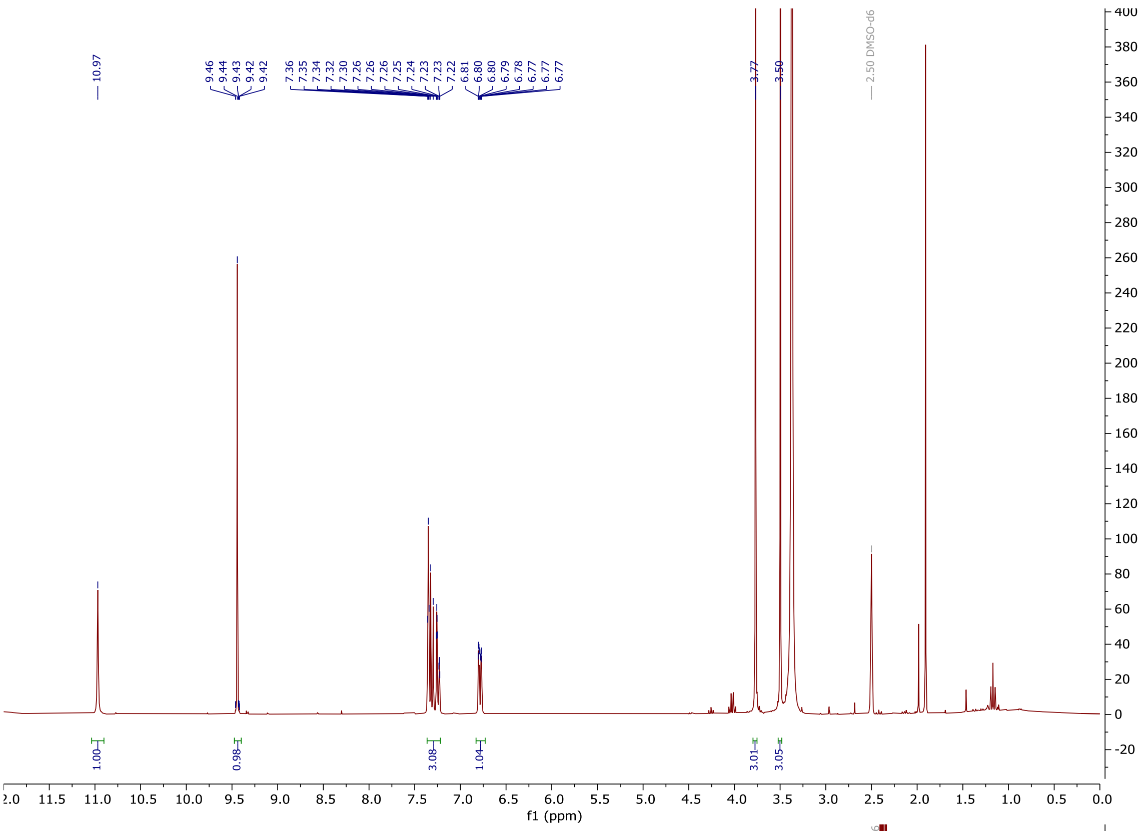
10.97
9.46
9.44
9.43
9.42
9.42
7.36
7.35
7.34
7.32
7.30
7.26
7.26
7.26
7.25
7.24
7.23
7.23
7.22
6.81
6.80
6.80
6.79
6.78
6.77
6.77
6.77
3.77
3.50
2.50 DMSO-d6
1.00
0.98
3.08
1.04
3.01
3.05
f1 (ppm)

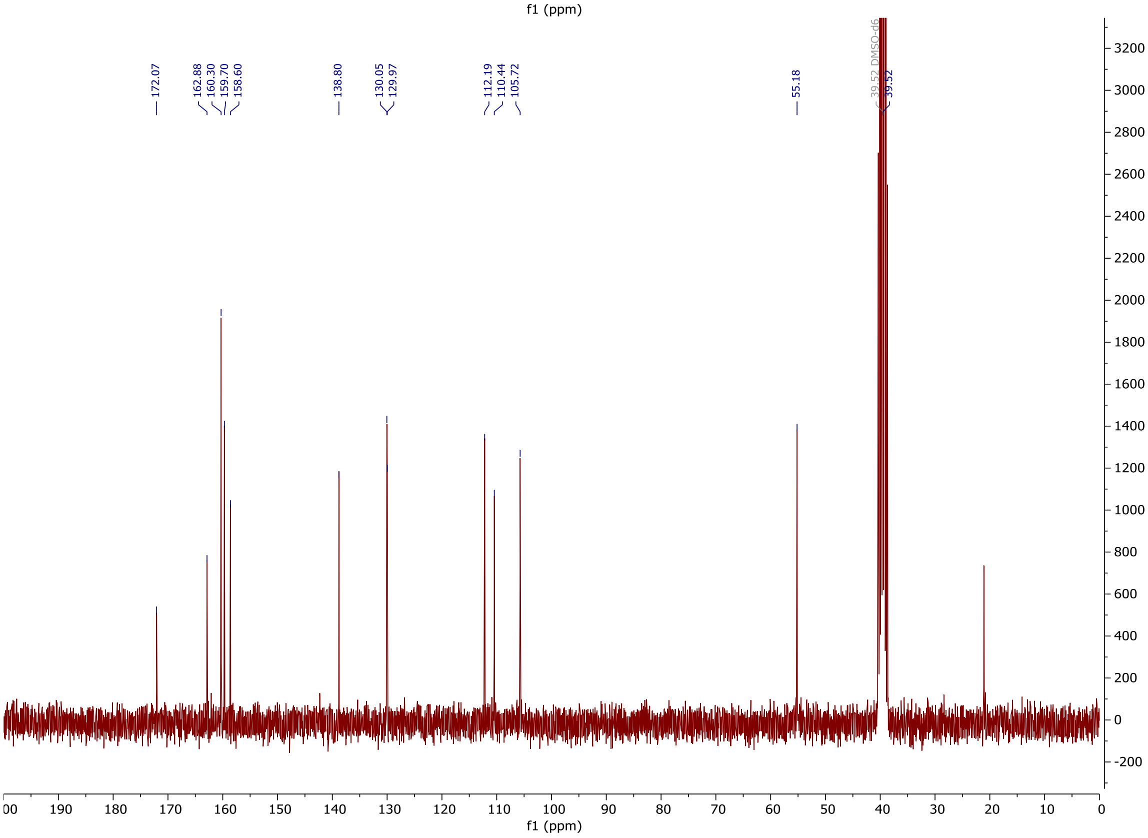
172.07
162.88
160.30
159.70
158.60
138.80
130.05
129.97
112.19
110.44
105.72
55.18
39.52 DMSO-d6
39.52
f1 (ppm)

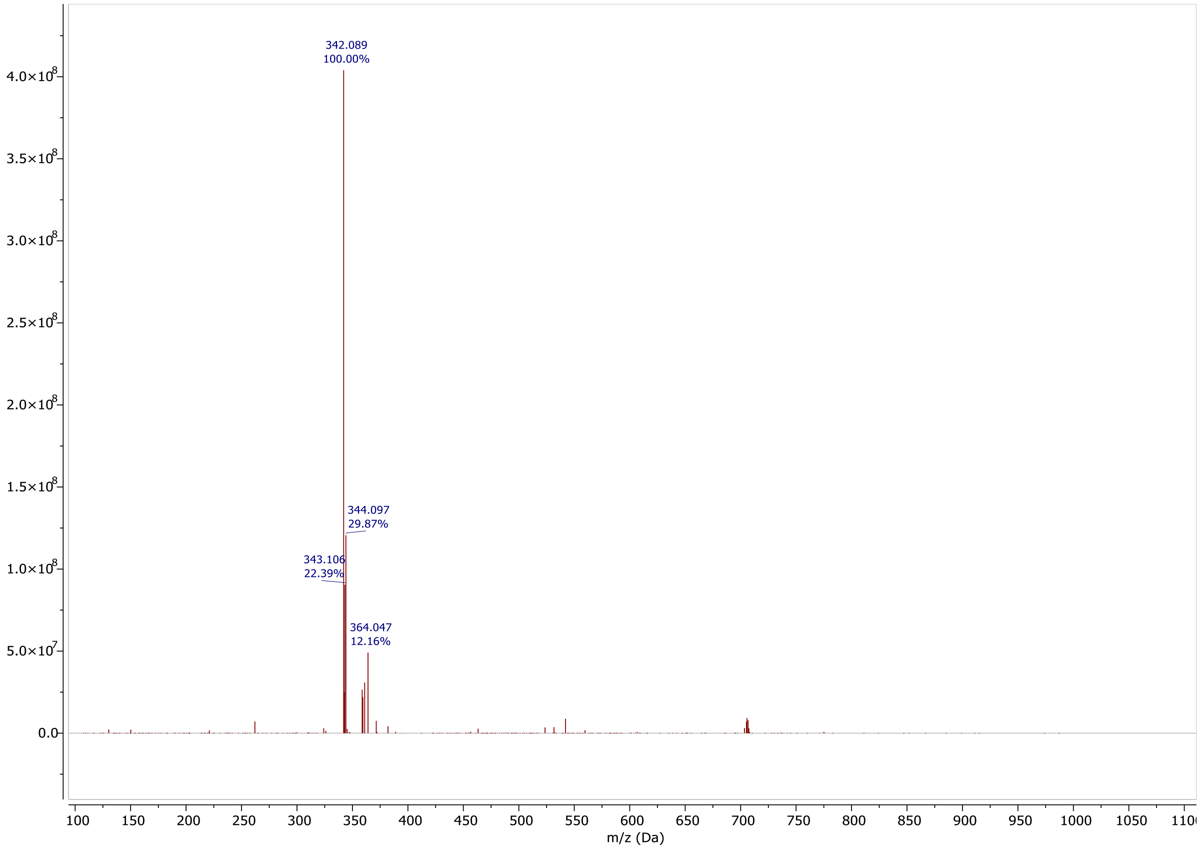

342.089
100.00%
344.097
29.87%
343.106
22.39%
364.047
12.16%
4.0×10^8
3.5×10^8
3.0×10^8
2.5×10^8
2.0×10^8
1.5×10^8
1.0×10^8
5.0×10^7
0.0
100 150 200 250 300 350 400 450 500 550 600 650 700 750 800 850 900 950 1000 1050 1100
m/z (Da)

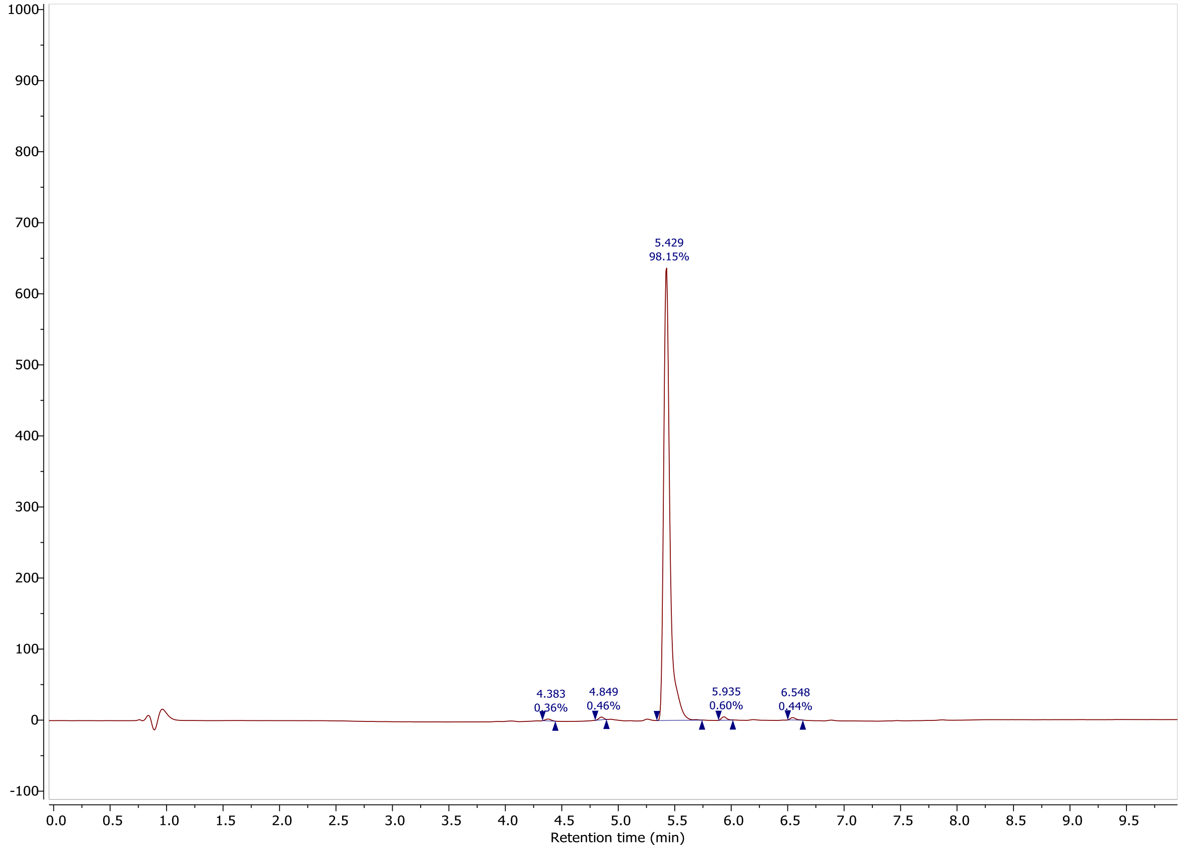

5.429
98.15%
4.383
0.36%
4.849
0.46%
5.935
0.60%
6.548
0.44%
1000
900
800
700
600
500
400
300
200
100
0
-100
0.0 0.5 1.0 1.5 2.0 2.5 3.0 3.5 4.0 4.5 5.0 5.5 6.0 6.5 7.0 7.5 8.0 8.5 9.0 9.5
Retention time (min)

6f

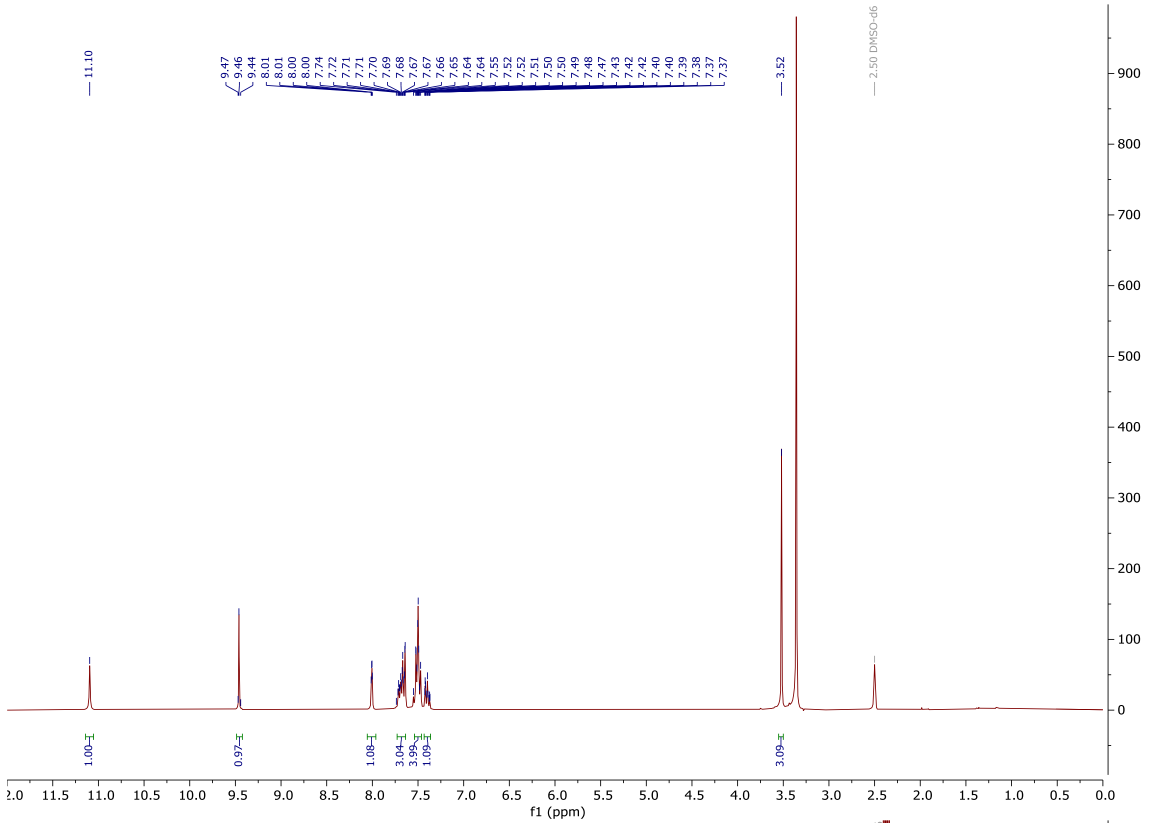

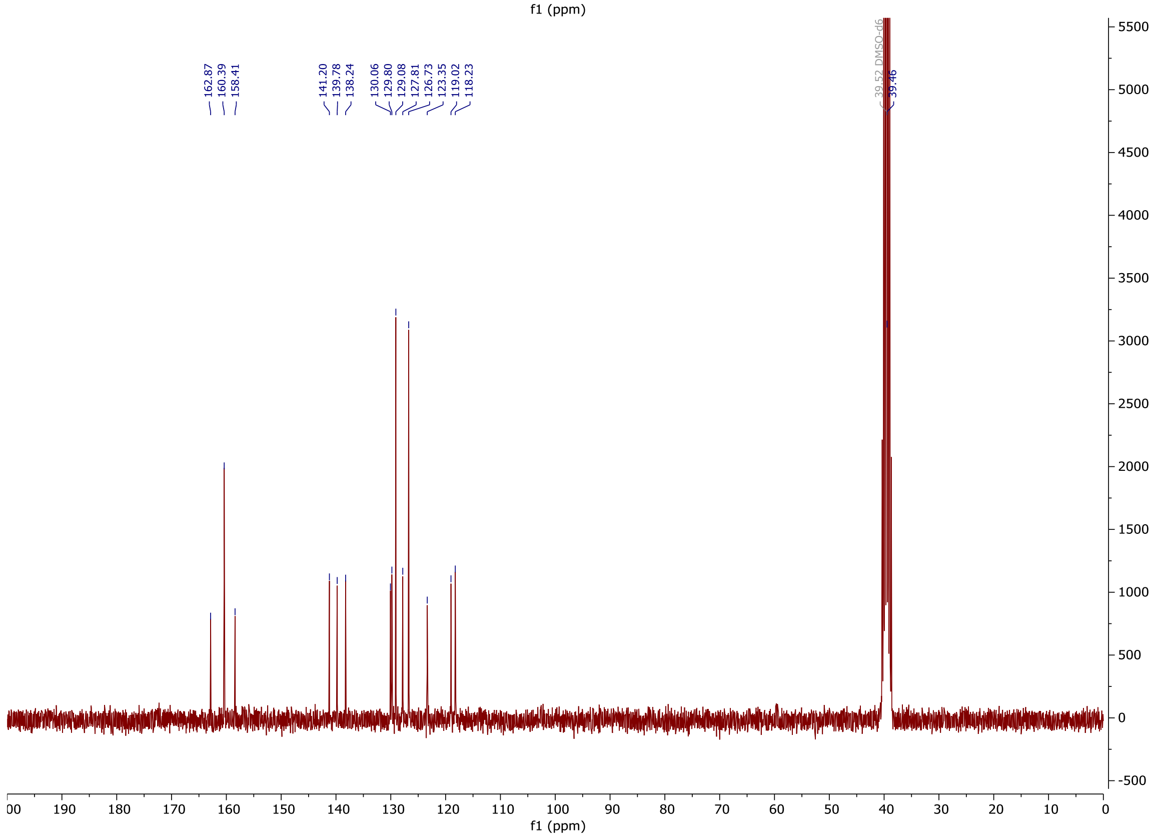

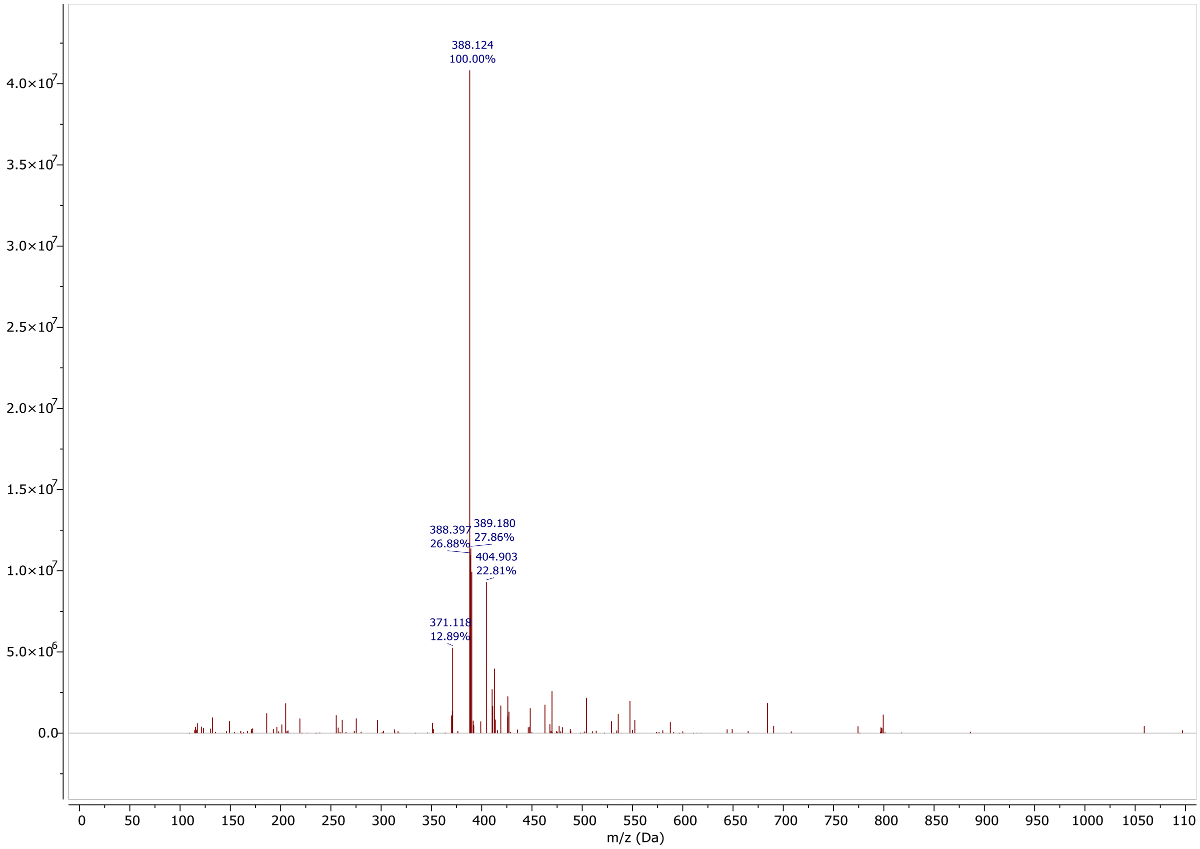
388.124
100.00%
388.397
26.88%
389.180
27.86%
404.903
22.81%
371.118
12.89%
m/z (Da)

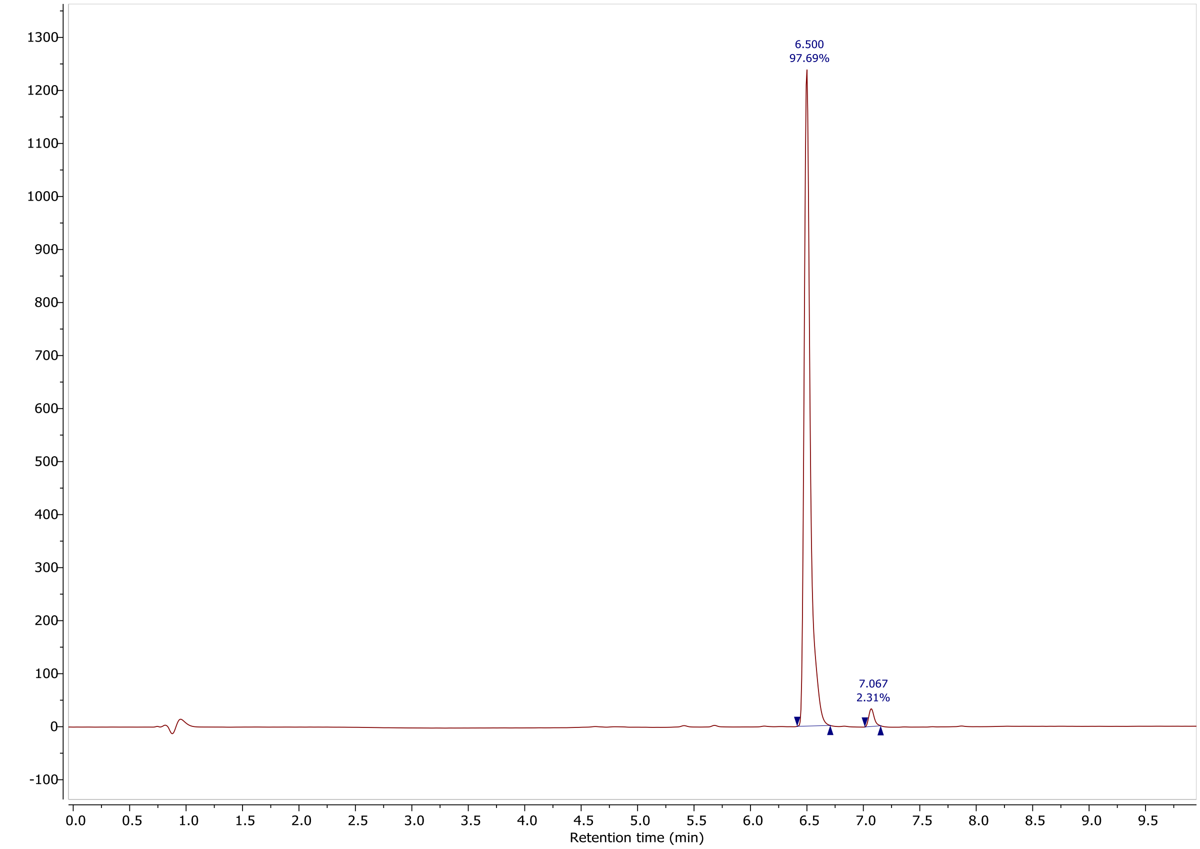
6.500
97.69%
7.067
2.31%
Retention time (min)

6g

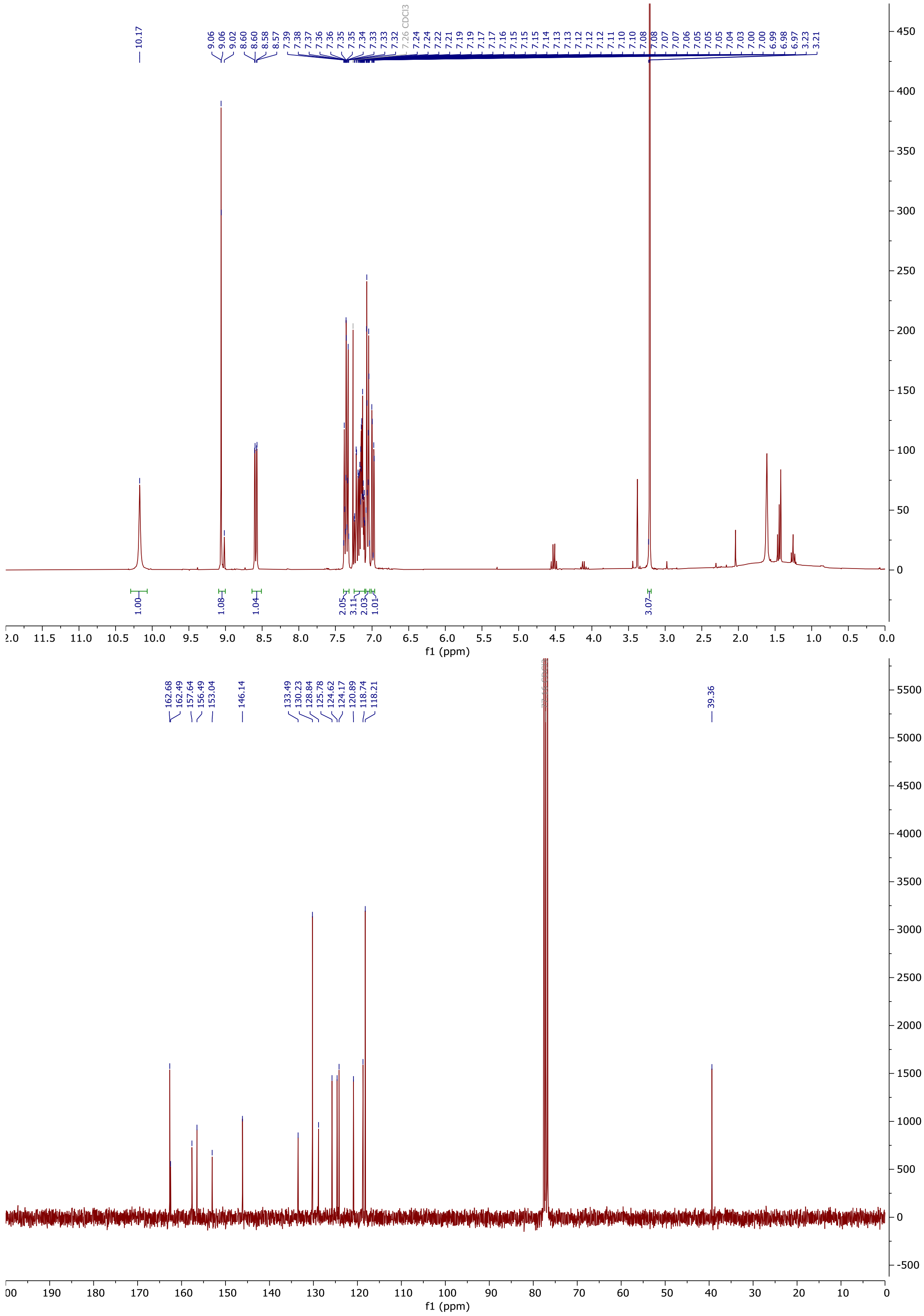

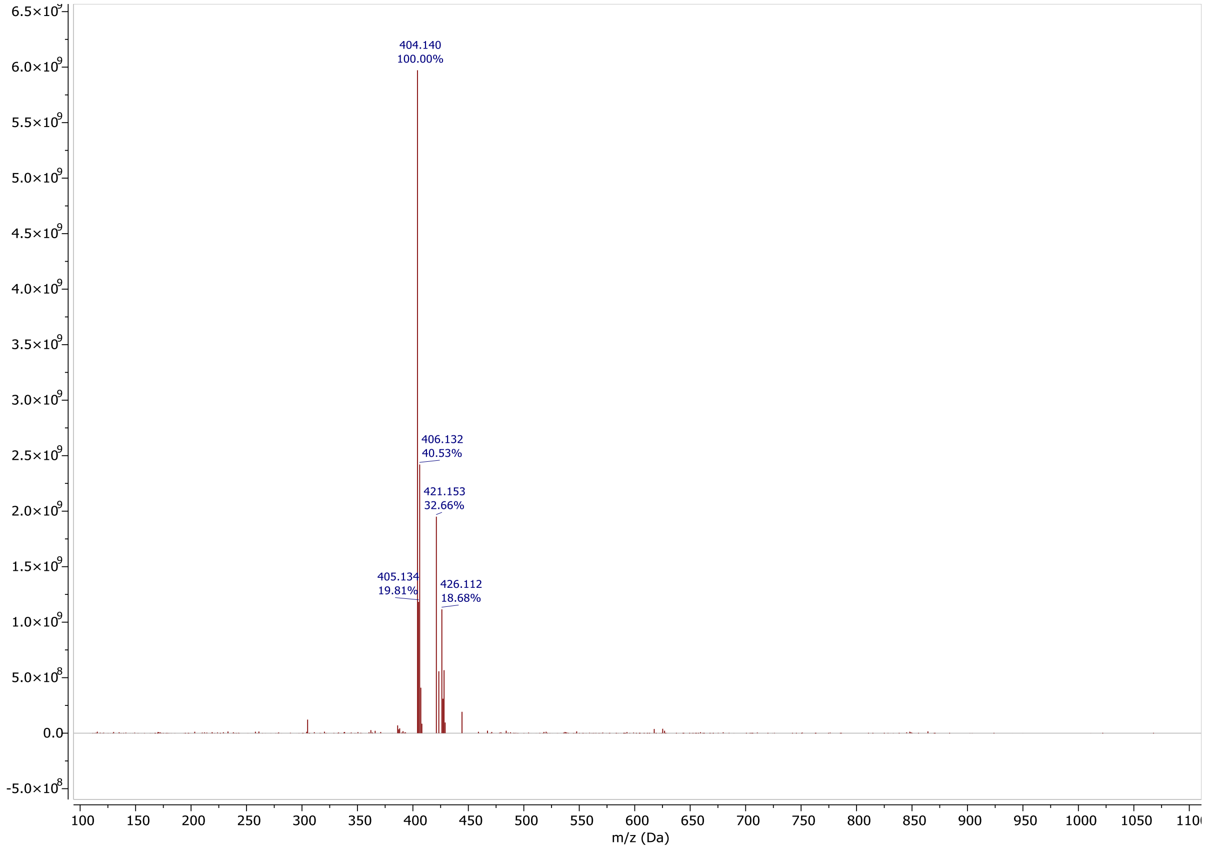
404.140
100.00%
406.132
40.53%
421.153
32.66%
405.134
19.81%
426.112
18.68%
m/z (Da)

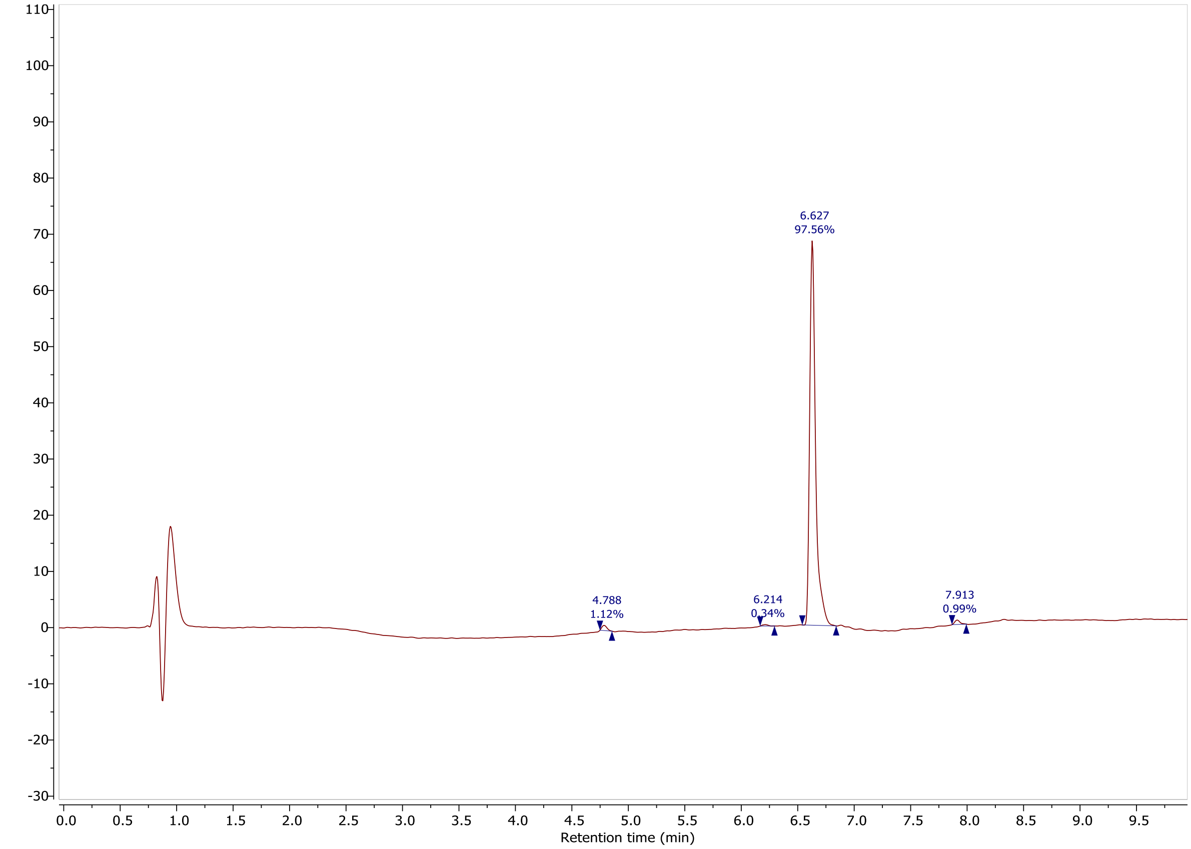
6.627
97.56%
4.788
1.12%
6.214
0.34%
7.913
0.99%
Retention time (min)

6h

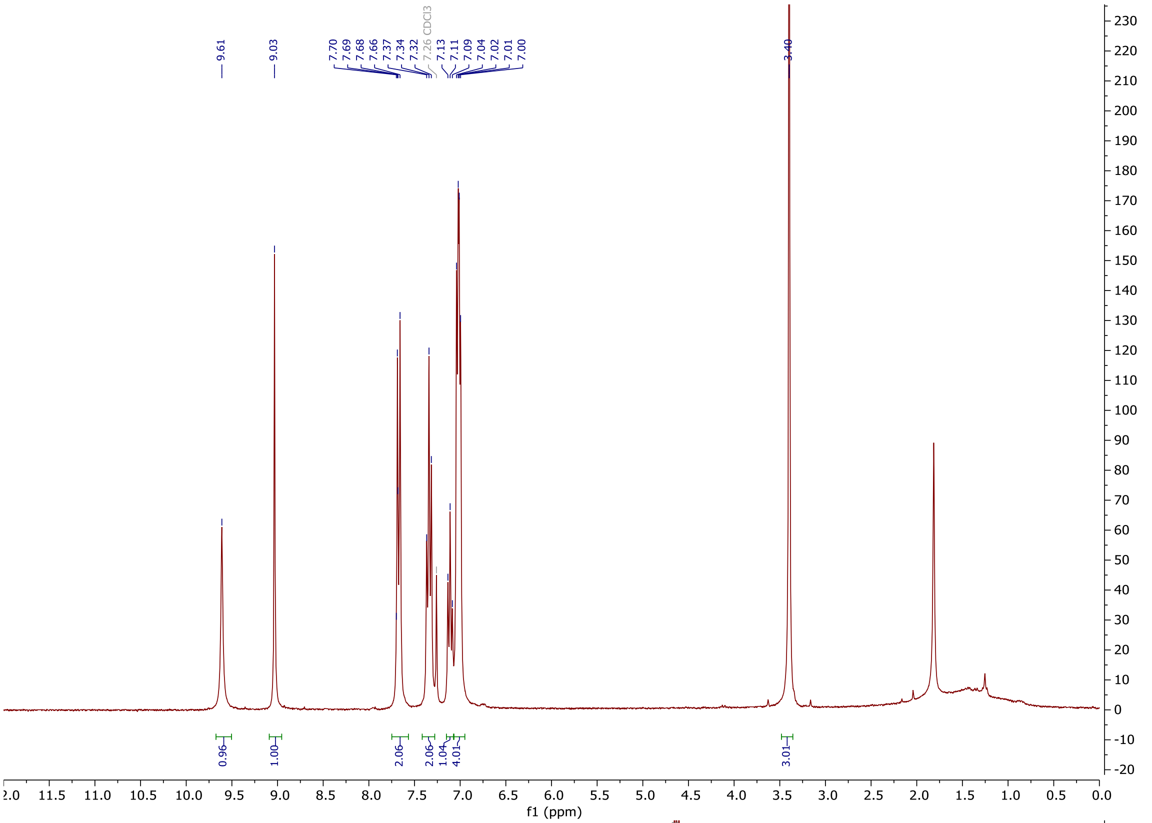

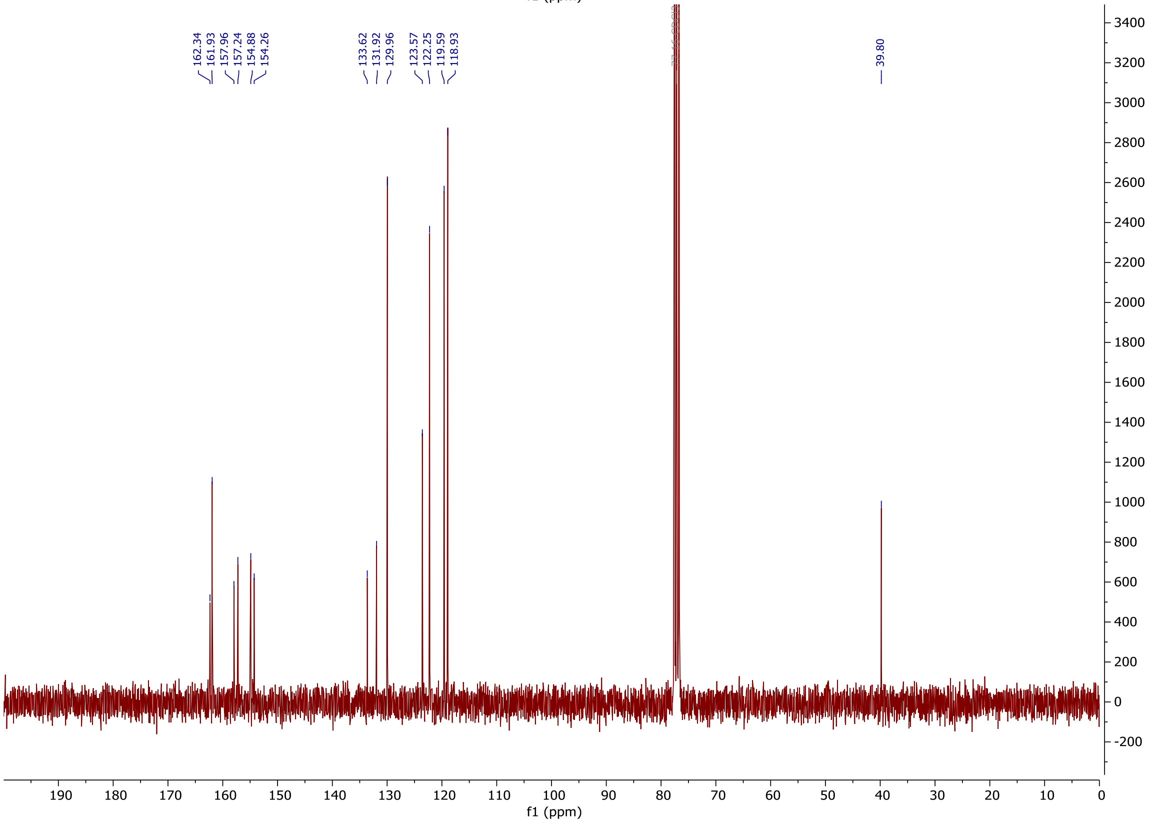

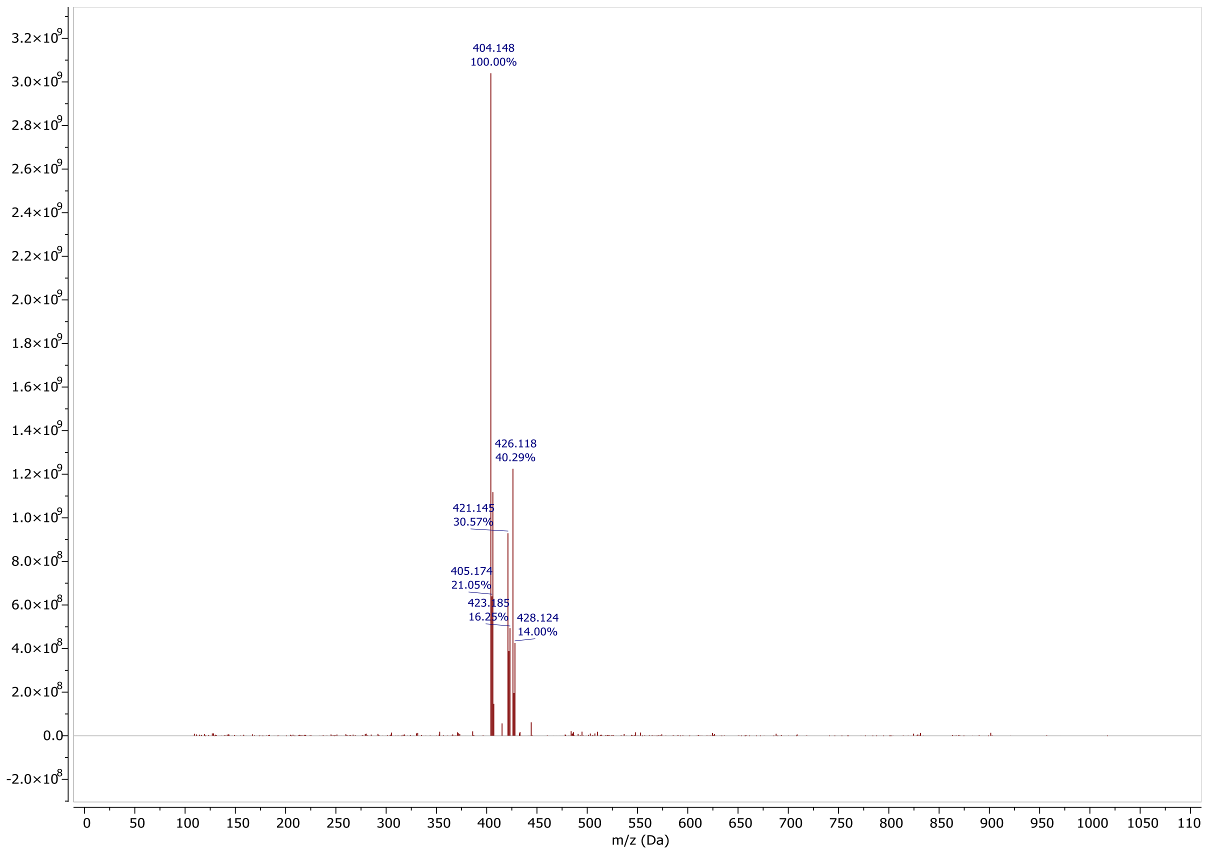
404.148
100.00%
426.118
40.29%
421.145
30.57%
405.174
21.05%
423.185
16.25%
428.124
14.00%
m/z (Da)

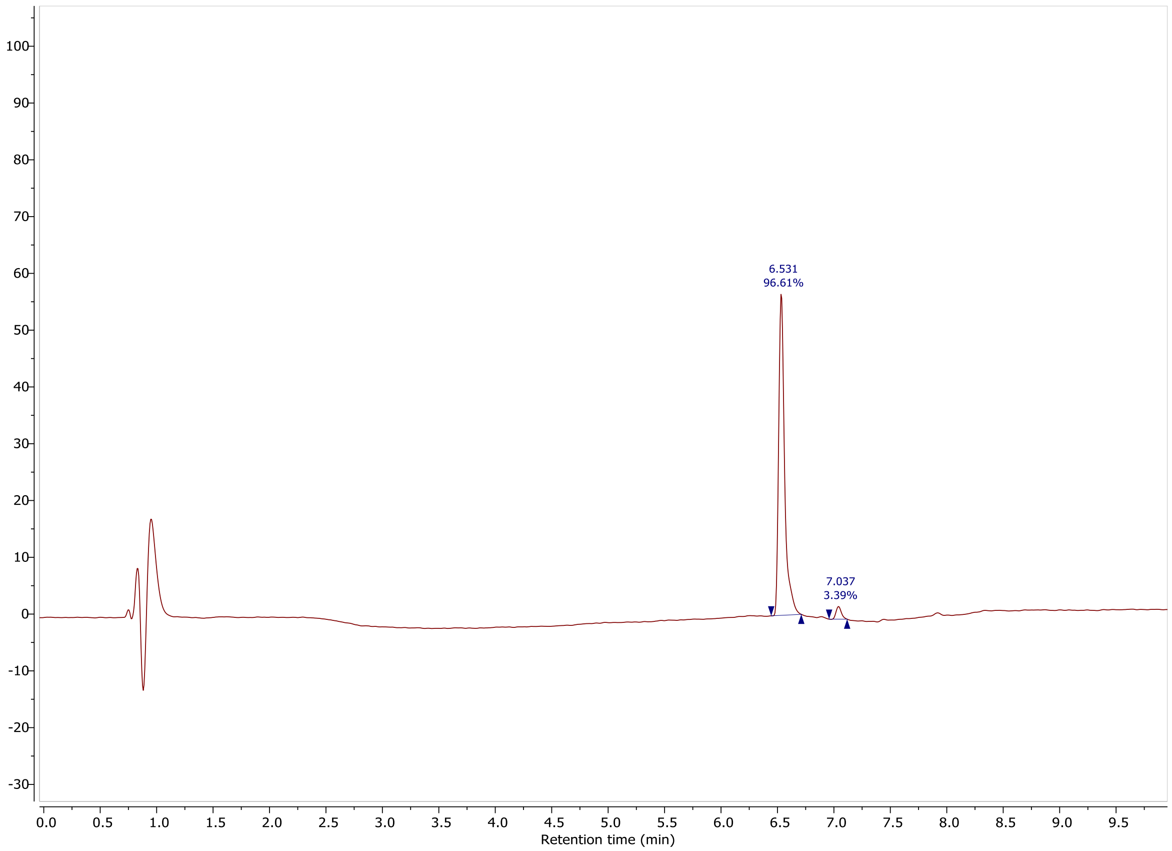
6.531
96.61%
7.037
3.39%
Retention time (min)

6i

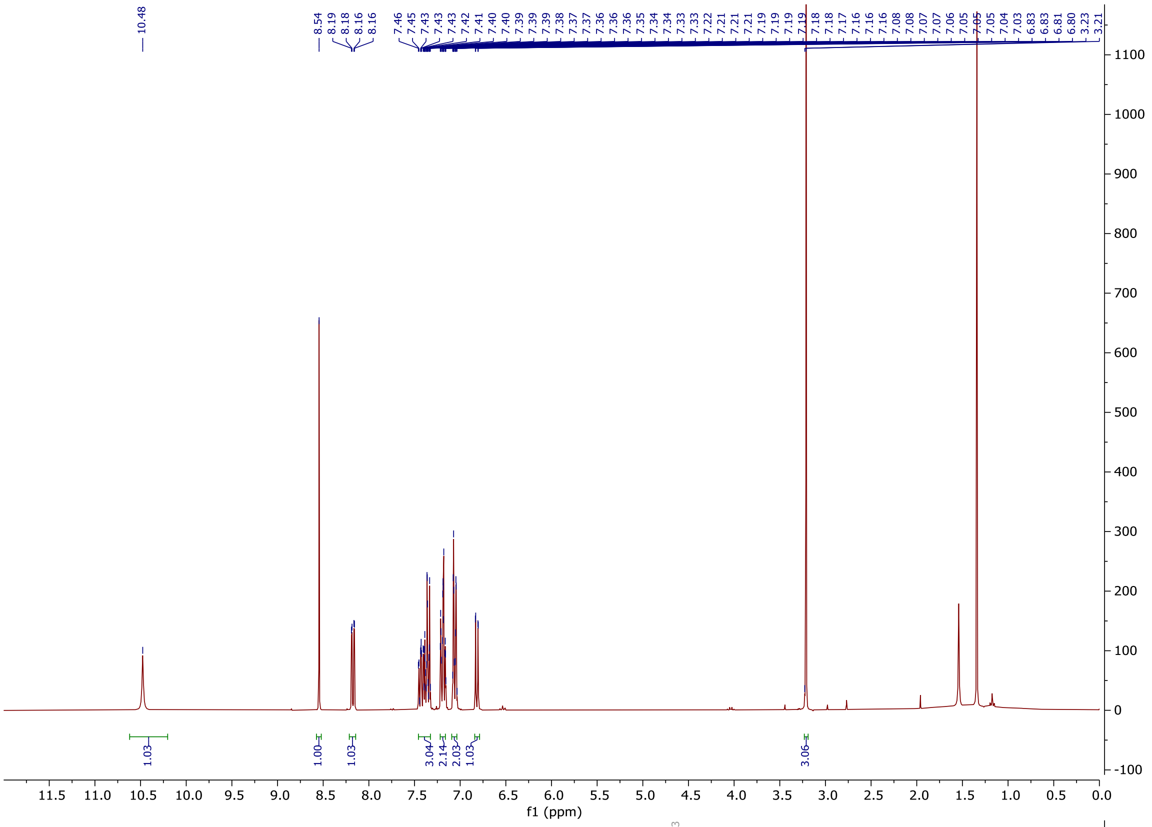

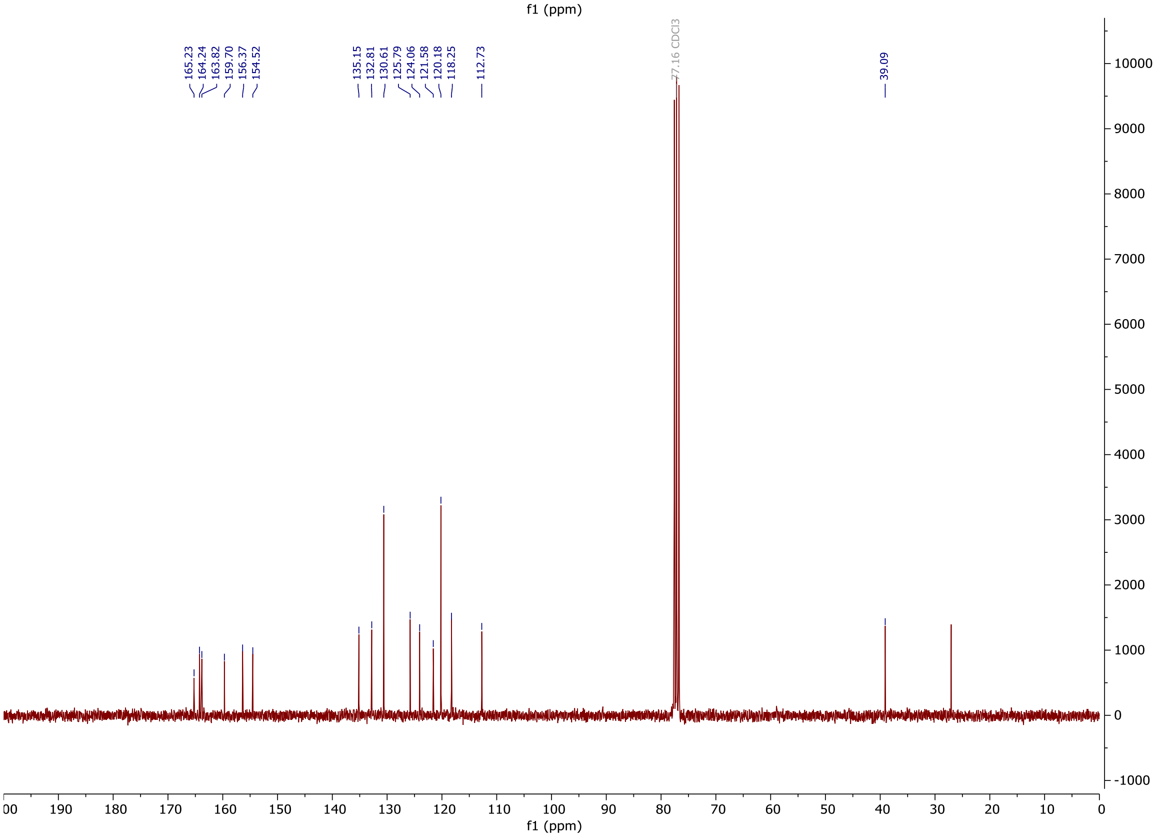

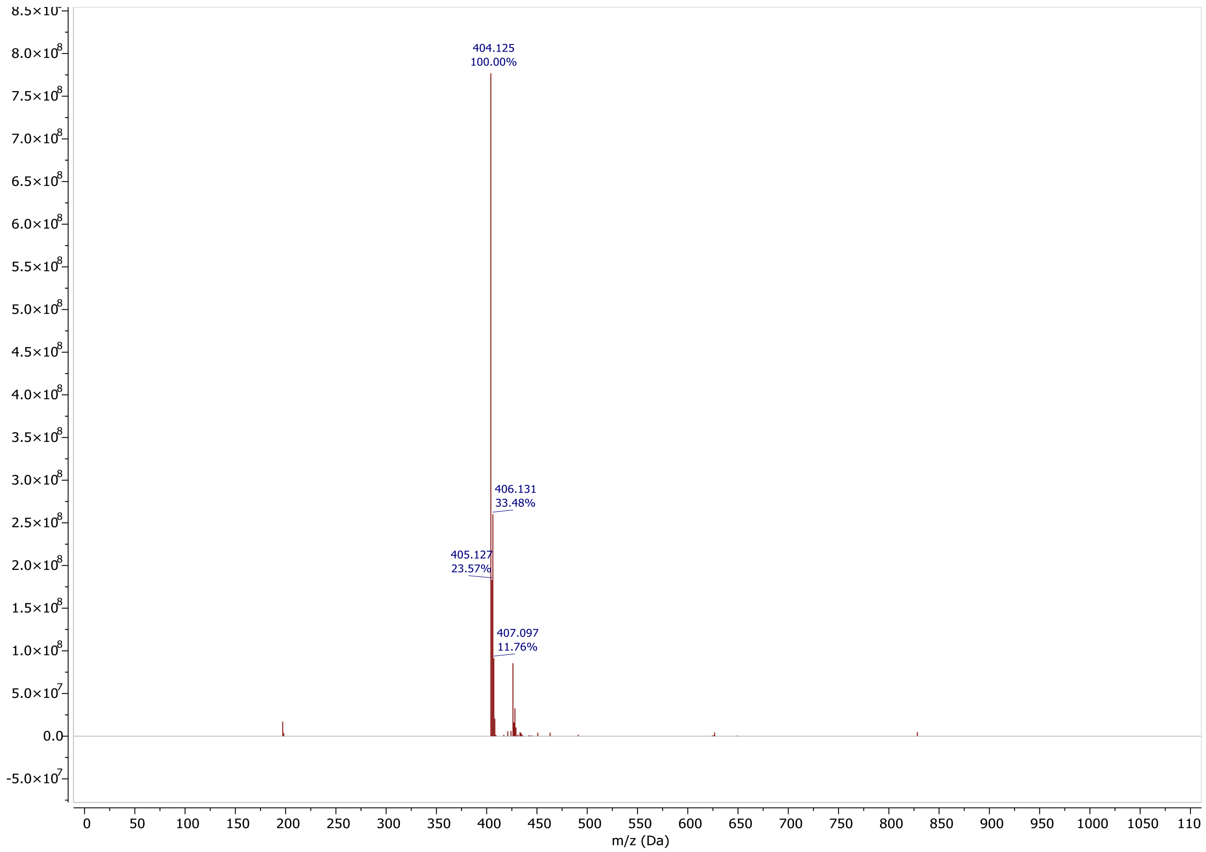
404.125
100.00%
406.131
33.48%
405.127
23.57%
407.097
11.76%
m/z (Da)

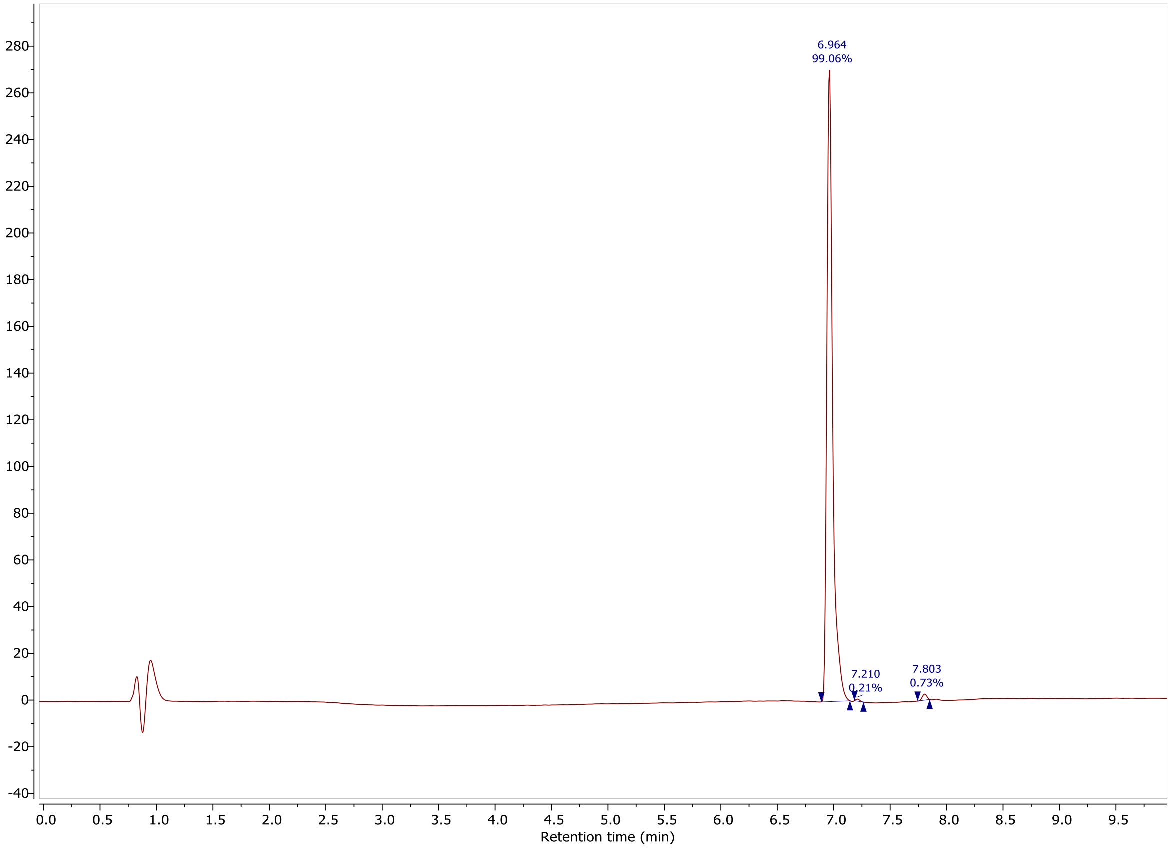
6.964
99.06%
7.210
0.21%
7.803
0.73%
Retention time (min)

6k

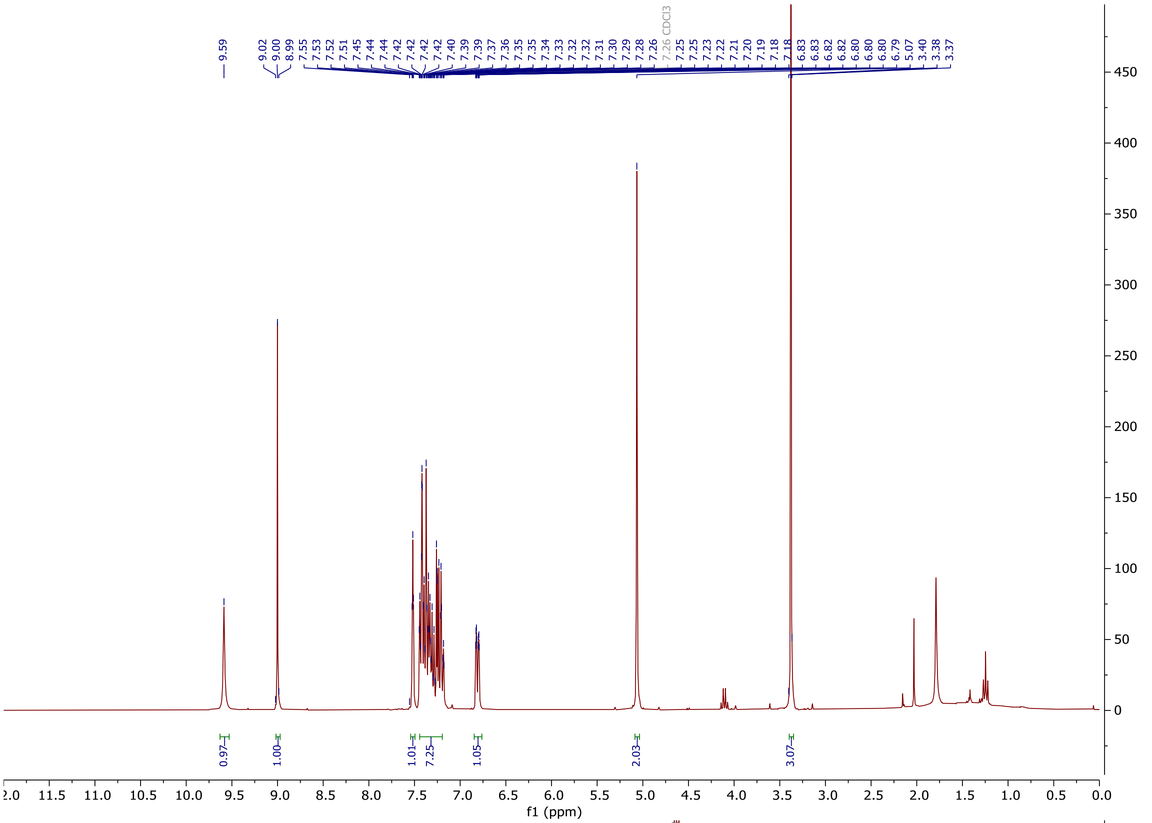
9.59
9.02
9.00
8.99
7.55
7.53
7.52
7.51
7.45
7.44
7.42
7.42
7.42
7.42
7.40
7.39
7.39
7.37
7.36
7.35
7.35
7.34
7.33
7.32
7.32
7.31
7.30
7.29
7.28
7.26
7.26 CDCl3
7.25
7.25
7.23
7.22
7.21
7.20
7.19
7.18
7.18
6.83
6.83
6.82
6.82
6.80
6.80
6.80
6.79
5.07
3.40
3.38
3.37
0.97
1.00
1.01
7.25
1.05
2.03
3.07
f1 (ppm)

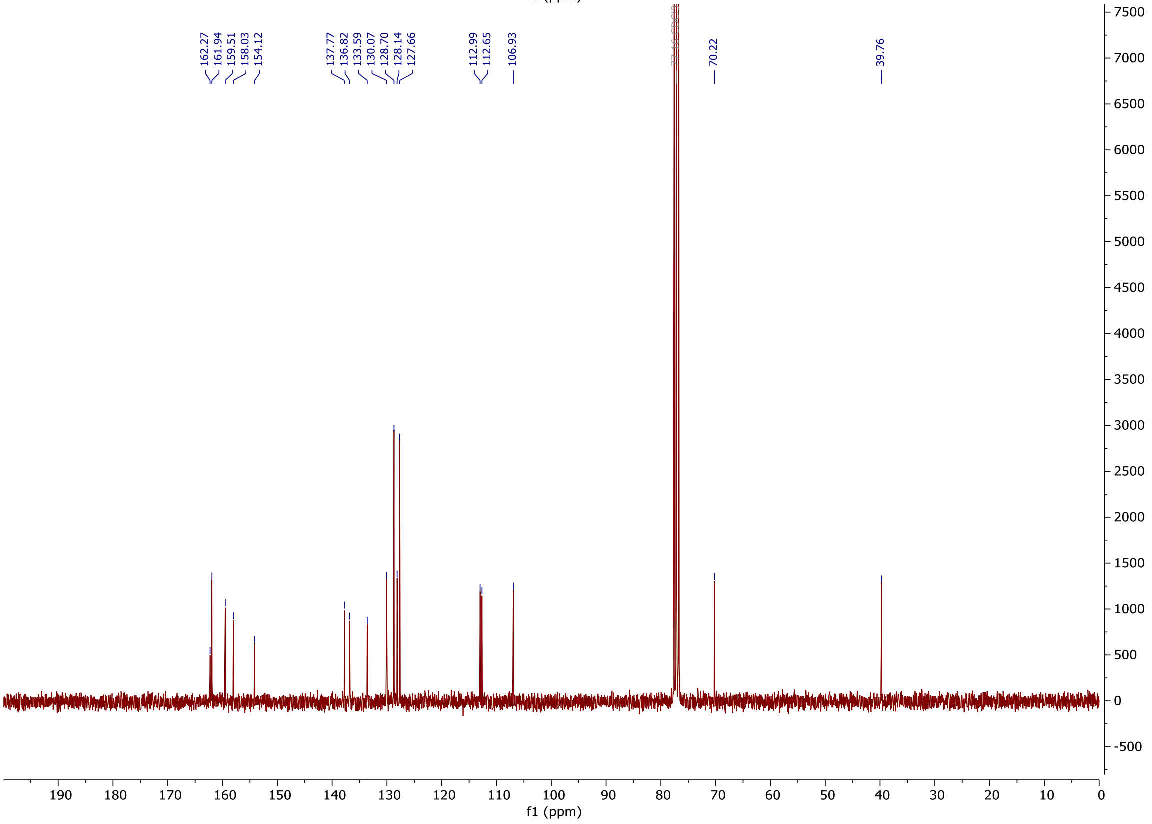
162.27
161.94
159.51
158.03
154.12
137.77
136.82
133.59
130.07
128.70
128.14
127.66
112.99
112.65
106.93
70.22
39.76
f1 (ppm)

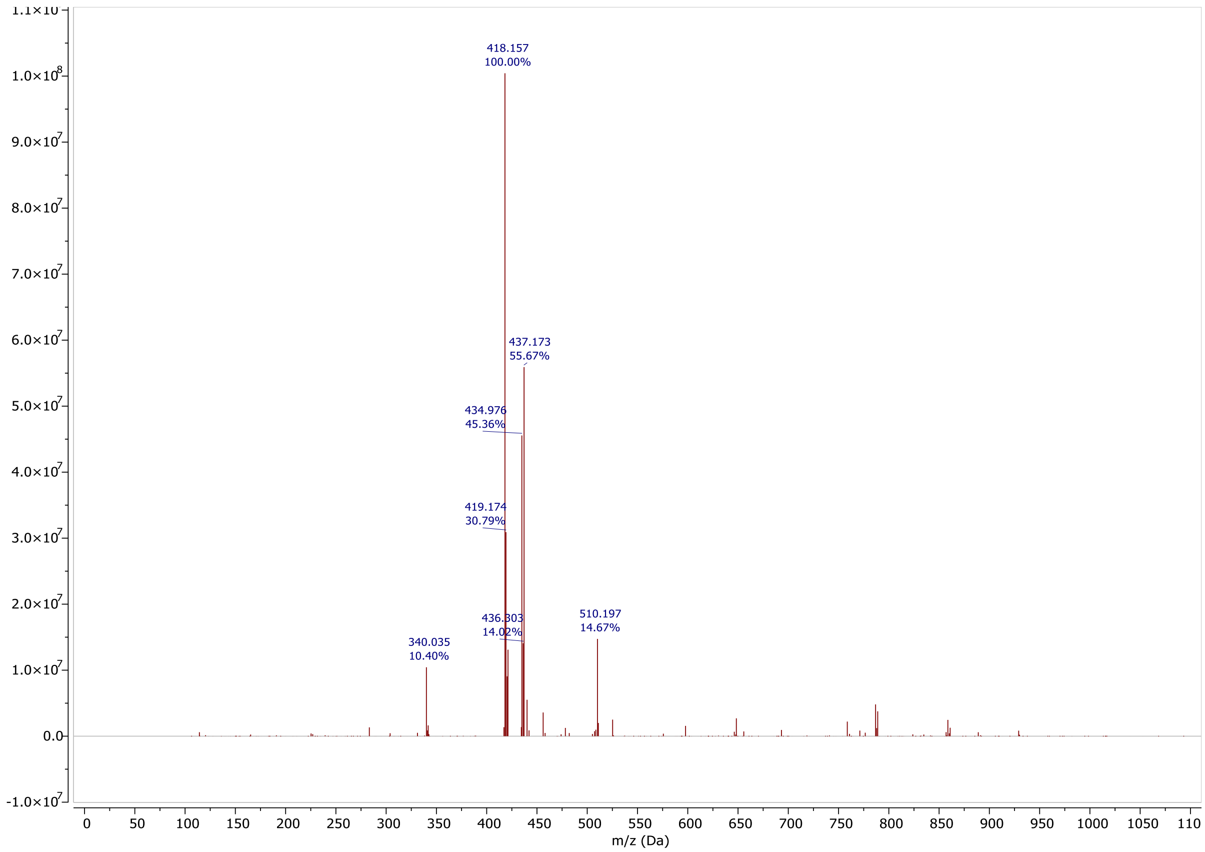
418.157
100.00%
437.173
55.67%
434.976
45.36%
419.174
30.79%
436.303
14.02%
510.197
14.67%
340.035
10.40%
m/z (Da)

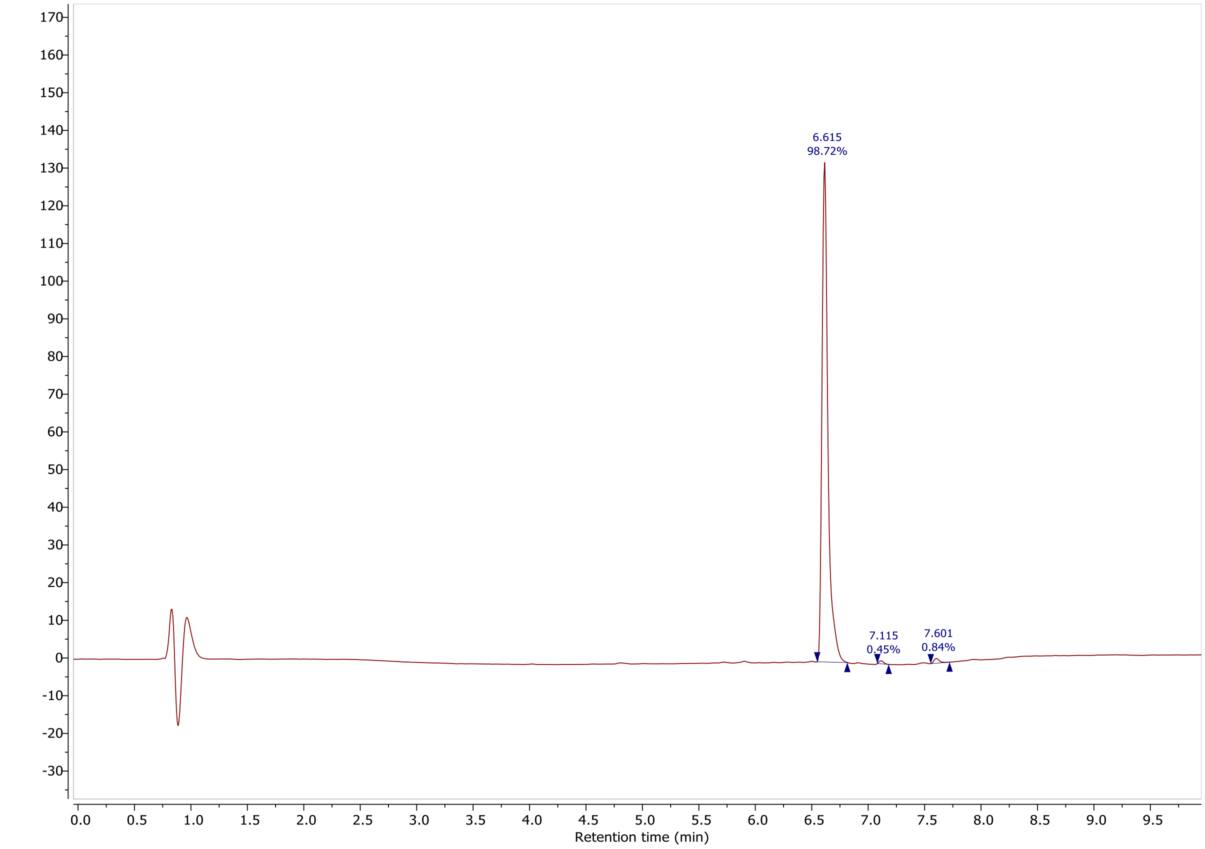
6.615
98.72%
7.115
0.45%
7.601
0.84%
Retention time (min)

6l

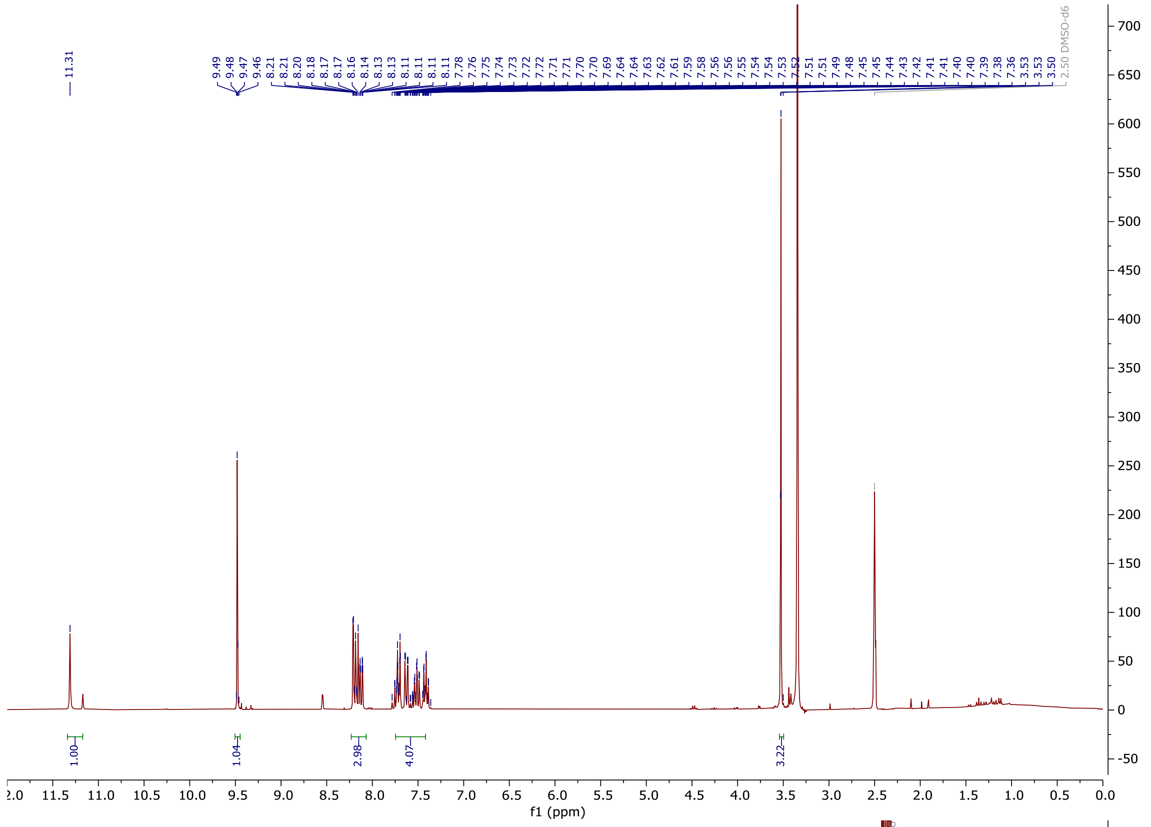

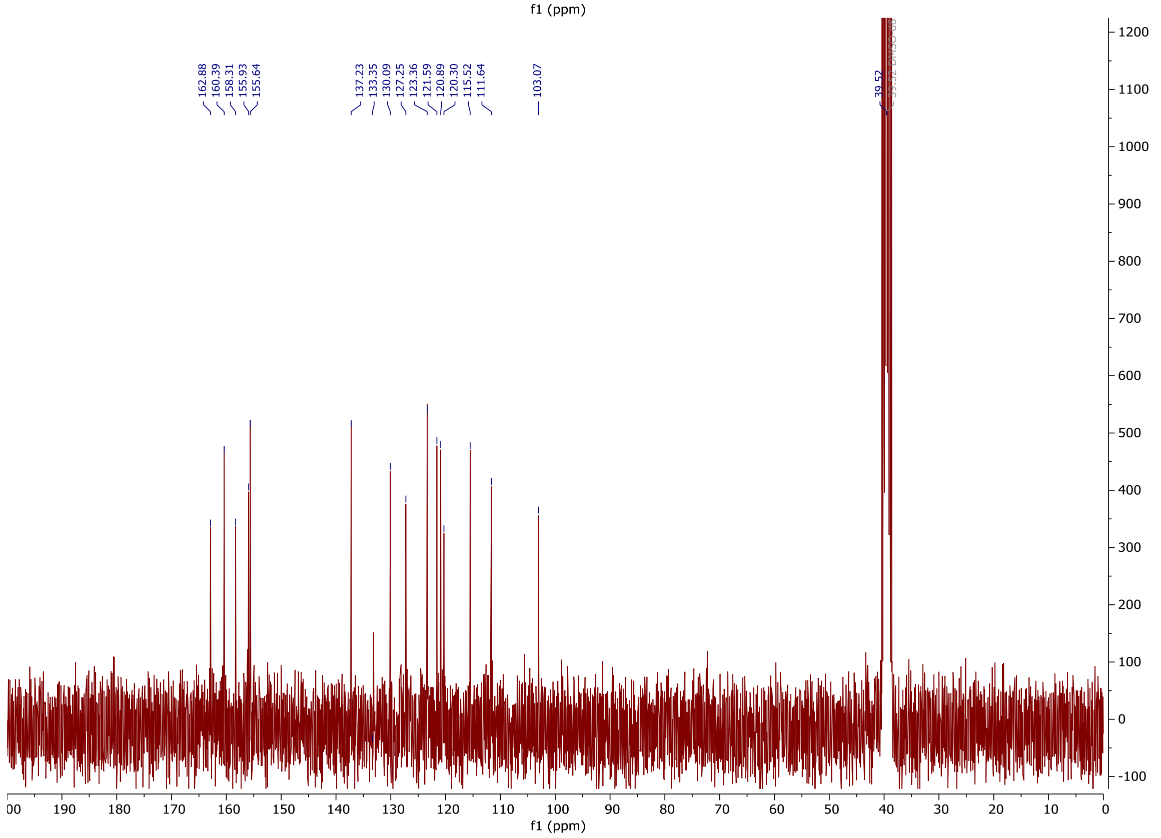

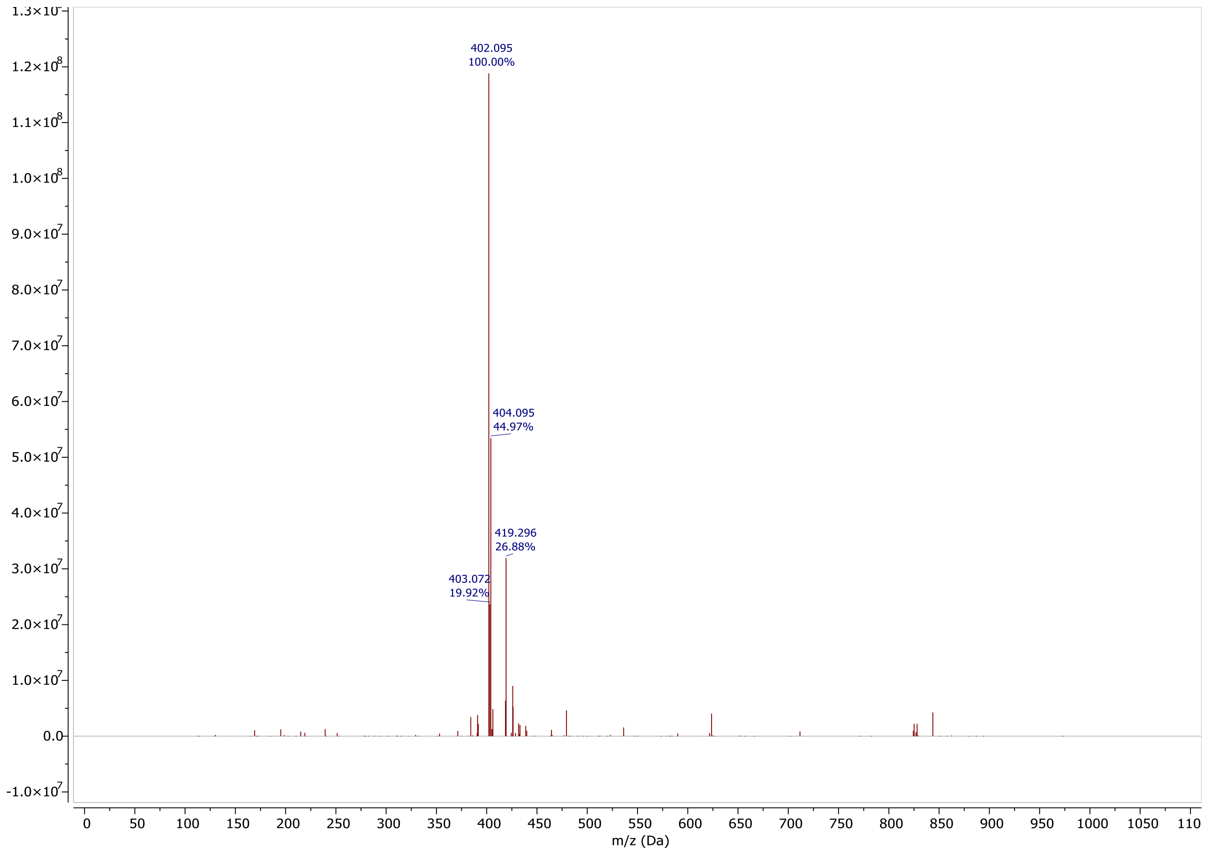
402.095
100.00%
404.095
44.97%
419.296
26.88%
403.072
19.92%
m/z (Da)

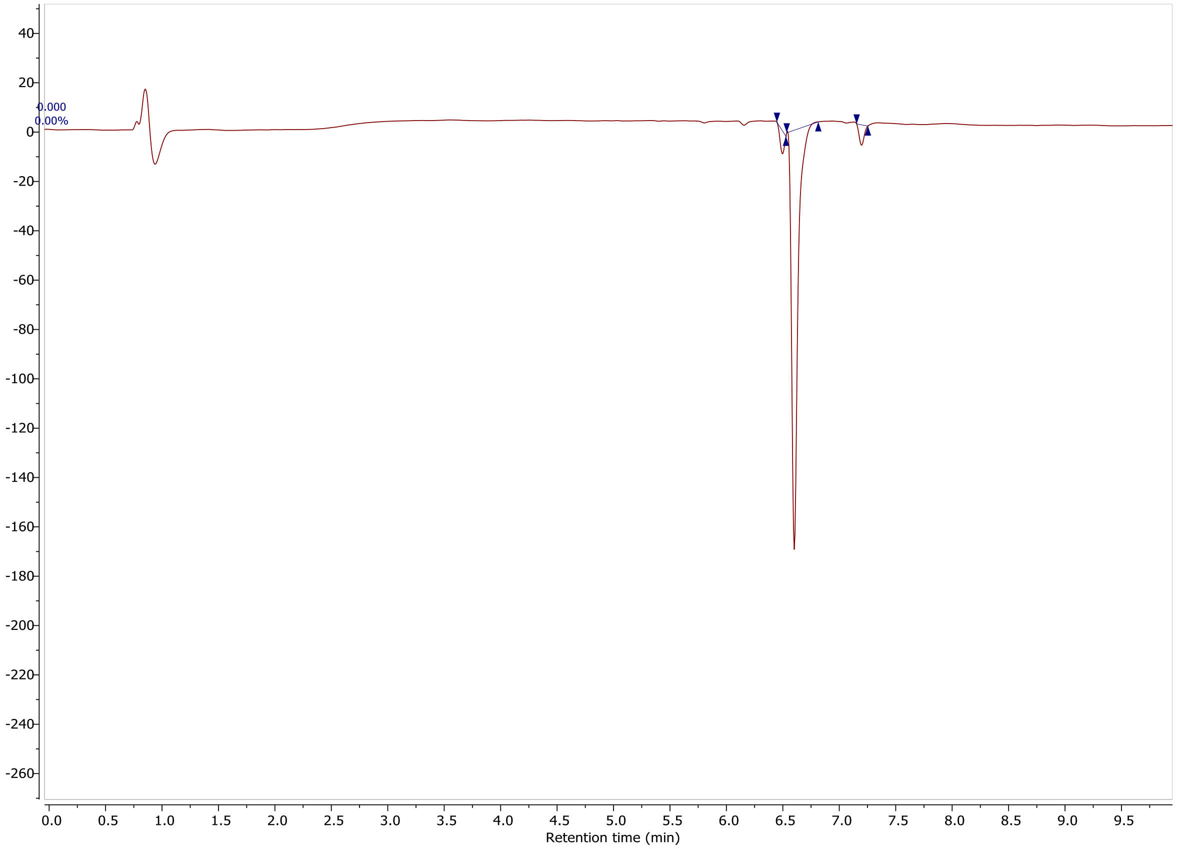
0.000
0.00%
Retention time (min)

6m

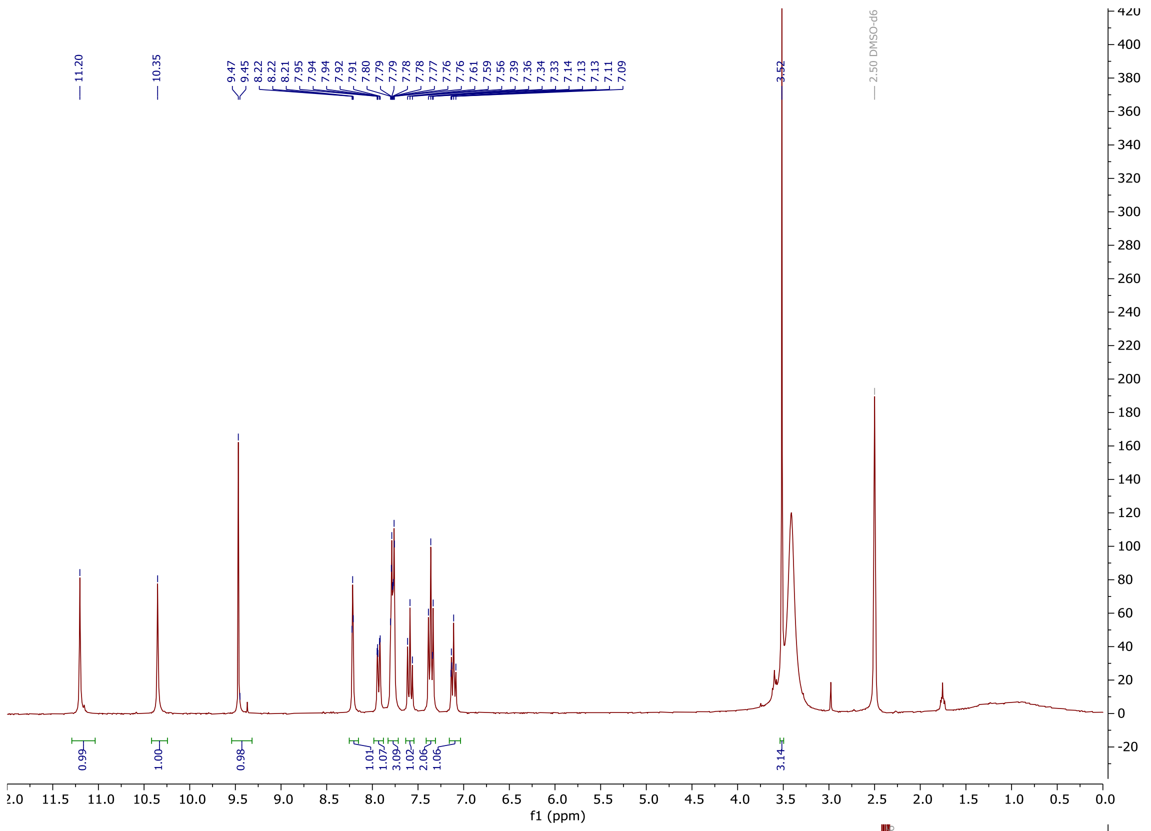

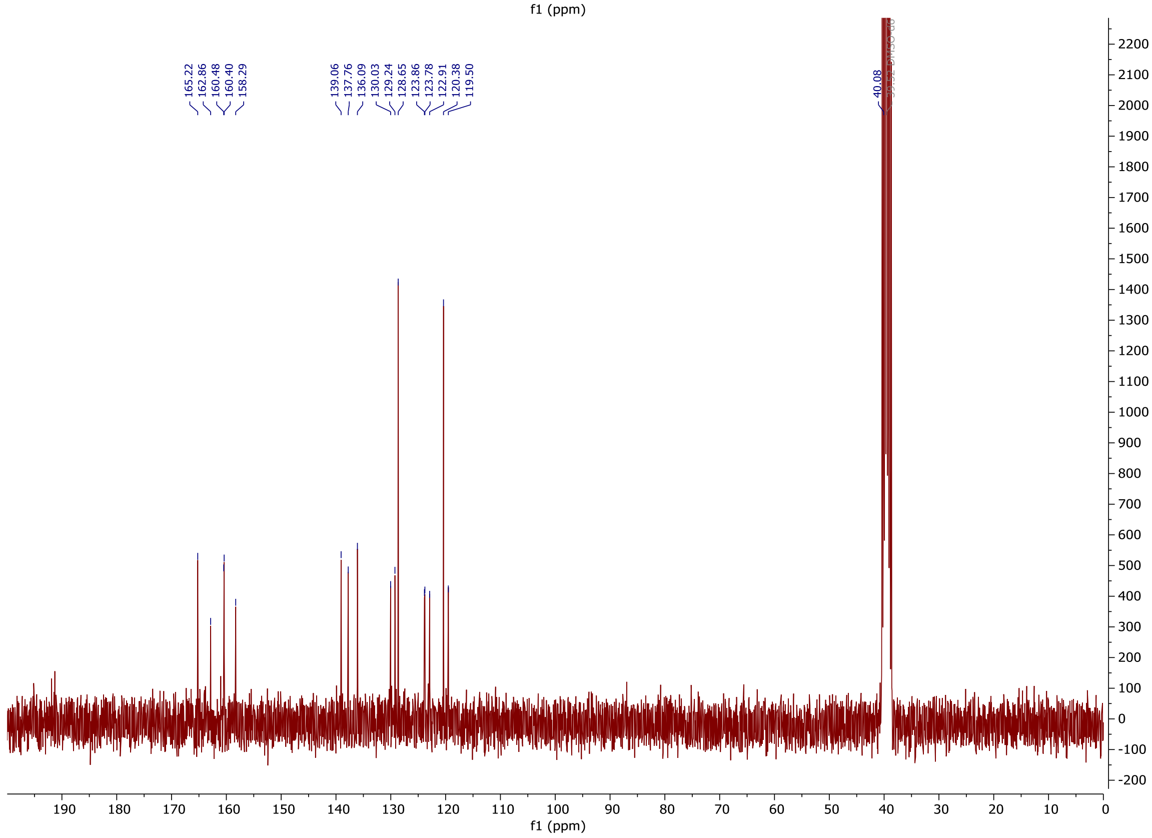

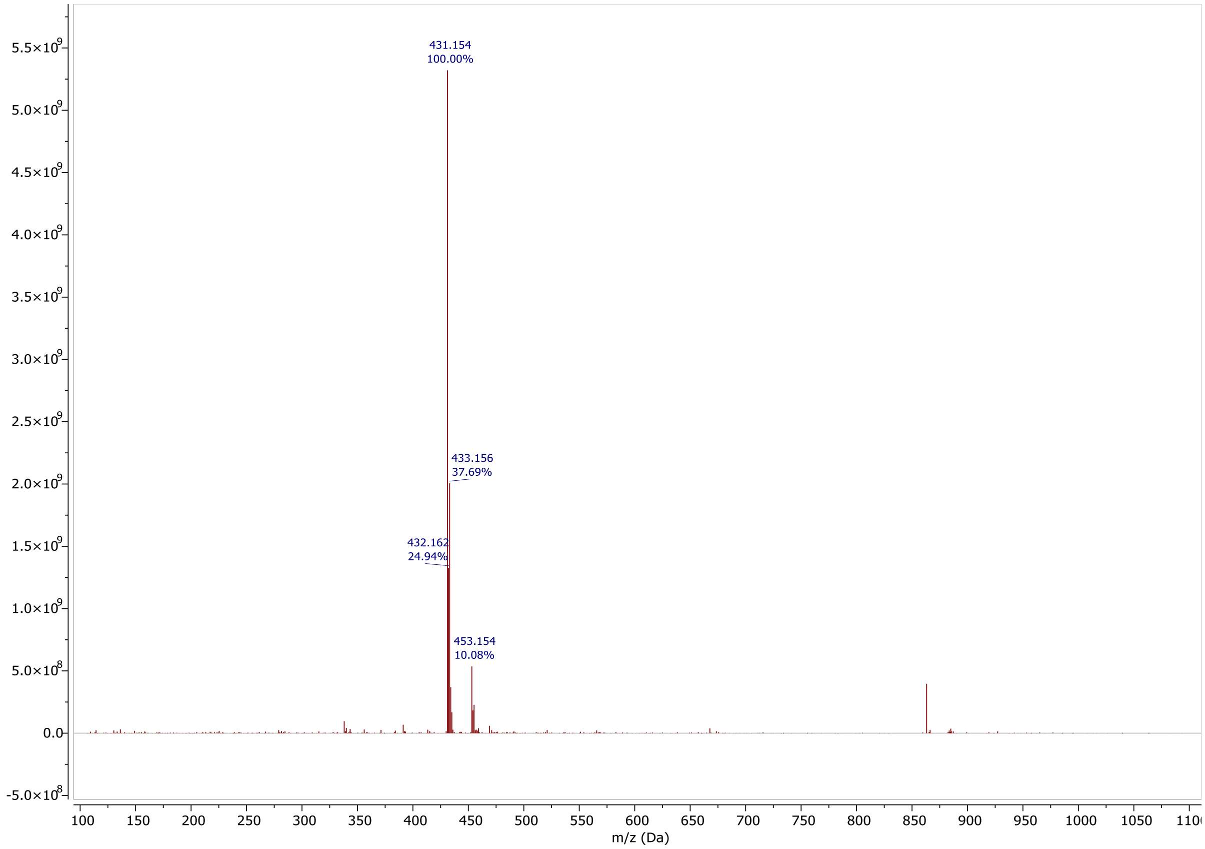
431.154
100.00%
433.156
37.69%
432.162
24.94%
453.154
10.08%
m/z (Da)

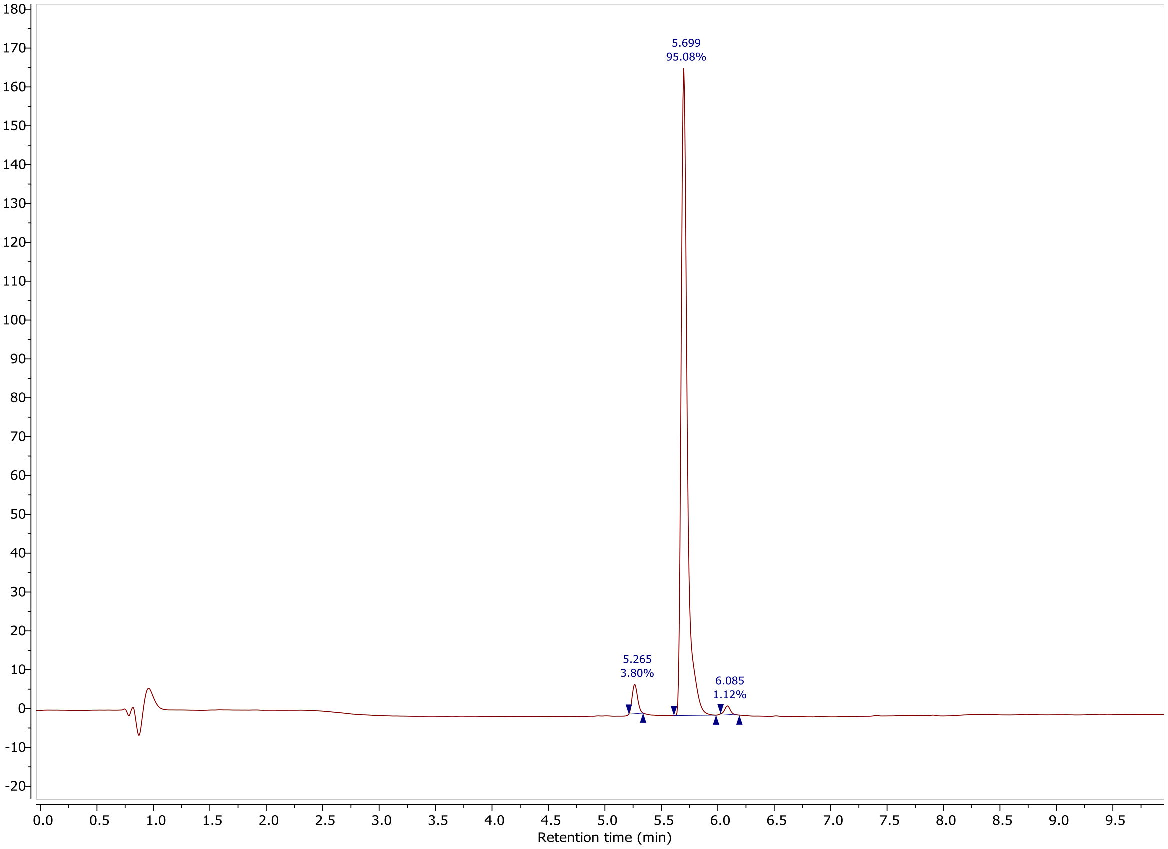
5.699
95.08%
5.265
3.80%
6.085
1.12%
Retention time (min)

6n

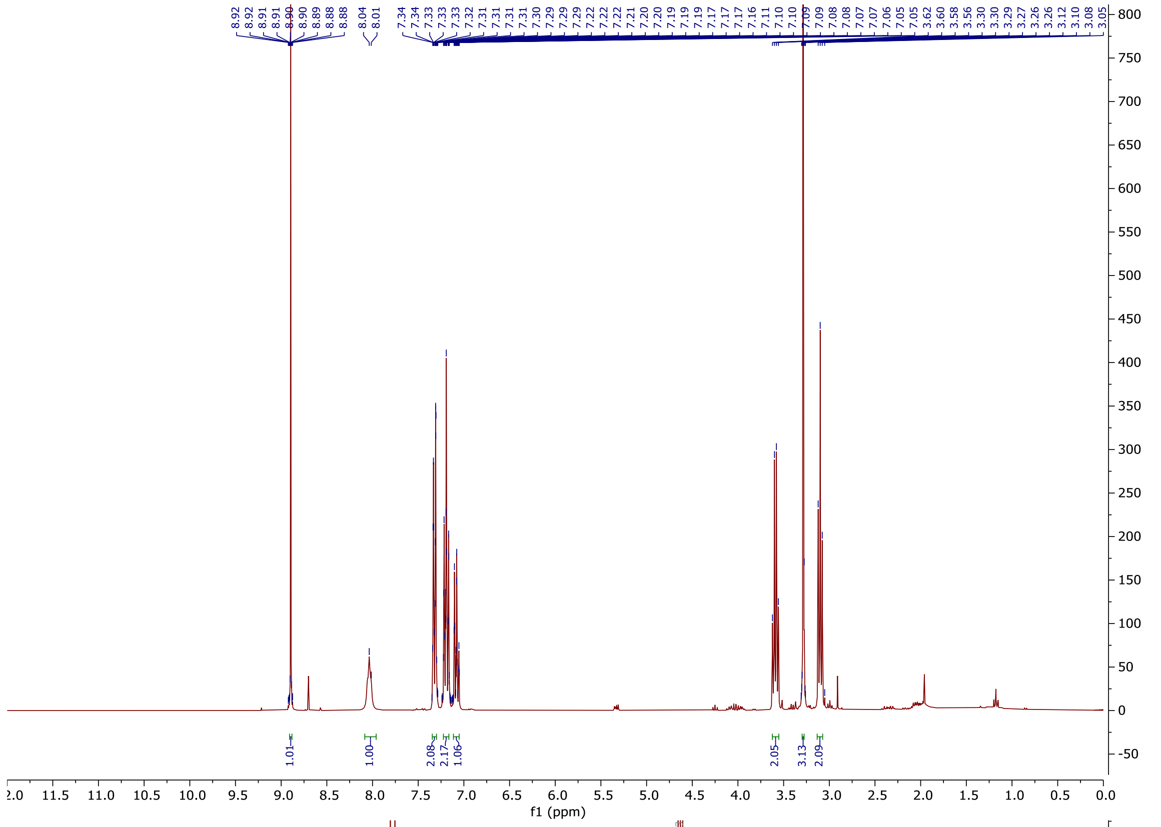

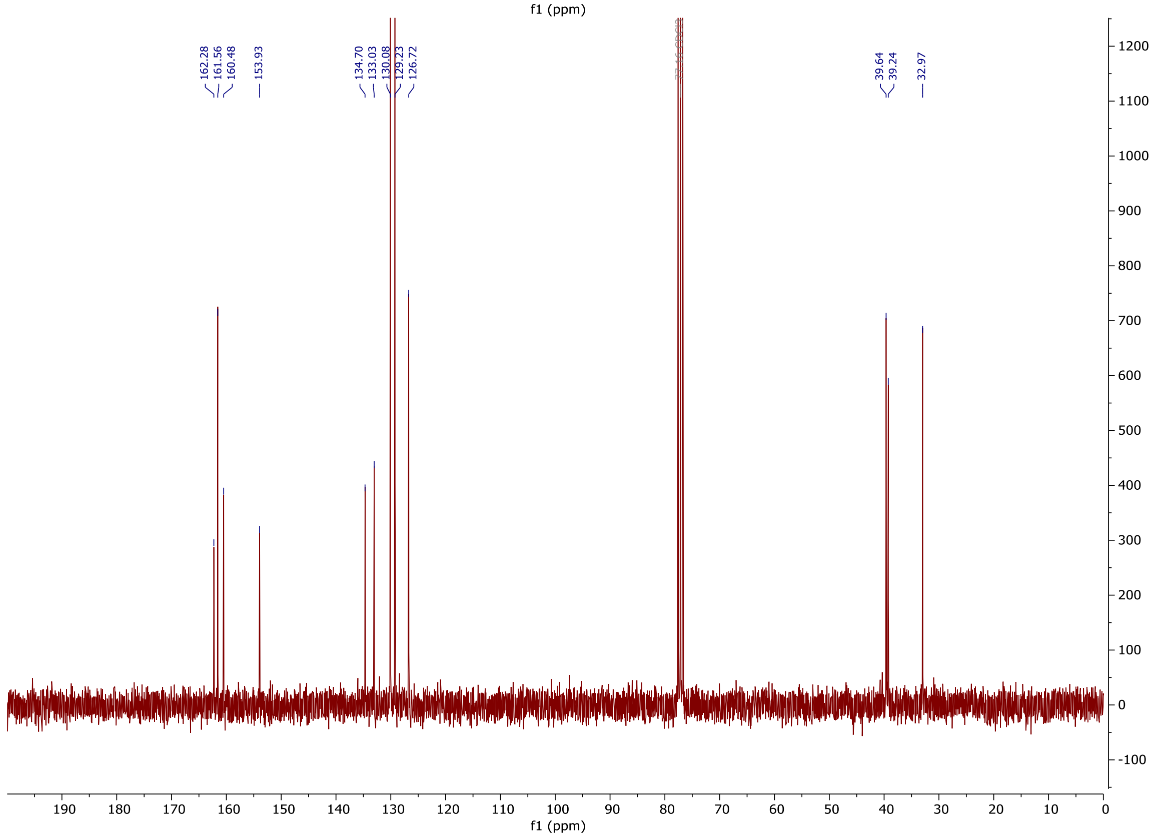

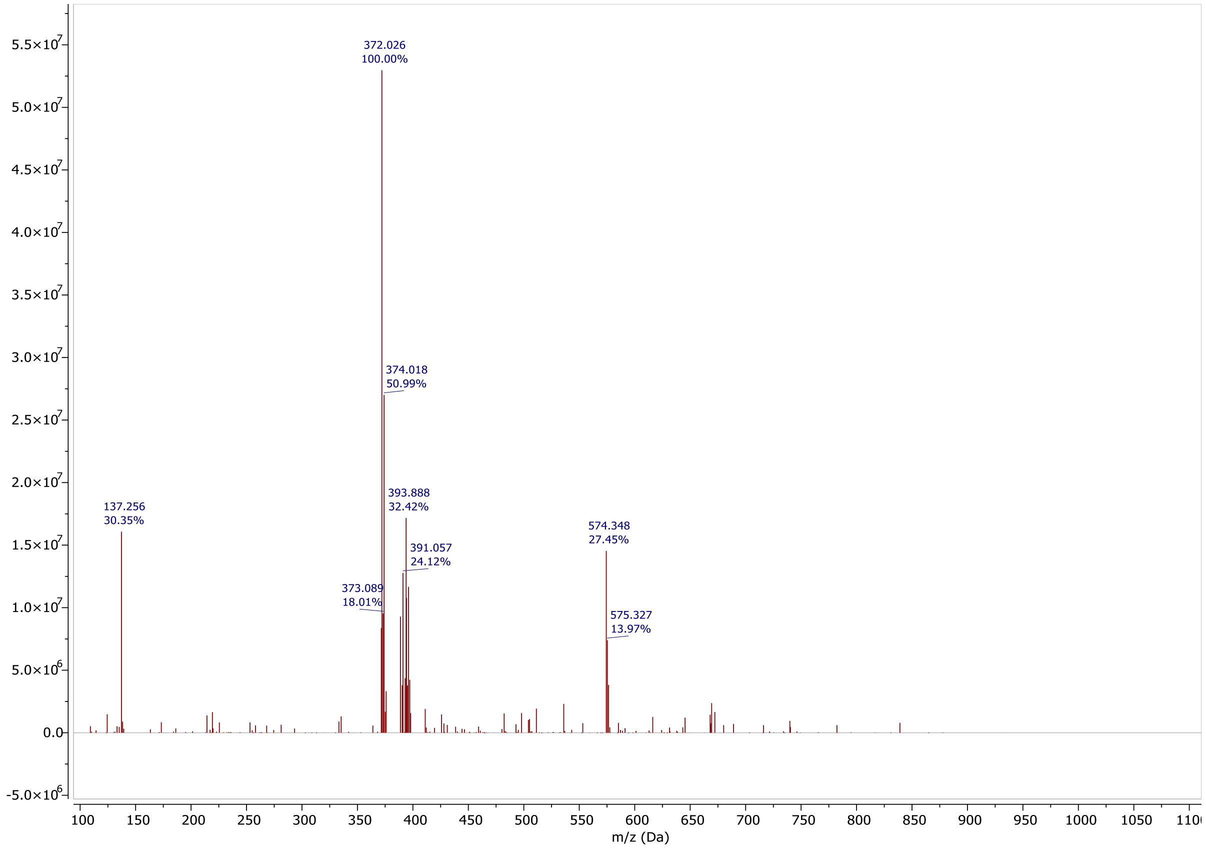
372.026
100.00%
374.018
50.99%
393.888
32.42%
391.057
24.12%
373.089
18.01%
137.256
30.35%
574.348
27.45%
575.327
13.97%
m/z (Da)

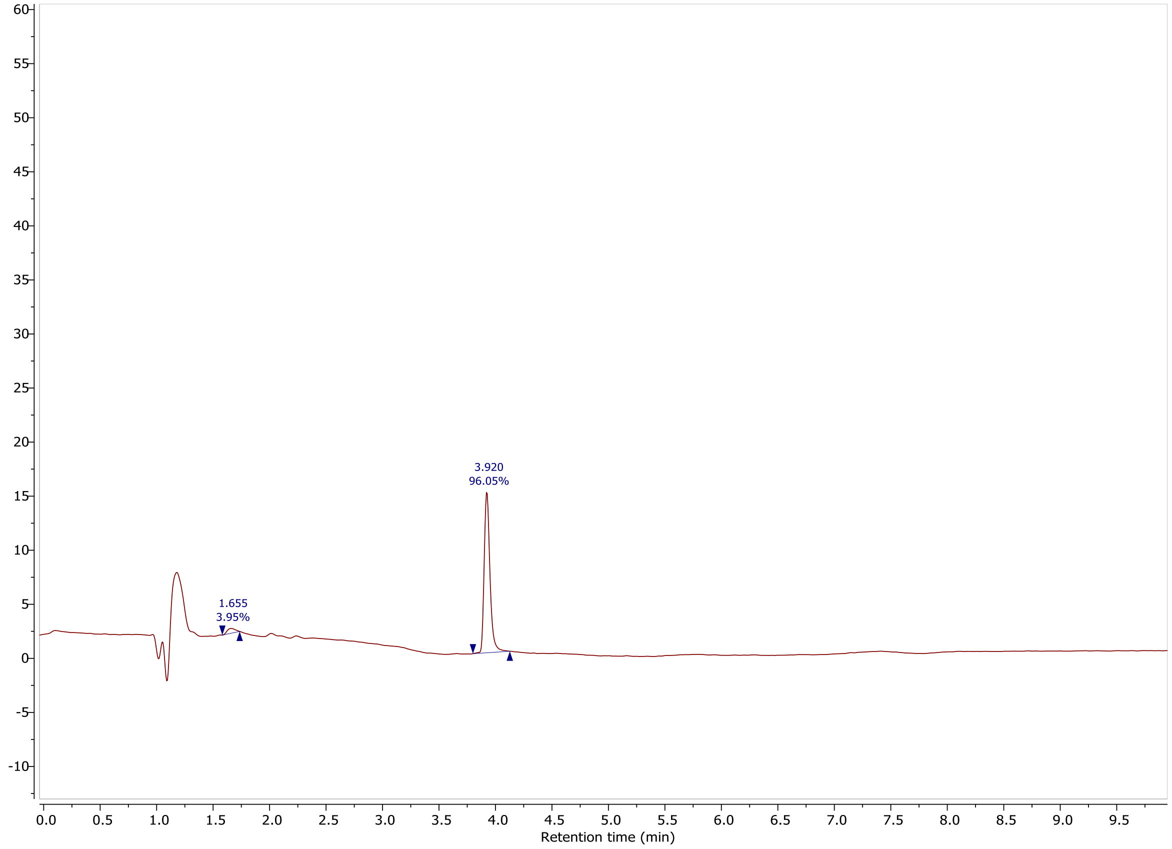
3.920
96.05%
1.655
3.95%
Retention time (min)

7a

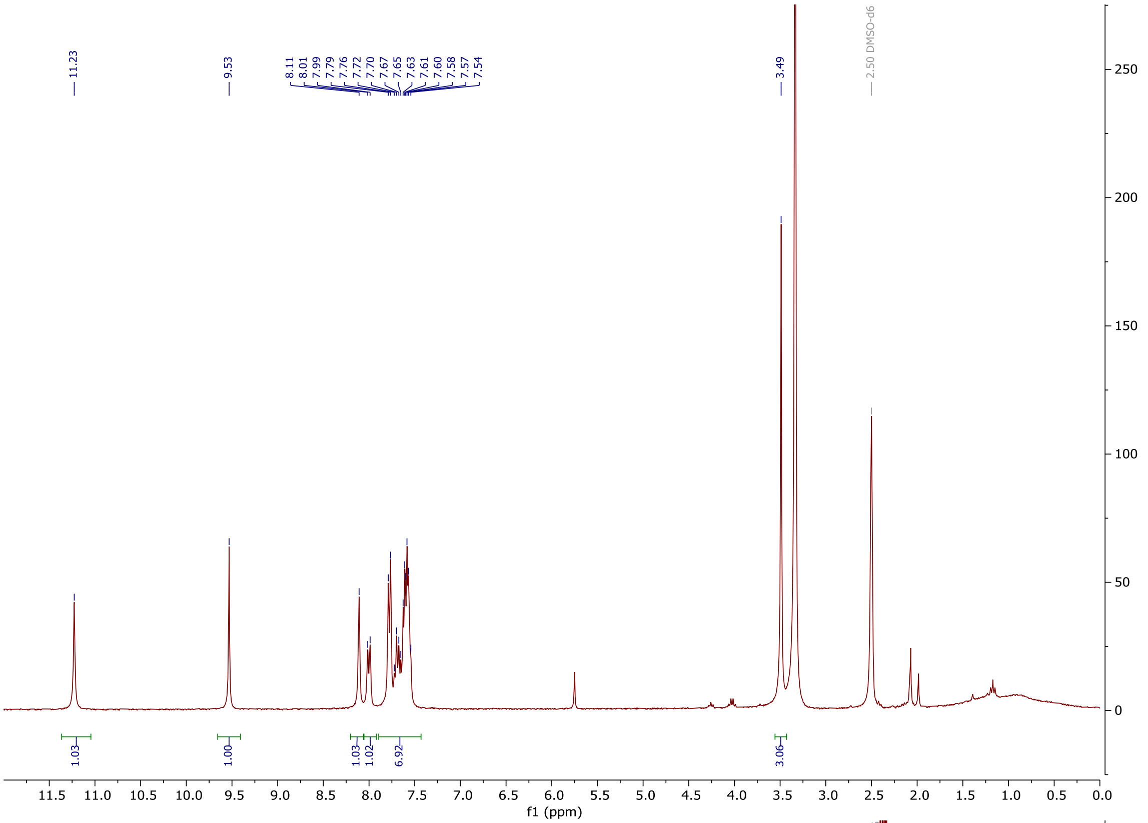
11.23
9.53
8.11 8.01 7.99 7.79 7.76 7.72 7.70 7.67 7.65 7.63 7.61 7.60 7.58 7.57 7.54
3.49
2.50 DMSO-d6
1.03
1.00
1.03
1.02
6.92
3.06
f1 (ppm)

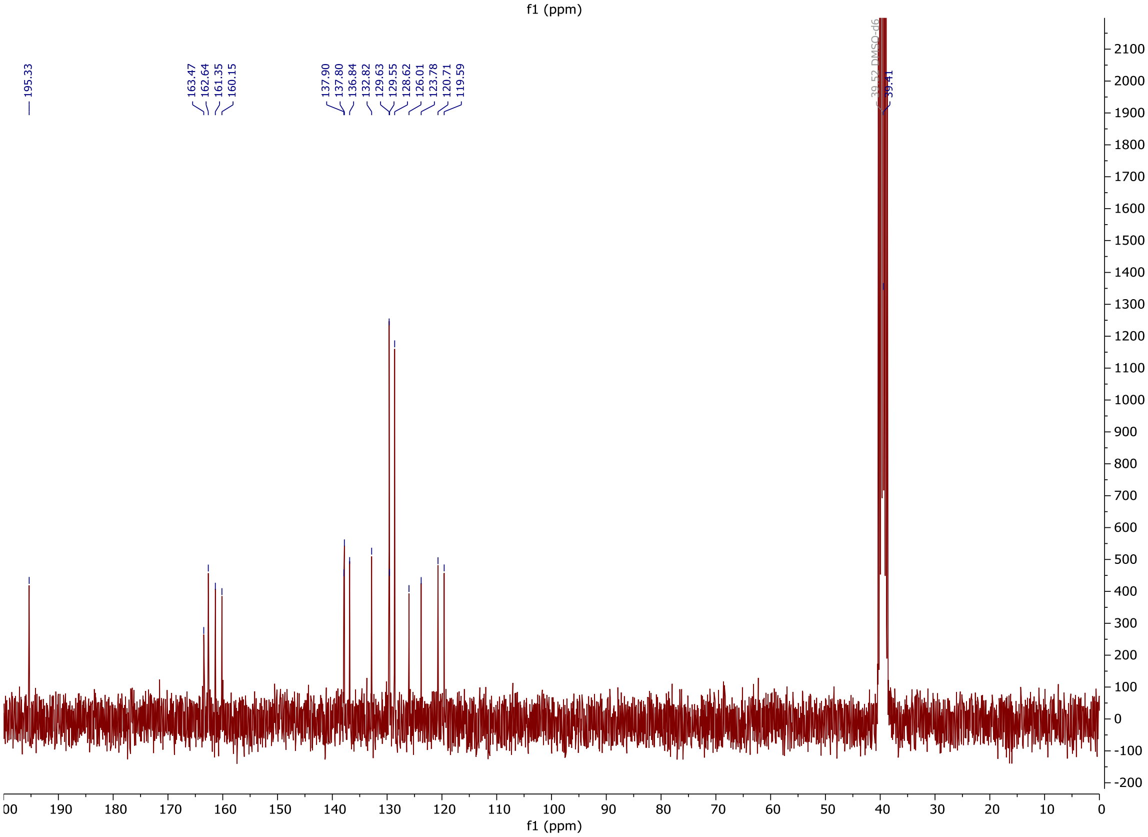
195.33
163.47 162.64 161.35 160.15
137.90 137.80 136.84 132.82 129.63 129.55 128.62 126.01 123.78 120.71 119.59
39.52 DMSO-d6
39.41
f1 (ppm)

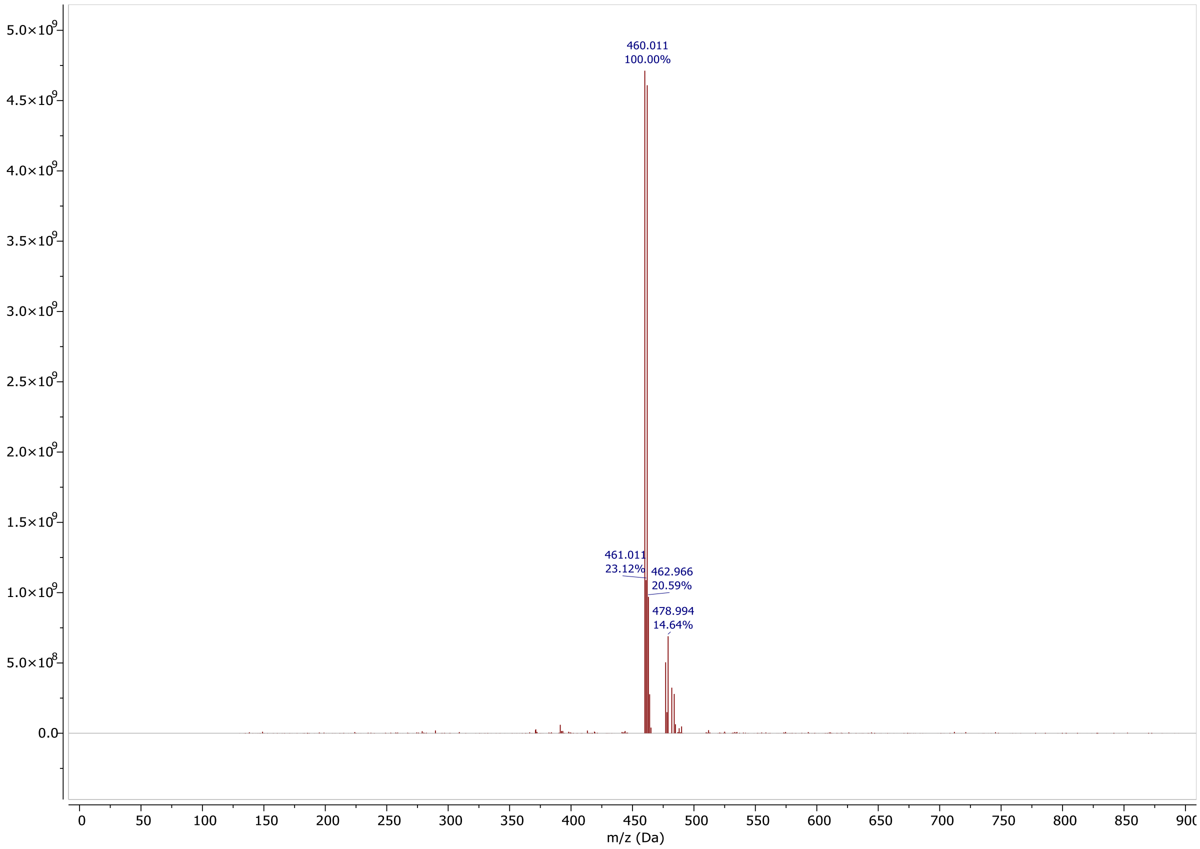
460.011
100.00%
461.011
23.12%
462.966
20.59%
478.994
14.64%
m/z (Da)

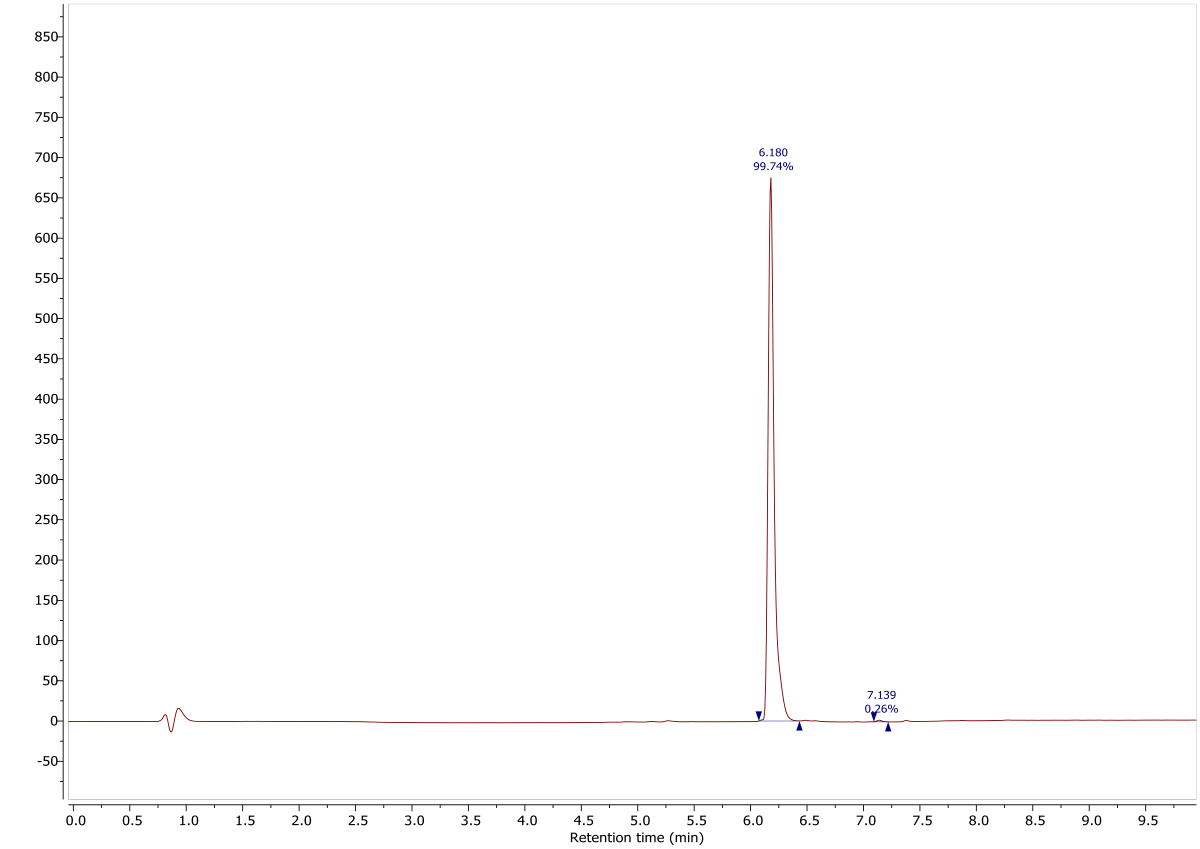
6.180
99.74%
7.139
0.26%
Retention time (min)

7b

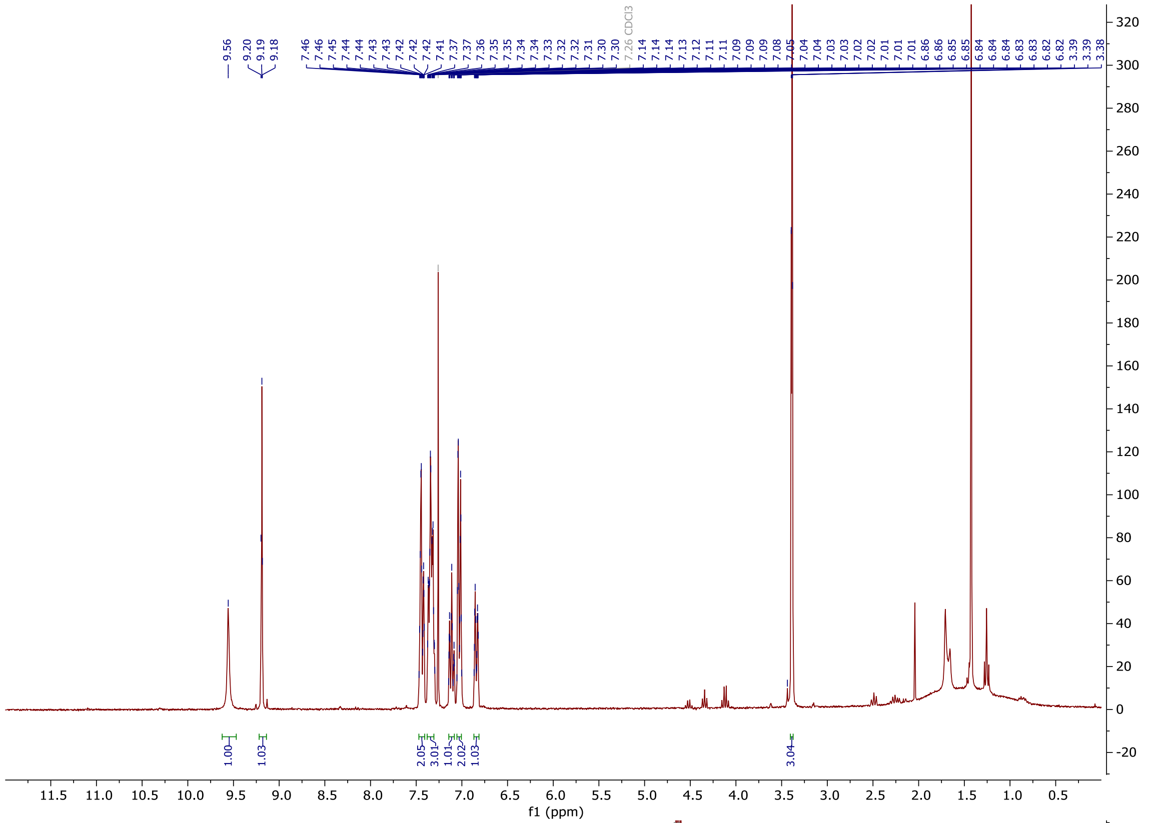
9.56
9.20
9.19
9.18
7.46
7.46
7.45
7.44
7.44
7.43
7.43
7.42
7.42
7.41
7.37
7.37
7.36
7.35
7.35
7.34
7.34
7.33
7.32
7.32
7.31
7.30
7.30
7.26 CDCl3
7.14
7.14
7.14
7.13
7.12
7.11
7.11
7.09
7.09
7.09
7.08
7.05
7.04
7.04
7.03
7.03
7.02
7.02
7.01
7.01
7.01
6.86
6.86
6.85
6.85
6.84
6.84
6.84
6.83
6.83
6.82
6.82
3.39
3.39
3.38
1.00
1.03
2.05
3.01
1.01
2.02
1.03
3.04
f1 (ppm)

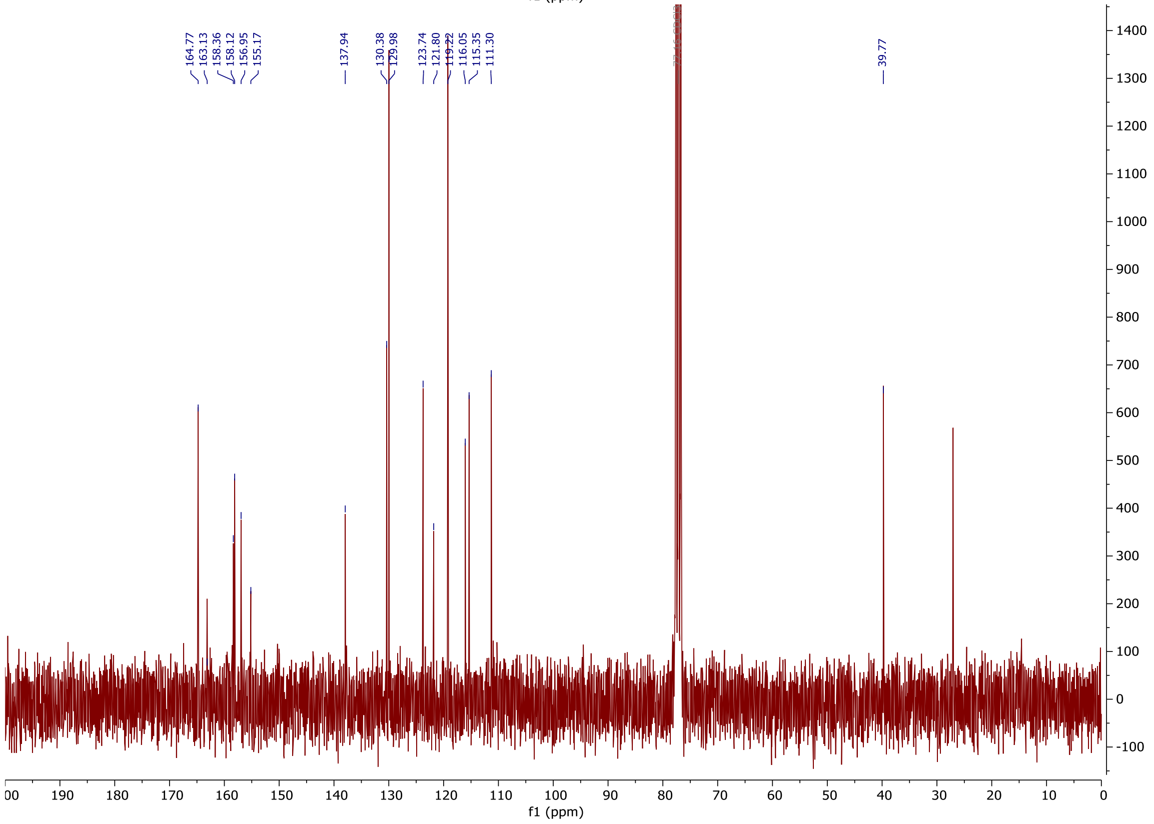
164.77
163.13
158.36
158.12
156.95
155.17
137.94
130.38
129.98
123.74
121.80
119.22
116.05
115.35
111.30
39.77
f1 (ppm)

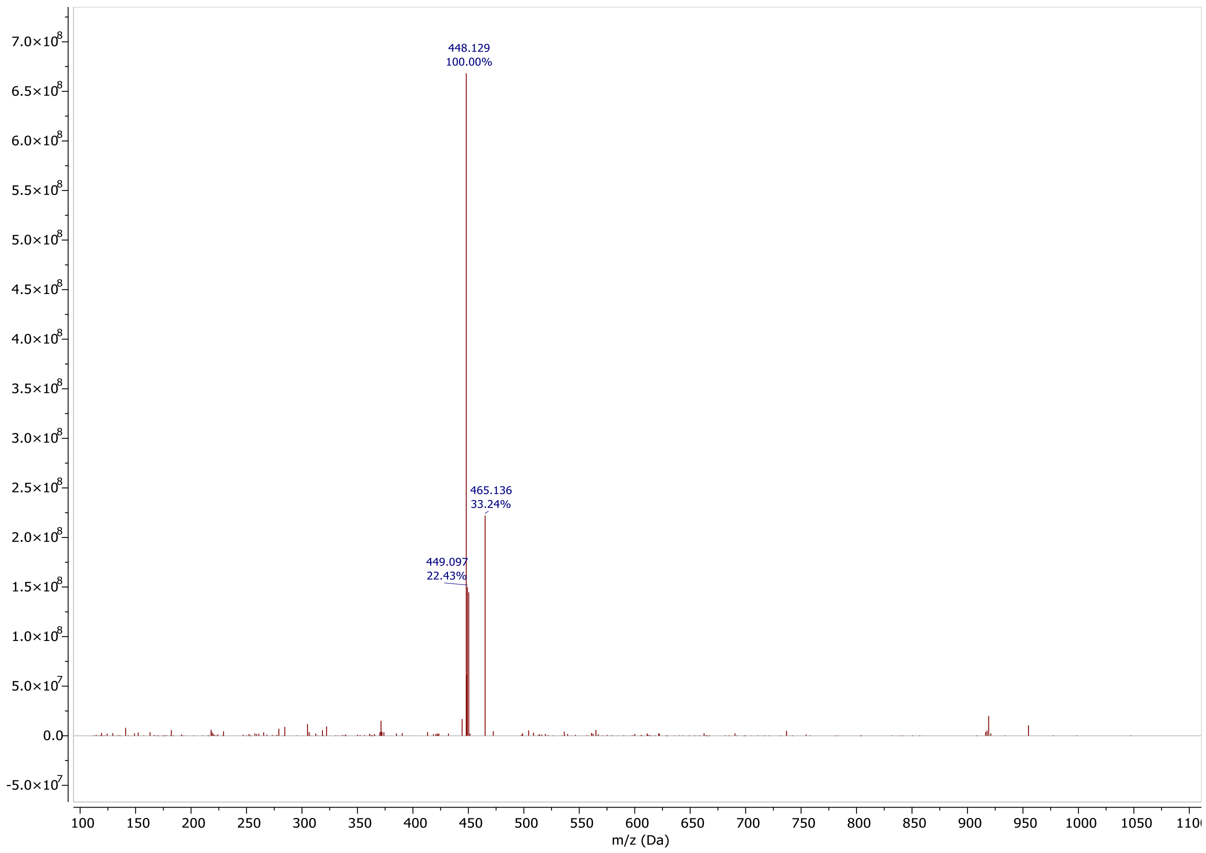
448.129
100.00%
465.136
33.24%
449.097
22.43%
m/z (Da)

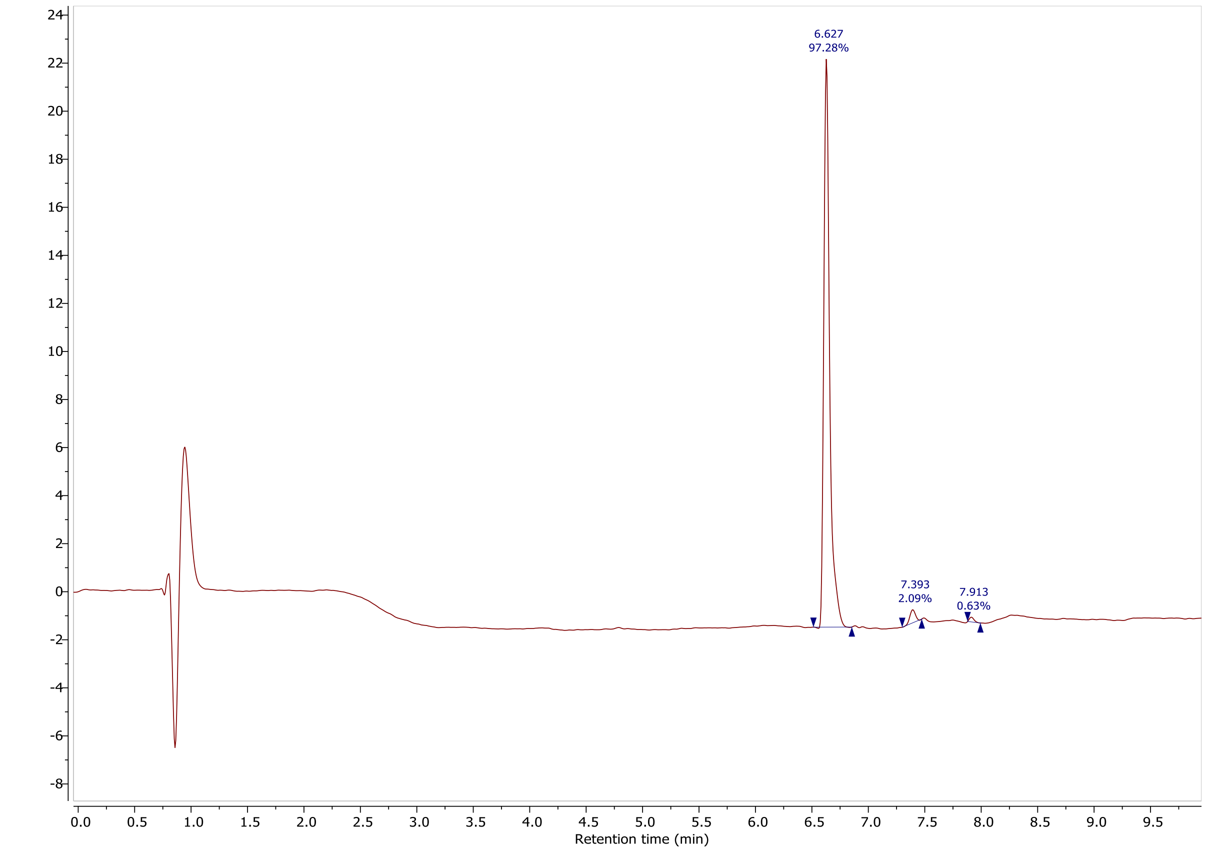
6.627
97.28%
7.393
2.09%
7.913
0.63%
Retention time (min)

7c

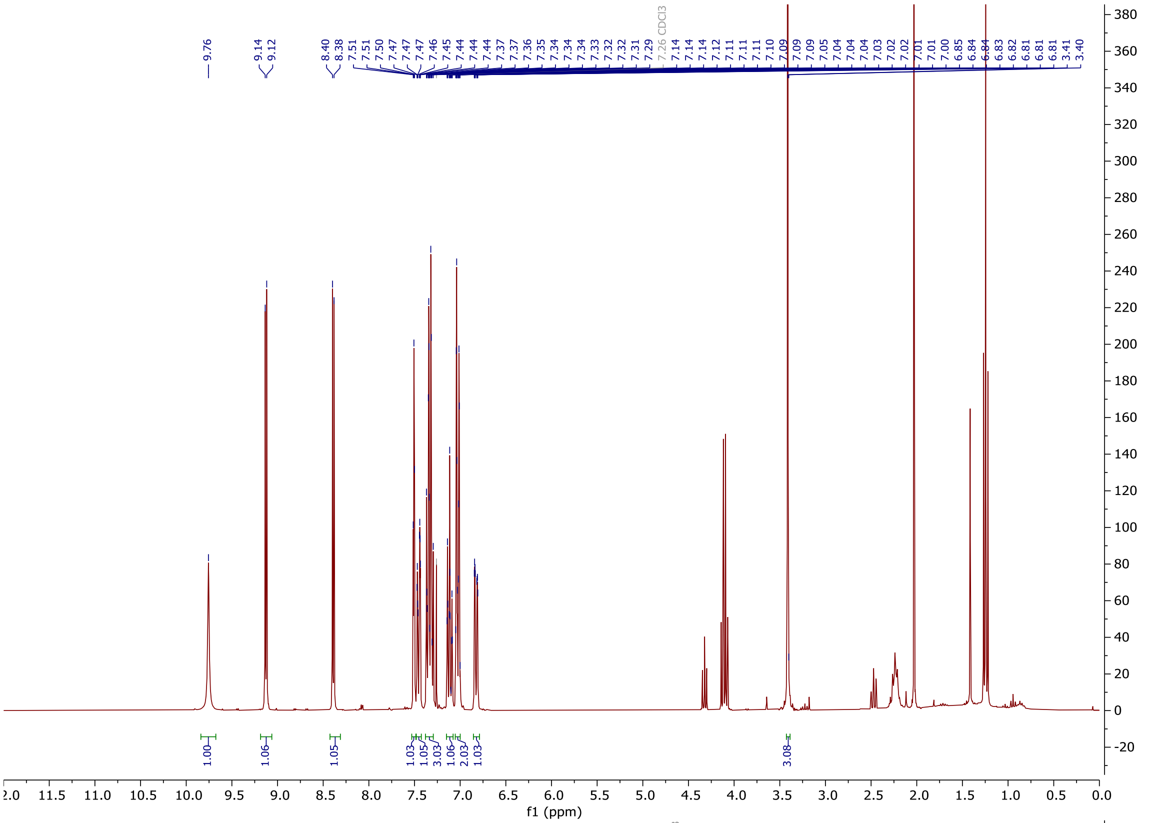
9.76
9.14
9.12
8.40
8.38
7.51
7.51
7.50
7.47
7.47
7.46
7.45
7.44
7.44
7.44
7.37
7.37
7.36
7.35
7.34
7.34
7.34
7.33
7.32
7.32
7.31
7.29
7.26 CDCl3
7.14
7.14
7.14
7.14
7.12
7.11
7.11
7.11
7.10
7.09
7.09
7.09
7.05
7.04
7.04
7.04
7.03
7.02
7.02
7.01
7.01
7.00
6.85
6.84
6.84
6.83
6.82
6.81
6.81
6.81
3.41
3.40
1.00
1.06
1.05
1.03
1.05
3.03
1.06
2.03
1.03
3.08
f1 (ppm)

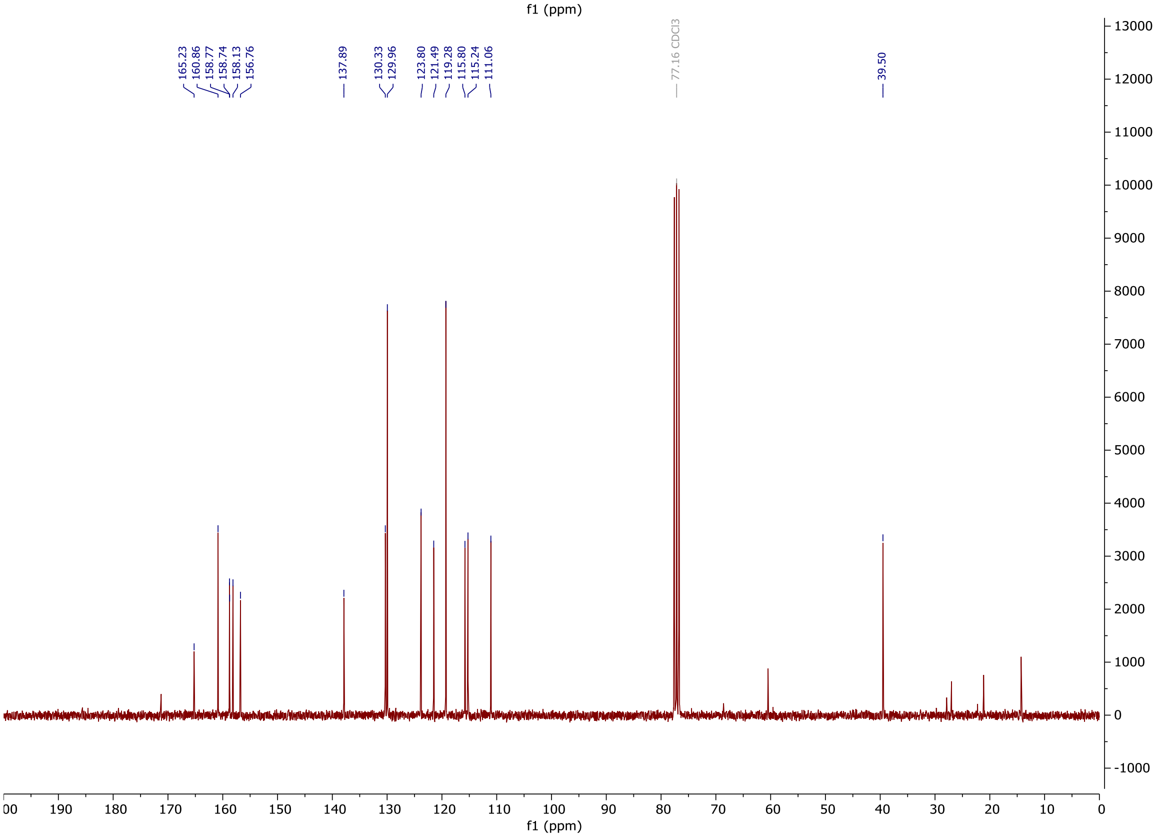
165.23
160.86
158.77
158.74
158.13
156.76
137.89
130.33
129.96
123.80
121.49
119.28
115.80
115.24
111.06
77.16 CDCl3
39.50
f1 (ppm)

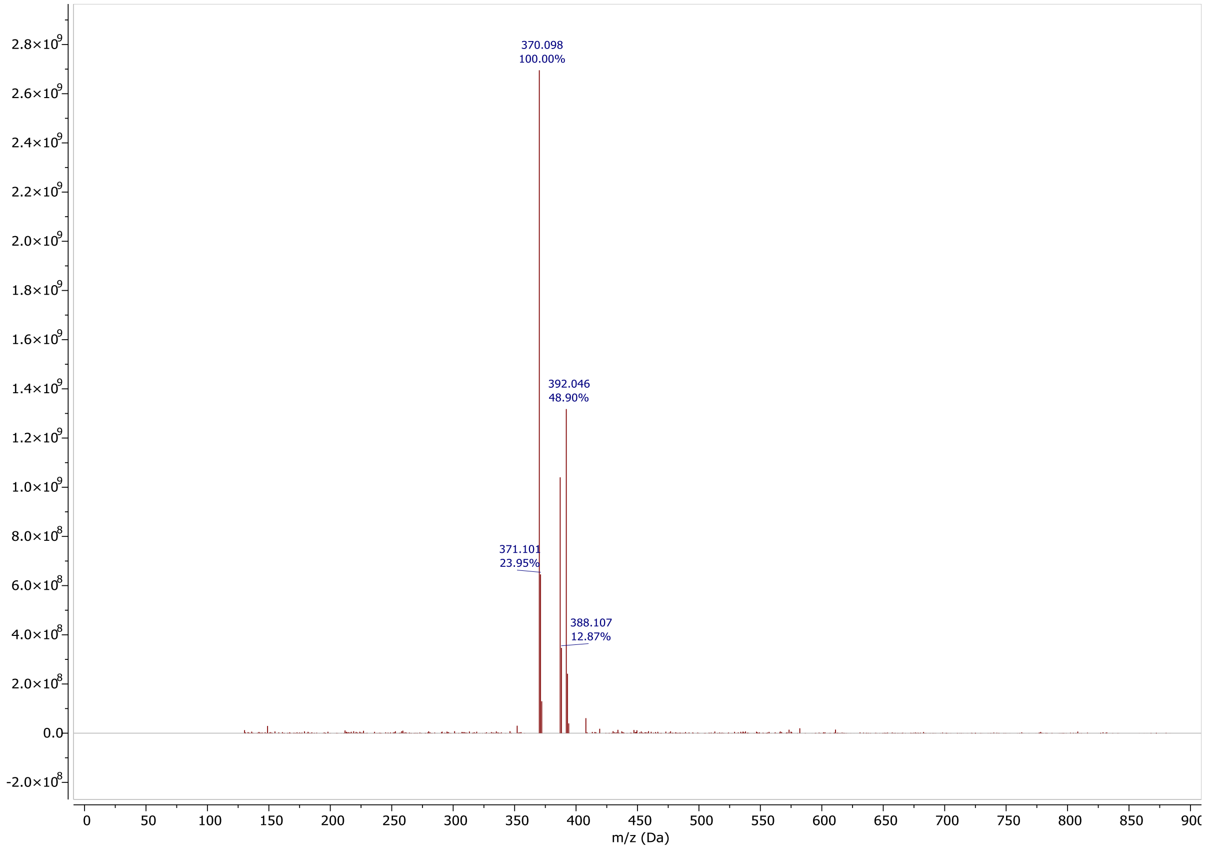
370.098
100.00%
392.046
48.90%
371.101
23.95%
388.107
12.87%
m/z (Da)

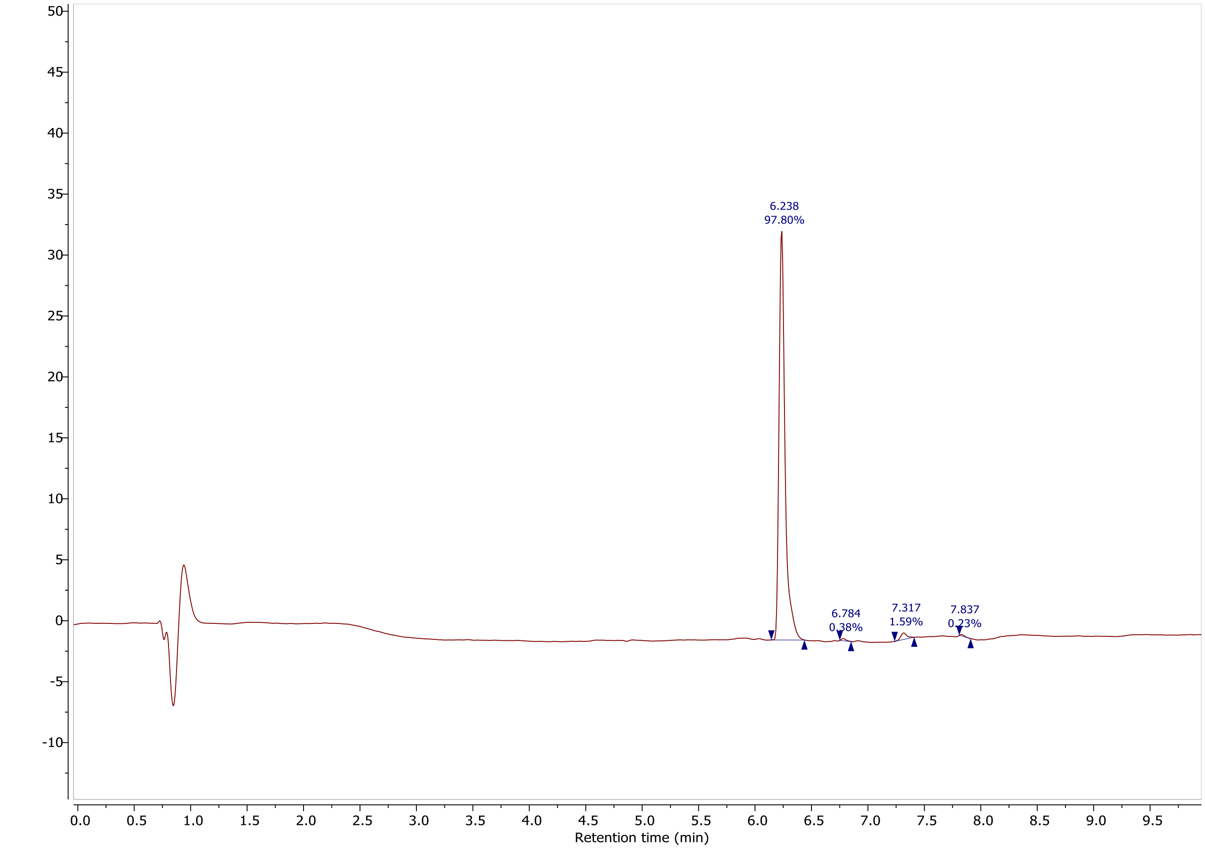
6.238
97.80%
6.784
0.38%
7.317
1.59%
7.837
0.23%
Retention time (min)

7d

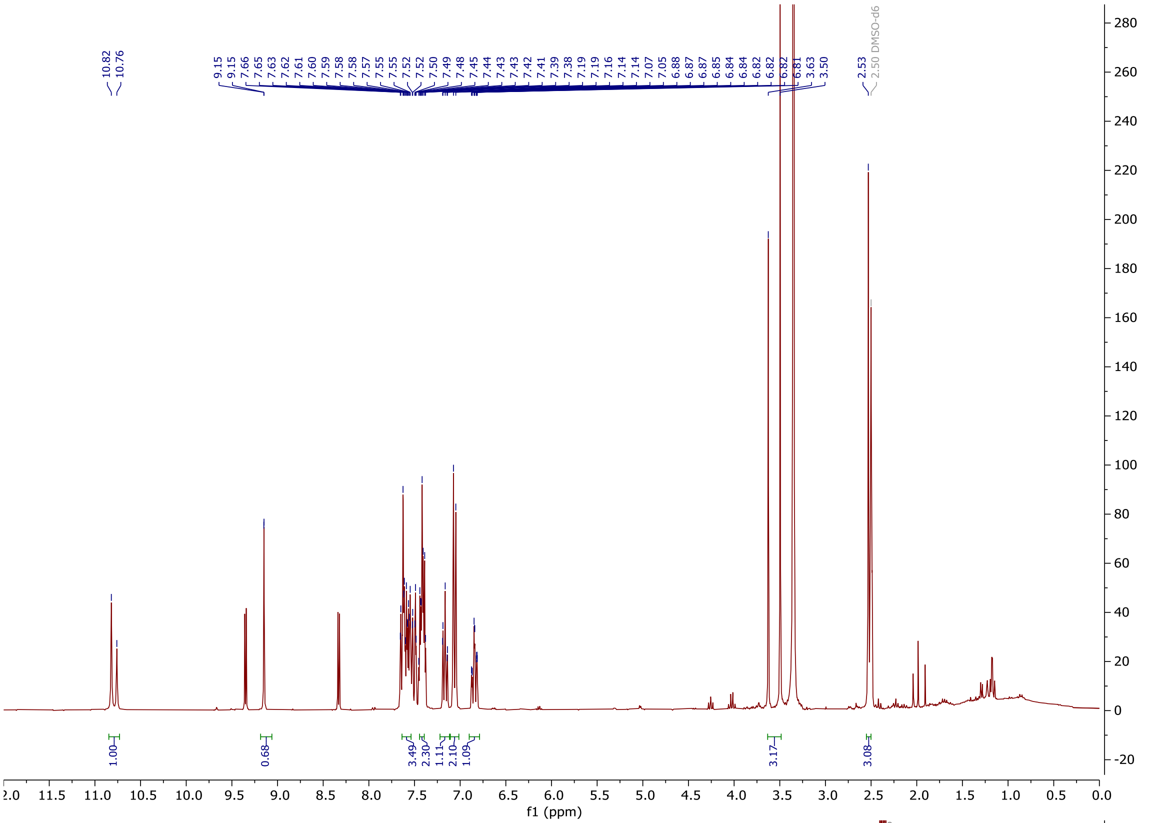
10.82
10.76
9.15
9.15
7.66
7.65
7.63
7.62
7.61
7.60
7.59
7.58
7.58
7.57
7.55
7.55
7.52
7.52
7.50
7.49
7.48
7.45
7.44
7.43
7.42
7.41
7.39
7.38
7.19
7.19
7.16
7.14
7.14
7.07
7.05
6.88
6.87
6.87
6.85
6.84
6.84
6.82
6.82
6.82
6.81
3.63
3.50
2.53
2.50 DMSO-d6
1.00
0.68
3.49
2.30
1.11
2.10
1.09
3.17
3.08
f1 (ppm)

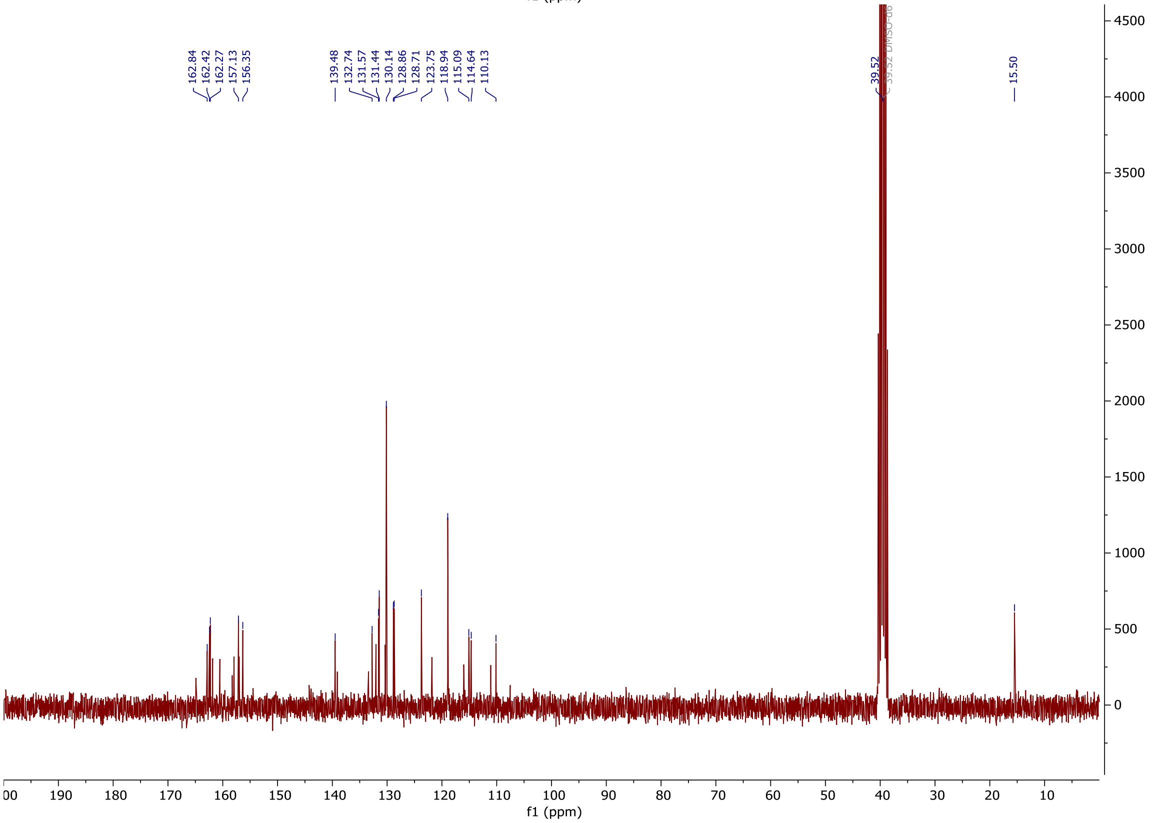
162.84
162.42
162.27
157.13
156.35
139.48
132.74
131.57
131.44
130.14
128.86
128.71
123.75
118.94
115.09
114.64
110.13
39.52
39.52 DMSO-d6
15.50
f1 (ppm)

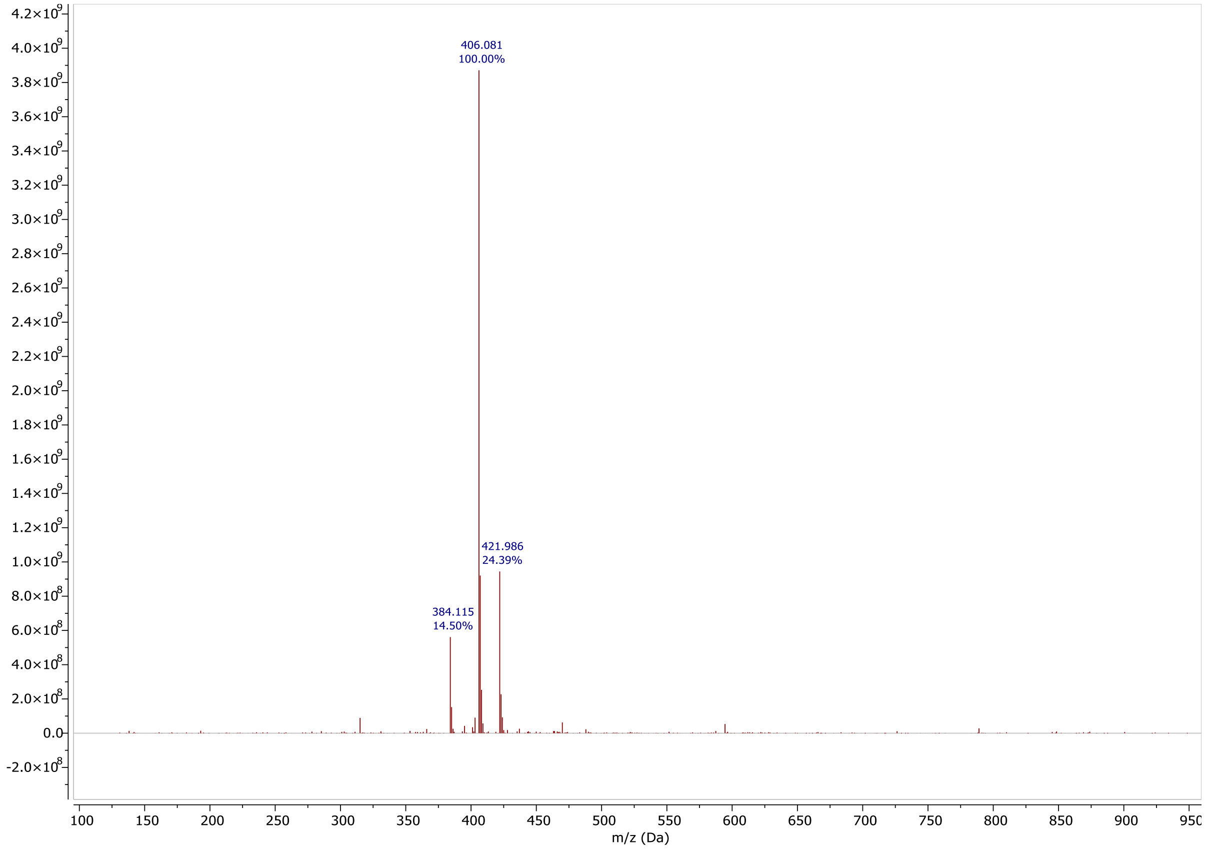
406.081
100.00%
421.986
24.39%
384.115
14.50%
m/z (Da)

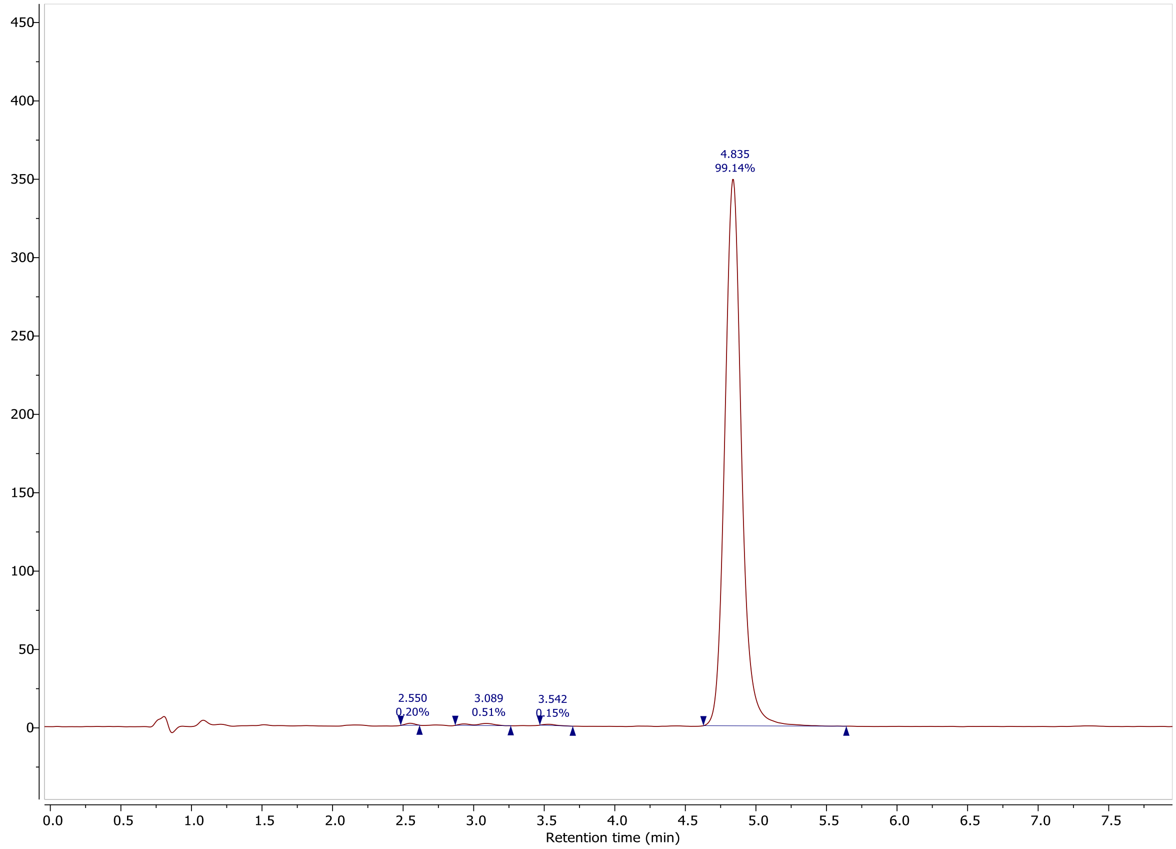
4.835
99.14%
2.550
0.20%
3.089
0.51%
3.542
0.15%
Retention time (min)

7e

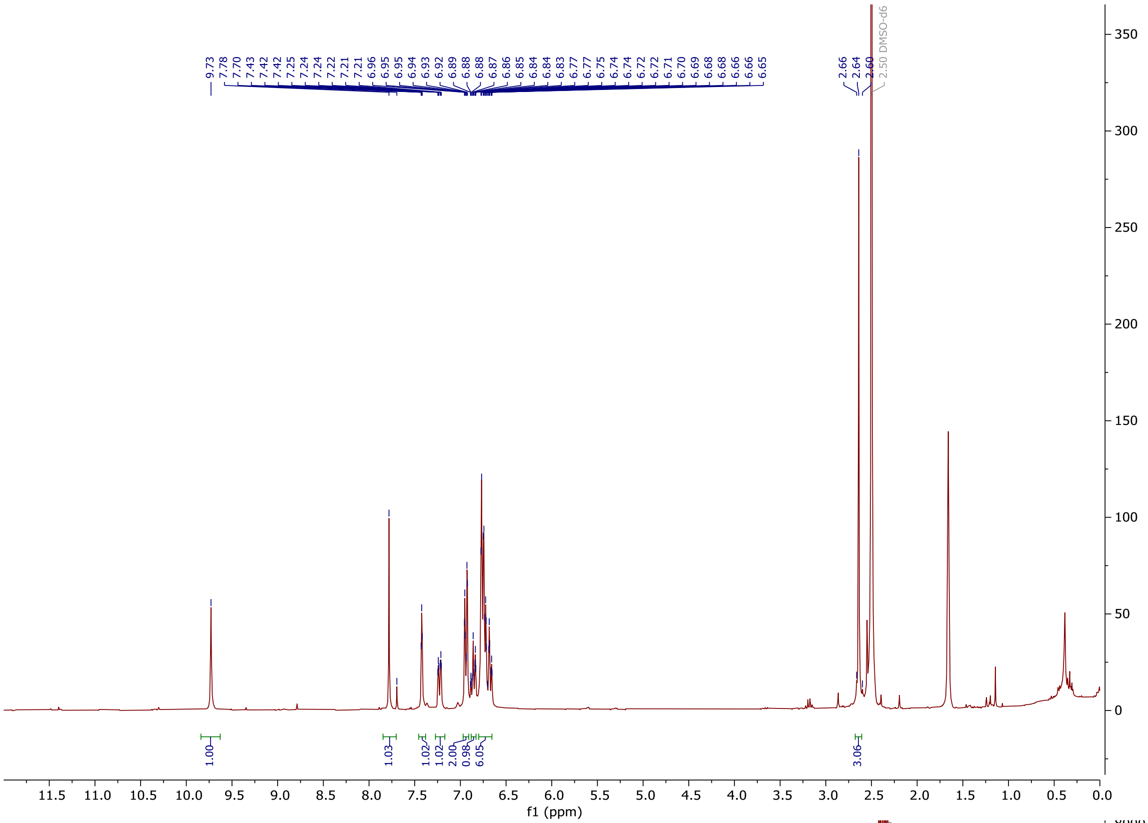
9.73
7.78
7.70
7.43
7.42
7.25
7.24
7.24
7.22
7.21
7.21
6.96
6.95
6.95
6.94
6.93
6.92
6.89
6.88
6.88
6.87
6.86
6.85
6.84
6.84
6.83
6.77
6.77
6.75
6.74
6.74
6.72
6.72
6.71
6.70
6.69
6.68
6.68
6.66
6.66
6.65
2.66
2.64
2.60
2.50 DMSO-d6
1.00
1.03
1.02
1.02
2.00
0.98
6.05
3.06
f1 (ppm)

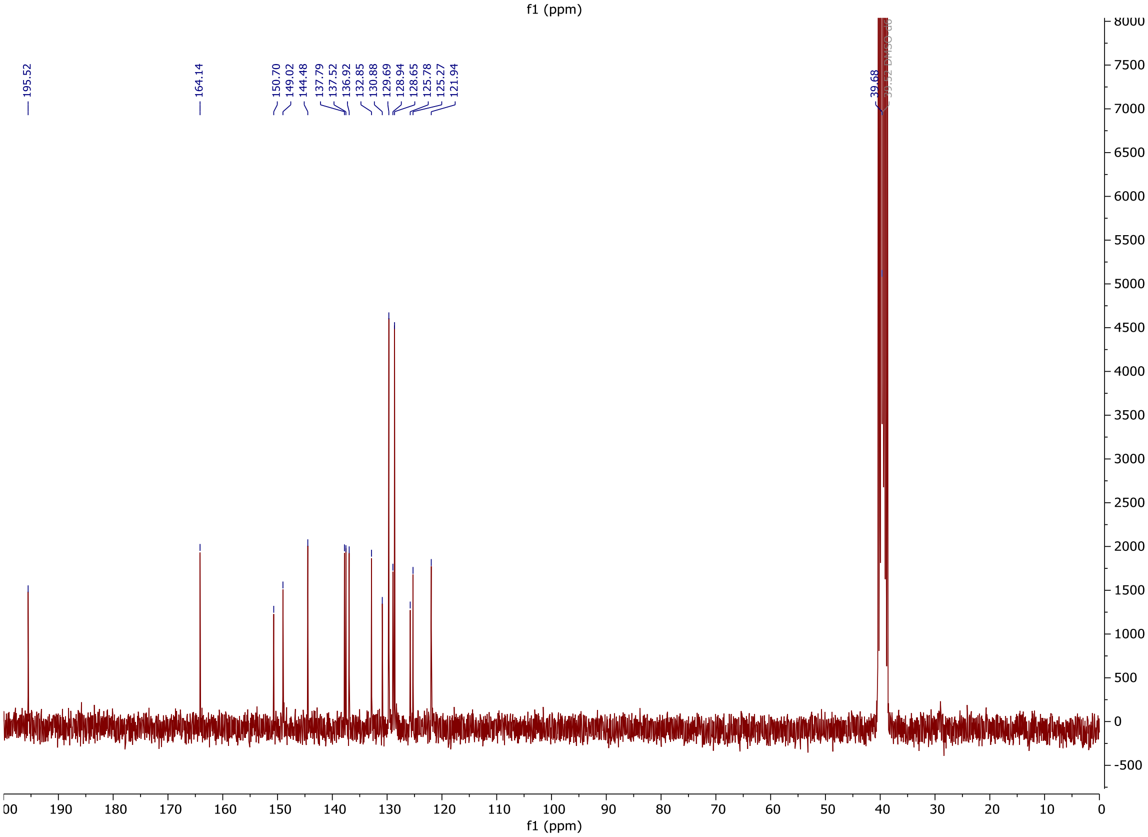
195.52
164.14
150.70
149.02
144.48
137.79
137.52
136.92
132.85
130.88
129.69
128.94
128.65
125.78
125.27
121.94
39.68
f1 (ppm)

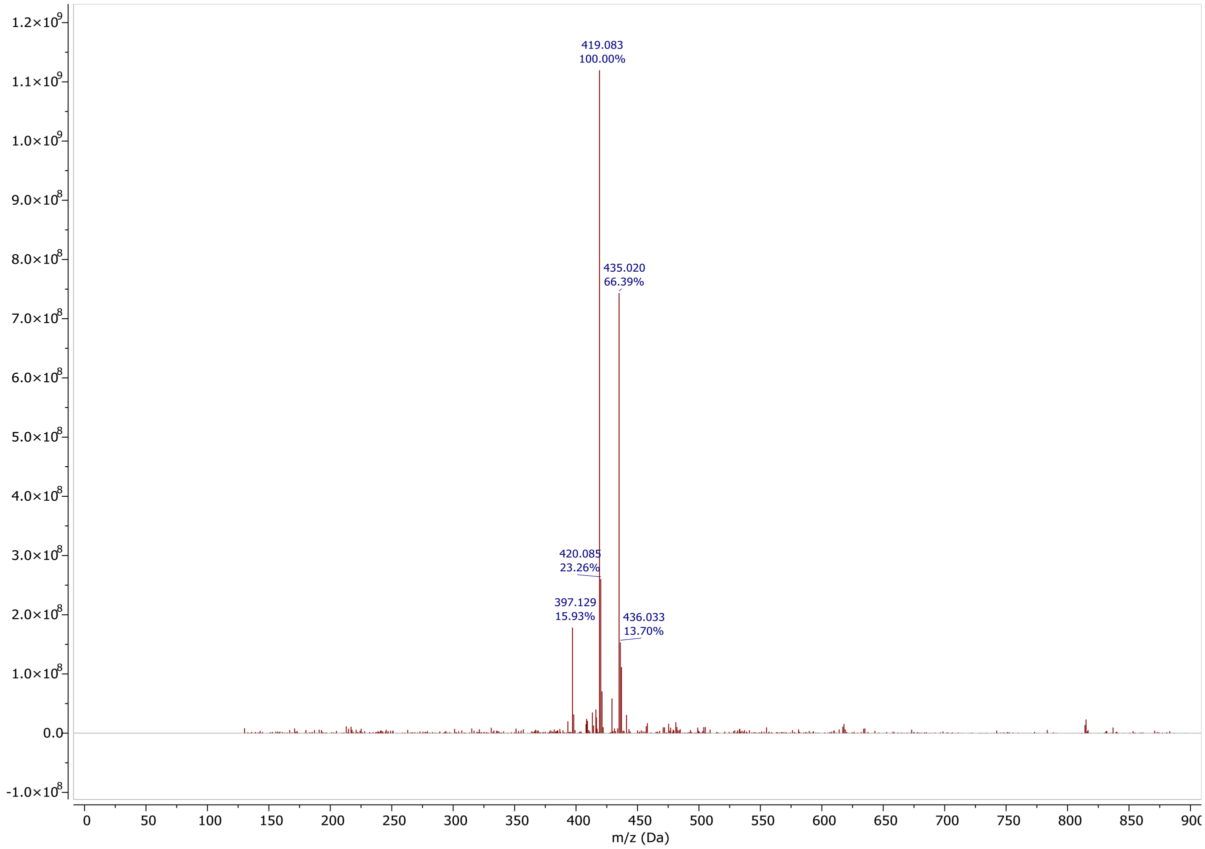
419.083
100.00%
435.020
66.39%
420.085
23.26%
397.129
15.93%
436.033
13.70%
m/z (Da)

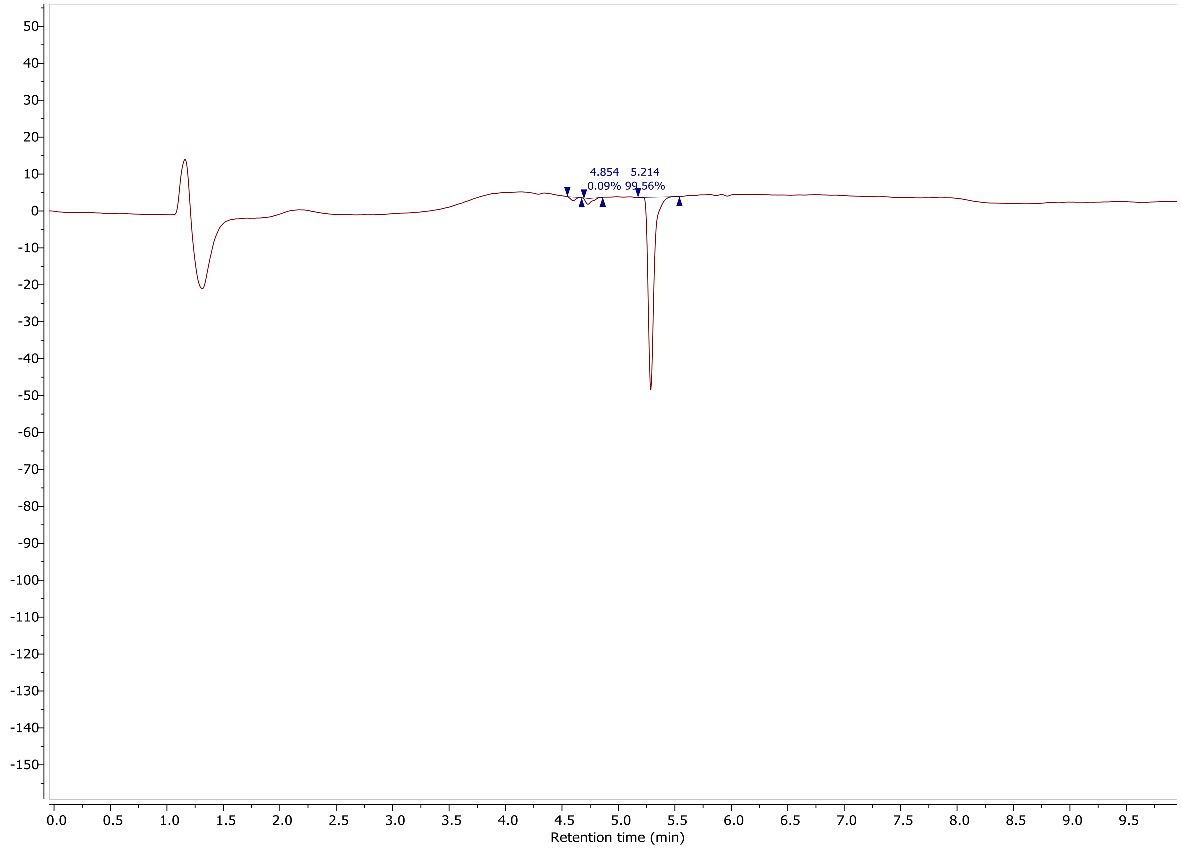
4.854
0.09%
5.214
99.56%
Retention time (min)

8

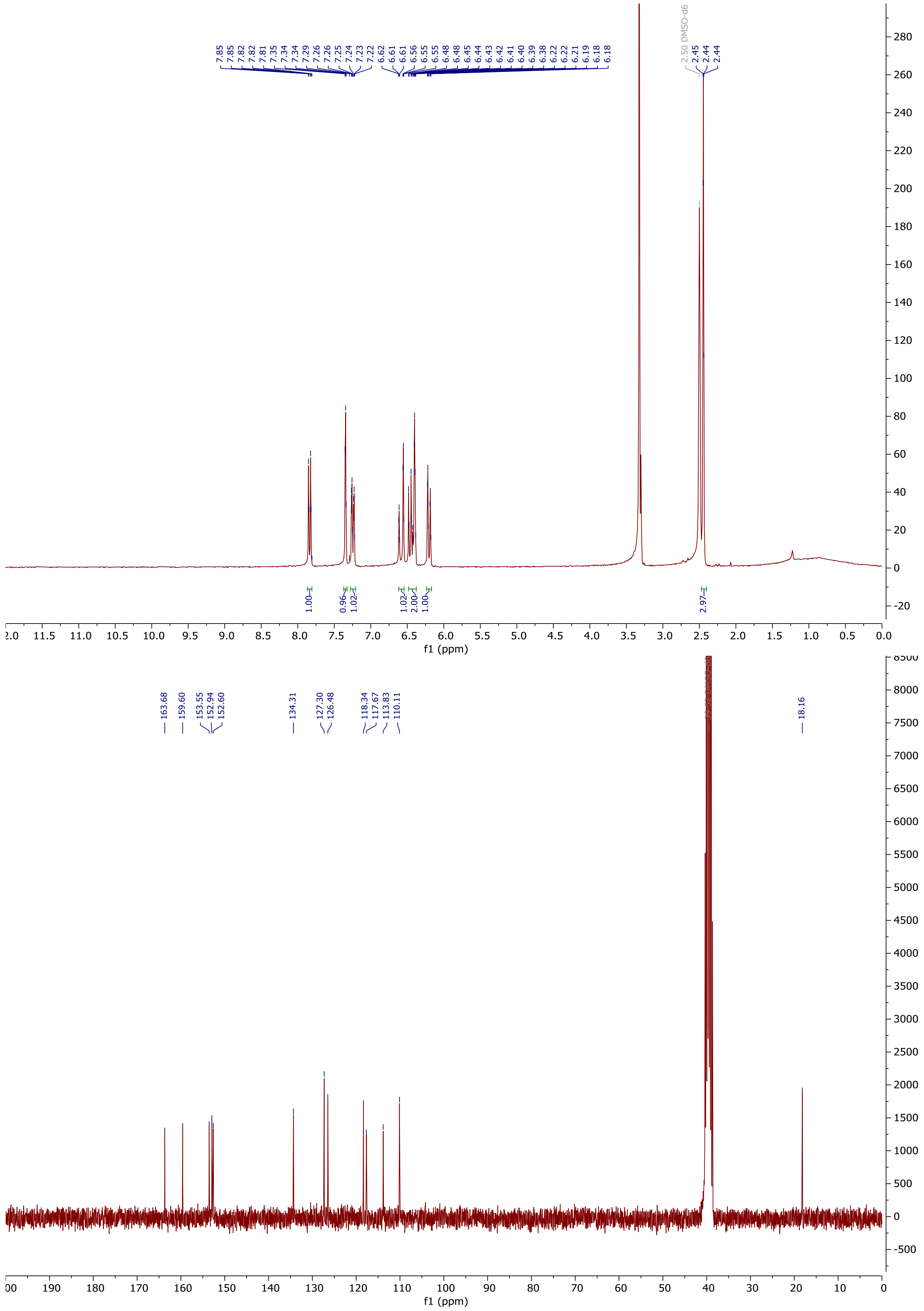
7.85
7.85
7.82
7.82
7.81
7.35
7.34
7.34
7.29
7.26
7.26
7.25
7.24
7.23
7.22
6.62
6.61
6.61
6.56
6.55
6.55
6.48
6.48
6.45
6.44
6.43
6.42
6.41
6.40
6.39
6.38
6.22
6.22
6.21
6.19
6.18
6.18
2.50 DMSO-d6
2.45
2.44
2.44
1.00
0.96
1.02
1.02
2.00
1.00
2.97
f1 (ppm)
163.68
159.60
153.55
152.94
152.60
134.31
127.30
126.48
118.34
117.67
113.83
110.11
18.16
f1 (ppm)

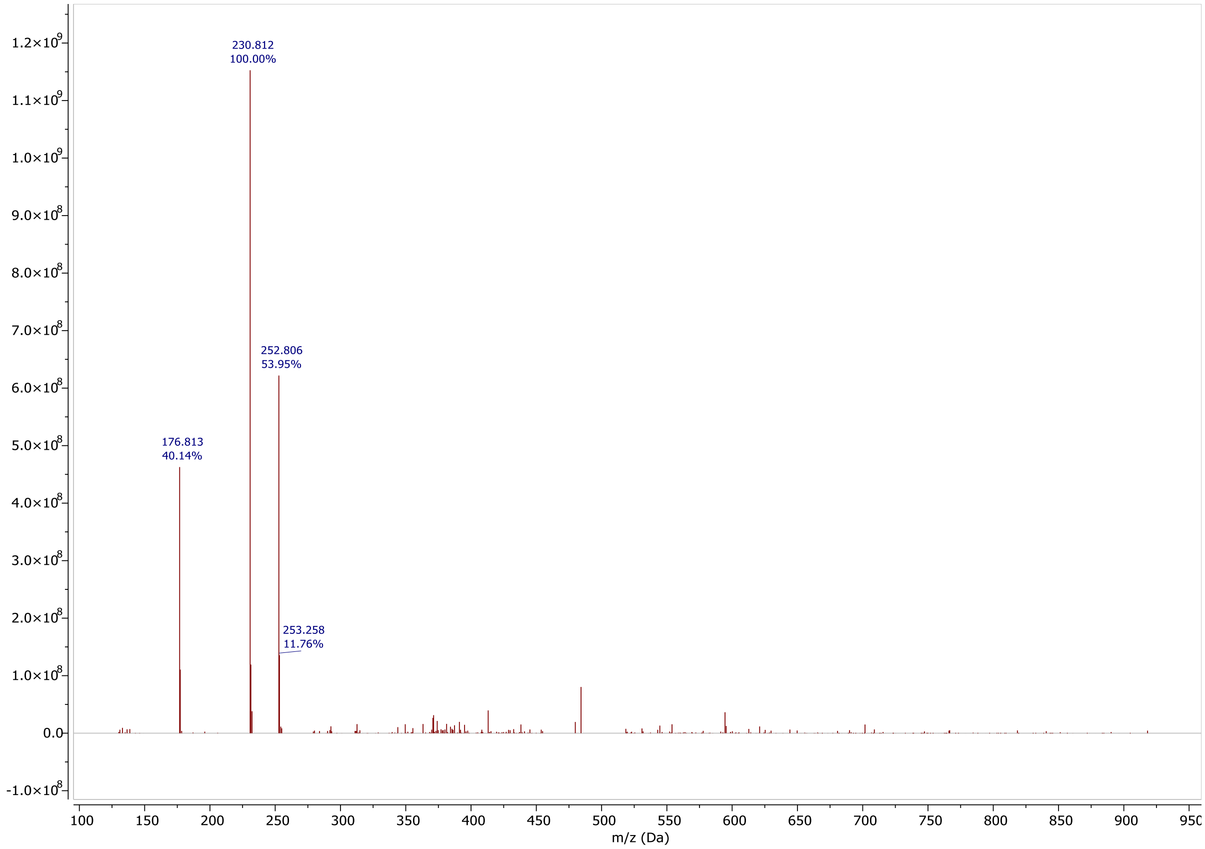
230.812
100.00%
252.806
53.95%
176.813
40.14%
253.258
11.76%
m/z (Da)

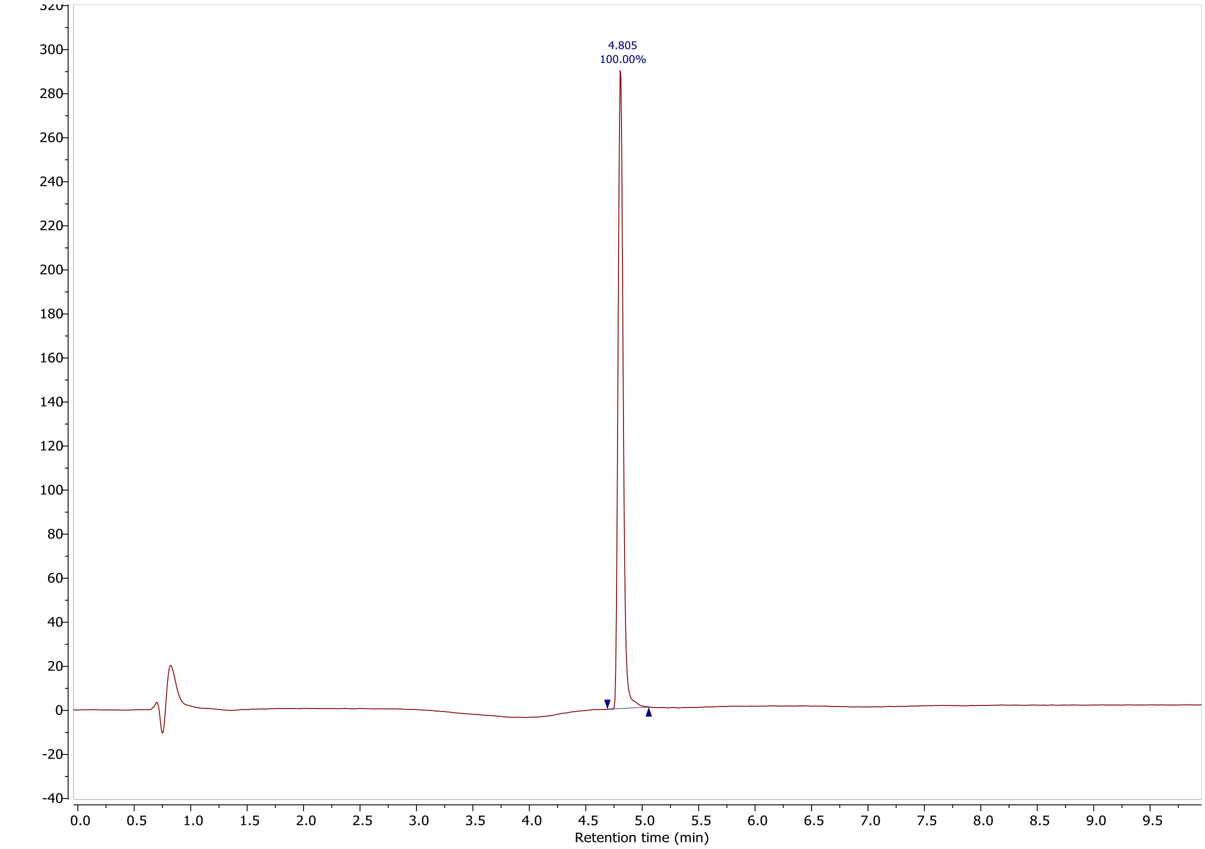
4.805
100.00%
Retention time (min)
